# Sequence-specific fluorescence turn-on arises from base pairing-templated tautomerism in the tricyclic cytidine analogue ^DEA^tC

**DOI:** 10.1039/d5cb00243e

**Published:** 2025-12-07

**Authors:** Ana Shalamberidze, Harrison R. Pearce, Andrew L. Cooksy, Byron W. Purse

**Affiliations:** a Department of Chemistry and Biochemistry, San Diego State University San Diego CA USA bpurse@sdsu.edu

## Abstract

Fluorescent probes for measuring the structure, dynamics, cellular localization, and biochemistry of DNA and RNA are useful for determining the regulatory mechanisms of gene expression. Intrinsically fluorescent, Watson–Crick-capable nucleobase analogues are especially powerful because they can precisely probe desired loci while minimally perturbing native nucleic acid function. Here, we study the fluorescent responses of the tricyclic pyrimidine analogue ^DEA^tC to base pairing with adenine, guanine, and a set of noncanonical nucleobases in duplex DNA oligonucleotides. We find that single-stranded oligonucleotides containing one ^DEA^tC exhibit up to a fivefold fluorescence increase upon hybrid duplex formation and base pairing with G, and a lesser degree of fluorescence turn-on when base pairing with inosine. Other purine nucleobases do not induce significant fluorescence turn-on. Solvent kinetic isotope effect measurements, excitation–emission matrix (EEM) analysis, and spectral comparisons indicate that fluorescence turn-on originates from base pairing-templated tautomerism. The non-emissive T-like form predominates in the single strand and in duplexes paired with A, whereas the emissive C-like tautomer is selectively stabilized upon duplex formation when paired with G. Density functional theory (DFT) calculations further support this tautomeric control model. Although base stacking influences overall brightness, it does not alter the mechanism or specificity of fluorescence turn-on. Modulation of emission through tautomeric control offers a powerful strategy for designing nucleobase analogues with base pairing-specific fluorescence responses.

## Abstract 2

In solution and single-stranded DNA, ^DEA^tC predominantly adopts a weakly emissive thymine-like tautomer, whereas base pairing with guanine or inosine stabilizes its bright cytidine-like tautomer, yielding a sequence-dependent fluorescence increase.

## Graphical abstract

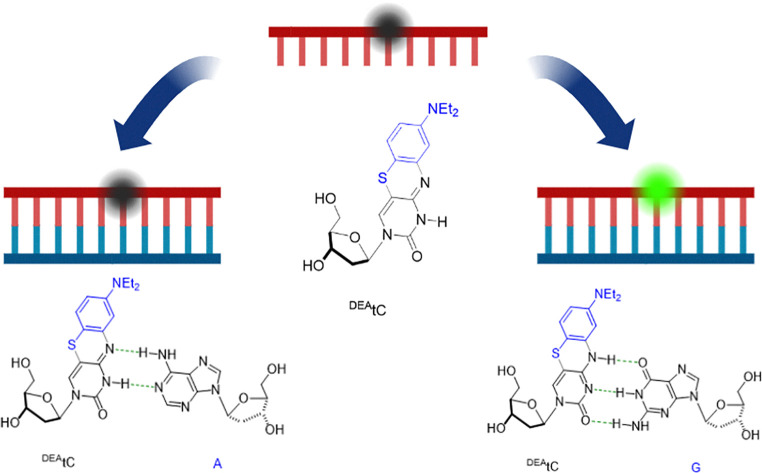

## Introduction

Fluorescent nucleobase analogues (FBAs) are powerful tools for studying the structure, dynamics and chemistry of nucleic acids in isolation, in complexation with other biomolecules, and in biological environments such as cells, tissues, and even whole organisms.^1–10^ Many applications of fluorescently labeled nucleic acids have employed traditional fluorophores such as Alexa Fluors, cyanines, and rhodamines covalently tethered to nucleic acids by flexible linkers, often installed using modified phosphoramidites.^11–15^ Although this approach enables the selection of very bright fluorophores that absorb and emit in nearly any desired window of the optical spectrum, the flexibility of the tethers limits positional precision, allowing the dyes to sample multiple local environments and sometimes disrupt biomolecular interactions or alter cellular localization.^16–20^ Fluorescent nucleobase analogues address these problems by substituting for native nucleobases while retaining Watson–Crick base pairing and, in many cases, preserving the natural, global conformation of A- and B-form helices, G-quadruplexes, i-motifs, and folded RNA structures.^21–29^ By virtue of their direct participation in base pairing and stacking, they can sense and report on both local and longer-range changes in nucleic acid conformation with high precision and sensitivity. Recent applications of FBAs have included studies on riboswitch function, measurements of the activity of base editing enzymes, imaging the uptake of exogenous mRNA and the synthesis, localization, and degradation of RNA in living cells, and the nascent area of single-molecule fluorescence studies on nucleic acids.^5,9,10,28–31^

**Fig. 1:**
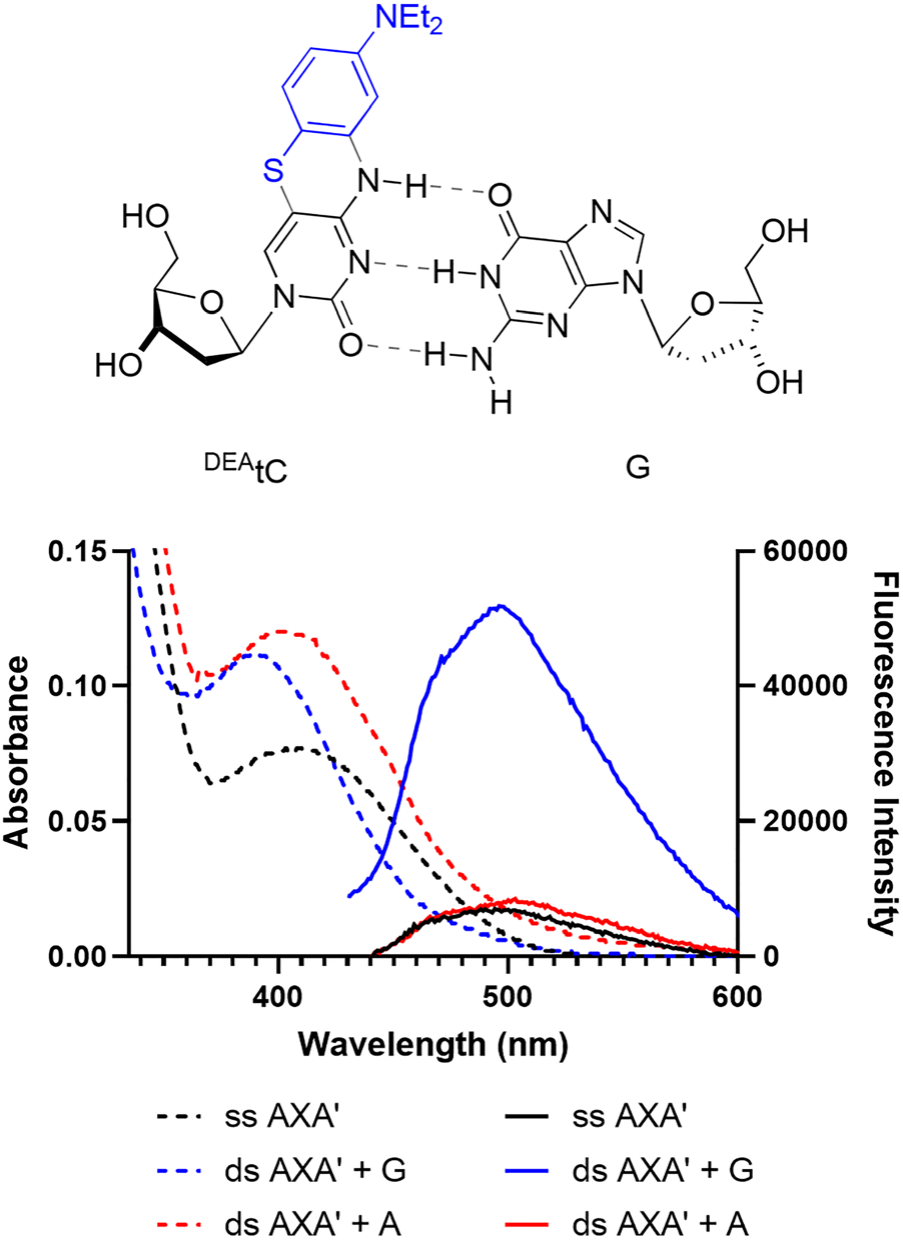
The nucleobase analogue ^DEA^tC, when incorporated into the AXA′ oligonucleotide, exhibits a 5-fold increase in fluorescence intensity when base-paired with guanine, but not when base-paired with adenine (the AXA′ oligonucleotide sequence is shown in Table 1). Absorption (dashed) and emission (solid) spectra were recorded in 1× PBS buffer at pH 7.4 at 37 µM and 0.72 µM, respectively. Emission spectra were collected using excitation at 395 nm.

While advances in the design, synthesis, and applications of FBAs are already enabling discoveries in nucleic acids biology, they are still limited by low brightness and clustering in the blue–green window of the visible spectrum.^21,28^ The design of more red-shifted and brighter FBAs, optionally with the capacity to report on specific changes in their local environment, is a great challenge because it is difficult to understand mechanistically how the local environment—particularly base pairing and stacking—influences the excited state and the competing pathways of radiative and nonradiative relaxation.^22,32–34^ Most FBAs are quenched by base stacking, often due to photoinduced electron transfer (PET), some retain their fluorescence, and a tiny number of known FBAs show a significant fluorescence increase.^26,35–39^ Our group has developed and studied the tricyclic cytidine analogue ^DEA^tC, which is, to the best of our knowledge, the FBA with the greatest known increase in fluorescence intensity upon the formation of matched Watson–Crick base pairs in duplex DNA oligonucleotides and in DNA–RNA heteroduplexes (Fig. 1).^26,27,39^ The fluorescence quantum yield of the ^DEA^tC nucleoside is *Φ*_em_ = 0.006 in 1× PBS buffer at pH 7.4, and this increases up to *Φ*_em_ = 0.12 in matched duplex DNA or *Φ*_em_ = 0.20 in a matched DNA–RNA heteroduplex, *i.e.* when ^DEA^tC is base paired with guanine. The magnitude of fluorescence turn-on depends to some degree on the identity of the stacked, neighboring bases, with greater influence in DNA–DNA than in DNA–RNA. No fluorescence turn-on is observed in ^DEA^tC:A base pairs or when ^DEA^tC is opposite an abasic site. While ^DEA^tC provides a powerful readout for matched base pairing that can distinguish between single nucleobases immediately upon hybridization, the mechanism that explains its specific fluorescence turn-on response is not known and base pairing partners beyond the canonical purines A and G have not been studied. Accordingly, the goal of the present study is to measure the fluorescence turn-on response of ^DEA^tC upon hybridization and base pairing with a broad set of noncanonical nucleobases and to determine this mechanism.

## Results and discussion

**Fig. 2:**
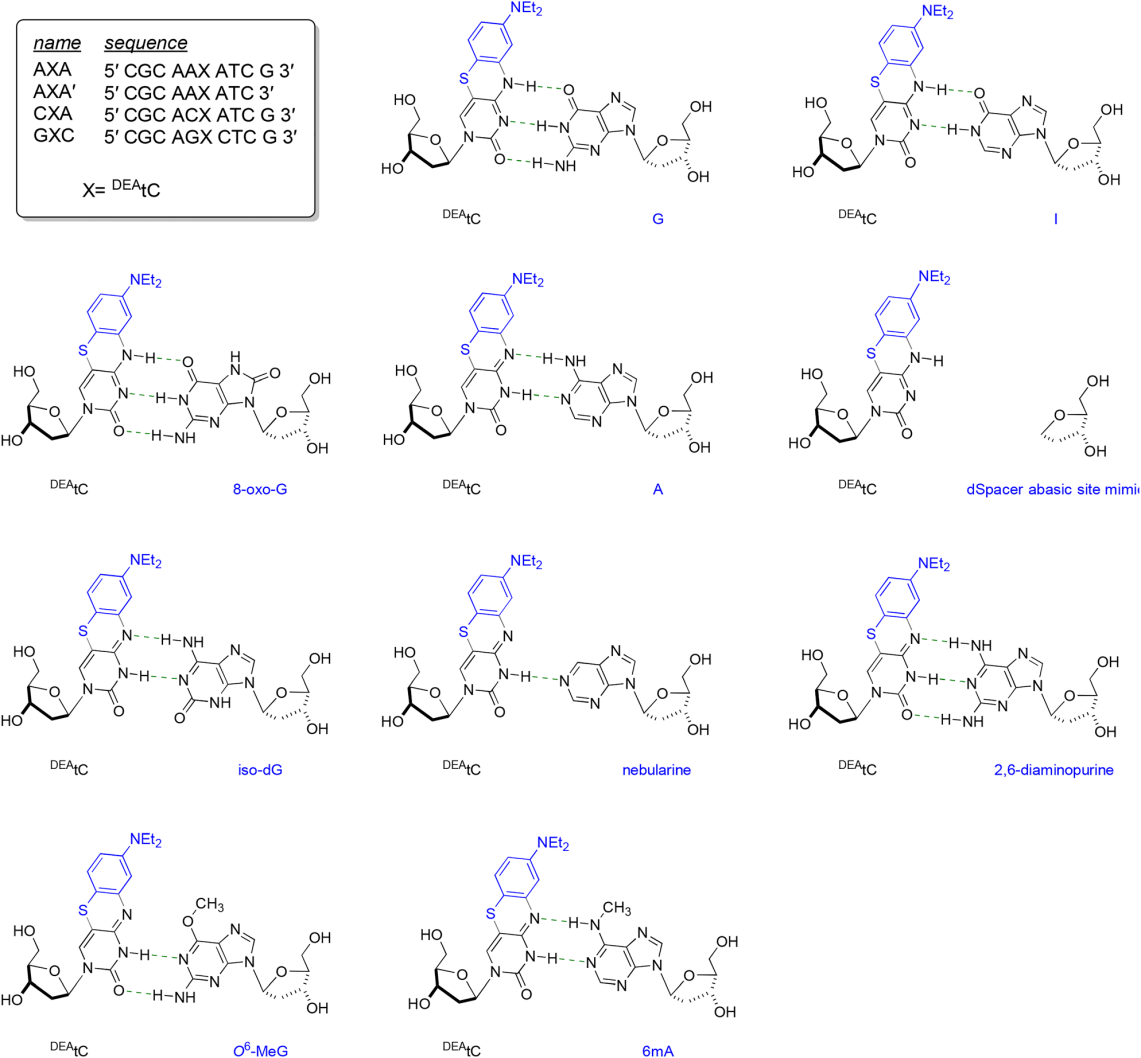
Sequences of the AXA, AXA′, GXC, and CXA oligonucleotides, which contain ^DEA^tC, and the structure of ^DEA^tC base pairs used in this study.

We selected three neighboring nucleobase contexts and 10 base pairing partners in complementary strands to make a comprehensive assessment of how Watson–Crick-like base pairing changes the fluorescence of ^DEA^tC (Fig. 2). The 10-mer DNA oligonucleotide sequences were chosen for consistency with our past studies.^26,27,39^ The rationale for selecting the AXA, GXC, and CXA neighboring base contexts is to cover the broadest range of neighboring base effects previously observed for ^DEA^tC:G base pairs.^26,39^ Past studies have shown that sequence changes further removed from the nearest neighbors of an FBA have little effect on fluorescence, except when those distal changes include additional modified nucleosides well suited for effects such as photoinduced electron transfer (PET) and fluorescence resonance energy transfer (FRET).^40–43^ The GXC sequence was found to be the brightest overall (*Φ*_em_ = 0.12 in dsDNA), the AXA sequences have the greatest magnitude of fluorescence turn-on from single-strand to duplex (from *Φ*_em_ = 0.008 to *Φ*_em_ = 0.042, a 5-fold increase), and the CXA sequence is the least bright and has the smallest degree of fluorescence turn-on (from *Φ*_em_ = 0.014 to *Φ*_em_ = 0.017, a 20% increase). For that reason, we designed this study to focus on nearest neighbor effects. We included the AXA′ sequence, which lacks a 3′-terminal G, to verify that sequence changes distant from the nearest neighbors and expected to affect only overall duplex stability do not significantly alter the fluorescence response. The ^DEA^tC 2′-deoxyribonucleoside and its corresponding DMTr-protected phosphoramidite were synthesized using previously published methods.^26^ The AXA, AXA′, GXC, and CXA oligonucleotides were prepared by solid-phase DNA synthesis and the complementary strands containing canonical and noncanonical nucleobases were synthesized using commercially available amidites (for details, see the SI).

First, we sought to measure the fluorescent response of each of the AXA, AXA′, GXC, and CXA oligonucleotides to hybridization with all 10 complementary strands (Fig. 2 and Tables 1 and 2 and Tables S4 and S5). To ensure the completeness of duplex formation, we started with 0.72 µM solutions of each of these ^DEA^tC-containing oligonucleotides in 1× PBS buffer at pH 7.4 and titrated in up to 2 equivalents of each complimentary sequence, measuring the fluorescence change during the titration (Fig. S1–S4). For all these experiments, we observed saturation of the fluorescence change at nearly a 1 : 1 ratio of ^DEA^tC-containing strand to its complement, with slight variations in this ratio attributable to random experimental error. Compared with AXA, the AXA′ sequence required slightly more complementary strand to reach saturation, but no significant differences were observed in its absorption, emission, or fluorescence turn-on responses, consistent with our expectation (Fig. S2 and S4 and Fig. S10–S13). Further addition of the complementary strands beyond the 1 : 1 ratio resulted in little change in fluorescence. As expected for these sequences, an equilibrium of matched duplex formation is attained at ambient temperature; the use of an annealing protocol resulted in no significant differences.

**Table 1:** Steady-state fluorescence measurements of ^DEA^tC in single- and double-stranded DNA oligonucleotides for the AXA′ sequence 5′-CGCAAXATC-3′, where X = ^DEA^tC. Absorption and emission measurements were recorded in 1× PBS buffer at pH 7.4 at 37 µM and 0.72 µM, respectively. Emission spectra were collected using excitation at 395 nm

Sequence name	*λ*_max_ Absorption/nm	*λ*_max_ Excitation/nm	*λ*_max_ Emission/nm	Fluorescence intensity change^a^
ss AXA′^b^	409	386	496	n/a
ds G^c^	390	397	494	5.53
ds A	401	395	494	1.34
ds I	391	394	493	3.44
ds 8oxoG	392	396	495	1.66
ds dSpacer	414	389	494	0.84

a Fluorescence intensity change is reported as the ratio of the integrated emission intensity of each duplex with respect to ss AXA′ using 395 nm excitation.

b ss = single-stranded.

c ds G refers to the matched complementary duplex with ^DEA^tC base paired with G; other duplex names follow this convention.

**Table 2:** Steady-state fluorescence measurements of ^DEA^tC in single- and double-stranded DNA oligonucleotides for the GXC sequence 5′-CGCAGXCTCG-3′, where X = ^DEA^tC. Absorption and emission measurements were recorded in 1× PBS buffer at pH 7.4 at 37 µM and 0.72 µM, respectively. Emission spectra were collected using excitation at 395 nm

Sequence name	*λ*_max_ Absorption/nm	*λ*_max_ Excitation/nm	*λ*_max_ Emission/nm	Fluorescence intensity change^a^
ss GXC^b^	409	396	499	n/a
ds G^c^	390	396	499	5.42
ds A	415	397	500	0.72
ds I	391	396	499	3.24
ds 8oxoG	392	397	498	1.53
ds dSpacer	416	397	498	0.32

a Fluorescence intensity change is reported as the ratio of the integrated emission intensity of each duplex with respect to ss GXC using 395 nm excitation.

b ss = single-stranded.

c ds G refers to the matched complementary duplex with ^DEA^tC base paired with G; other duplex names follow this convention.

**Fig. 3:**
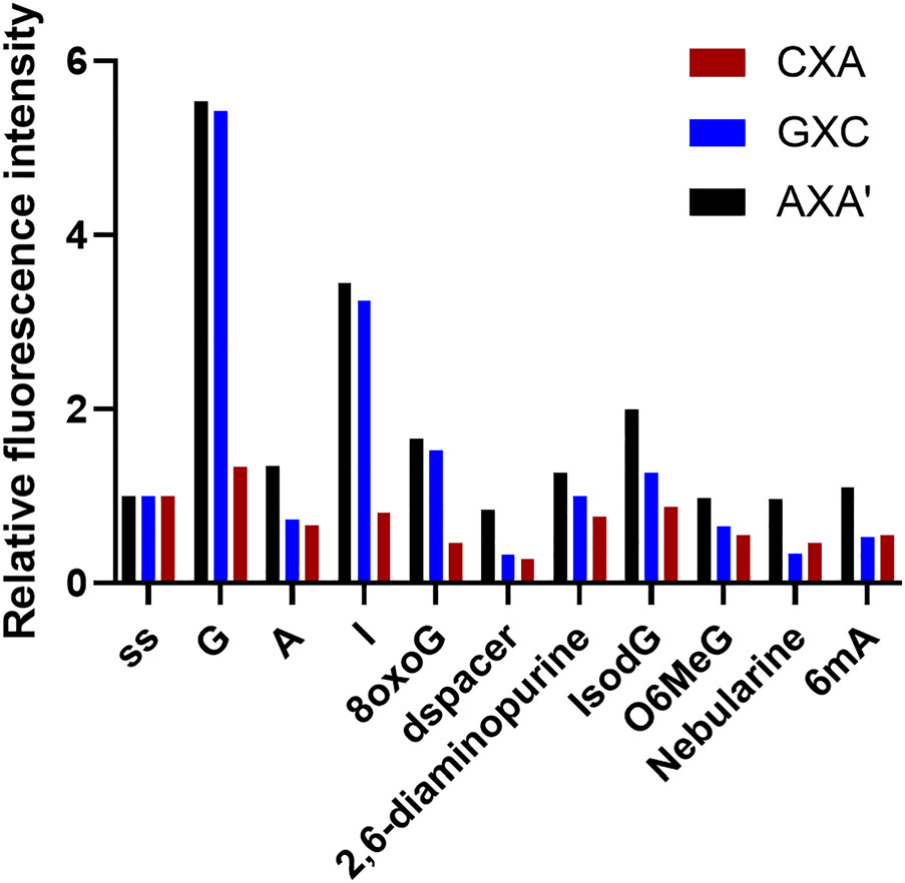
Relative fluorescence intensity of the single-stranded (ss) CXA, GXC, and AXA′ oligonucleotides and their hybrid duplexes with G, A, and noncanonical nucleobases. Fluorescence intensity change is reported as the ratio of the integrated emission intensity of each duplex with respect to the ^DEA^tC-containing single strand, using 395 nm excitation.

The resulting data shows that ^DEA^tC's fluorescence turn-on response is unique to base pairing with guanine-like partners that would be expected to favor Watson–Crick base pairing with a cytosine-like tautomer of ^DEA^tC (Fig. 3 and Fig. S10). Fluorescence turn-on is greatest for base pairing with guanine, as previously observed, and we detect a significant but lesser degree of fluorescence turn-on in ^DEA^tC:inosine base pairs, which lack the exocyclic amino group N2 of guanine. 8-Oxo-G has the same Watson–Crick edge as guanine, but does not induce a fluorescence turn-on when base-paired with ^DEA^tC. These results are consistent for the AXA′, AXA, GXC, and CXA sequences, with the latter showing only small fluorescence changes. Other base pairing partners, which include those expected to favor a thymine-like tautomer of ^DEA^tC, the formation of wobble base pairs, or, like nebularine, have greatly reduced capacity to form hydrogen bonds, show no significant fluorescence turn-on, a result also observed when ^DEA^tC is present opposite an abasic site.

**Fig. 4:**
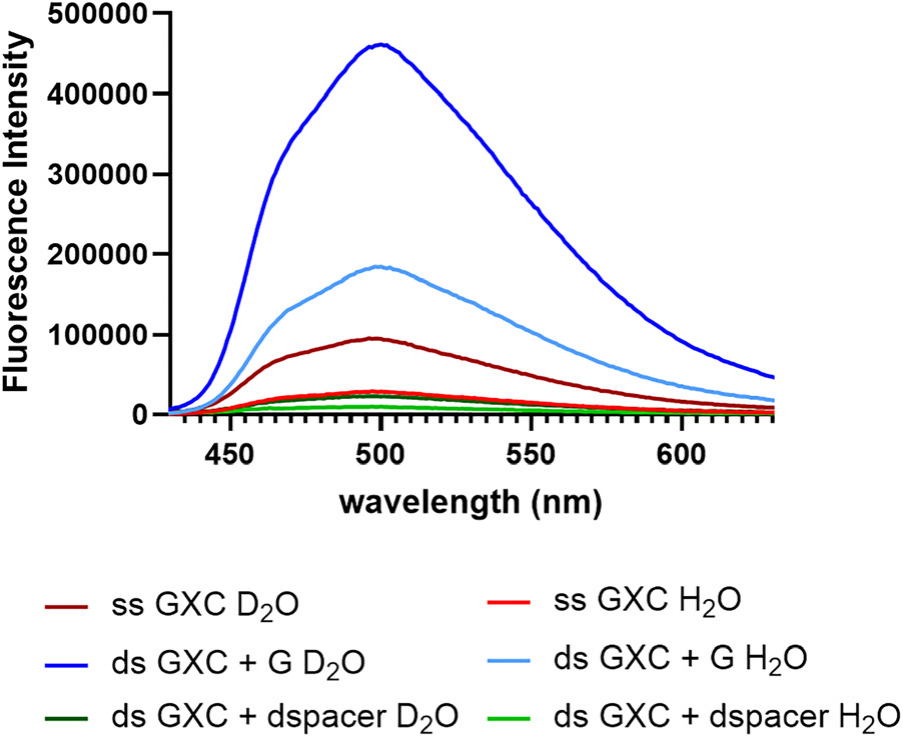
Fluorescence intensity comparison of the GXC sequence (0.72 µM) as a single-stranded oligonucleotide and in hybrid duplexes with ^DEA^tC base paired with G or opposite the dSpacer abasic site mimic in normal (H_2_O; light colors) *vs.* deuterated (D_2_O; dark colors) 1× PBS buffer, pH 7.4. Excitation was at 395 nm.

Having observed that ^DEA^tC's fluorescence turn-on is unique to base pairing with certain G-like partners, we next sought to determine the mechanism that would explain this specificity. Our past structural determination work using ^1^H NMR spectroscopy has shown that duplex DNA oligonucleotides containing a single substitution of ^DEA^tC for C retain the normal B-form conformation and that ^DEA^tC engages in Watson–Crick base pairing and base stacking similar to that of cytosine, but with extended π stacking.^39^ Recognizing that the fluorescence quantum yield of ^DEA^tC is lowest as a free nucleoside and that engagement in a specific configuration of Watson–Crick-edge hydrogen bonding is crucial, we first hypothesized that desolvation and hydrogen bonding of ^DEA^tC in the duplex protects this FBA from excited-state proton transfer (ESPT), a prominent fluorescence quenching mechanism, thereby inducing turn-on. To test this hypothesis, we measured the influence of buffer deuteration on the fluorescence intensity of the single-stranded GXC oligonucleotide and the hybrid duplexes with ^DEA^tC base paired with G or opposite the dSpacer abasic site mimic, a 1′,2′-dideoxyribose (Fig. 4). For these experiments, we measured the fluorescence intensity of the single-stranded and duplex oligonucleotides in replicate, using 1× PBS buffer at pH 7.4 and an equivalent buffer prepared in D_2_O. We found that the brightness in deuterated buffer ÷ the brightness in regular buffer = 3.23 for the GXC ssDNA oligonucleotide. Because buffer deuteration slows the rate of ESPT, a kinetic isotope effect, we conclude that ESPT is a significant quenching mechanism of ^DEA^tC's fluorescence in the GXC oligonucleotide. When repeating these experiments using the GXC duplexes with ^DEA^tC:G and ^DEA^tC:dSpacer base pairs, respectively, we found that the fluorescence intensity ratio for samples in deuterated *vs.* regular buffer ratio is 2.40 for the former and 2.20 for the latter. Duplex formation attenuates the rate of quenching by ESPT, but this attenuation does not depend on base pairing. Accordingly, the hypothesis is disproved. The specificity of ^DEA^tC's fluorescence turn-on is not the result of the special ability of a G:C-like configuration of Watson–Crick hydrogen bonding to slow the rate of ESPT.

Desolvation of ^DEA^tC when base paired and stacked is, by itself, an insufficient mechanism to explain the specificity of fluorescence turn-on, because one would expect a similar degree of desolvation in many of the base pairing configurations of ^DEA^tC that do not exhibit fluorescence turn-on (Fig. 3).

**Fig. 5:**
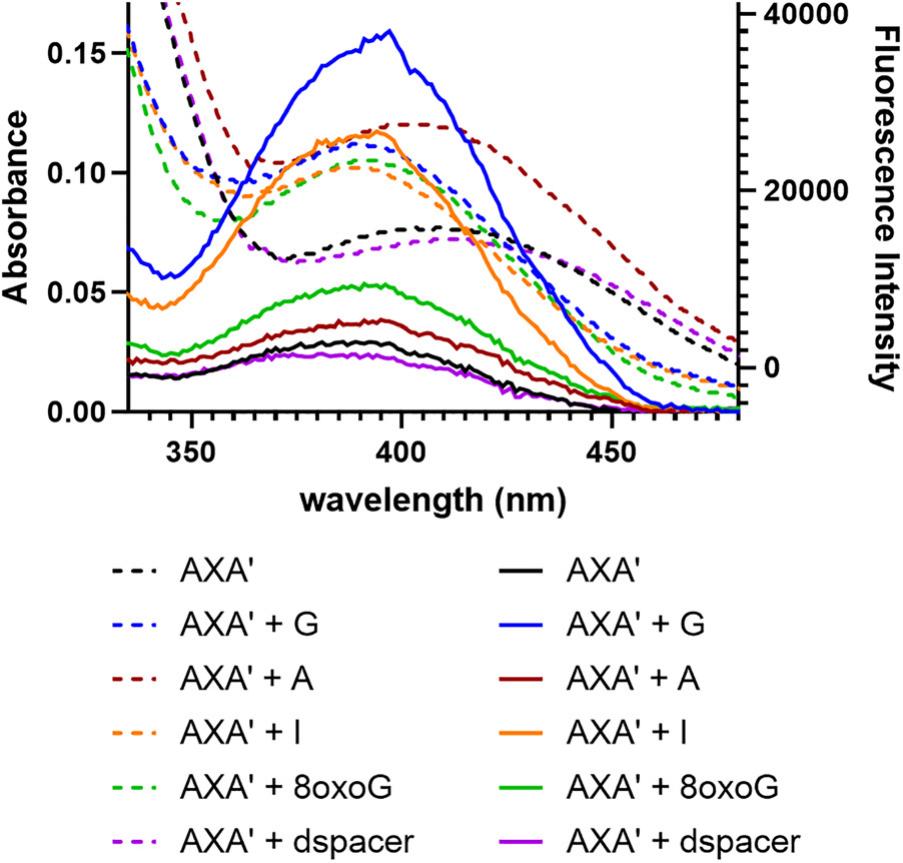
Absorption (dashed) and excitation (solid; collected using an emission wavelength of 500 nm) of the single-stranded (ss) AXA′ oligonucleotide and hybrid duplexes with ^DEA^tC base paired with G, A, I, 8-oxo-G, and dSpacer (1′,1′-dideoxyribose). Absorption and excitation spectra were recorded in 1× PBS buffer at pH 7.4 at 37 µM and 0.72 µM, respectively. Excitation spectra were collected by monitoring emission at 500 nm.

Next, we hypothesized that tautomerism of ^DEA^tC might explain this specificity. ^DEA^tC is the diethylamino derivative of tC, a well characterized fluorescent cytidine analogue that has nearly the same fluorescent brightness as a free nucleoside, in single-stranded, and in duplex oligonucleotides.^44^ While we have previously reported ^DEA^tC's fluorescence turn-on response upon hybrid duplex formation,^26,27,39^ its tautomerism has not yet been experimentally assessed. Tautomerism is established as a significant determinant of the photophysical properties of some fluorescent nucleobase analogues,^22,45,46^ and although a reported ABN study revealed a modest base pairing-templated tautomerism effect in duplex DNA,^29^ to our knowledge it has not been shown to serve as the dominant mechanism driving fluorescence changes in duplex oligonucleotides. A previous study examined the incorporation of d(tC)TP and d(tC^O^)TP, the triphosphate forms of the related cytidine analogues tC and tC^O^, by human DNA polymerase α and the Klenow fragment.^47^ These reactions showed increased misincorporation opposite adenine, attributed to the greater tendency of tC and tC^O^ to interconvert between tautomers compared with natural cytosine. To investigate how tautomerism of ^DEA^tC might contribute to its fluorescence turn-on specificity, we compared its absorption and excitation spectra as a function of hybridization (Fig. 5). These measurements are informative because the absorption spectrum reflects all species with appreciable extinction coefficients at each wavelength, whereas the excitation spectrum isolates the subset of those species that are emissive at the monitored wavelength. Accordingly, significant differences between absorption and excitation spectra indicate the presence of multiple ground-state species, only some of which are fluorescent (or, more properly, exhibit differing fluorescence) upon excitation.

We recorded absorption and excitation spectra for the AXA′ sequence as a single-stranded oligonucleotide and in hybrid duplexes with G, A, I, 8-oxo-G, and dSpacer (1′,2′-dideoxyribose). The excitation spectra were collected using 500 nm as the emission wavelength, close to *λ*_max_ for the fluorescence. By plotting these spectra together, clear differences are observed, depending on ^DEA^tC's base pairing partner (data for the AXA′ sequence are shown in Fig. 5; very similar data are observed for GXC as shown in Fig. S5). First, we note that all excitation spectra have a maximum near 395 nm and a shoulder at 370 nm, with the exception of AXA′:dSpacer, which is slightly more absorbing at 370 nm and less absorbing at 395 nm. The maximum at 395 nm—and the overall appearance of the excitation spectra—matches the absorption spectra when ^DEA^tC is base paired with G, I, and 8-oxo-G, all configurations that are expected to strongly reinforce ^DEA^tC's C-like hydrogen bonding configuration in Watson–Crick base pairing. However, the absorption and excitation spectra do not match for the single-stranded AXA′ oligonucleotide and in hybrid duplexes wherein ^DEA^tC is base paired with A and dSpacer, configurations that would favor ^DEA^tC's T-like hydrogen bonding configuration in Watson–Crick base pairing or would not template its tautomerism, respectively. Notably, both these hybrid duplexes have less fluorescence intensity than the single-stranded AXA′ oligonucleotide and *λ*_max,abs_ is red-shifted by 9 nm and 18 nm, respectively, without an accompanying change in *λ*_max_ for excitation. Rather, the excitation spectra closely resemble those observed when ^DEA^tC is base paired with G.

These results can be explained by a model wherein ^DEA^tC's tautomerism, which is templated by base pairing, is the primary control of its fluorescence. When ^DEA^tC is base paired with G or inosine, its cytosine-like tautomer is stabilized and is the predominant species present. The absorption and excitation spectra match, and the greatest fluorescent brightness is observed. In contrast, when ^DEA^tC is base paired with A, the thymine-like tautomer is templated and is the major species present, which is indicated by the red-shifted *λ*_max,abs_. This tautomer is nearly non-emissive. A minor population of the C-like tautomer persists, explaining the retention of an excitation spectrum similar to those observed when ^DEA^tC is base paired with G or inosine, and a low degree of residual fluorescence. The single-stranded AXA′ oligonucleotide has a similar mismatch between its absorption and emission spectra with the former red-shifted, indicating that the T-like tautomer is favored and explaining the low *Φ*_em_ = 0.008.^26^

**Fig. 6:**
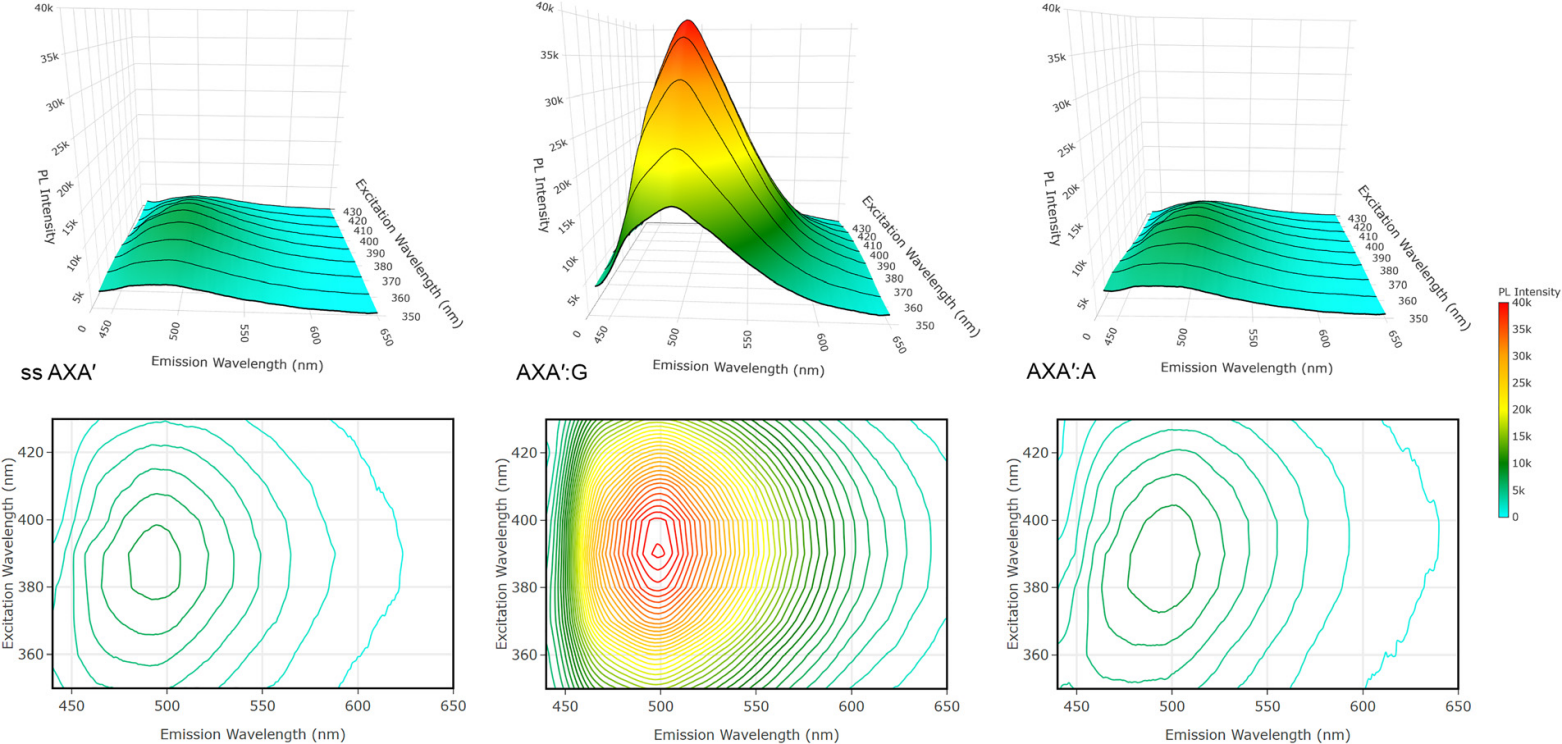
Excitation–emission matrix plots for single-stranded AXA′, the AXA′:G duplex, and the AXA′:A duplex. Data were collected in 1× PBS buffer at pH 7.4 at and 0.72 µM AXA′ strand.

To validate this model, we recorded emission spectra across excitation wavelengths from 350–450 nm and constructed excitation–emission matrices (EEMs) for the single-stranded AXA′ oligonucleotide, the AXA′:A duplex, and the AXA′:G duplex (Fig. 6, shown as both 3D surface and contour plots; complementary 2D plots and data for AXA are shown in the Fig. S12 and S13). EEM analysis sensitively reveals the presence of multiple emissive species and how their relative populations shift in response to environmental changes, in this case those arising from base pairing. Although quantitative deconvolution is limited by spectral overlap between the C- and T-like tautomers of ^DEA^tC, the EEMs clearly show tautomeric shifts induced by base pairing. The single-stranded AXA′ construct exhibits low overall fluorescence, with a main emission at 499 nm (C-like tautomer) and a distinct shoulder at 463 nm (T-like tautomer). In this unpaired state, the non-emissive T-like tautomer predominates, but the minor C-like species dominates the observed spectrum owing to its much higher intrinsic fluorescence. Upon duplex formation with adenine, base pairing stabilizes and templates the T-like tautomer, enhancing the 463 nm feature and producing stronger contours in the lower-left quadrant of the EEM plot. In contrast, base pairing with guanine templates the emissive C-like tautomer and suppresses the T-like tautomer almost completely, resulting in the pronounced fluorescence enhancement characteristic of ^DEA^tC turn-on.

This model of tautomerism-controlled fluorescence turn-on explains the fluorescent response to all base pairing partners except 8-oxo-G, which templates the C-like tautomer through Watson–Crick base pairing but does not elicit a fluorescence turn-on. The templating of the C-like tautomer is indicated by the matched absorption and excitation spectra with *λ*_max_ = 395 nm, and in this special case the lack of a significant fluorescence turn-on is the result of quenching by photo-induced electron transfer (PET). 8-Oxo-G has been previously reported to be a potent quencher of other FBAs by this mechanism, which is enabled by its electron deficiency as compared with guanine.^48,49^ Inosine is less able to stably template the C-like tautomer of ^DEA^tC because its base pair lacks a hydrogen bond; the fluorescence turn-on is accordingly attenuated. The other base pairing partners studied favor the T-like tautomer of ^DEA^tC, explaining the low fluorescence emission in these hybrid duplexes.

**Fig. 7:**
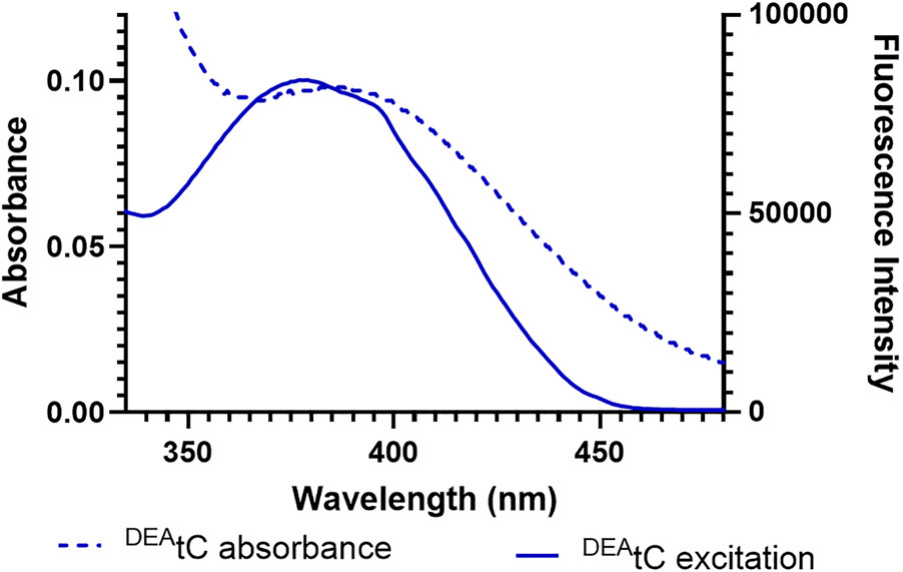
Absorption (dashed) and excitation (solid; collected using an emission wavelength of 500 nm) of the ^DEA^tC 2′-deoxyribonucleoside in Milli-Q water. *λ*_max,abs_ = 377 nm and *λ*_max,ex_ = 385 nm.

The ^DEA^tC nucleoside is similarly non-emissive in Milli-Q water and 1× PBS buffer at pH 7.4. Although both spectra are blue-shifted with respect to those observed in single-stranded and duplex oligonucleotides, a discrepancy is again observed between the absorption and excitation spectra, with the latter blue-shifted as compared with the former (Fig. 7). The low fluorescence emission of the ^DEA^tC nucleoside can be explained by significant adoption of the T-like tautomer in aqueous solution and its greater solvation as compared with the environment in oligonucleotides. The relative blue-shift of its absorption and excitation spectra with respect to those observed in oligonucleotides indicates that base stacking stabilizes the excited state relative to the ground state.

Computational studies further support this model of fluorescence turn-on. We carried out B3LYP-D3(BJ)/cc-pVDZ/SMD^50–56^ geometry optimizations of ^DEA^tC base paired with guanine, inosine and adenine using an AXA trimer duplex constrained in the normal B-form geometry, with water as the implicit solvent. Following geometry optimization, we truncated the base pairs at the 1′-carbon, replacing the phosphoribose backbone with hydrogens, frozen in position. Absorption spectra were predicted with the TD-CAM-B3LYP^57^ method and cc-pVDZ, aug-cc-pVDZ, and aug-cc-pVTZ basis sets. Relative B3LYP energies of the monomeric C-like and T-like tautomers differed by less than 0.1 kcal mol^−1^ when expanding the basis set from aug-cc-pVDZ to aug-cc-pVTZ basis sets, so B3LYP/aug-cc-pVDZ calculations were used for single-point energy calculations of the relative ground state energies in the dimer and base stacked systems. Similarly, absorption wavelengths of the monomers differed by less than 1 nm between the aug-cc-pVDZ and aug-cc-pVTZ basis sets. While B3LYP has been a robust method for geometry optimizations, the long-range corrections present in the CAM-B3LYP functional make CAM-B3LYP a more accurate method over a wide range of electronic transitions.^58,59^

Comparing the electronic energies of the two tautomers, the C-like tautomer is calculated to be more stable than the T-like tautomer even as a monomer, but only by 5 kcal mol^−1^. An exhaustive benchmark study of DFT calculations found an RMSD of 5.50 kcal mol^−1^ for B3LYP isomerization energies,^60^ and it is unsurprising that the experimental evidence indicates that the T-like free nucleoside is more stable than the C-like. Upon formation of the base-paired complexes with inosine, our calculations predict a substantial 28 kcal mol^−1^ stabilization of the C-like tautomer relative to T-like, well outside the expected error range and strongly supporting the hypothesis that base-pairing preferentially templates the ^DEA^tC to the C-like form. We could not make a comparable analysis for base pairing with guanine because its stronger templating capacity caused self-consistent field (SCF) convergence problems when modeling the base pair between the T-like tautomer of ^DEA^tC and guanine.

The wavelengths of the trimer absorption spectra are predicted to be much shorter than experimentally observed (Table 3), but the calculated wavelength differences between C-like and T-like tautomer complexes replicate the significant red-shift observed in experiment when comparing C-like forms (342–356 nm) to T-like forms (364–378 nm). Although superior methods will be needed to more accurately predict the spectra quantitatively, these results again indicate that base-pairing with G is templating the ^DEA^tC into the C-like tautomer.

**Table 3:** CAM-B3LYP/aug-cc-pVDZ/SMD//B3LYP/cc-pVDZ calculated maximum absorption wavelengths for ^DEA^tC

Configuration	Calculated *λ*_max,abs_/nm for the C-like tautomer	Calculated *λ*_max,abs_/nm for the T-like tautomer
^DEA^tC nucleobase	342	378
^DEA^tC in single-stranded AXA	356	378
^DEA^tC in AXA and base paired with G	356	—
^DEA^tC in AXA and base paired with I	353	364
^DEA^tC in AXA and base paired with A	—	377

## Conclusions

In this study, we examined how the fluorescent nucleobase analogue ^DEA^tC responds to base pairing with a series of purine partners in duplex DNA oligonucleotides. The strongest response is to base-pairing with G, which affords up to a 5-fold fluorescence turn-on as compared with the ^DEA^tC-containing single-stranded oligonucleotide. Inosine induces up to a 3-fold fluorescence turn-on and there is little response to base pairing with other purines. Solvent kinetic isotope effects show that excited-state proton transfer is a significant quenching mechanism of ^DEA^tC, which limits its brightness, but this mechanism is not influenced by base pairing and its attenuation in duplexes cannot explain the selectivity and magnitude of fluorescence turn-on. Instead, drawing on differences revealed by absorption and excitation spectra together with excitation–emission matrix analysis, and supported by DFT calculations, we find that ^DEA^tC's low fluorescence as a free nucleoside arises from its predominantly thymine-like tautomeric state. Base pairing in hybrid duplexes can shift this equilibrium. When paired with guanine or inosine, the cytosine-like, emissive tautomer is favored, producing fluorescence turn-on. The effect is stronger with guanine because its triply hydrogen-bonded base pair with ^DEA^tC provides a more powerful tautomeric templating influence. In contrast, base pairs that stabilize ^DEA^tC's T-like tautomer exhibit low fluorescence. Only the absence of fluorescence induction by 8-oxo-G falls outside this framework, though it can be accounted for by the well-known efficiency of 8-oxo-G as a PET quencher. In addition, the computational model accurately predicts both the enhanced stability of the C-like tautomer upon base pairing with guanine and inosine and the blue-shift of the C-like monomer absorption spectrum relative to the T-like tautomer. Accordingly, ^DEA^tC's fluorescence turn-on property is a highly specific result of templated tautomerism induced by base pairing. This fluorescence turn-on mechanism complements the more-studied approach to induced fluorescence turn-on in duplex nucleic acids, wherein the fluorophore is rigidified and bond rotation is restricted by local structure.^38,61^ In our opinion, further development of nucleoside analogues designed for fluorescence turn-on by induced tautomerism presents an exciting opportunity to impart useful probing capabilities to this powerful class of molecular sensors.

## Author contributions

AS: methodology, visualization, writing – review & editing; HRP: formal analysis, methodology, visualization, writing – review & editing; ALC: conceptualization, formal analysis, supervision, validation, writing – review & editing; BWP: conceptualization, data curation, methodology, project administration, resources, supervision, validation, writing – original draft, writing – review & editing.

## Conflicts of interest

There are no conflicts to declare.

## Supplementary Material

CB-007-D5CB00243E-s001

CB-007-D5CB00243E-s002

## Data Availability

The data supporting the findings of this study are available within the paper and its supplementary information (SI). Supplementary information: experimental procedures, sequences of oligonucleotides, fluorescence titration data, absorption/excitation/emission spectra, supporting tables and figures, and computational methods with optimized geometries and calculated absorption data. See DOI: https://doi.org/10.1039/d5cb00243e. Output files from the computational work are available at the ioChem-BD database DOI: https://doi.org/10.19061/iochem-bd-6-576.
