# Supplementary material for: Sequence-specific fluorescence turn-on arises from base pairing-templated tautomerism in the tricyclic cytidine analogue DEAtC
Source: RSC Chem Biol. 2025 Dec 7;7(2):216–25. doi: 10.1039/d5cb00243e (PMC12690291; doi:10.1039/d5cb00243e)
Supplement: CB-007-D5CB00243E-s001 [file CB-007-D5CB00243E-s001.pdf]

Supplementary Information (SI) for RSC Chemical Biology.
This journal is © The Royal Society of Chemistry 2025

Integrated DNA Technologies

Analytical ESI-MS Report

The oligonucleotides containing D-spacer, iso-dG, 2,6-diaminopurine, inosine, and canonical bases were prepared by Integrated DNA Technologies, Inc. (Coralville, IA) and analyzed by HPLC-MS in the following spectra.

Sales Order:	20000529
Reference ID:	468502500
Manufacturing ID:	71520181

Instrument:	SD-LTQ-06	Operator ID:	3598860
Acquired:	1/16/2024 7:11 PM	Reviewed:	1/16/2024 9:14 PM

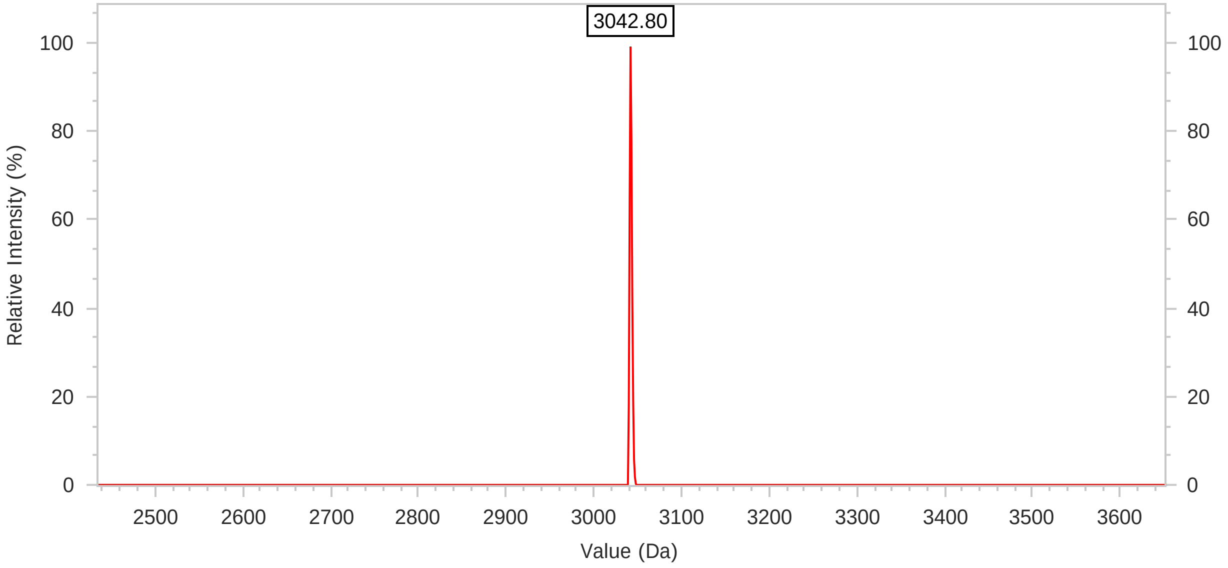

Sequence Name:	AS004-AXA-mismatch
Sequence:	5'- CGA TAT TGC G -3'
Calculated Molecular Weight:	3043.0
Measured Molecular Weight:	3042.80

Integrated DNA Technologies

Analytical ESI-MS Report

Sales Order: 20000529
Reference ID: 468502501
Manufacturing ID: 71520180

Instrument: SD-LTQ-06
Acquired: 1/16/2024 7:11 PM
Operator ID: 3598860
Reviewed: 1/16/2024 9:14 PM

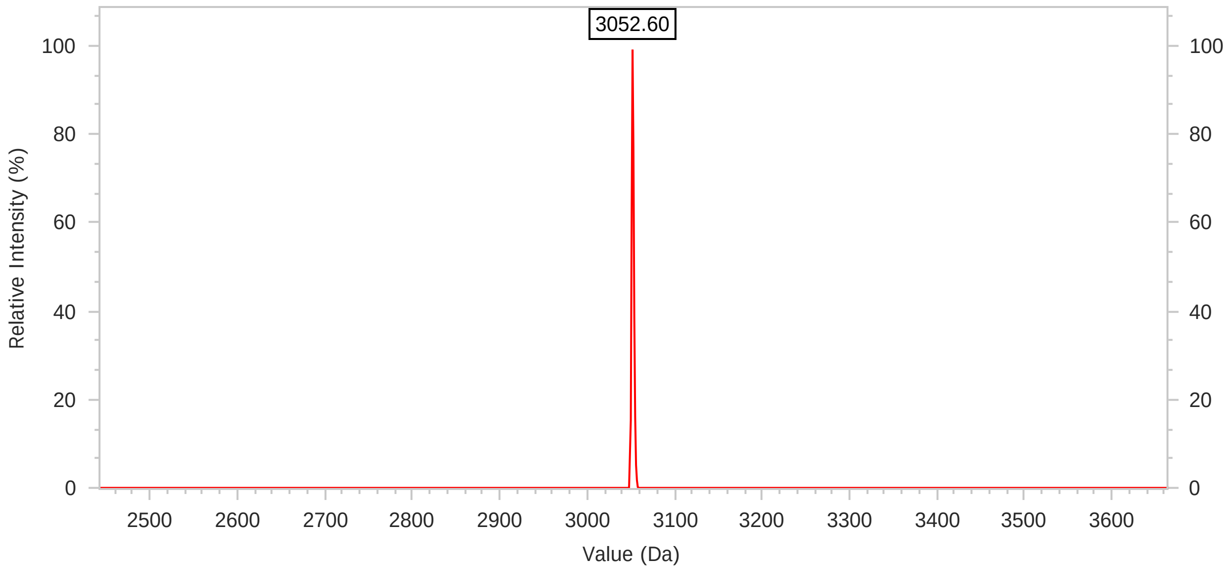

Sequence Name: AS006-GXC-mismatch
Sequence: 5'- CGA GAC TGC G -3'
Calculated Molecular Weight: 3053.0
Measured Molecular Weight: 3052.60

Integrated DNA Technologies

Analytical ESI-MS Report

Sales Order: 20121707
Reference ID: 473831386
Manufacturing ID: 72193006

Instrument:	SD-LTQ-06	Operator ID:	3574260
Acquired:	3/2/2024 6:53 PM	Reviewed:	3/2/2024 9:16 PM

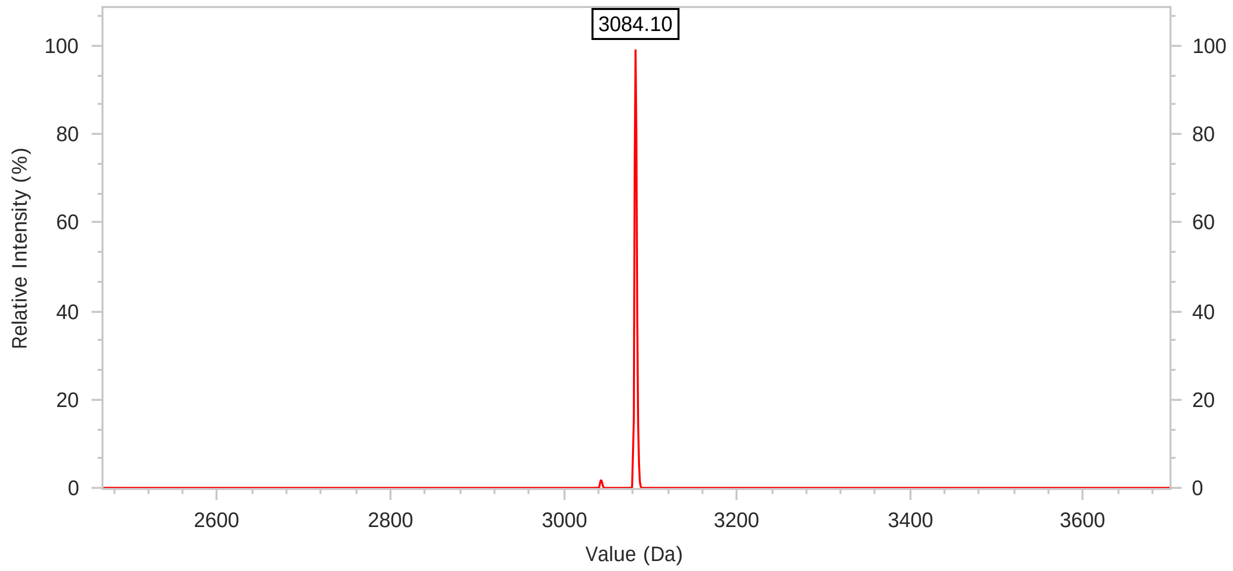

Sequence Name: AS005-comp
Sequence: 5'- CGA TGG TGC G -3'
Calculated Molecular Weight: 3084.0
Measured Molecular Weight: 3084.10

Integrated DNA Technologies

Analytical ESI-MS Report

Sales Order:	20121707
Reference ID:	473831387
Manufacturing ID:	72193007

Instrument:	SD-LTQ-06	Operator ID:	3574260
Acquired:	3/2/2024 6:53 PM	Reviewed:	3/2/2024 9:16 PM

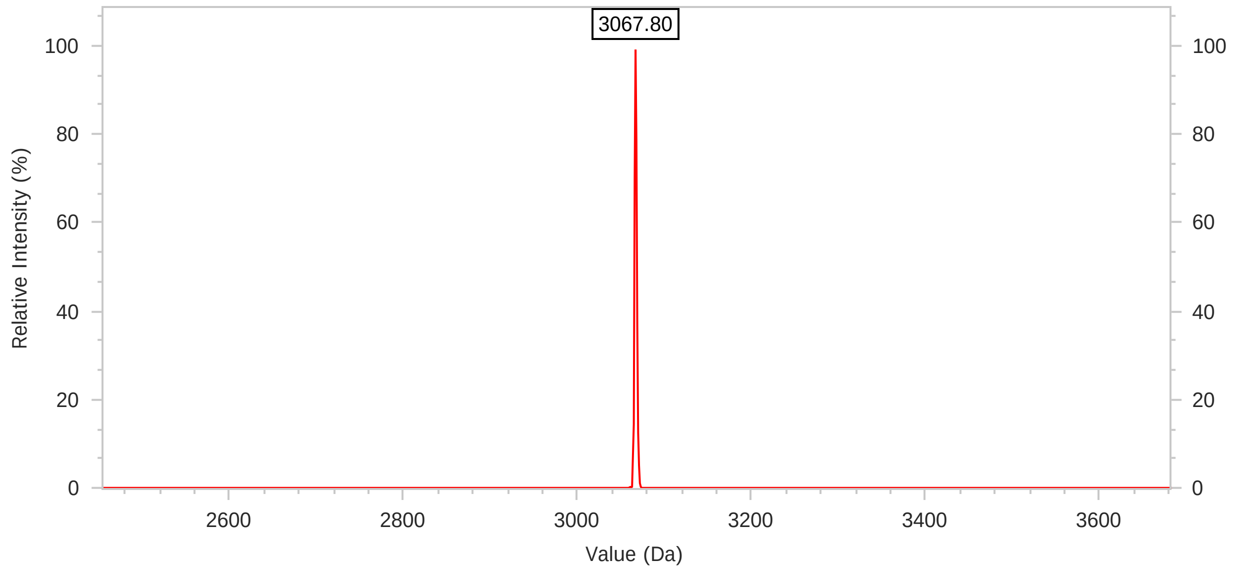

Sequence Name:	AS005-mismatch
Sequence:	5'- CGA TAG TGC G -3'
Calculated Molecular Weight:	3068.0
Measured Molecular Weight:	3067.80

Integrated DNA Technologies

Analytical ESI-MS Report

Sales Order: 20000529
Reference ID: 468502502
Manufacturing ID: 71520179

Instrument:	SD-LTQ-06	Operator ID:	3598860
Acquired:	1/16/2024 7:11 PM	Reviewed:	1/16/2024 9:14 PM

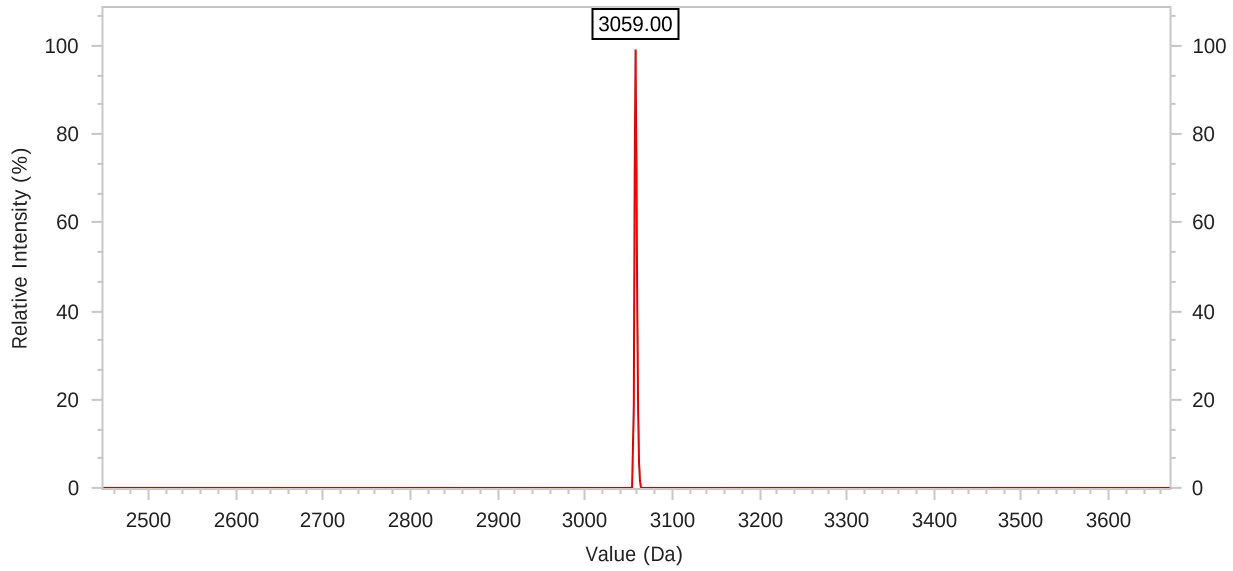

Sequence Name: AS004-AXA-comp
Sequence: 5'- CGA TGT TGC G -3'
Calculated Molecular Weight: 3059.0
Measured Molecular Weight: 3059.00

Integrated DNA Technologies

Analytical ESI-MS Report

Sales Order: 20000529
Reference ID: 468502503
Manufacturing ID: 71520178

Instrument:	SD-LTQ-06	Operator ID:	3598860
Acquired:	1/16/2024 7:10 PM	Reviewed:	1/16/2024 9:14 PM

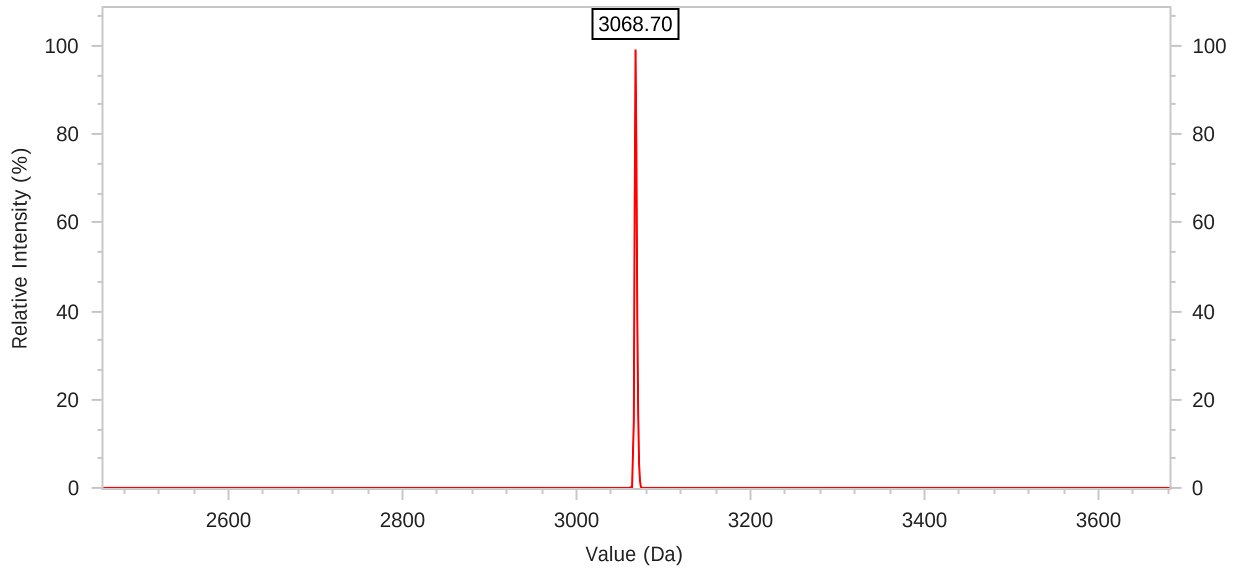

Sequence Name: AS006-GXC-comp
Sequence: 5'- CGA GGC TGC G -3'
Calculated Molecular Weight: 3069.0
Measured Molecular Weight: 3068.70

Integrated DNA Technologies

Analytical ESI-MS Report

Sales Order: 20000529
Reference ID: 468502504
Manufacturing ID: 622410618

Instrument: MS-IALTQ-11
Acquired: 1/25/2024 1:11 PM
Operator ID: 3581103
Reviewed: 1/25/2024 1:17 PM

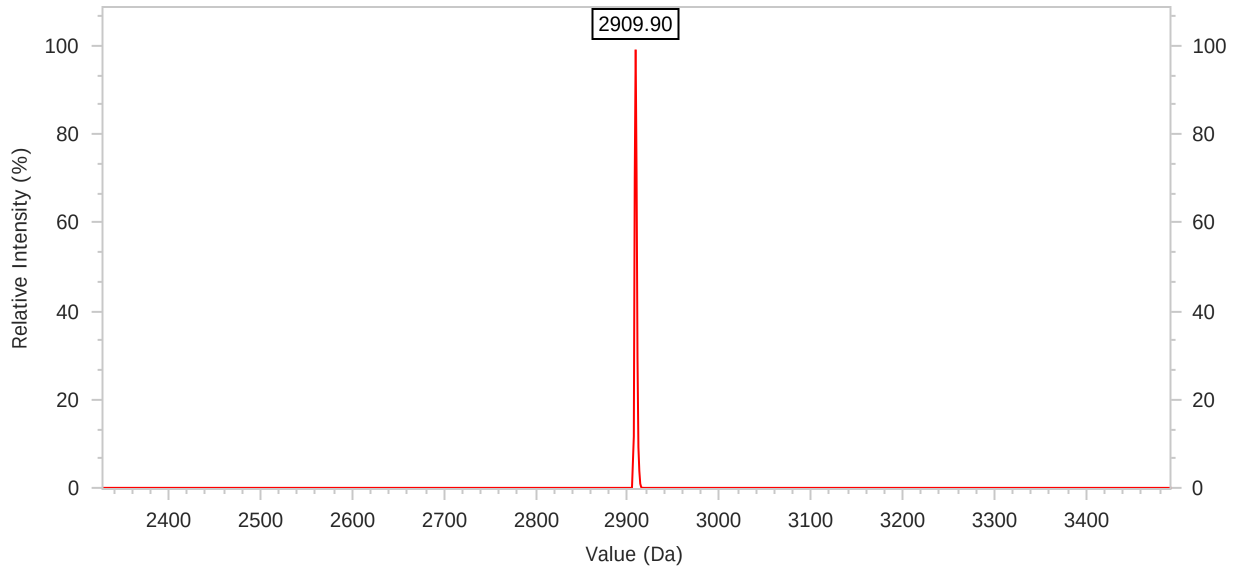

Sequence Name: AS004-AXA-dspacer-comp
Sequence: 5'- CGA T/idSp/TT GCG -3'
Calculated Molecular Weight: 2909.9
Measured Molecular Weight: 2909.90

Integrated DNA Technologies

Analytical ESI-MS Report

Sales Order: 20000529
Reference ID: 468502505
Manufacturing ID: 622499340

Instrument:	MS-IALTQ-02	Operator ID:	4024
Acquired:	1/26/2024 3:26 PM	Reviewed:	1/26/2024 4:53 PM

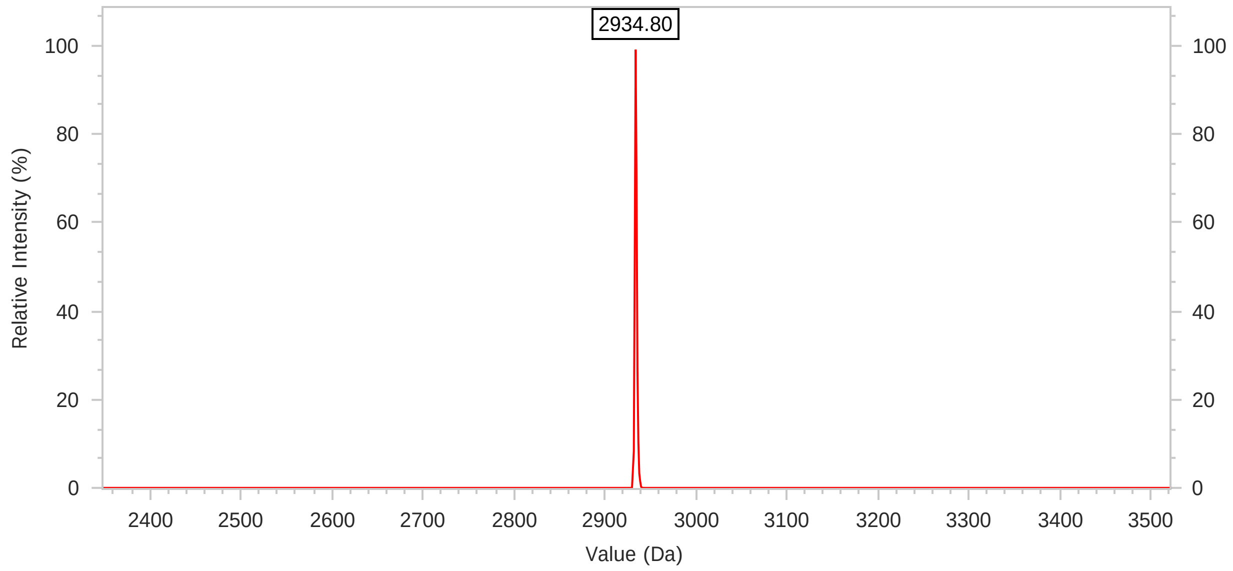

Sequence Name: AS005-CXA-dspacer-comp
Sequence: 5'- CGA T/idSp/GT GCG -3'
Calculated Molecular Weight: 2934.9
Measured Molecular Weight: 2934.80

Integrated DNA Technologies

Analytical ESI-MS Report

Sales Order: 20000529
Reference ID: 468502506
Manufacturing ID: 622410636

Instrument:	MS-IALTQ-11	Operator ID:	3581103
Acquired:	1/25/2024 1:03 PM	Reviewed:	1/25/2024 1:17 PM

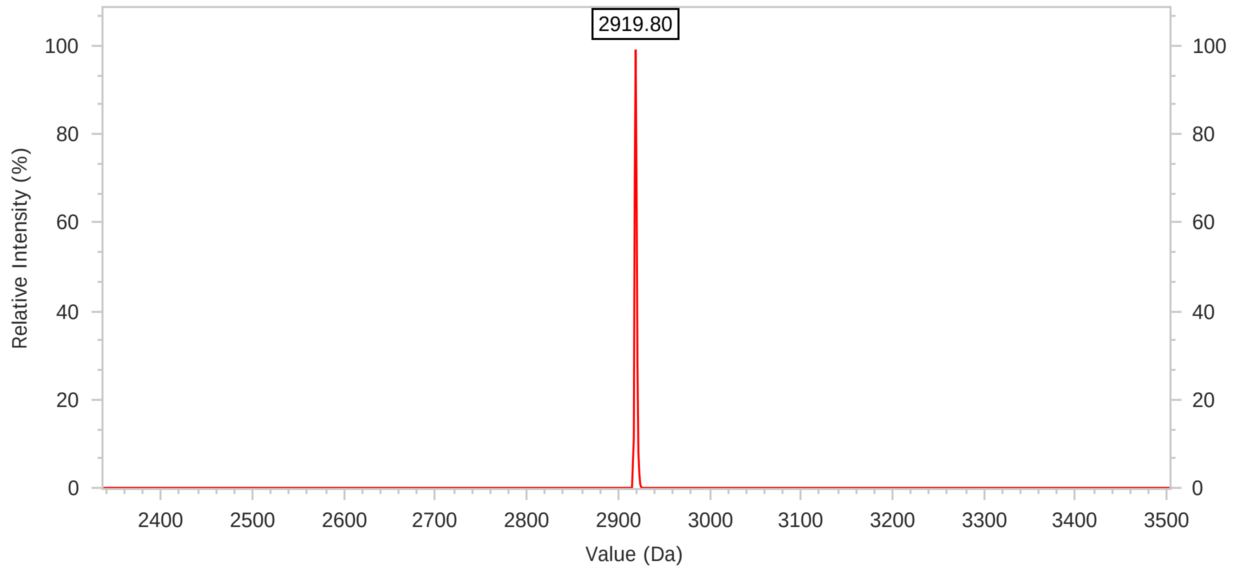

Sequence Name: AS006-GXC-dspacer-comp
Sequence: 5'- CGA G/idSp/CT GCG -3'
Calculated Molecular Weight: 2919.9
Measured Molecular Weight: 2919.80

Integrated DNA Technologies

Analytical ESI-MS Report

Sales Order: 20000529
Reference ID: 468502491
Manufacturing ID: 71559161

Instrument: SD-LTQ-05
Acquired: 1/18/2024 1:27 PM
Operator ID: 3598860
Reviewed: 1/18/2024 4:34 PM

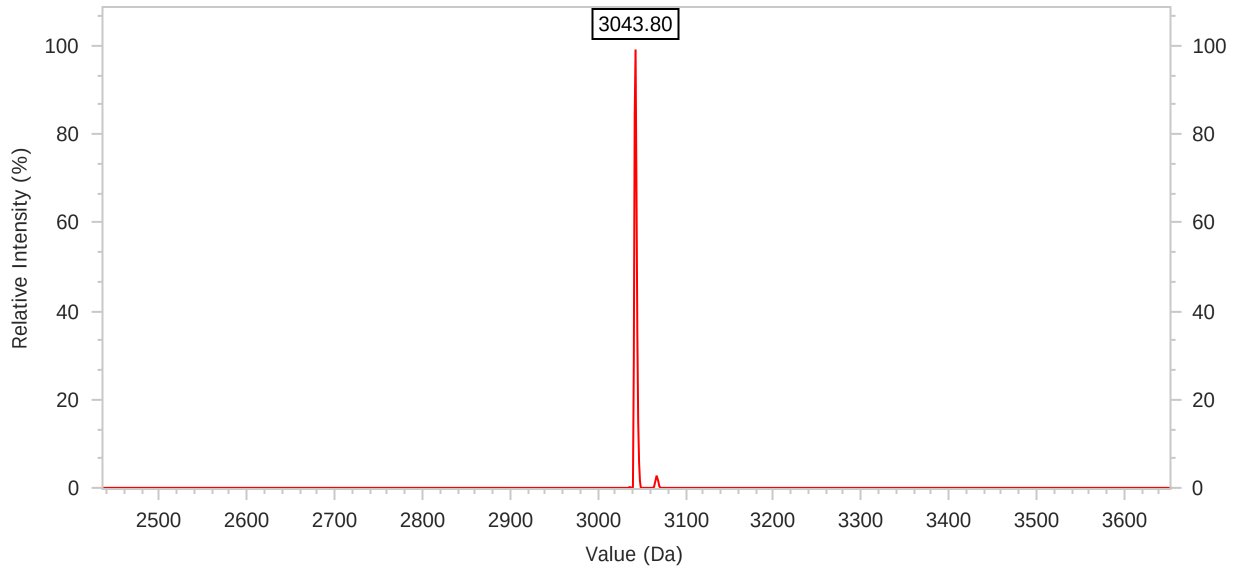

Sequence Name: AS004-AXA-inosine-comp
Sequence: 5'- CGA T/ideoxyI/T TGC G -3'
Calculated Molecular Weight: 3044.0
Measured Molecular Weight: 3043.80

Integrated DNA Technologies

Analytical OligoPro CE Report

Sample ID: 468502491-10 HCO A2 27847-O1
Instrument: Oligo Pro (Offline) Operator: HTA
Acquired: 1/18/2024 3:23:02 PM Reviewed: 1/18/2024 3:25:09 PM

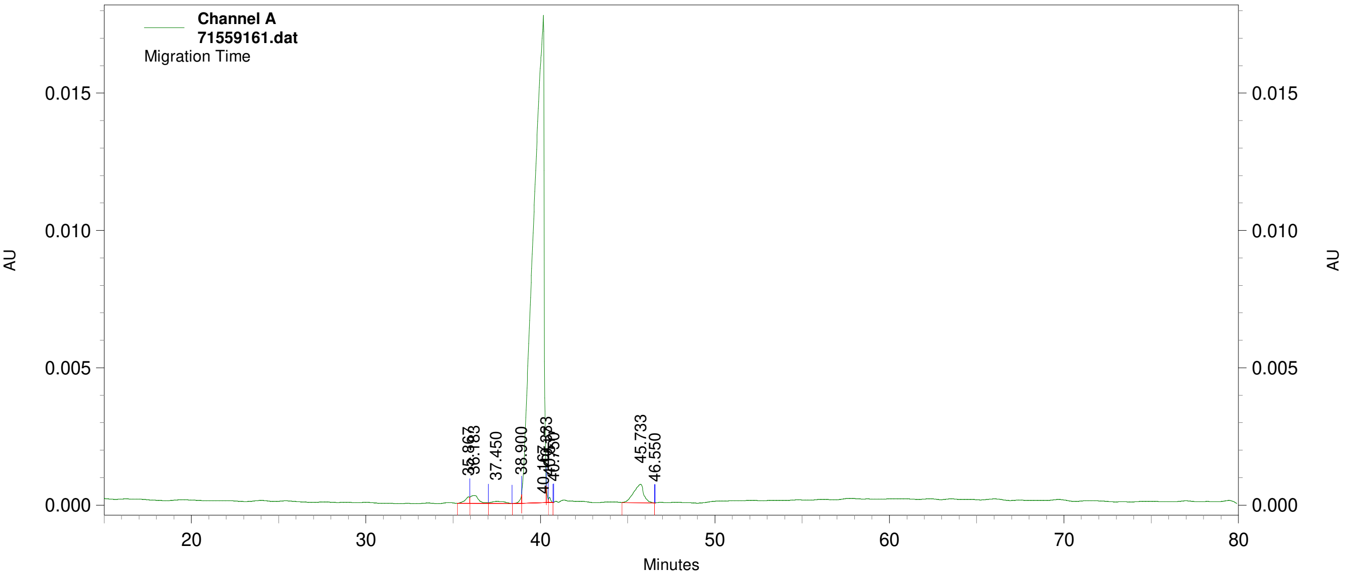

Channel A Results

Peak #	Time	Height	Corrected Area	Corrected Area Percent
1	35.87	234	110	0.6
2	36.18	284	237	1.3
3	37.45	73	98	0.5
4	38.90	259	42	0.2
5	40.17	17759	17090	93.9
6	40.33	591	53	0.3
7	40.52	188	34	0.2
8	40.75	6	0	0.0
9	45.73	673	543	3.0
10	46.55	0	0	0.0
Totals		20067		100.0

Integrated DNA Technologies

Analytical ESI-MS Report

Sales Order: 20000529
Reference ID: 468502492
Manufacturing ID: 71559156

Instrument: SD-LTQ-05
Acquired: 1/18/2024 1:27 PM
Operator ID: 3598860
Reviewed: 1/18/2024 4:34 PM

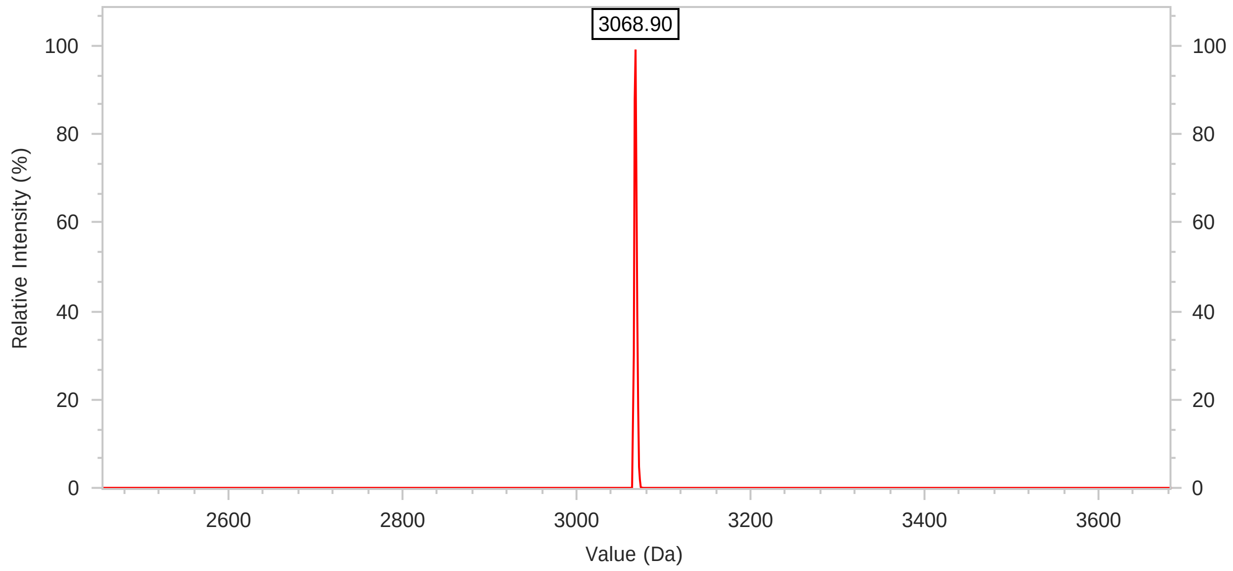

Sequence Name: AS005-CXA-inosine-comp
Sequence: 5'- CGA T/ideoxyI/G TGC G -3'
Calculated Molecular Weight: 3069.0
Measured Molecular Weight: 3068.90

Integrated DNA Technologies

Analytical OligoPro CE Report

Sample ID:	468502492-10 HCO A1 27847-O1		
Instrument:	Oligo Pro (Offline)	Operator:	HTA
Acquired:	1/18/2024 3:22:58 PM	Reviewed:	1/18/2024 3:24:45 PM

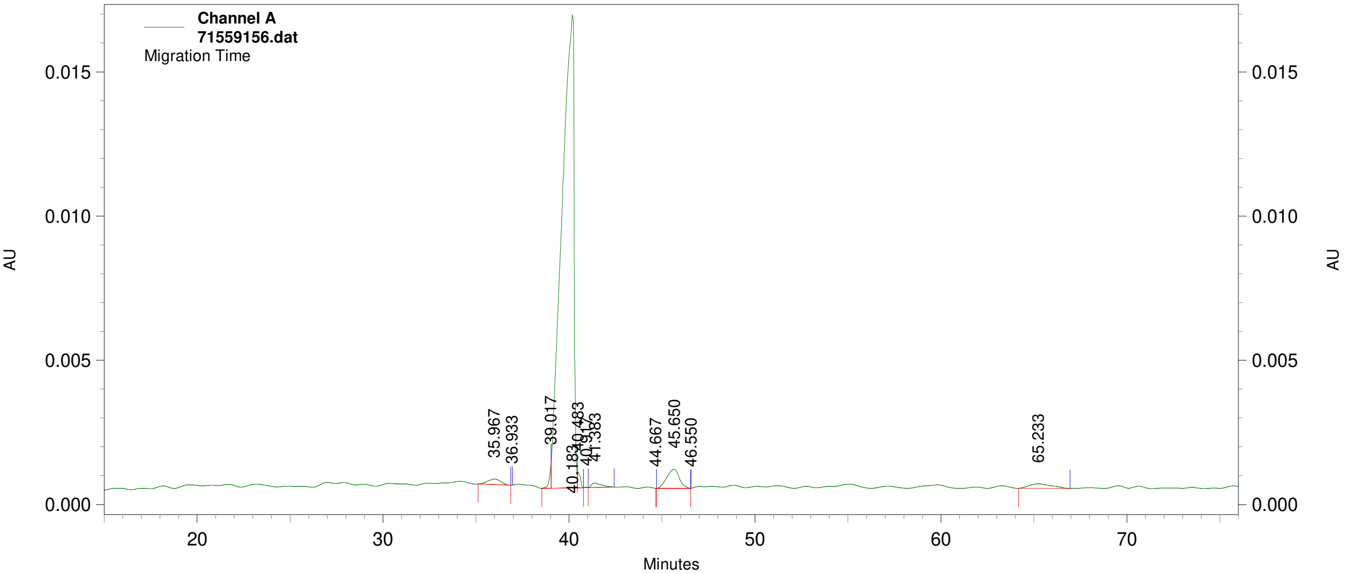

Channel A Results

Peak #	Time	Height	Corrected Area	Corrected Area Percent
1	35.97	187	229	1.2
2	36.93	1	0	0.0
3	39.02	770	113	0.6
4	40.18	16414	17125	92.4
5	40.48	549	126	0.7
6	40.92	26	5	0.0
7	41.38	160	135	0.7
8	44.67	0	0	0.0
9	45.65	654	611	3.3
10	46.55	1	0	0.0
11	65.23	156	185	1.0
Totals		18918		100.0

Integrated DNA Technologies

Analytical ESI-MS Report

Sales Order: 20000529
Reference ID: 468502493
Manufacturing ID: 71559167

Instrument: SD-LTQ-06
Acquired: 1/18/2024 7:39 PM
Operator ID: 3597521
Reviewed: 1/19/2024 12:58 AM

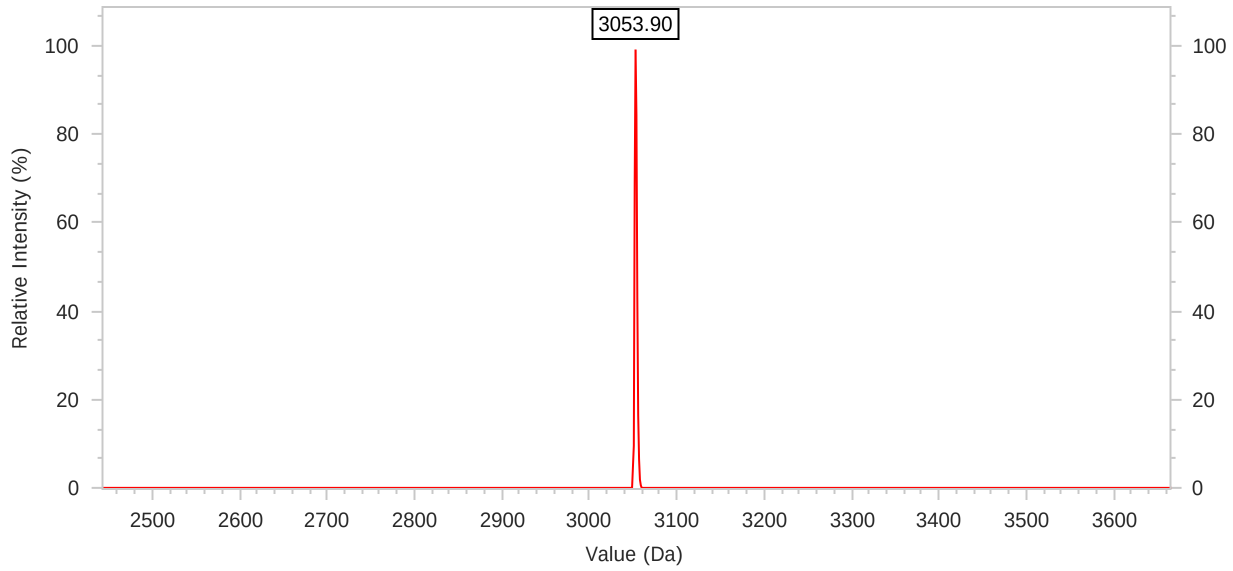

Sequence Name: AS006-GXC-inosine-comp
Sequence: 5'- CGA G/ideoxyI/C TGC G -3'
Calculated Molecular Weight: 3054.0
Measured Molecular Weight: 3053.90

Integrated DNA Technologies

Analytical RP HPLC Report

Sample ID: 468502493-10 MNP A2 27853-H2
Instrument: SD-HPLC-01 (Offline) Operator: HTA (HTA)
Acquired: 1/19/2024 4:22:03 AM (GMT -08:00) Reviewed: 1/19/2024 5:48:22 AM (GMT -08:00)

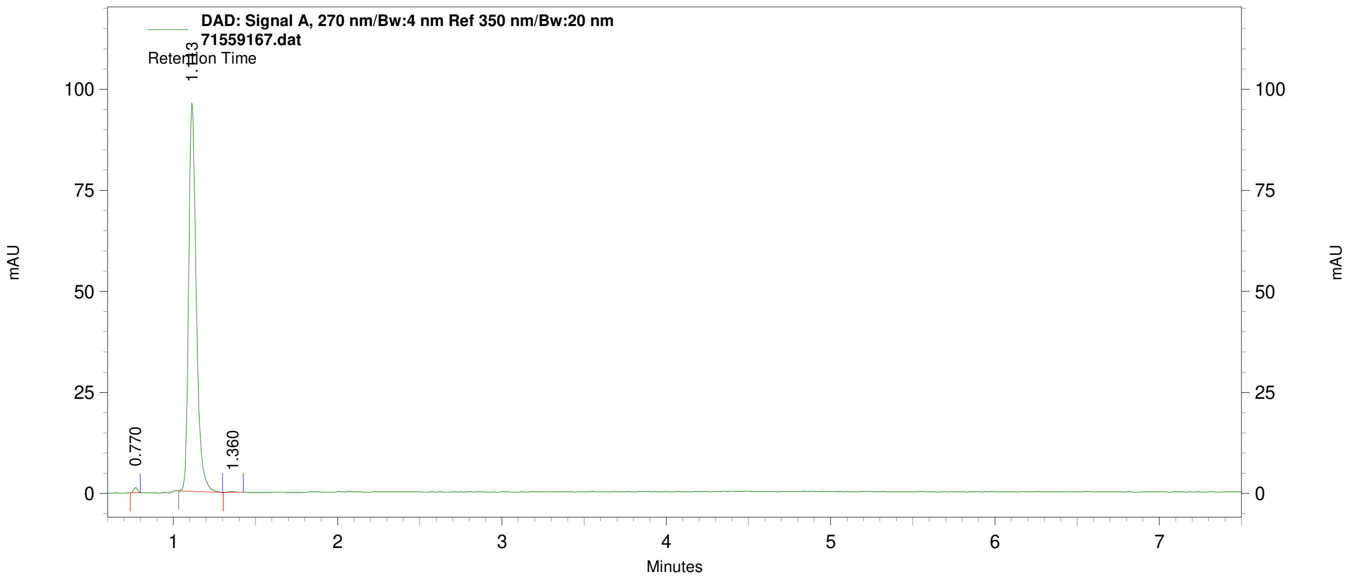

DAD: Signal A, 270 nm/Bw:4 nm Ref 350 nm/Bw:20 nm Results

Peak #	Time	Height	Area	Area Percent
1	0.77	2647	4460	0.7
2	1.11	201353	647410	99.2
3	1.36	340	1013	0.2

Totals				
		204340	652883	100.0

Integrated DNA Technologies

Analytical ESI-MS Report

Sales Order: 20000529
Reference ID: 468502494
Manufacturing ID: 622206426

Instrument:	MS-IALTQ-10	Operator ID:	2338
Acquired:	1/24/2024 5:44 AM	Reviewed:	1/24/2024 6:23 AM

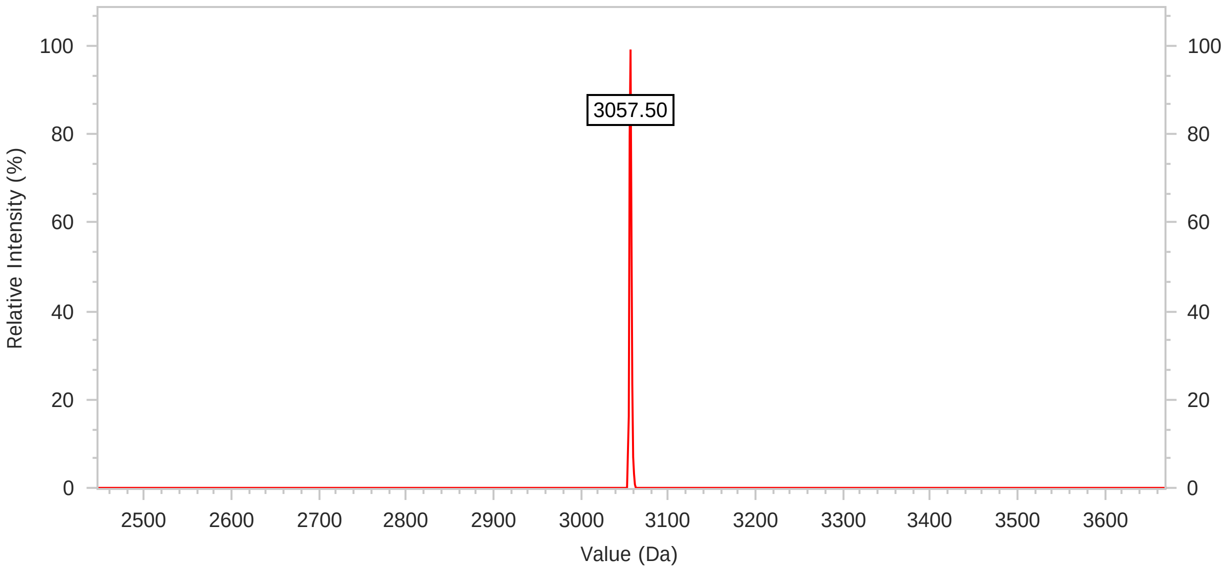

Sequence Name: AS004-AXA-diaminopurine-comp
Sequence: 5'- CGA T/i6diPr/T TGC G -3'
Calculated Molecular Weight: 3058.0
Measured Molecular Weight: 3057.50

Integrated DNA Technologies

Analytical OligoPro CE Report

Sample ID: 468502494-10 HCO E7 219729-O5
Instrument: Oligo Pro (Offline) Operator: HTA
Acquired: 1/24/2024 7:12:00 AM Reviewed: 1/24/2024 9:19:35 AM

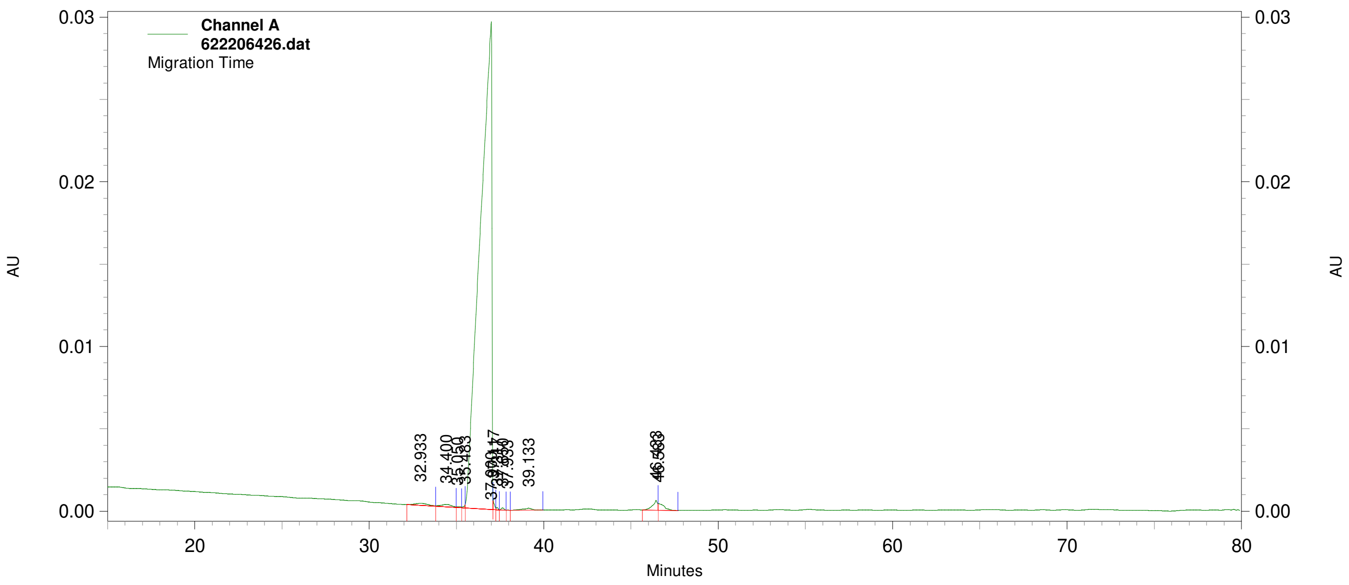

Channel A Results

Peak #	Time	Height	Corrected Area	Corrected Area Percent
1	32.93	119	178	0.5
2	34.40	140	161	0.5
3	35.05	47	23	0.1
4	35.48	147	25	0.1
5	37.00	29635	33960	97.0
6	37.12	613	70	0.2
7	37.32	141	26	0.1
8	37.65	131	34	0.1
9	37.93	5	0	0.0
10	39.13	125	110	0.3
11	46.43	584	218	0.6
12	46.58	394	191	0.5
Totals		32081		100.0

Integrated DNA Technologies

Analytical ESI-MS Report

Sales Order: 20000529
Reference ID: 468502495
Manufacturing ID: 622241306

Instrument:	MS-IALTQ-01	Operator ID:	2338
Acquired:	1/25/2024 3:35 AM	Reviewed:	1/25/2024 4:47 AM

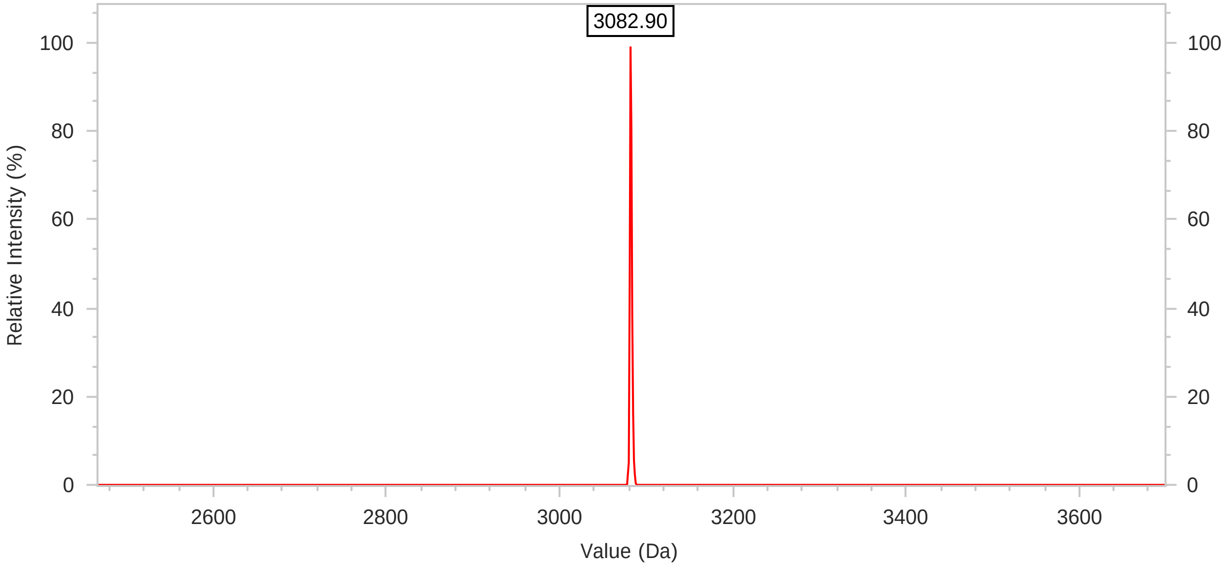

Sequence Name: AS005-CXA-diaminopurine-comp
Sequence: 5'- CGA T/i6diPr/G TGC G -3'
Calculated Molecular Weight: 3083.1
Measured Molecular Weight: 3082.90

Integrated DNA Technologies

Analytical OligoPro CE Report

Sample ID: 468502495-10 HCO B12 219775-O
Instrument: Oligo Pro (Offline) Operator: HTA
Acquired: 1/25/2024 3:26:00 AM Reviewed: 1/25/2024 6:42:24 AM

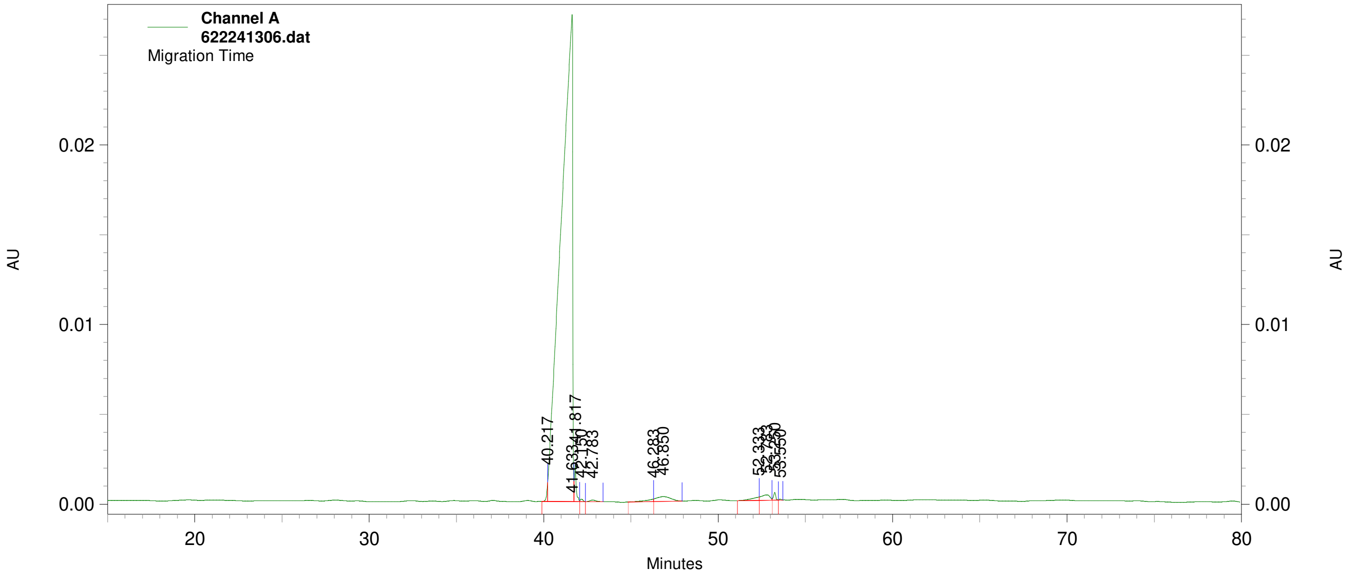

Channel A Results

Peak #	Time	Height	Corrected Area	Corrected Area Percent
1	40.22	853	91	0.3
2	41.63	27128	27781	95.7
3	41.82	2056	339	1.2
4	42.15	136	32	0.1
5	42.78	91	43	0.1
6	46.28	142	90	0.3
7	46.85	267	303	1.0
8	52.33	187	98	0.3
9	52.78	302	178	0.6
10	53.25	429	63	0.2
11	53.55	50	7	0.0

Totals				
		31641		100.0

Integrated DNA Technologies

Analytical ESI-MS Report

Sales Order: 20000529
Reference ID: 468502496
Manufacturing ID: 622241305

Instrument:	MS-IALTQ-01	Operator ID:	2338
Acquired:	1/25/2024 3:32 AM	Reviewed:	1/25/2024 4:47 AM

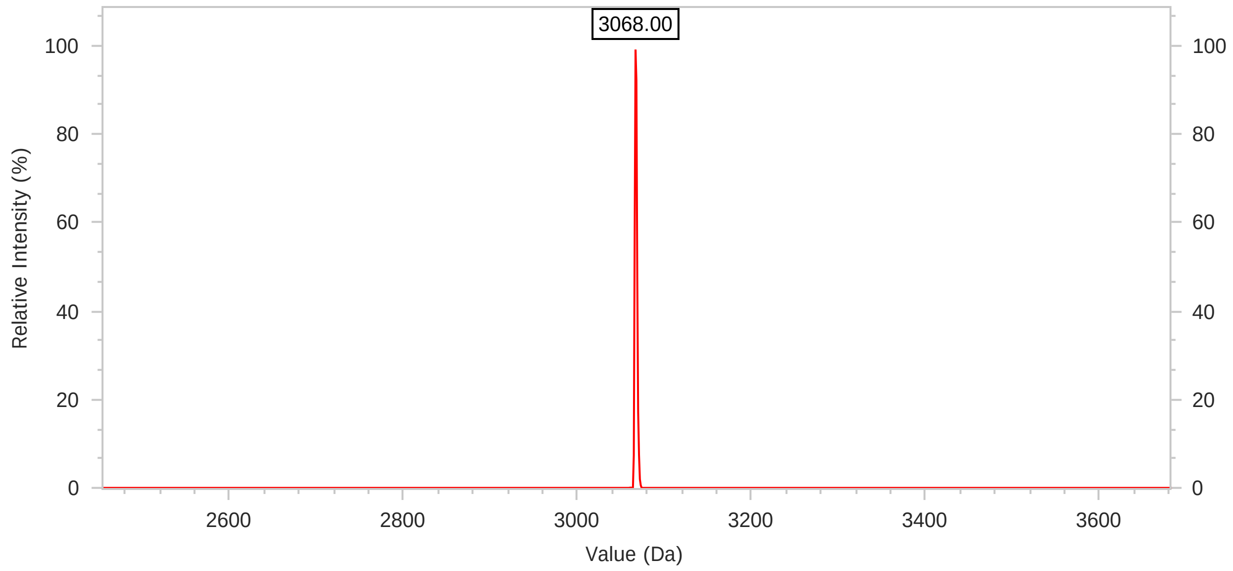

Sequence Name: AS006-GXC-diaminopurine-comp
Sequence: 5'- CGA G/i6diPr/C TGC G -3'
Calculated Molecular Weight: 3068.0
Measured Molecular Weight: 3068.00

Integrated DNA Technologies

Analytical OligoPro CE Report

Sample ID:	468502496-10 HCO B8 219775-O4		
Instrument:	Oligo Pro (Offline)	Operator:	HTA
Acquired:	1/25/2024 3:26:00 AM	Reviewed:	1/25/2024 6:40:39 AM

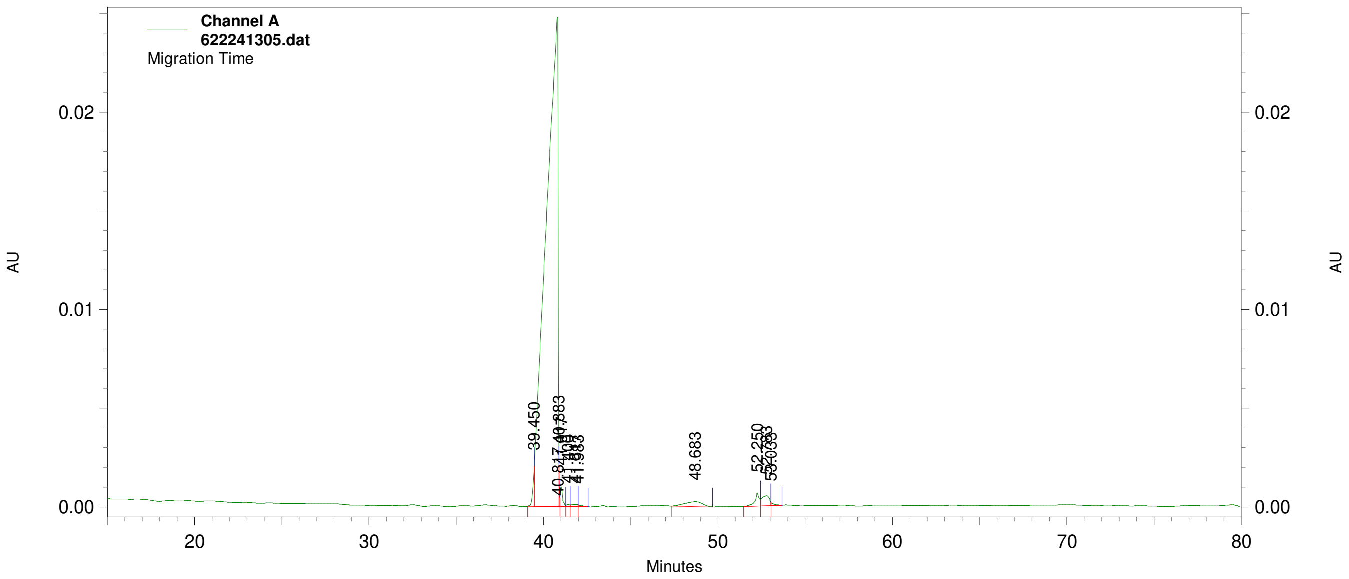

Channel A Results

Peak #	Time	Height	Corrected Area	Corrected Area Percent
1	39.45	1768	231	0.8
2	40.82	24790	26325	94.8
3	40.88	2091	77	0.3
4	41.02	1003	190	0.7
5	41.40	100	31	0.1
6	41.82	116	62	0.2
7	41.98	95	30	0.1
8	48.68	246	322	1.2
9	52.25	653	204	0.7
10	52.78	507	257	0.9
11	53.03	176	33	0.1
Totals		31545		100.0

Integrated DNA Technologies

Analytical ESI-MS Report

Sales Order: 20000529
Reference ID: 468502497
Manufacturing ID: 620782120

Instrument: MS-IALTQ-10
Acquired: 1/20/2024 4:08 PM
Operator ID: 3114
Reviewed: 1/20/2024 4:19 PM

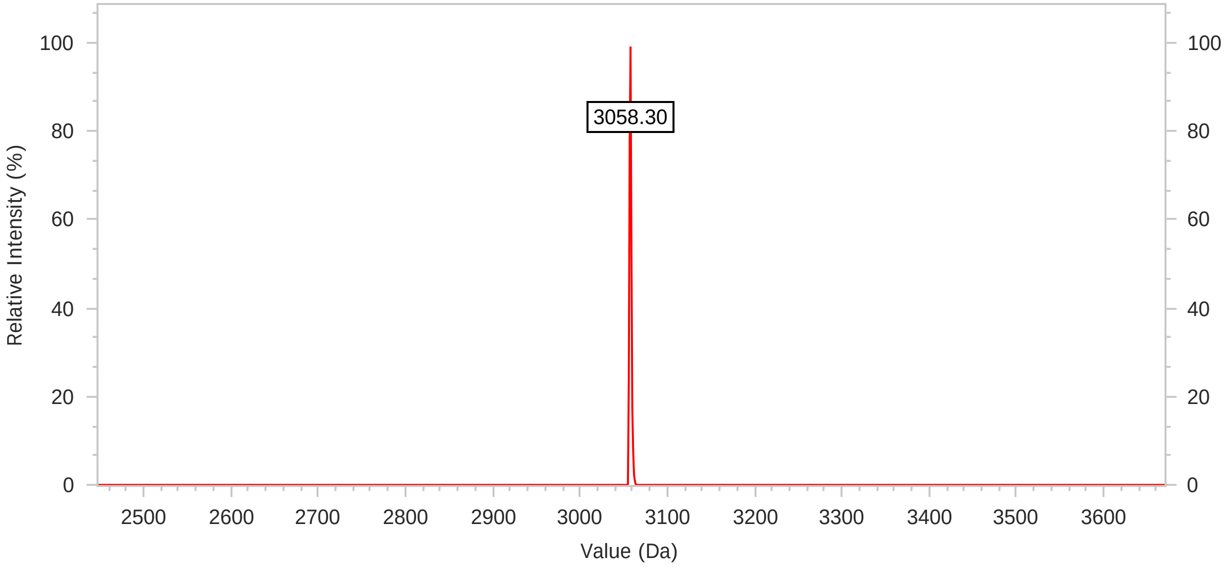

Sequence Name: AS004-AXA-isodG-comp
Sequence: 5'- CGA T/iisodG/T TGC G -3'
Calculated Molecular Weight: 3059.0
Measured Molecular Weight: 3058.30

Integrated DNA Technologies

Analytical OligoPro CE Report

Sample ID: 468502497-10 HCO C5 219577-O6
Instrument: Oligo Pro (Offline) Operator: HTA
Acquired: 1/20/2024 4:05:00 PM Reviewed: 1/20/2024 6:08:38 PM

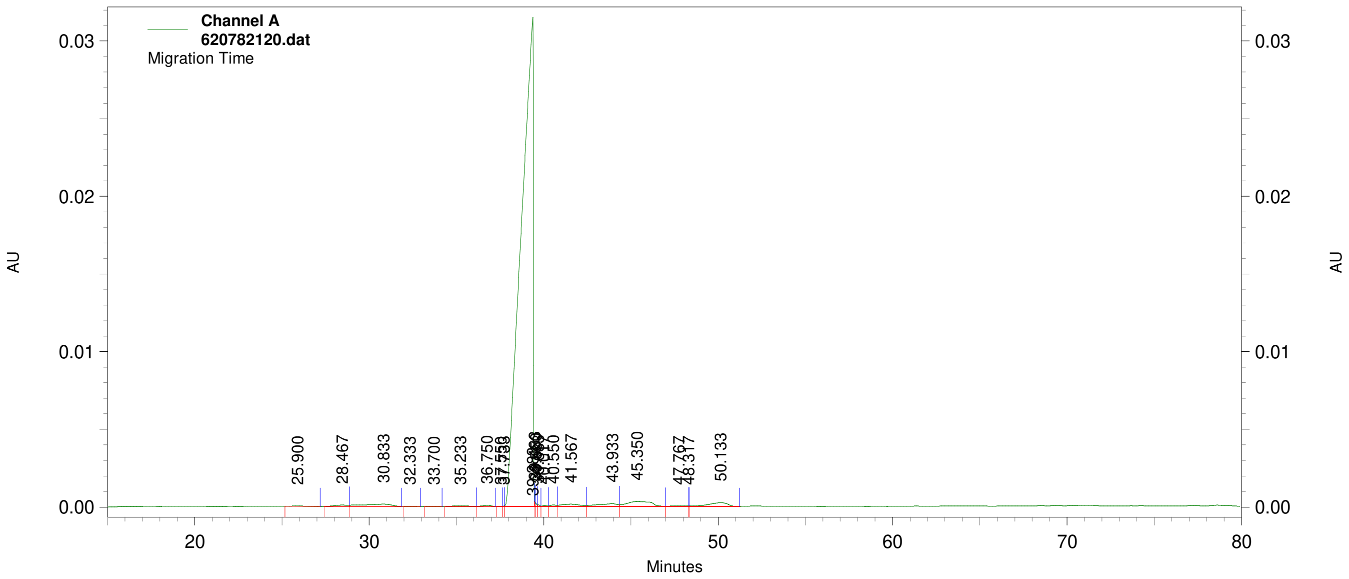

Channel A Results

Peak #	Time	Height	Corrected Area	Corrected Area Percent
1	25.90	27	58	0.2
2	28.47	87	157	0.4
3	30.83	152	531	1.4
4	32.33	7	7	0.0
5	33.70	13	12	0.0
6	35.23	37	64	0.2
7	36.75	76	63	0.2
8	37.55	13	3	0.0
9	37.73	31	4	0.0
10	39.38	31512	34928	93.1
11	39.48	331	17	0.0
12	39.55	238	26	0.1
13	39.68	113	21	0.1
14	40.02	55	23	0.1
15	40.55	92	54	0.1
16	41.57	151	239	0.6
17	43.93	196	295	0.8
18	45.35	320	639	1.7
19	47.77	35	43	0.1
20	48.32	30	1	0.0
21	50.13	243	346	0.9

Totals		33759		100.0

Integrated DNA Technologies

Analytical ESI-MS Report

Sales Order: 20000529
Reference ID: 468502498
Manufacturing ID: 620782115

Instrument: MS-IALTQ-07
Acquired: 1/20/2024 3:45 PM
Operator ID: 3114
Reviewed: 1/20/2024 4:19 PM

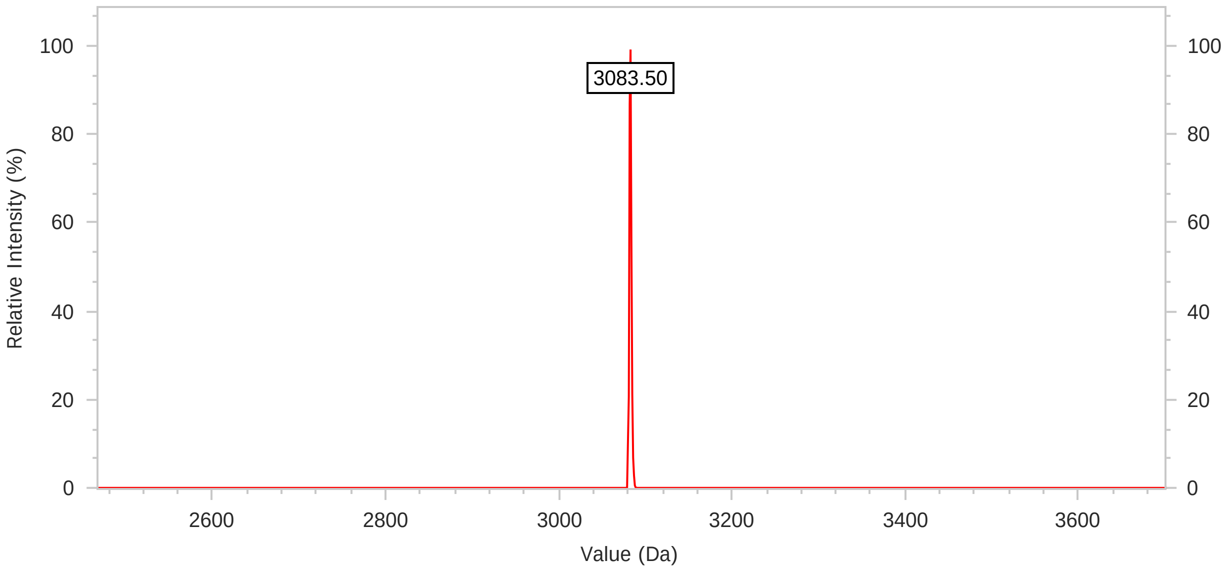

Sequence Name: AS005-CXA-isodG-comp
Sequence: 5'- CGA T/iisodG/G TGC G -3'
Calculated Molecular Weight: 3084.0
Measured Molecular Weight: 3083.50

Integrated DNA Technologies

Analytical OligoPro CE Report

Sample ID: 468502498-10 HCO B3 219577-O6
Instrument: Oligo Pro (Offline) Operator: HTA
Acquired: 1/20/2024 4:05:00 PM Reviewed: 1/20/2024 6:00:45 PM

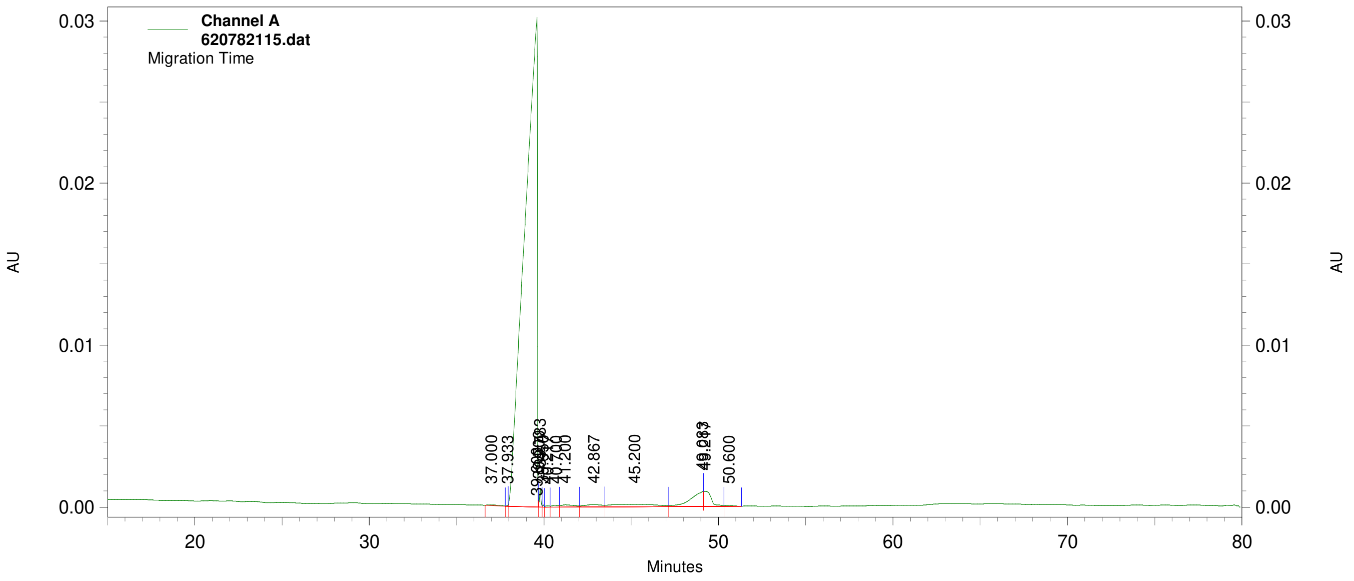

Channel A Results

Peak #	Time	Height	Corrected Area	Corrected Area Percent
1	37.00	36	46	0.1
2	37.93	58	6	0.0
3	39.60	30230	34728	93.2
4	39.70	412	17	0.0
5	39.78	1140	149	0.4
6	39.95	130	14	0.0
7	40.22	77	26	0.1
8	40.70	100	59	0.2
9	41.20	130	153	0.4
10	42.87	136	201	0.5
11	45.20	142	519	1.4
12	49.08	907	818	2.2
13	49.22	916	516	1.4
14	50.60	52	27	0.1
Totals		34466		100.0

Integrated DNA Technologies

Analytical ESI-MS Report

Sales Order: 20000529
Reference ID: 468502499
Manufacturing ID: 620782116

Instrument:	MS-IALTQ-07	Operator ID:	3114
Acquired:	1/20/2024 3:48 PM	Reviewed:	1/20/2024 4:19 PM

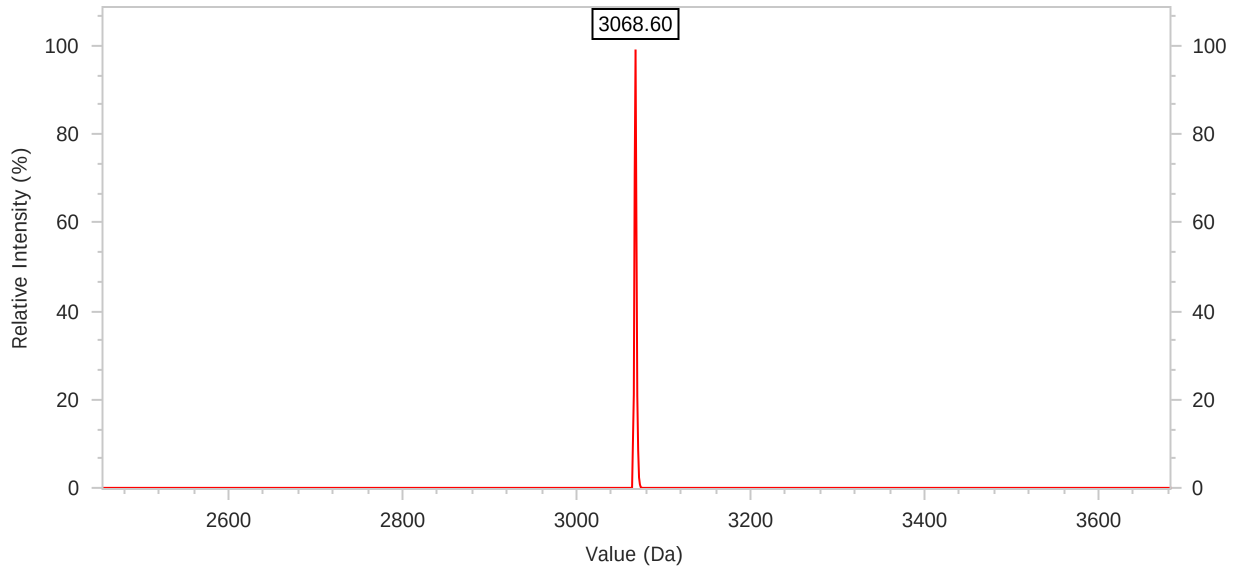

Sequence Name: AS006-GXC-isodG-comp
Sequence: 5'- CGA G/iisodG/C TGC G -3'
Calculated Molecular Weight: 3069.0
Measured Molecular Weight: 3068.60

Integrated DNA Technologies

Analytical OligoPro CE Report

Sample ID:	468502499-10 HCO B10 219577-O		
Instrument:	Oligo Pro (Offline)	Operator:	HTA
Acquired:	1/20/2024 4:05:00 PM	Reviewed:	1/20/2024 6:07:16 PM

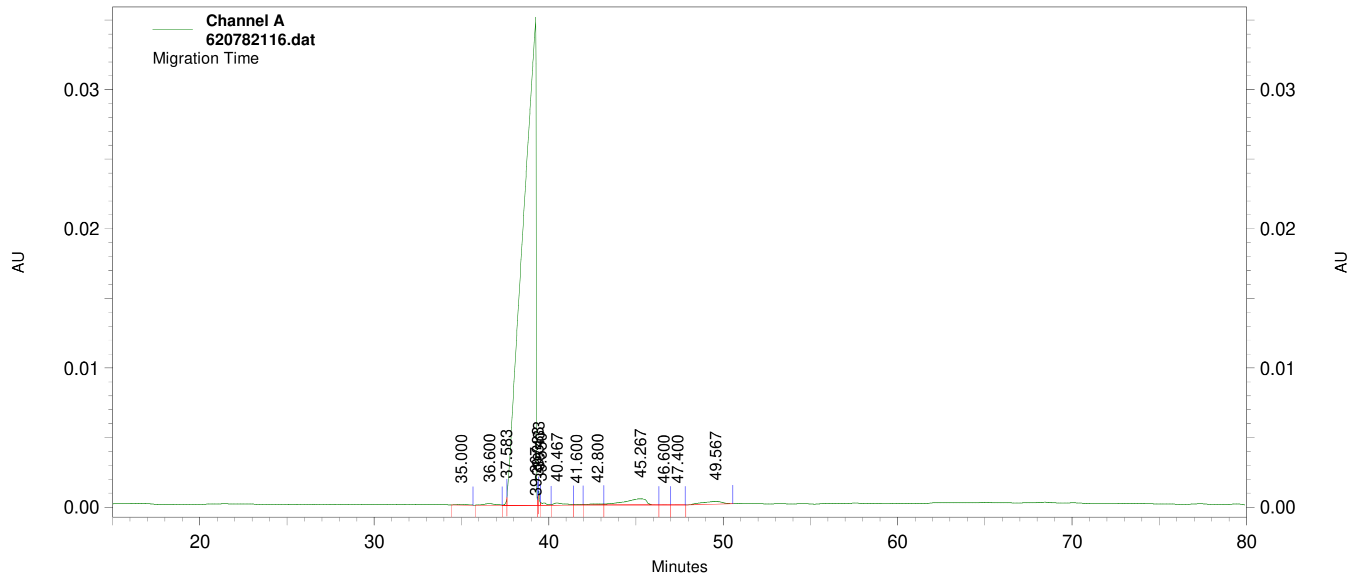

Channel A Results

Peak #	Time	Height	Corrected Area	Corrected Area Percent
1	35.00	55	55	0.1
2	36.60	106	117	0.3
3	37.58	400	42	0.1
4	39.27	35088	43041	95.8
5	39.38	592	39	0.1
6	39.43	998	102	0.2
7	39.55	175	79	0.2
8	40.47	152	156	0.3
9	41.60	56	39	0.1
10	42.80	85	113	0.3
11	45.27	449	822	1.8
12	46.60	22	12	0.0
13	47.40	17	11	0.0
14	49.57	194	299	0.7
Totals		38389		100.0

Integrated DNA Technologies

Analytical ESI-MS Report

Sales Order: 20314295
Reference ID: 481895981
Manufacturing ID: 644422904

Instrument:	MS-IALTQ-01	Operator ID:	3624262
Acquired:	5/27/2024 4:23 PM	Reviewed:	5/27/2024 6:44 PM

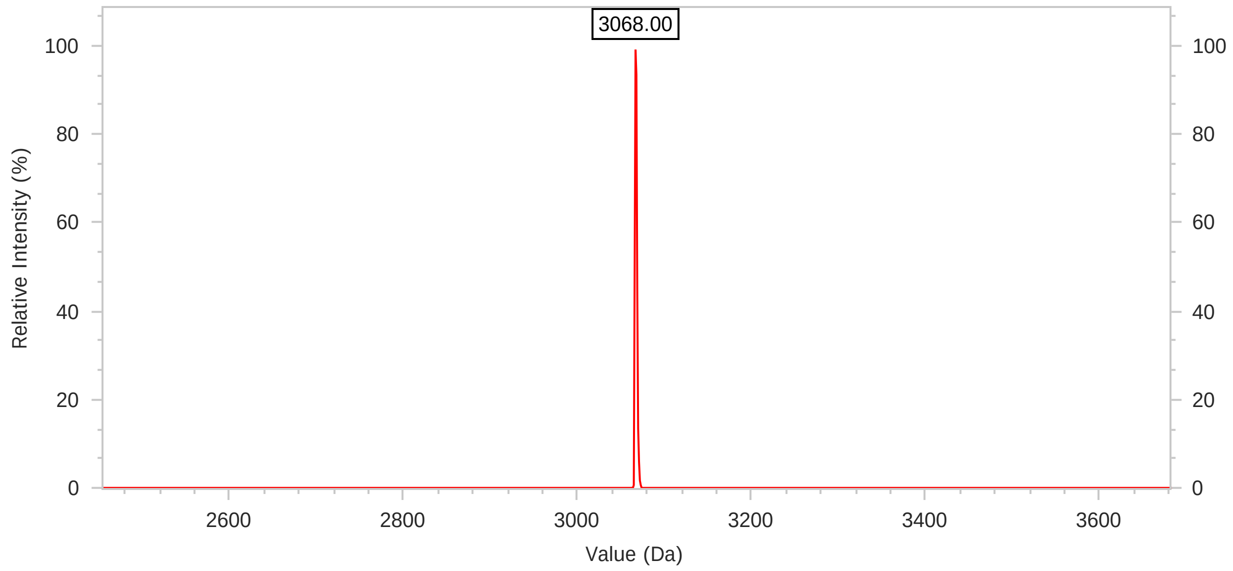

Sequence Name: AS006-GXC-diaminoopurine
Sequence: 5'- CGA G/i6diPr/C TGC G -3'
Calculated Molecular Weight: 3068.0
Measured Molecular Weight: 3068.00

Integrated DNA Technologies

Analytical OligoPro CE Report

Sample ID: 481895981-10 HPL D2 224747-O5
Instrument: Oligo Pro (Offline) Operator: HTA
Acquired: 5/27/2024 1:42:00 PM Reviewed: 5/27/2024 11:43:00 PM

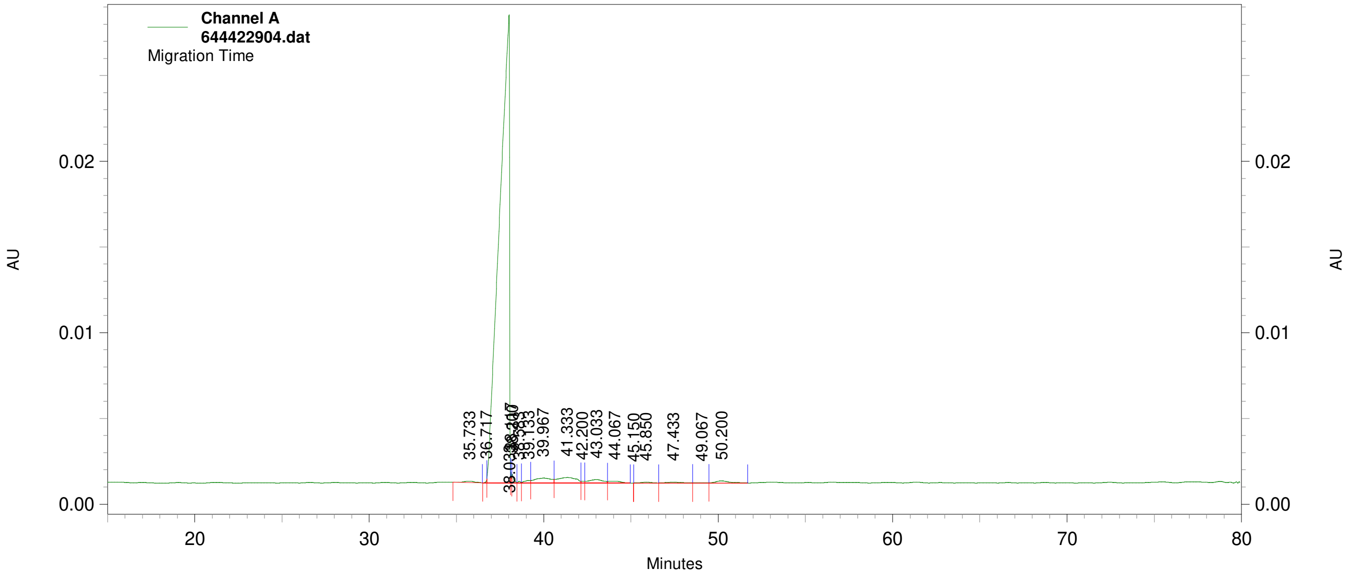

Channel A Results

Peak #	Time	Height	Corrected Area	Corrected Area Percent
1	35.73	89	94	0.3
2	36.72	155	16	0.1
3	38.03	27325	26406	93.3
4	38.12	625	37	0.1
5	38.20	515	87	0.3
6	38.58	91	22	0.1
7	39.13	142	81	0.3
8	39.97	281	427	1.5
9	41.33	316	517	1.8
10	42.20	104	28	0.1
11	43.03	199	239	0.8
12	44.07	111	111	0.4
13	45.15	0	0	0.0
14	45.85	47	39	0.1
15	47.43	43	55	0.2
16	49.07	20	14	0.1
17	50.20	131	125	0.4
Totals		30194		100.0

Integrated DNA Technologies

Analytical ESI-MS Report

Sales Order: 20314295
Reference ID: 481895980
Manufacturing ID: 646503707

Instrument: MS-IALTQ-12
Acquired: 6/7/2024 10:04 AM
Operator ID: 3587739
Reviewed: 6/7/2024 12:18 PM

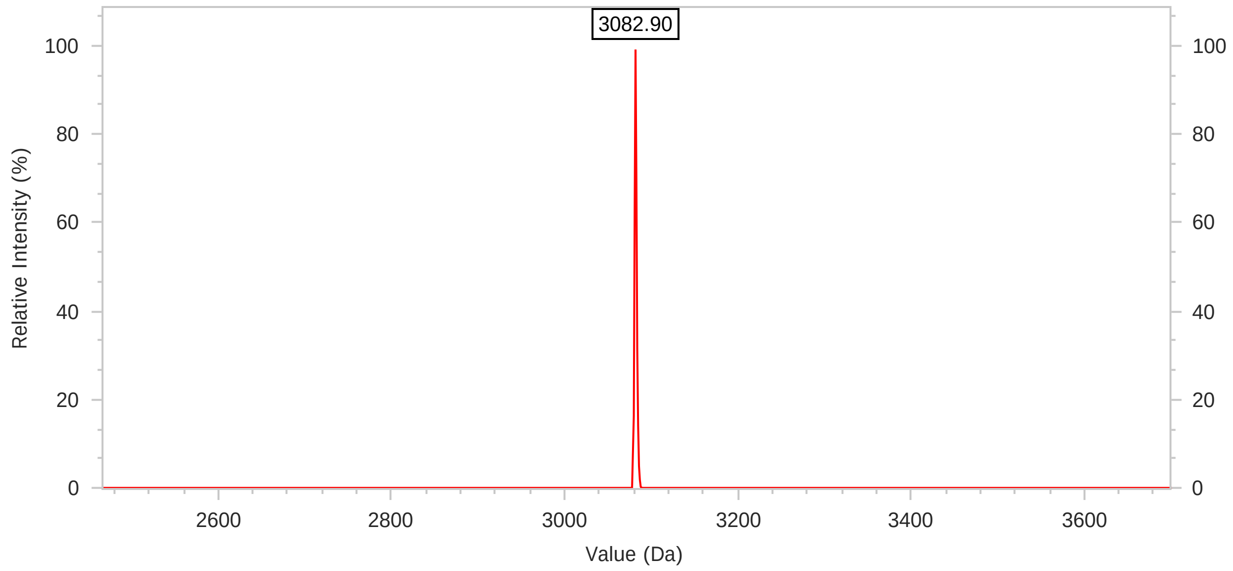

Sequence Name: AS005-CXA-diaminoopurine
Sequence: 5'- CGA T/i6diPr/G TGC G -3'
Calculated Molecular Weight: 3083.1
Measured Molecular Weight: 3082.90

Integrated DNA Technologies

Analytical OligoPro CE Report

Sample ID: 481895980-10 HPL A8 225190-O2
Instrument: Oligo Pro (Offline) Operator: HTA
Acquired: 6/7/2024 4:32:00 PM Reviewed: 6/7/2024 6:27:54 PM

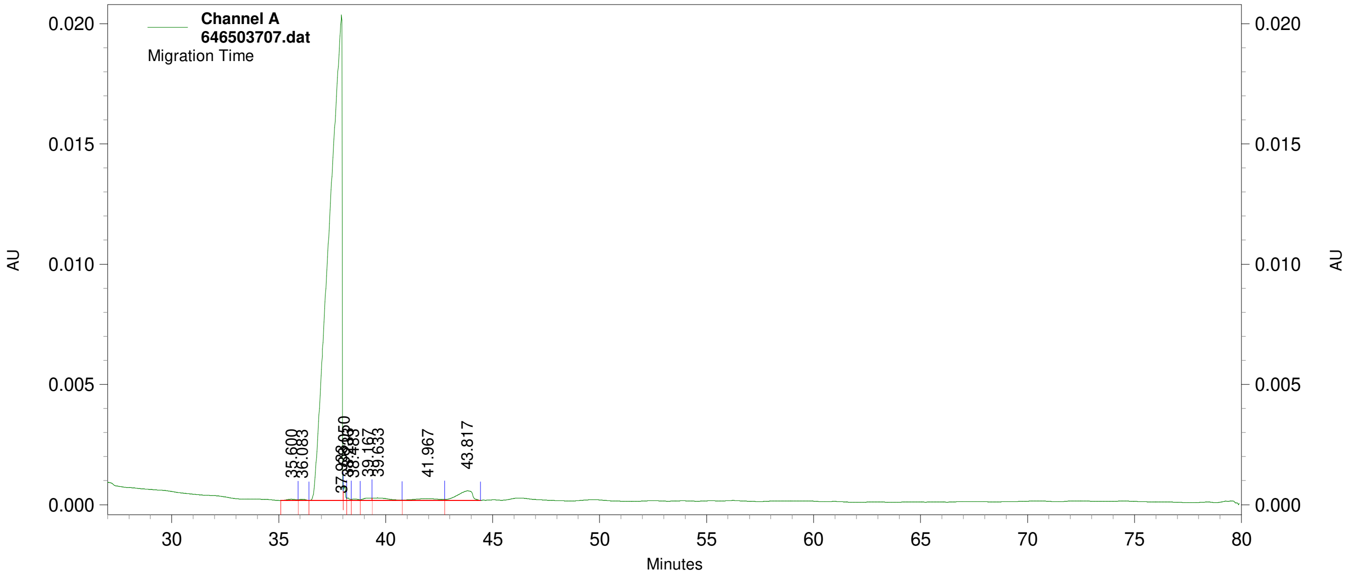

Channel A Results

Peak #	Time	Height	Corrected Area	Corrected Area Percent
1	35.60	54	41	0.2
2	36.08	49	28	0.1
3	37.93	20200	20159	95.6
4	38.05	598	86	0.4
5	38.23	106	21	0.1
6	38.48	58	29	0.1
7	39.17	101	63	0.3
8	39.63	105	122	0.6
9	41.97	68	126	0.6
10	43.82	397	400	1.9

Totals		21736		100.0

Integrated DNA Technologies

Analytical ESI-MS Report

Sales Order:	22956325
Reference ID:	588867694
Manufacturing ID:	732552441

Instrument:	HQC-LTQ-11	Operator ID:	3663144
Acquired:	8/18/2025 10:02 AM	Reviewed:	8/18/2025 1:18 PM

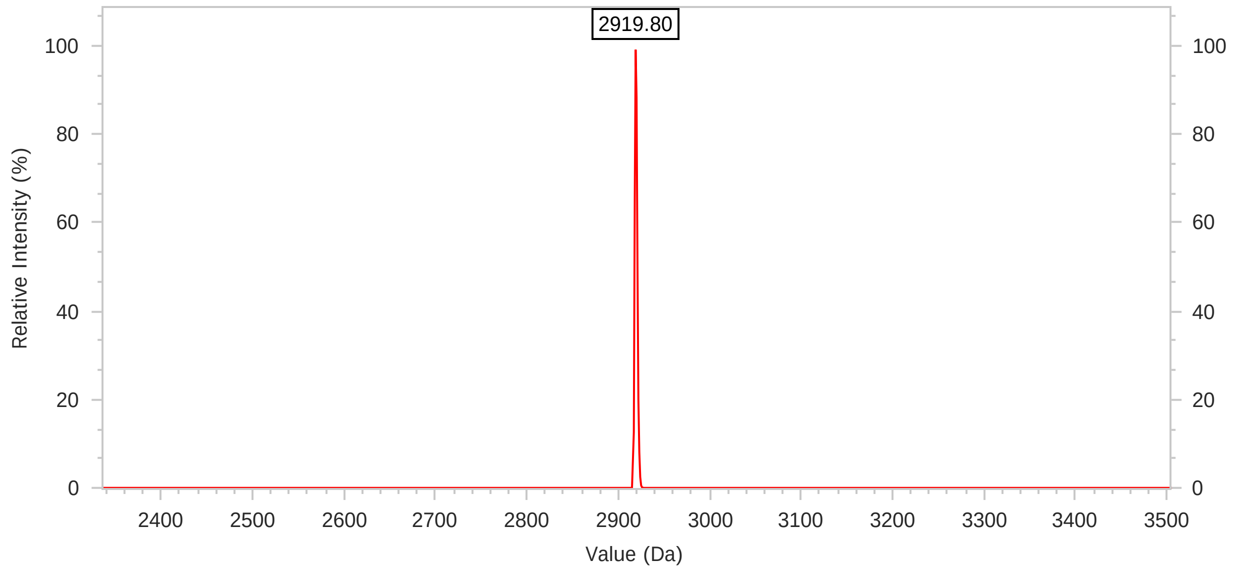

Sequence Name:	AS006 GXC dspacer
Sequence:	5'- CGA G/idSp/CT GCG -3'
Calculated Molecular Weight:	2919.9
Measured Molecular Weight:	2919.80

The following oligonucleotides were synthesized by the Keck Oligonucleotide Synthesis Facility at Yale University (New Haven, CT) and analyzed by LC-UV-ESI-TOFMS instrument by UCSD Molecular Mass Spectrometry Facility at University of California San Diego (San Diego, CA). The sequences in the table correspond to the sample ID codes on the spectra.

Sequence	Sample ID Code
CGCAAXATCG	ASAXA
CGCAAXATC	1
CGCACXATCG	2
CGCAGXCTCG	3
CGAT/8oxoG/TTGCG	4
CGAT/O6MeG/TTGCG	5
CGAT/Neb/TTGCG	6
CGAT/N6MeA/TTGCG	7
CGAT/N6MeA/GTGCG	8
CGAT/Neb/GTGCG	9
CGAT/O6MeG/GTGCG	10
CGAT/8oxoG/GTGCG	11
CGAG/8oxoG/CTGCG	12
CGAG/O6MeG/CTGCG	13
CGAG/Neb/CTGCG	14
CGAG/N6MeA/CTGCG	15

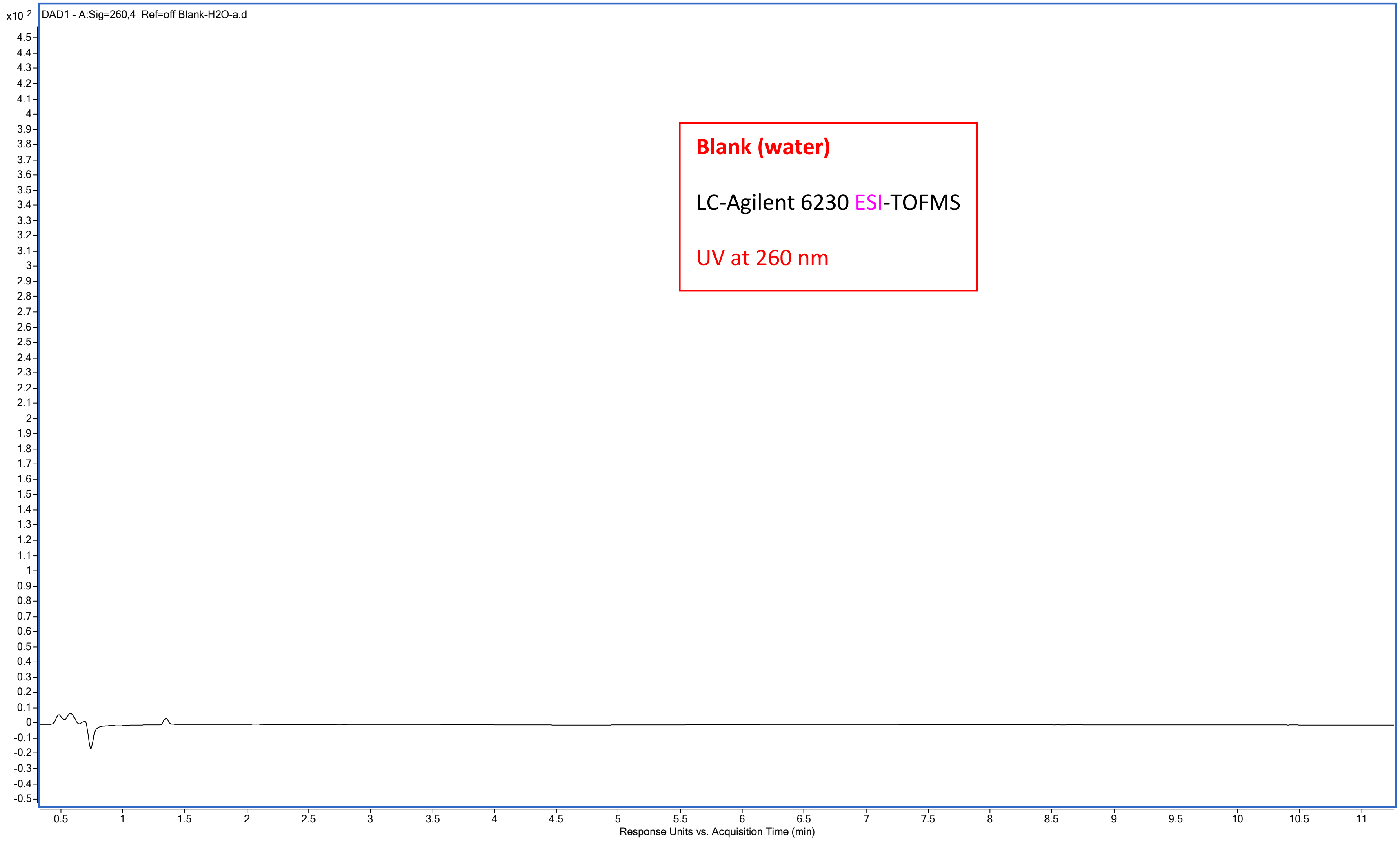
x10 2
DAD1 - A:Sig=260,4 Ref=off Blank-H2O-a.d
Blank (water)
LC-Agilent 6230 ESI-TOFMS
UV at 260 nm
Response Units vs. Acquisition Time (min)

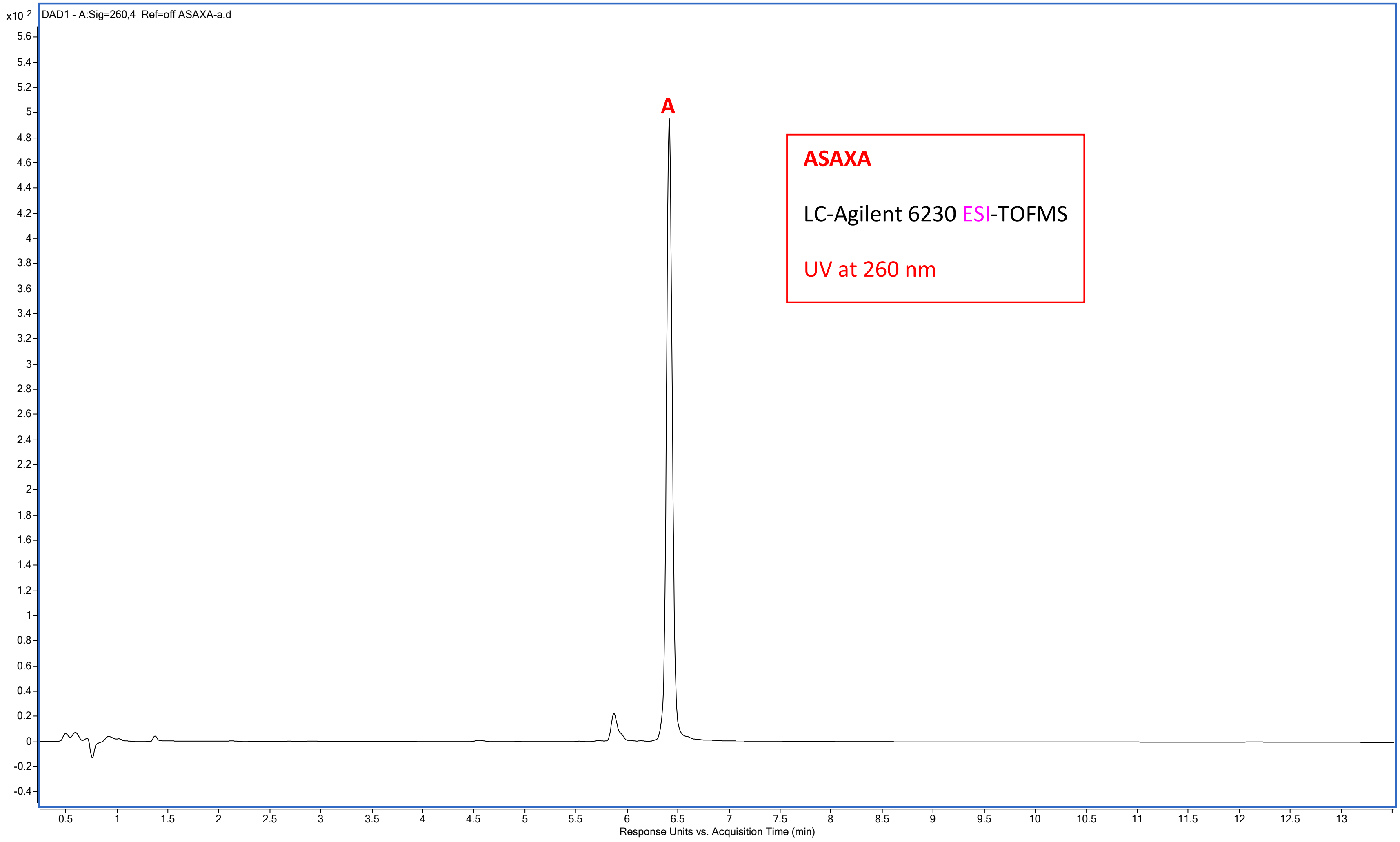

x10 2
DAD1 - A:Sig=260,4 Ref=off ASAXA-a.d
5.6
5.4
5.2
5
4.8
4.6
4.4
4.2
4
3.8
3.6
3.4
3.2
3
2.8
2.6
2.4
2.2
2
1.8
1.6
1.4
1.2
1
0.8
0.6
0.4
0.2
0
-0.2
-0.4
A
ASAXA
LC-Agilent 6230 ESI-TOFMS
UV at 260 nm
0.5 1 1.5 2 2.5 3 3.5 4 4.5 5 5.5 6 6.5 7 7.5 8 8.5 9 9.5 10 10.5 11 11.5 12 12.5 13
Response Units vs. Acquisition Time (min)

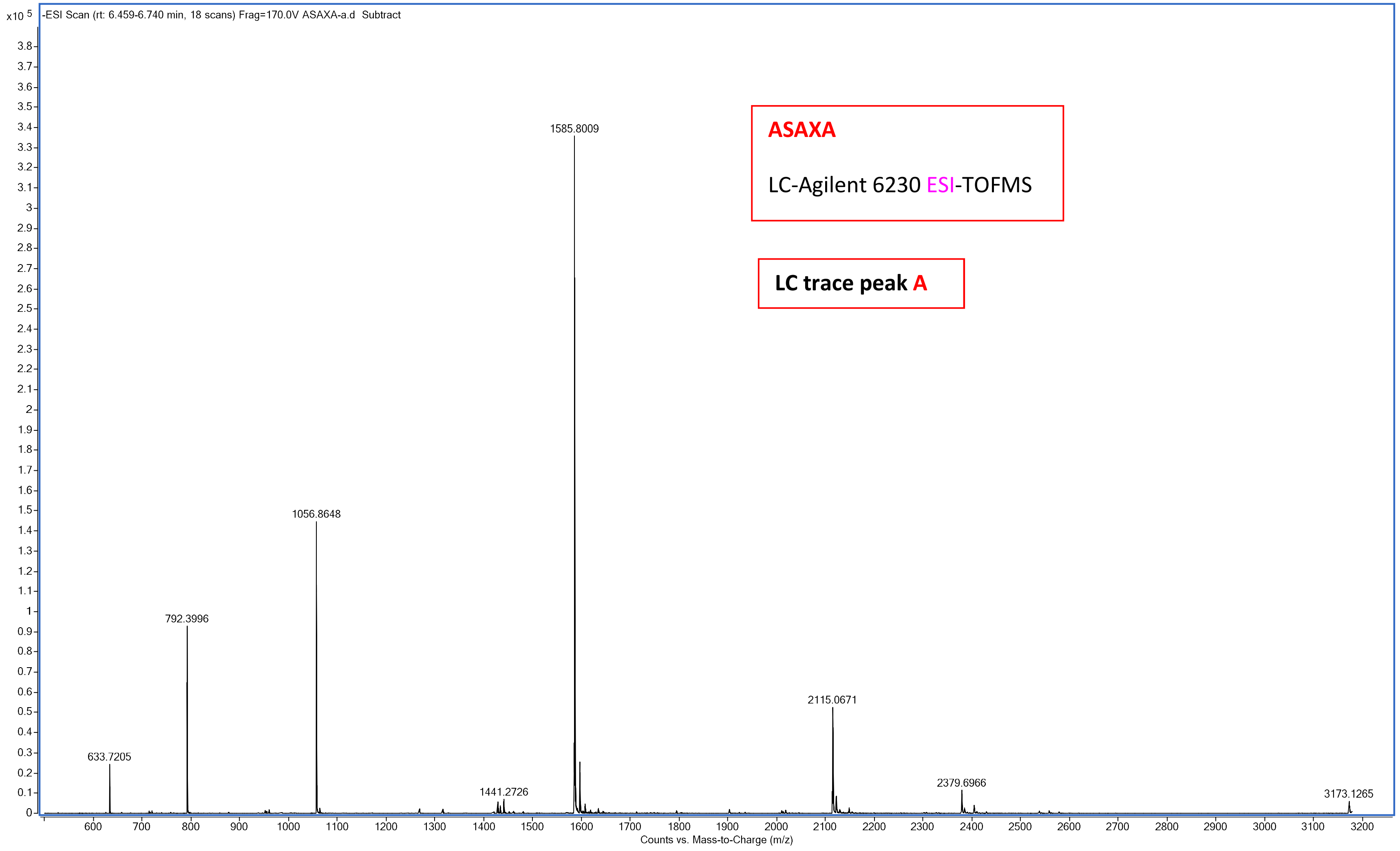
x10 5
-ESI Scan (rt: 6.459-6.740 min, 18 scans) Frag=170.0V ASAXA-a.d Subtract
ASAXA
LC-Agilent 6230 ESI-TOFMS
LC trace peak A
1585.8009
1056.8648
792.3996
633.7205
1441.2726
2115.0671
2379.6966
3173.1265
Counts vs. Mass-to-Charge (m/z)

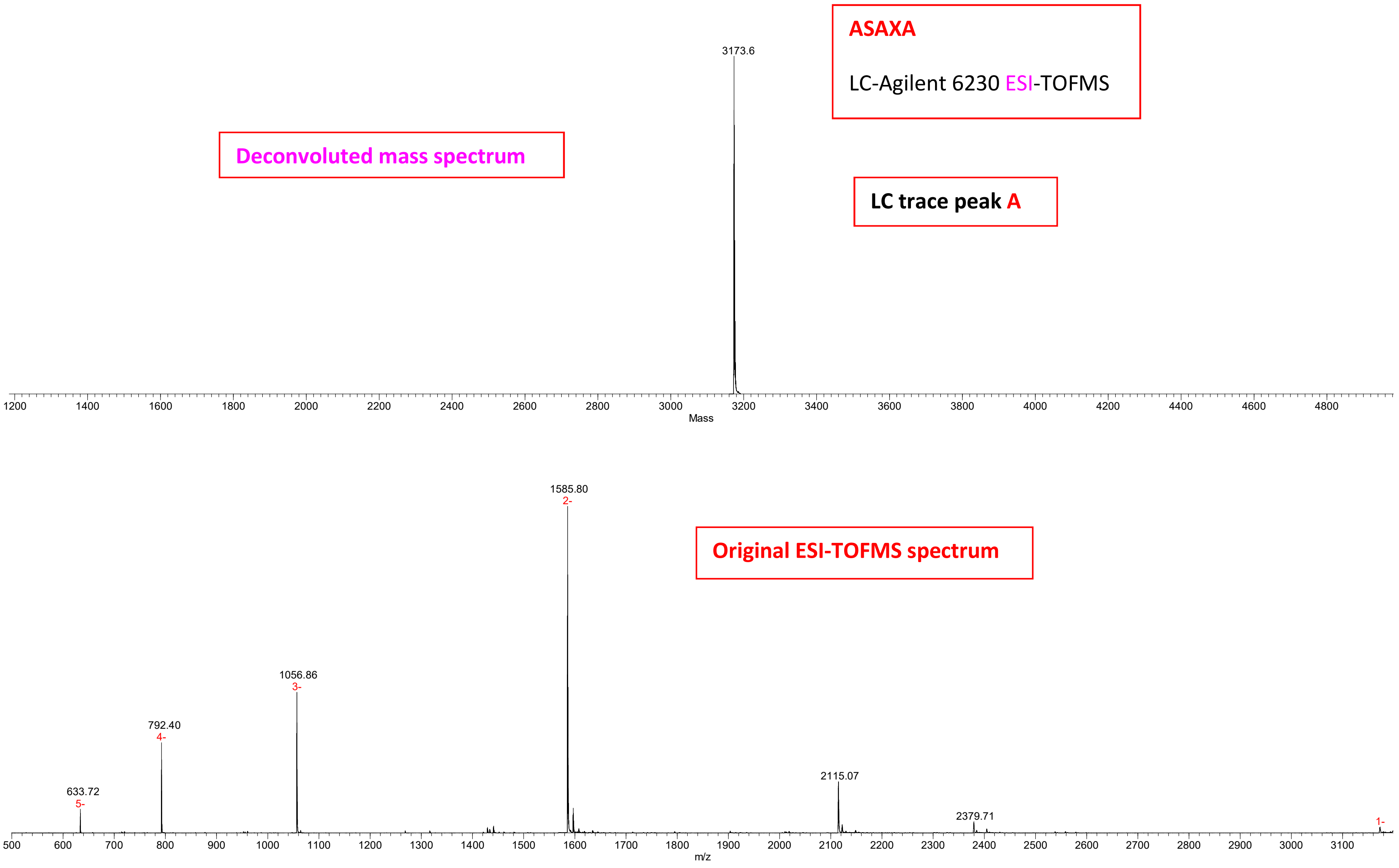
ASAXA
LC-Agilent 6230 ESI-TOFMS
Deconvoluted mass spectrum
LC trace peak A
3173.6
1200
1400
1600
1800
2000
2200
2400
2600
2800
3000
3200
3400
3600
3800
4000
4200
4400
4600
4800
Mass
Original ESI-TOFMS spectrum
1585.80
2-
1056.86
3-
792.40
4-
633.72
5-
2115.07
2379.71
1-
500
600
700
800
900
1000
1100
1200
1300
1400
1500
1600
1700
1800
1900
2000
2100
2200
2300
2400
2500
2600
2700
2800
2900
3000
3100
m/z

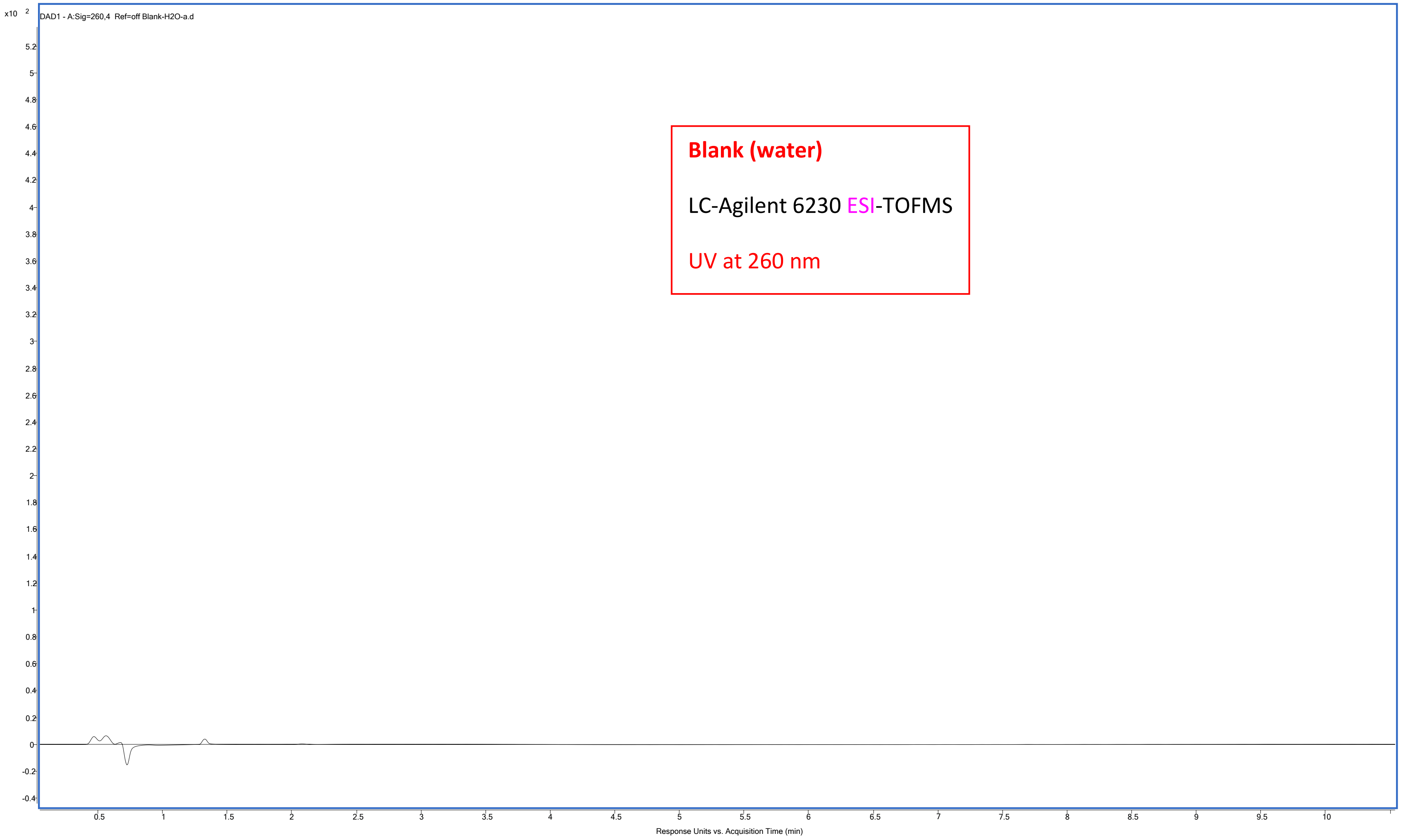

x10 2
DAD1 - A:Sig=260,4 Ref=off Blank-H2O-a.d
Blank (water)
LC-Agilent 6230 ESI-TOFMS
UV at 260 nm
5.2
5
4.8
4.6
4.4
4.2
4
3.8
3.6
3.4
3.2
3
2.8
2.6
2.4
2.2
2
1.8
1.6
1.4
1.2
1
0.8
0.6
0.4
0.2
0
-0.2
-0.4
0.5
1
1.5
2
2.5
3
3.5
4
4.5
5
5.5
6
6.5
7
7.5
8
8.5
9
9.5
10
Response Units vs. Acquisition Time (min)

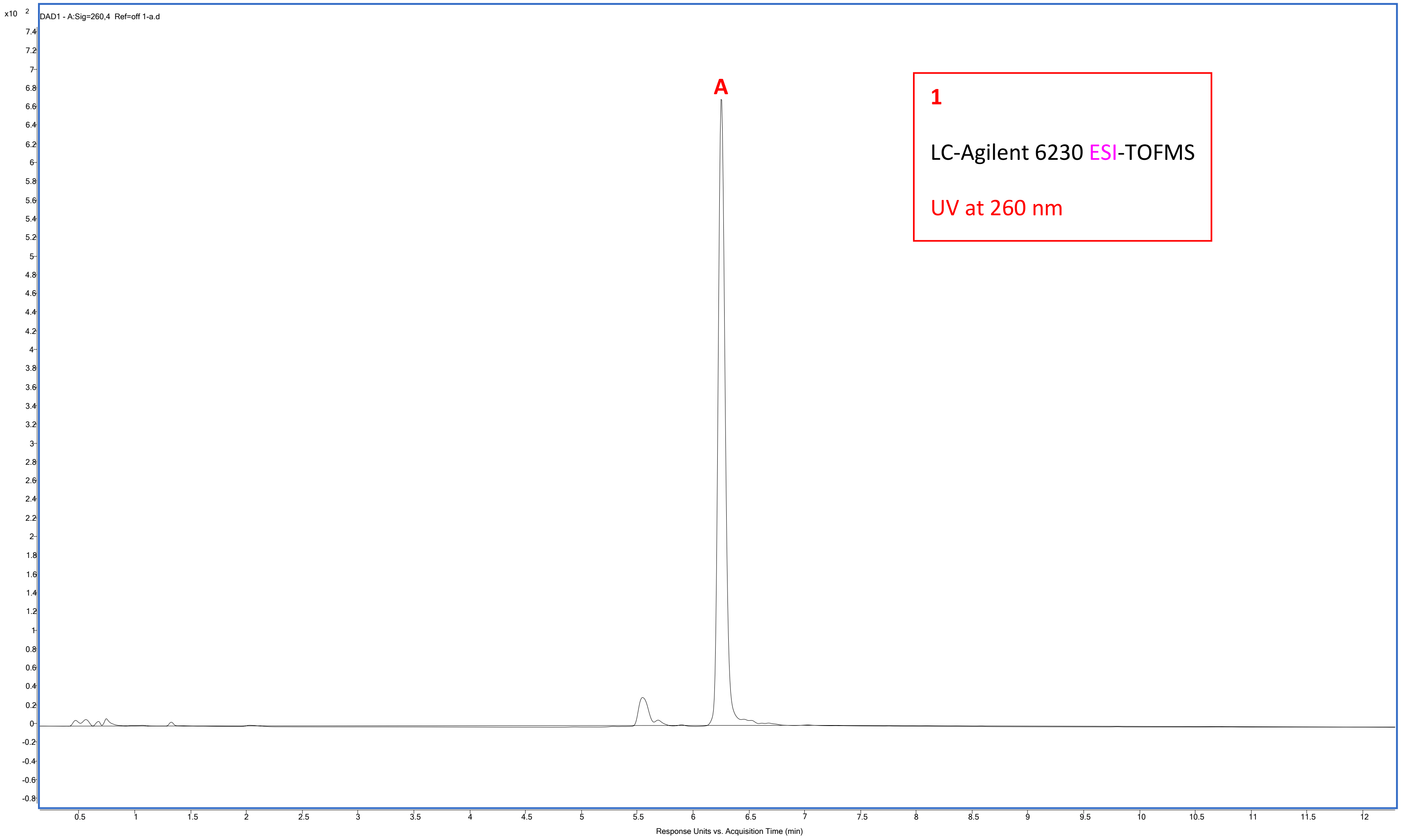

x10 2
DAD1 - A:Sig=260,4 Ref=off 1-a.d
A
1
LC-Agilent 6230 ESI-TOFMS
UV at 260 nm
Response Units vs. Acquisition Time (min)

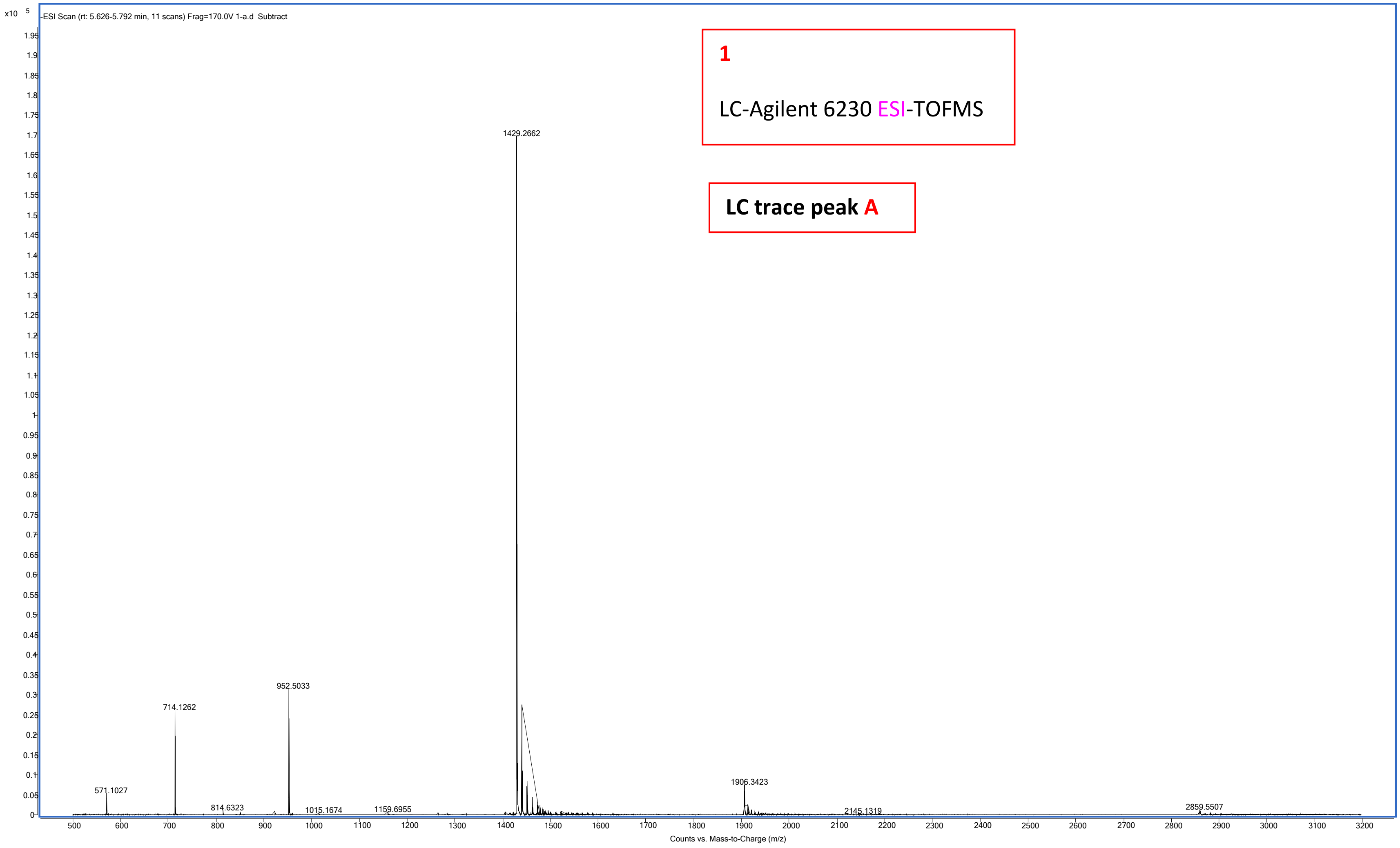

x10 5
-ESI Scan (rt: 5.626-5.792 min, 11 scans) Frag=170.0V 1-a.d Subtract
1
LC-Agilent 6230 ESI-TOFMS
LC trace peak A
1429.2662
952.5033
714.1262
571.1027
814.6323
1015.1674
1159.6955
1906.3423
2145.1319
2859.5507
Counts vs. Mass-to-Charge (m/z)

1

LC-Agilent 6230 ESI-TOFMS

Deconvoluted mass spectrum

LC trace peak A

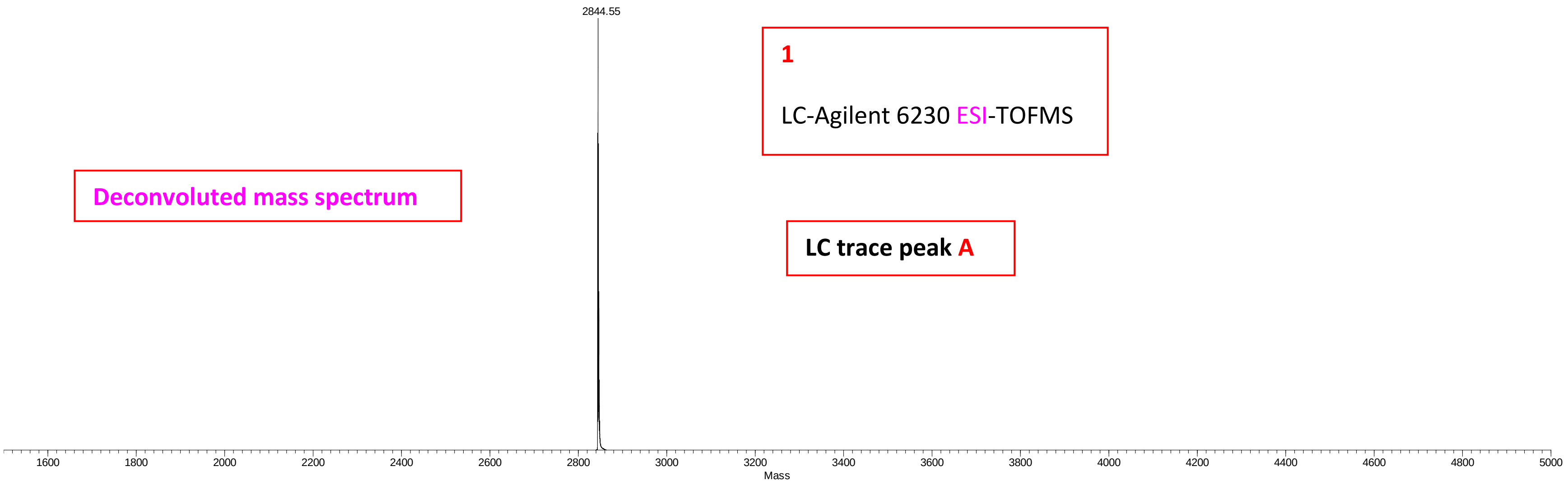

Original ESI-TOFMS spectrum

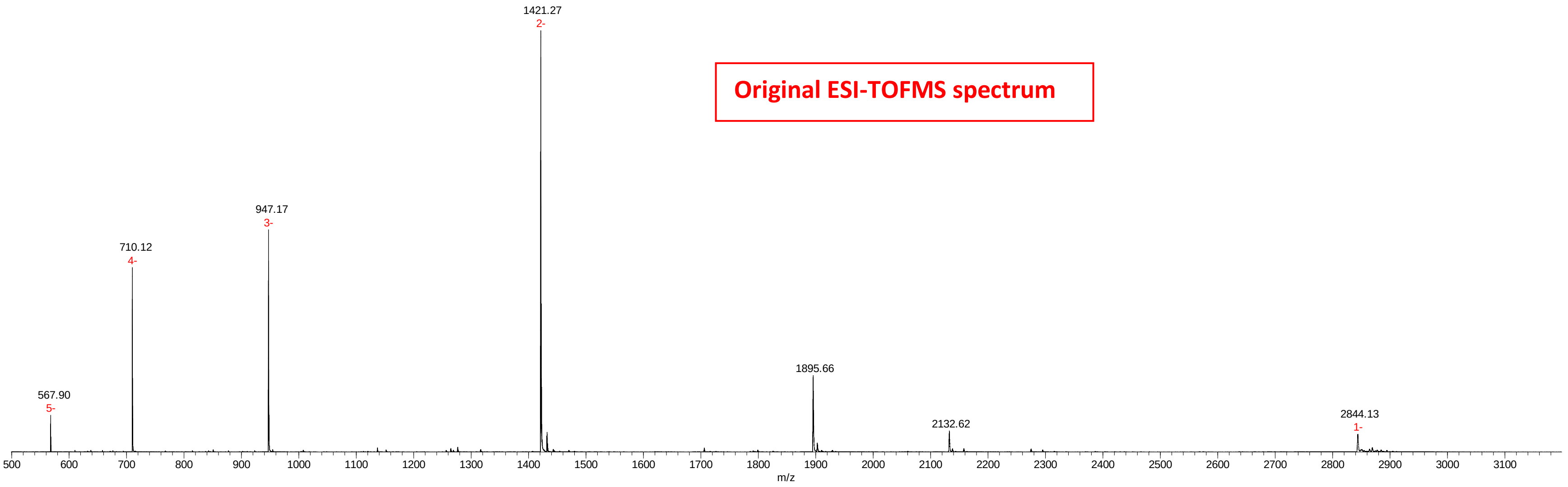

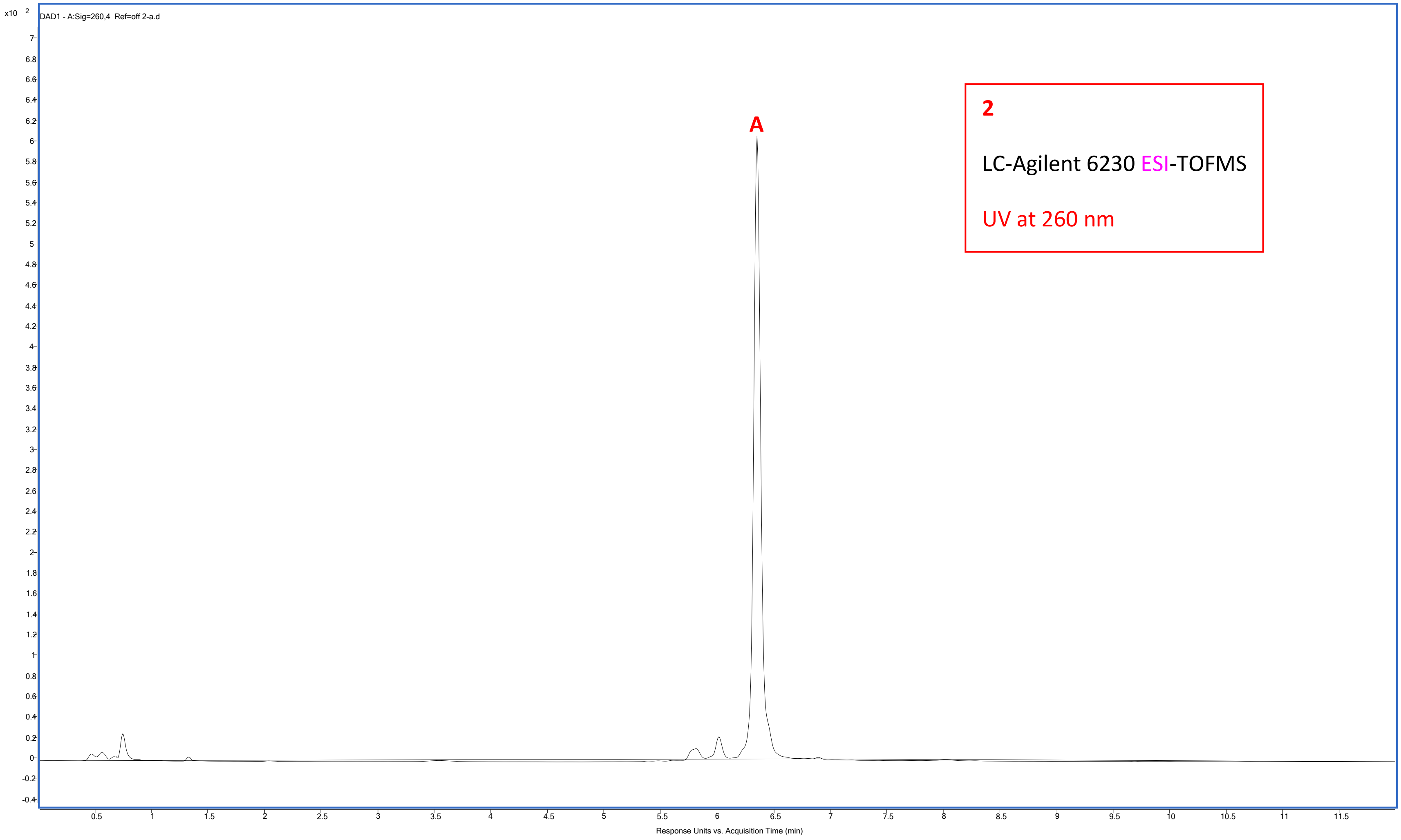

x10 2
DAD1 - A:Sig=260,4 Ref=off 2-a.d
A
2
LC-Agilent 6230 ESI-TOFMS
UV at 260 nm
Response Units vs. Acquisition Time (min)

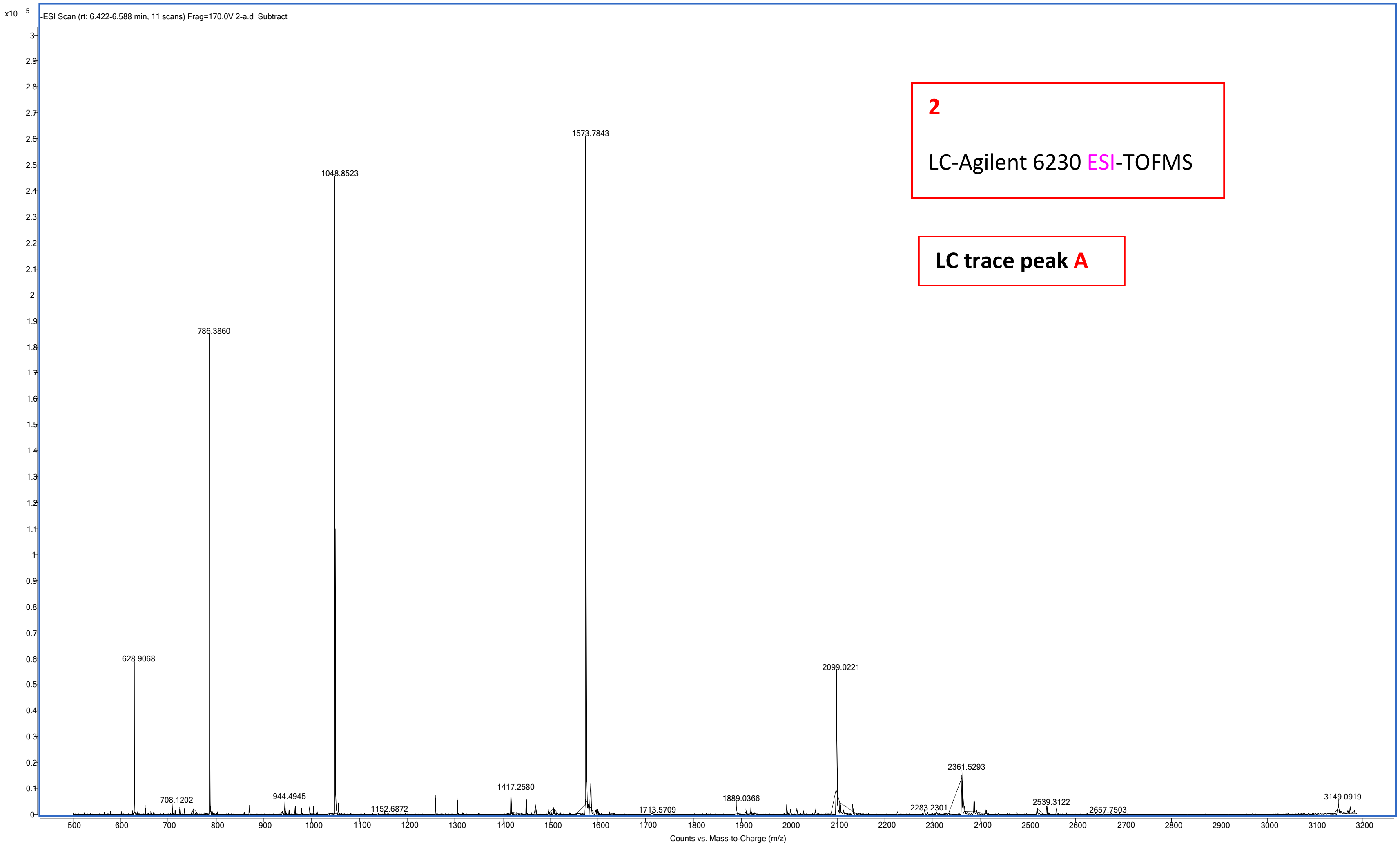

x10 5
-ESI Scan (rt: 6.422-6.588 min, 11 scans) Frag=170.0V 2-a.d Subtract
1573.7843
1048.8523
786.3860
628.9068
708.1202
944.4945
1152.6872
1417.2580
1713.5709
1889.0366
2099.0221
2283.2301
2361.5293
2539.3122
2657.7503
3149.0919
Counts vs. Mass-to-Charge (m/z)
2
LC-Agilent 6230 ESI-TOFMS
LC trace peak A

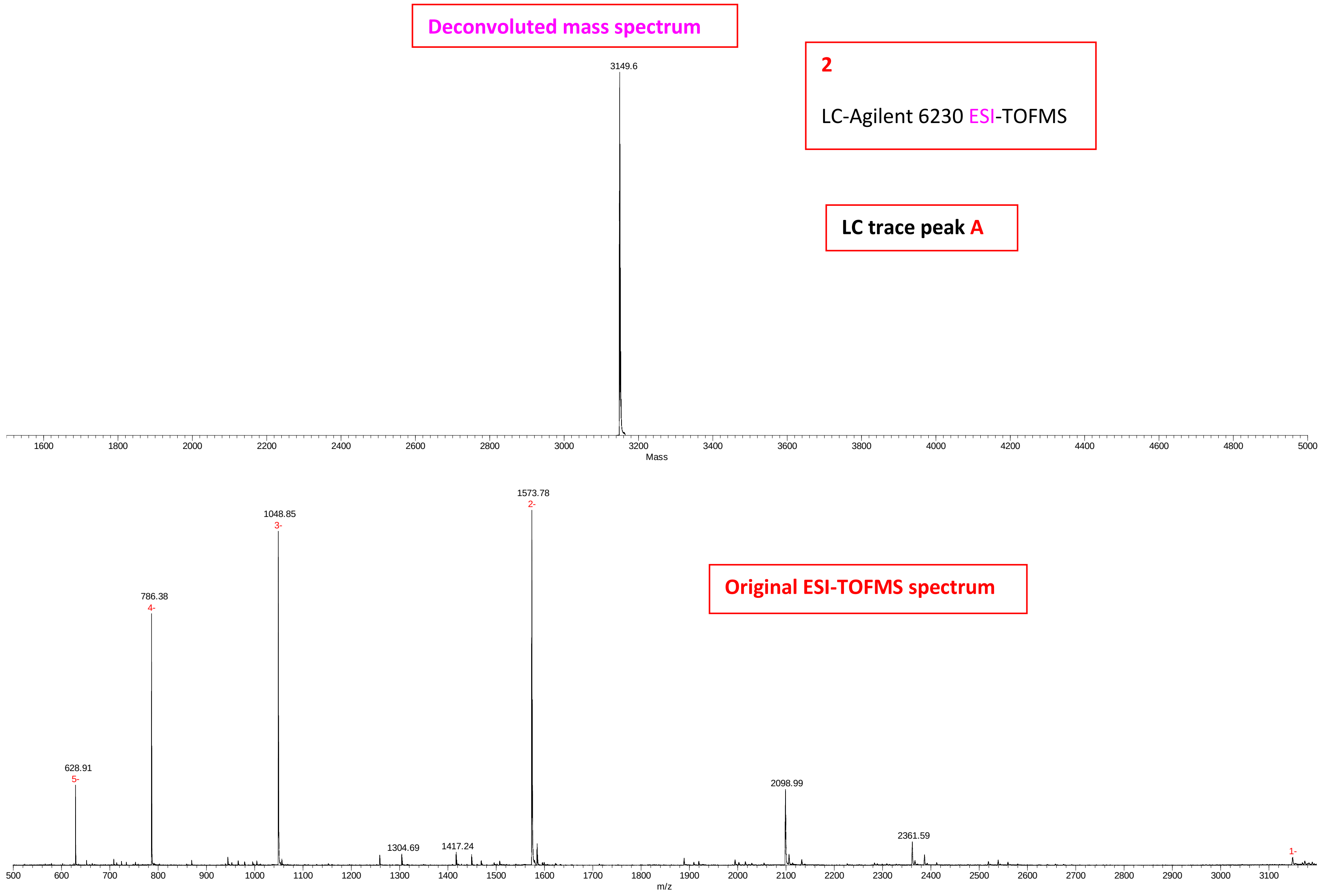

Deconvoluted mass spectrum
2
LC-Agilent 6230 ESI-TOFMS
LC trace peak A
3149.6
1600
1800
2000
2200
2400
2600
2800
3000
3200
3400
3600
3800
4000
4200
4400
4600
4800
5000
Mass
Original ESI-TOFMS spectrum
1573.78
2-
1048.85
3-
786.38
4-
628.91
5-
1304.69
1417.24
2098.99
2361.59
1-
500
600
700
800
900
1000
1100
1200
1300
1400
1500
1600
1700
1800
1900
2000
2100
2200
2300
2400
2500
2600
2700
2800
2900
3000
3100
m/z

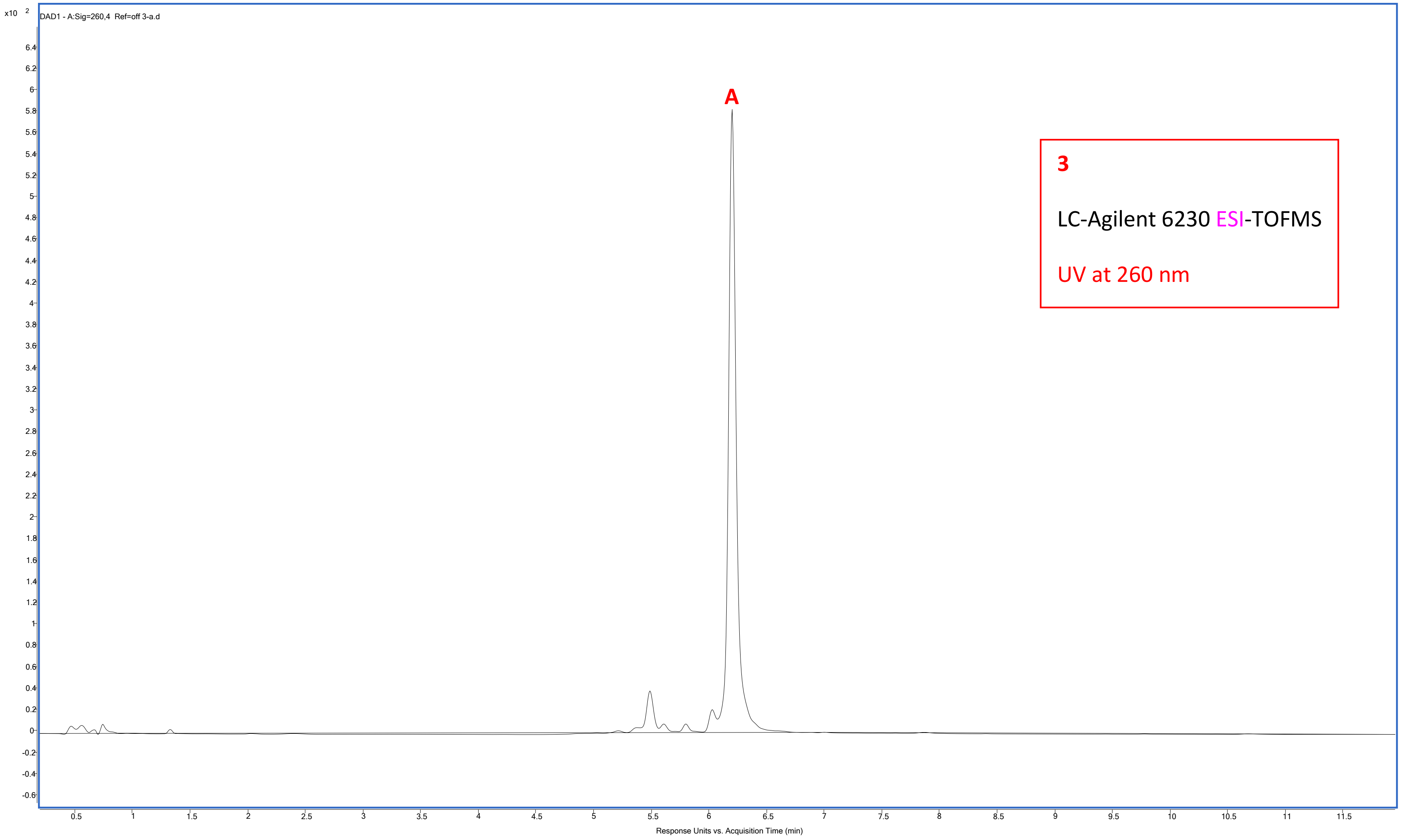

x10 2
DAD1 - A:Sig=260,4 Ref=off 3-a.d
A
3
LC-Agilent 6230 ESI-TOFMS
UV at 260 nm
Response Units vs. Acquisition Time (min)

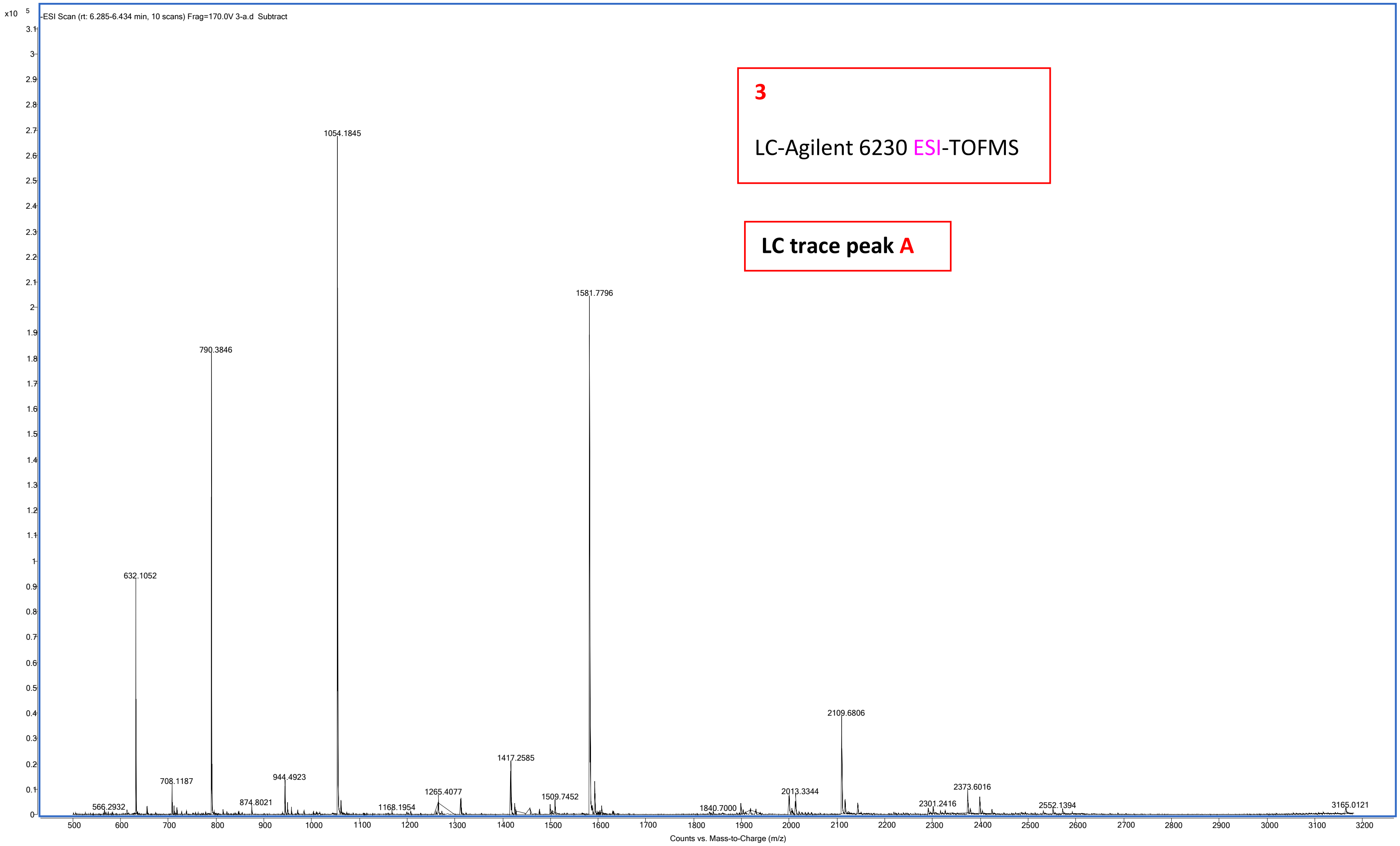
x10 5
-ESI Scan (rt: 6.285-6.434 min, 10 scans) Frag=170.0V 3-a.d Subtract
3
LC-Agilent 6230 ESI-TOFMS
LC trace peak A
1054.1845
1581.7796
790.3846
632.1052
2109.6806
1417.2585
944.4923
708.1187
1265.4077
2373.6016
2013.3344
1509.7452
874.8021
566.2932
1168.1954
1840.7000
2301.2416
2552.1394
3165.0121
Counts vs. Mass-to-Charge (m/z)

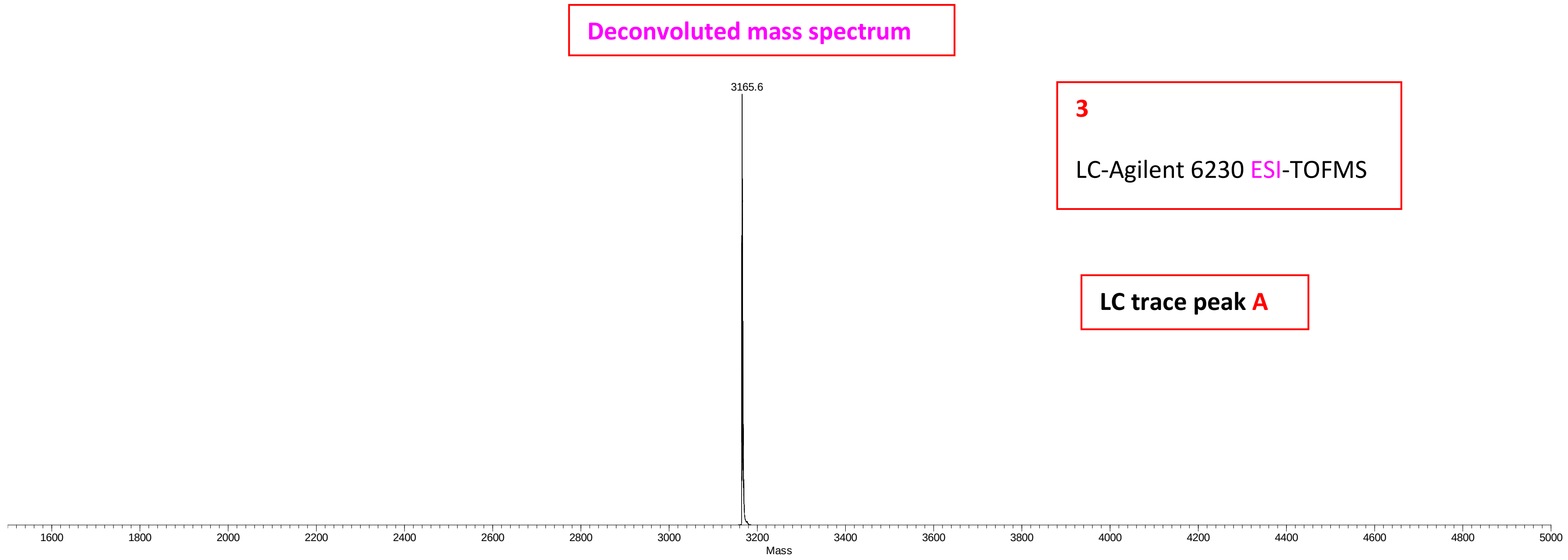
Deconvoluted mass spectrum
3165.6
3
LC-Agilent 6230 ESI-TOFMS
LC trace peak A
1600
1800
2000
2200
2400
2600
2800
3000
3200
3400
3600
3800
4000
4200
4400
4600
4800
5000
Mass

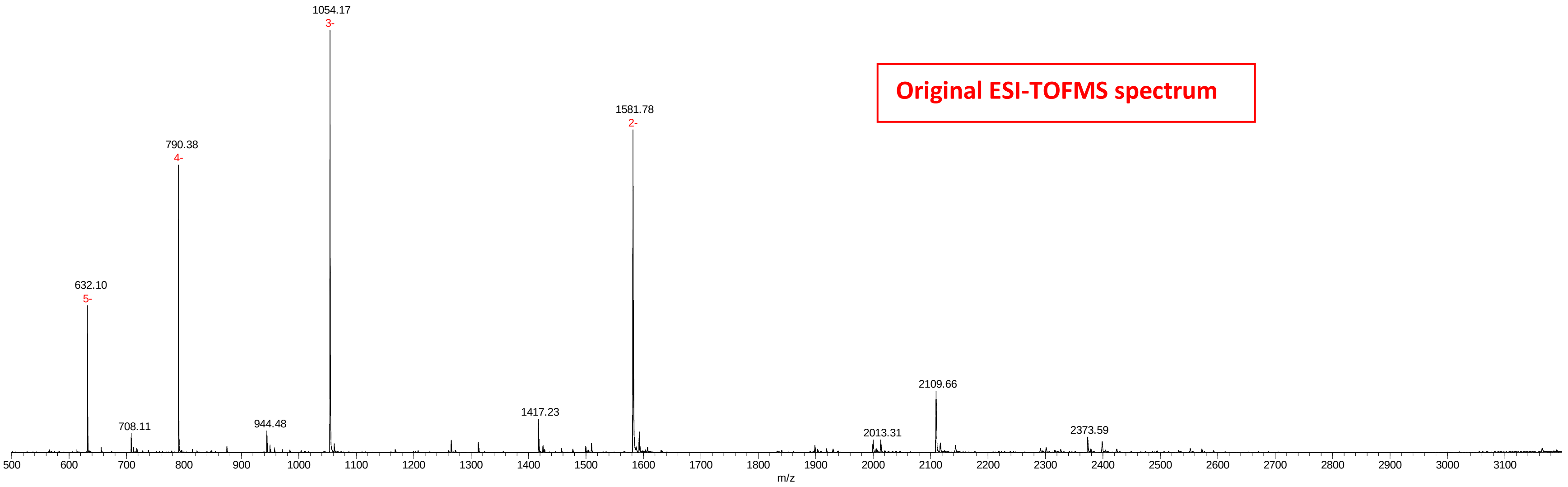
1054.17
3-
Original ESI-TOFMS spectrum
1581.78
2-
790.38
4-
632.10
5-
708.11
944.48
1417.23
2109.66
2013.31
2373.59
500
600
700
800
900
1000
1100
1200
1300
1400
1500
1600
1700
1800
1900
2000
2100
2200
2300
2400
2500
2600
2700
2800
2900
3000
3100
m/z

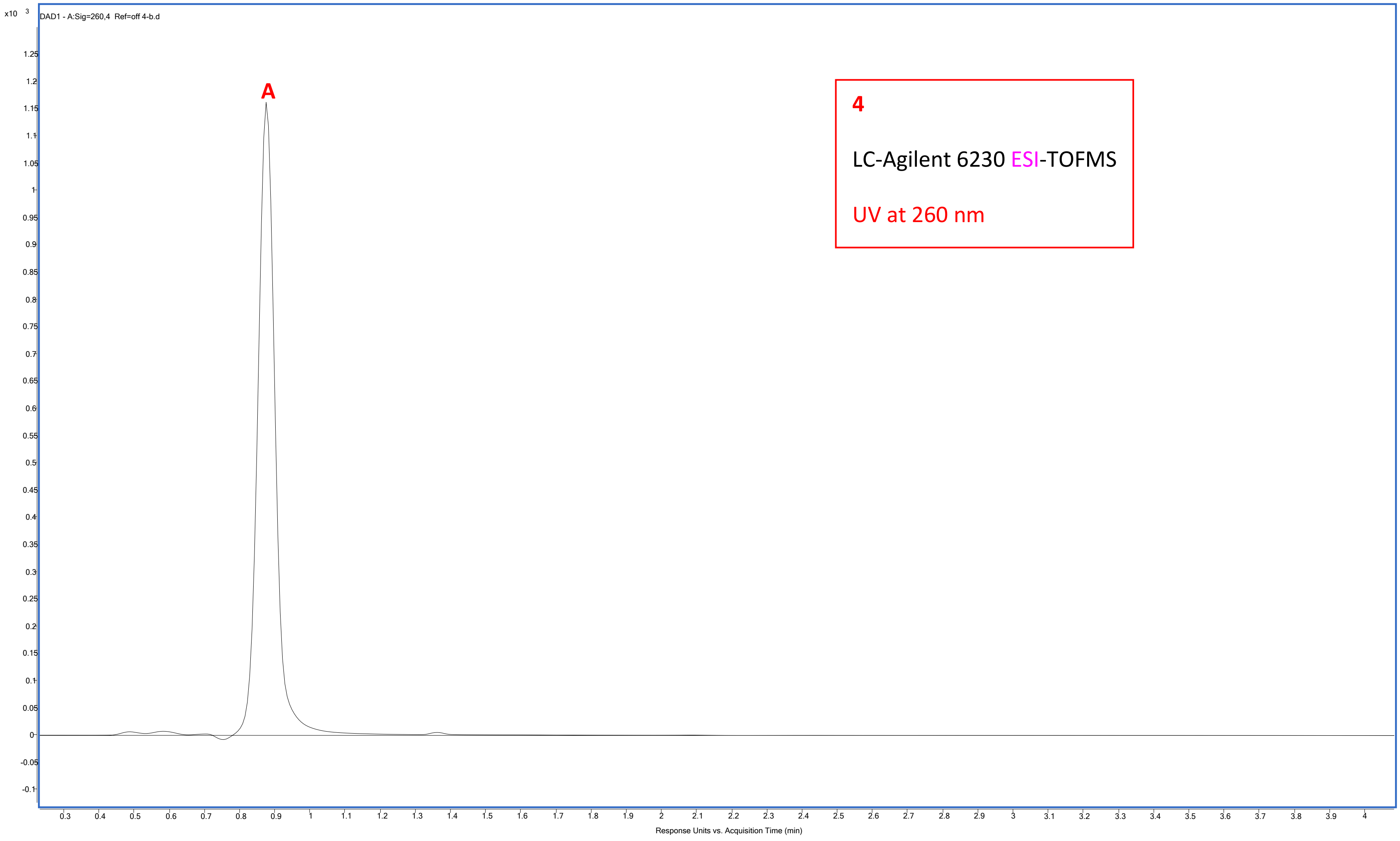
x10 3
DAD1 - A:Sig=260,4 Ref=off 4-b.d
A
4
LC-Agilent 6230 ESI-TOFMS
UV at 260 nm
Response Units vs. Acquisition Time (min)

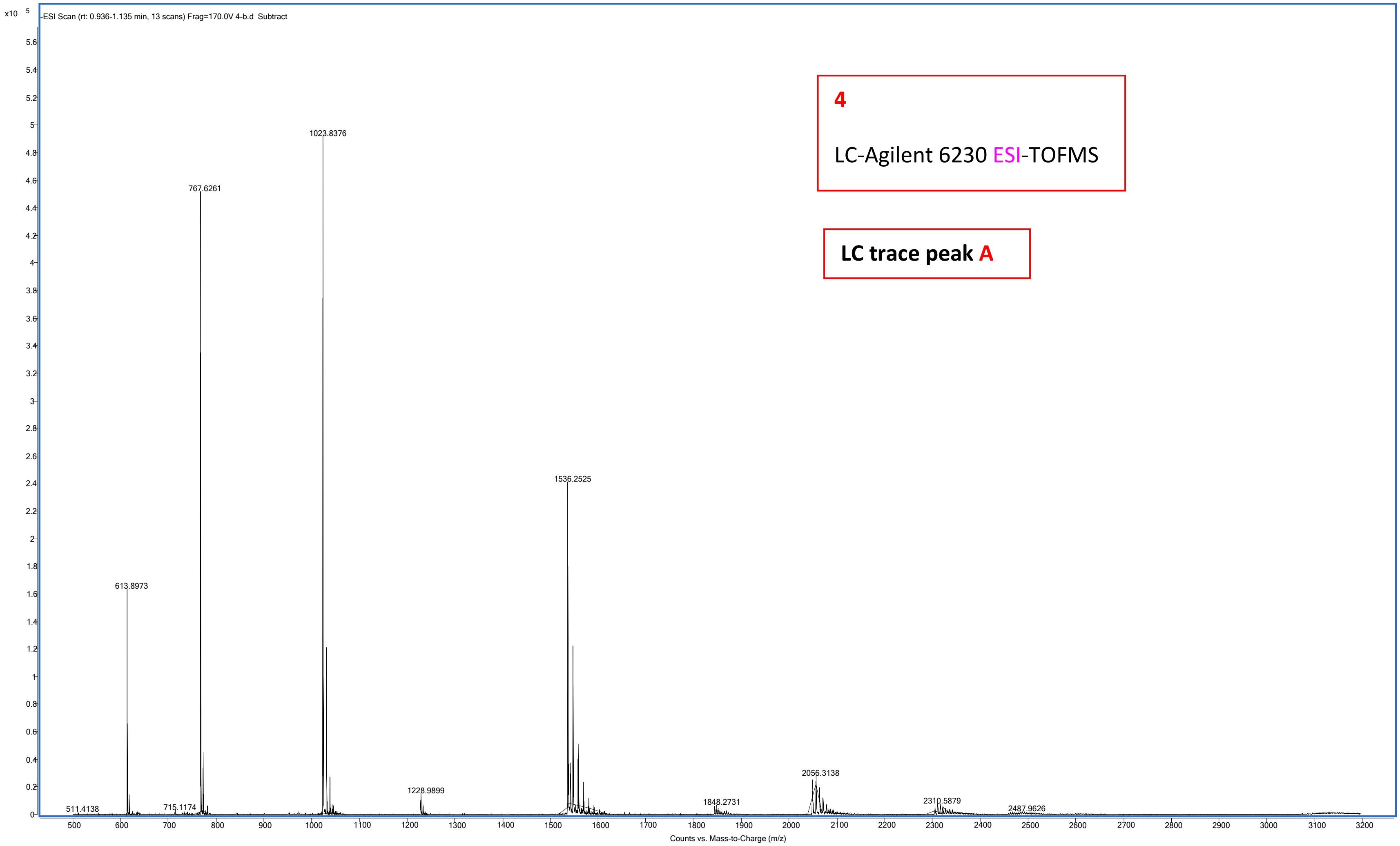

4

LC-Agilent 6230 ESI-TOFMS

LC trace peak A

Deconvoluted mass spectrum

4

LC-Agilent 6230 ESI-TOFMS

LC trace peak A

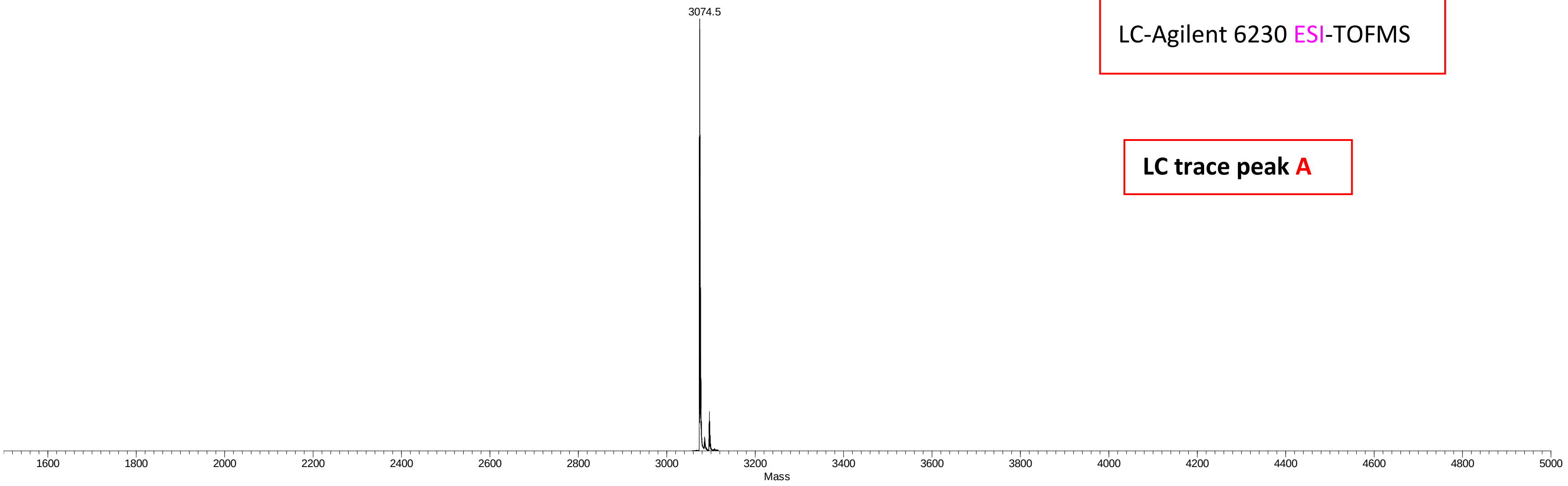

Original ESI-TOFMS spectrum

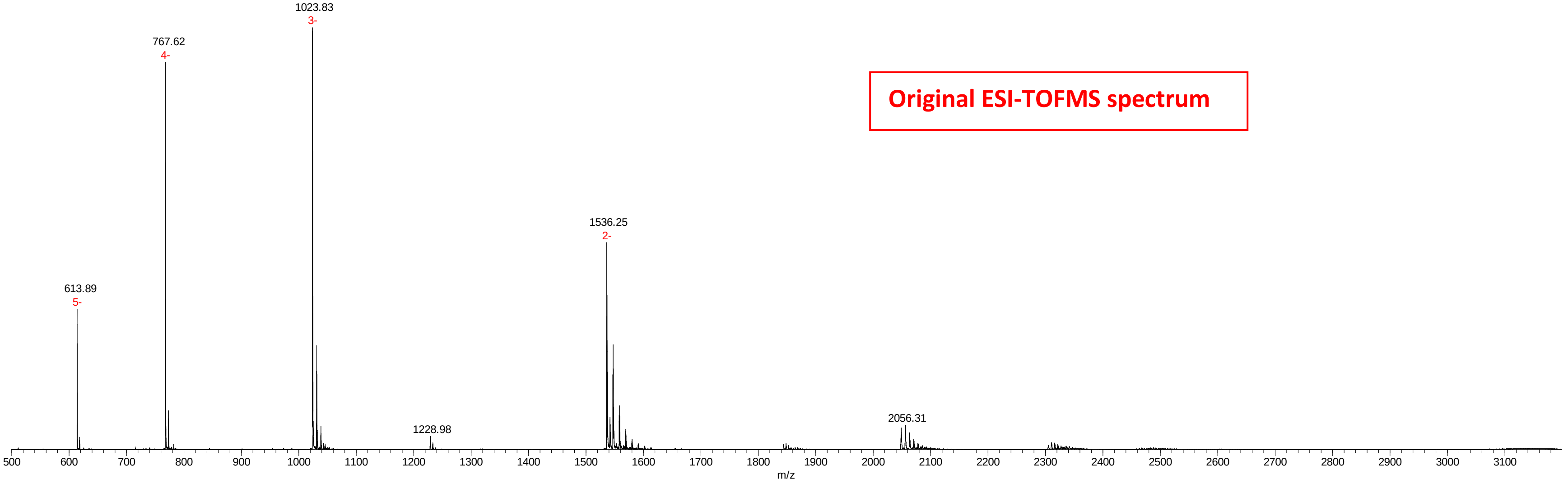

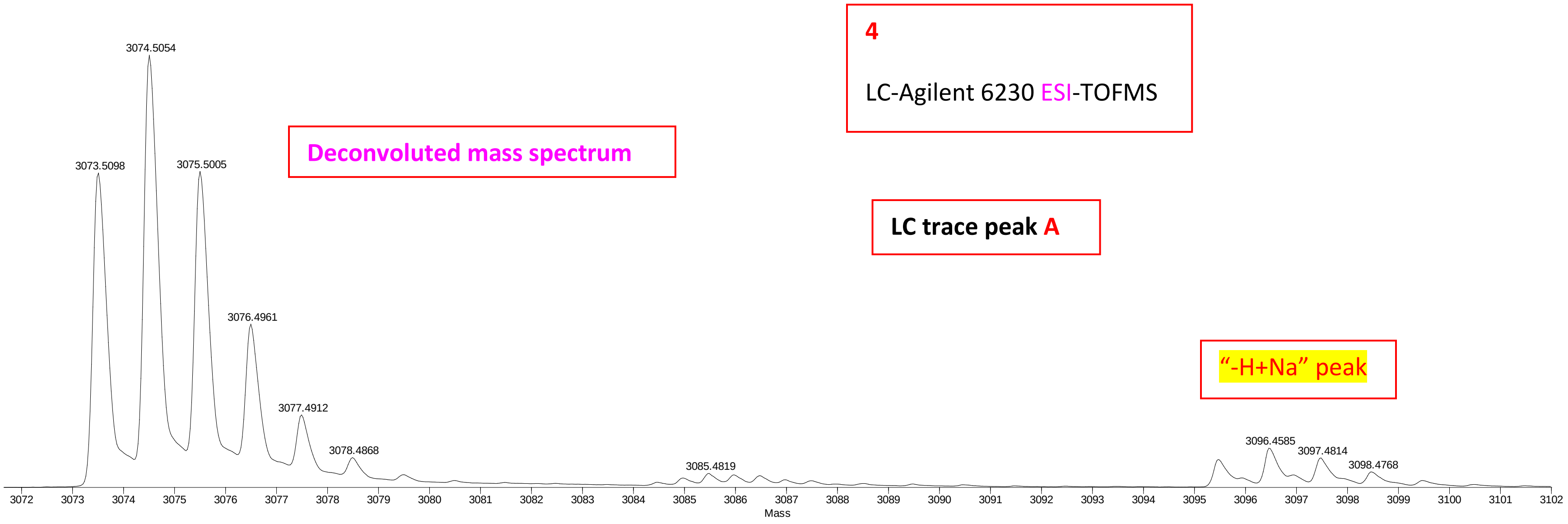
4
LC-Agilent 6230 ESI-TOFMS
Deconvoluted mass spectrum
LC trace peak A
“-H+Na” peak
3073.5098
3074.5054
3075.5005
3076.4961
3077.4912
3078.4868
3085.4819
3096.4585
3097.4814
3098.4768
3072
3073
3074
3075
3076
3077
3078
3079
3080
3081
3082
3083
3084
3085
3086
3087
3088
3089
3090
3091
3092
3093
3094
3095
3096
3097
3098
3099
3100
3101
3102
Mass

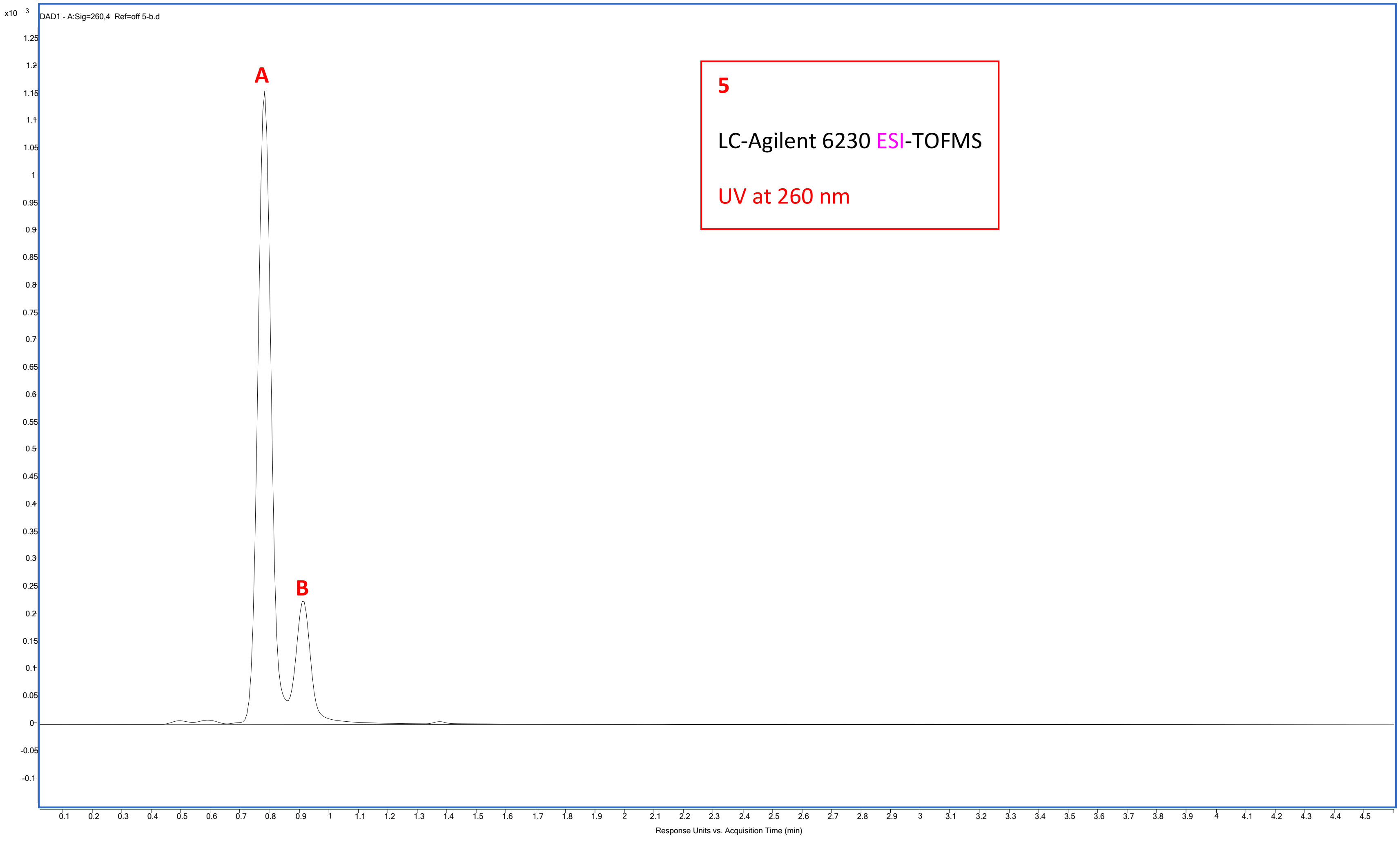
x10 3
DAD1 - A:Sig=260,4 Ref=off 5-b.d
A
B
5
LC-Agilent 6230 ESI-TOFMS
UV at 260 nm
Response Units vs. Acquisition Time (min)

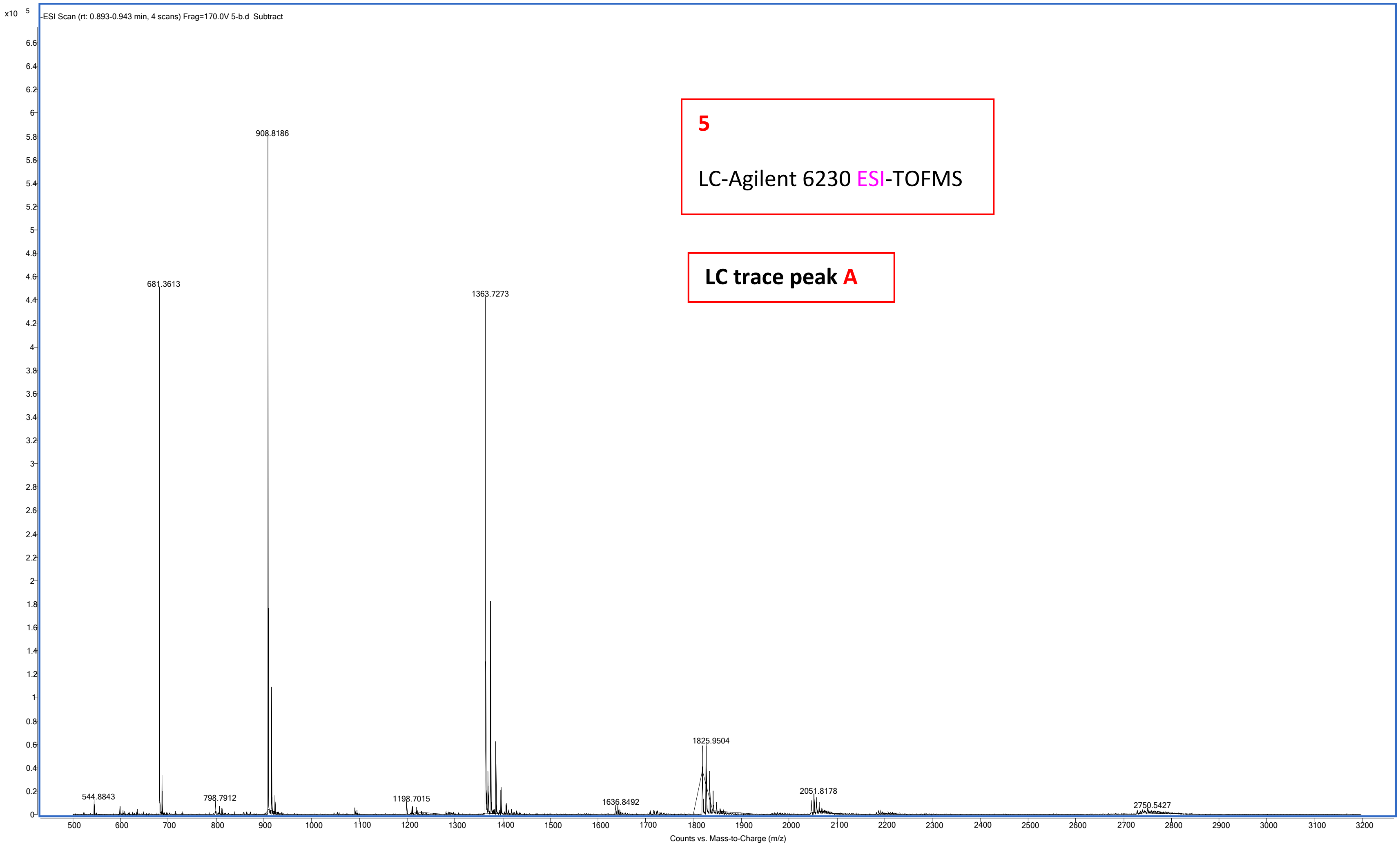
x10 5
-ESI Scan (rt: 0.893-0.943 min, 4 scans) Frag=170.0V 5-b.d Subtract
5
LC-Agilent 6230 ESI-TOFMS
LC trace peak A
908.8186
681.3613
1363.7273
1825.9504
2051.8178
544.8843
798.7912
1198.7015
1636.8492
2750.5427
Counts vs. Mass-to-Charge (m/z)

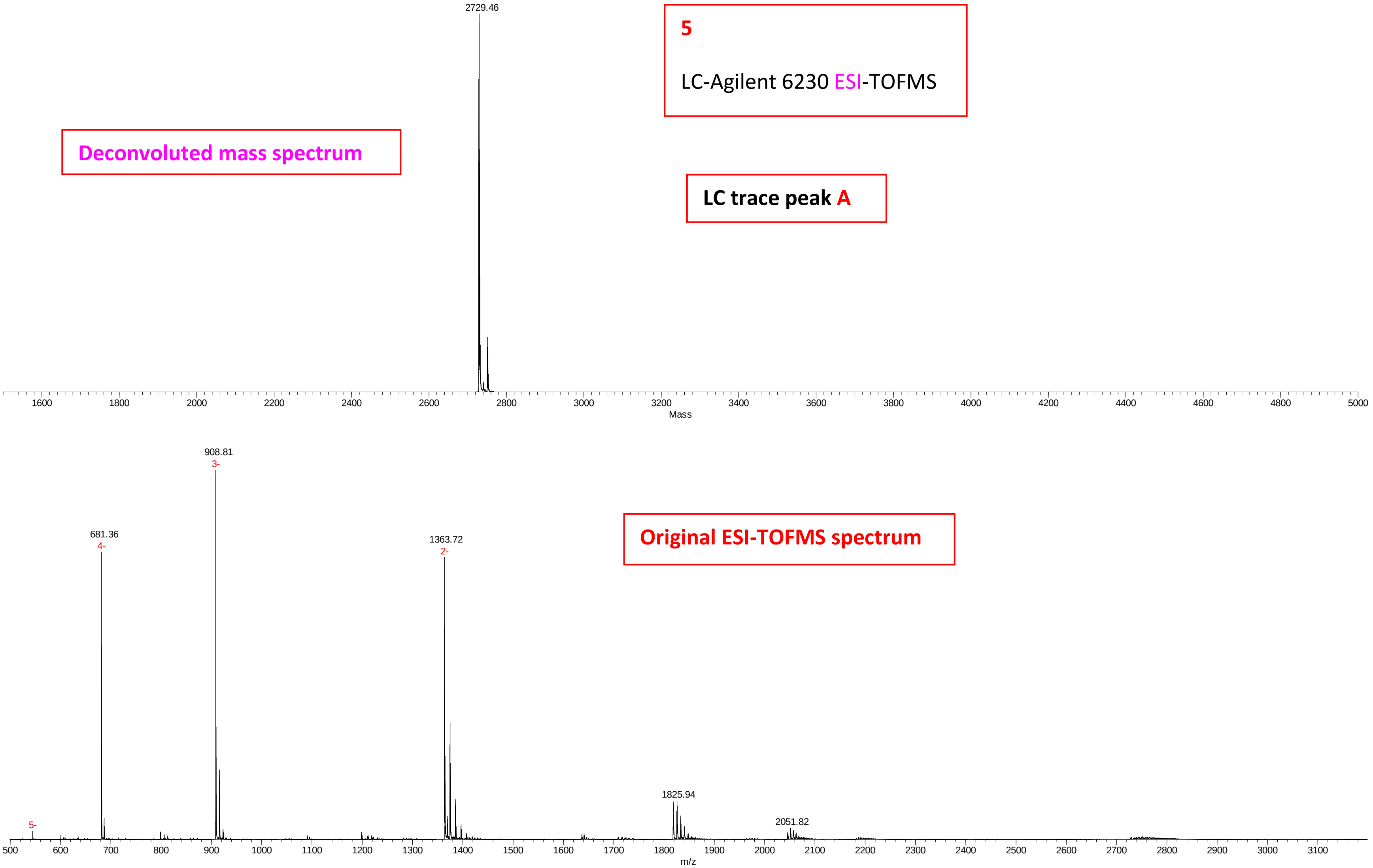

5
LC-Agilent 6230 ESI-TOFMS
Deconvoluted mass spectrum
LC trace peak A
2729.46
1600 1800 2000 2200 2400 2600 2800 3000 3200 3400 3600 3800 4000 4200 4400 4600 4800 5000
Mass
Original ESI-TOFMS spectrum
908.81
3-
681.36
4-
1363.72
2-
1825.94
2051.82
5-
500 600 700 800 900 1000 1100 1200 1300 1400 1500 1600 1700 1800 1900 2000 2100 2200 2300 2400 2500 2600 2700 2800 2900 3000 3100
m/z

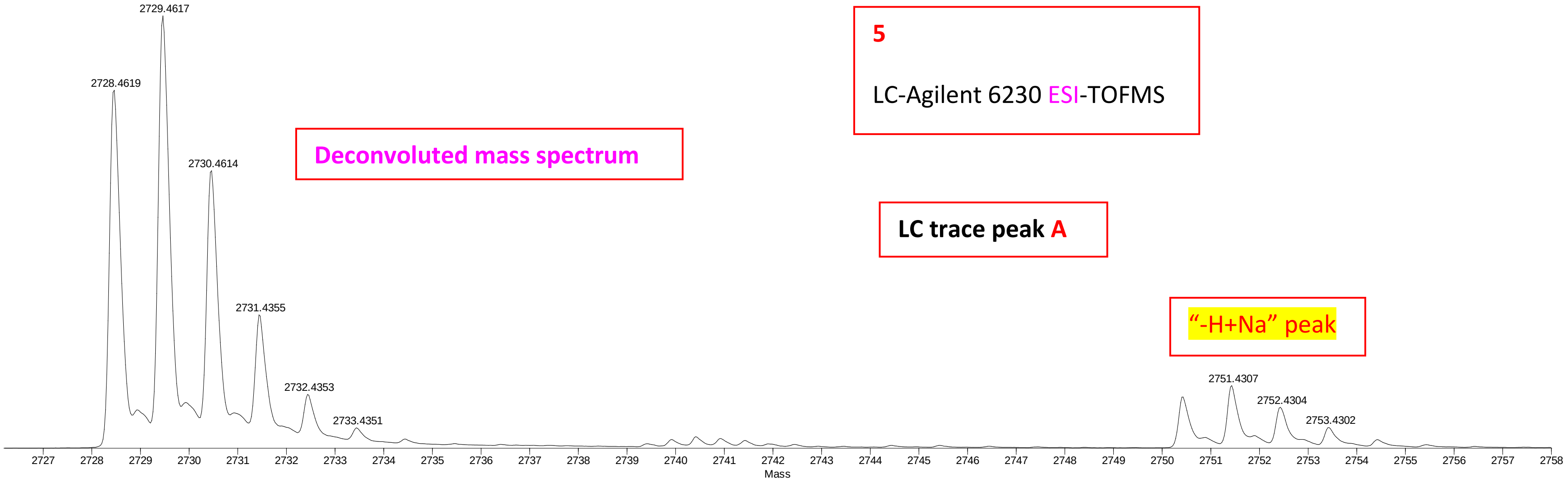
5
LC-Agilent 6230 ESI-TOFMS
Deconvoluted mass spectrum
LC trace peak A
“-H+Na” peak
2728.4619
2729.4617
2730.4614
2731.4355
2732.4353
2733.4351
2751.4307
2752.4304
2753.4302
2727 2728 2729 2730 2731 2732 2733 2734 2735 2736 2737 2738 2739 2740 2741 2742 2743 2744 2745 2746 2747 2748 2749 2750 2751 2752 2753 2754 2755 2756 2757 2758
Mass

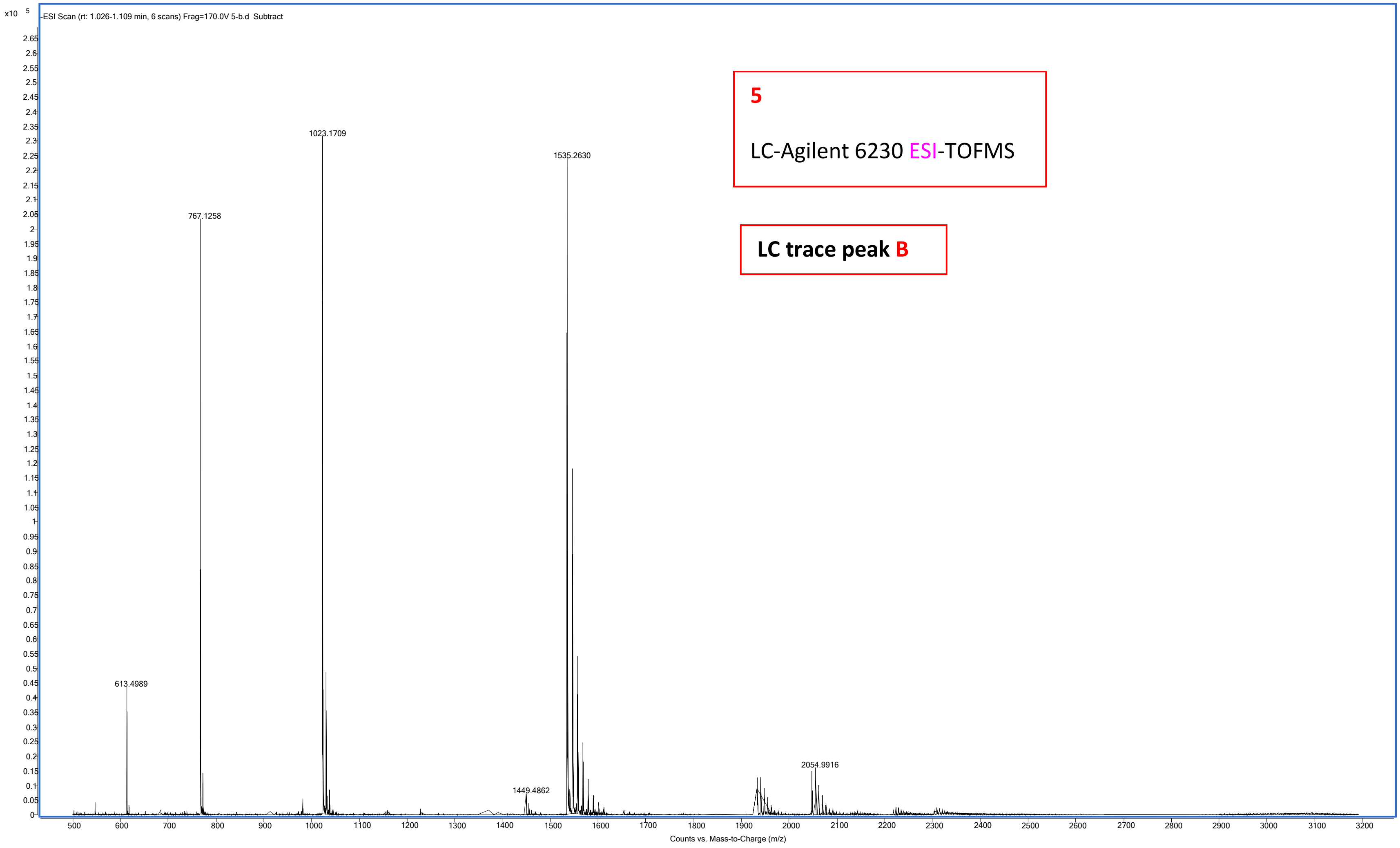
x10 5
-ESI Scan (rt: 1.026-1.109 min, 6 scans) Frag=170.0V 5-b.d Subtract
5
LC-Agilent 6230 ESI-TOFMS
LC trace peak B
1023.1709
1535.2630
767.1258
613.4989
1449.4862
2054.9916
Counts vs. Mass-to-Charge (m/z)

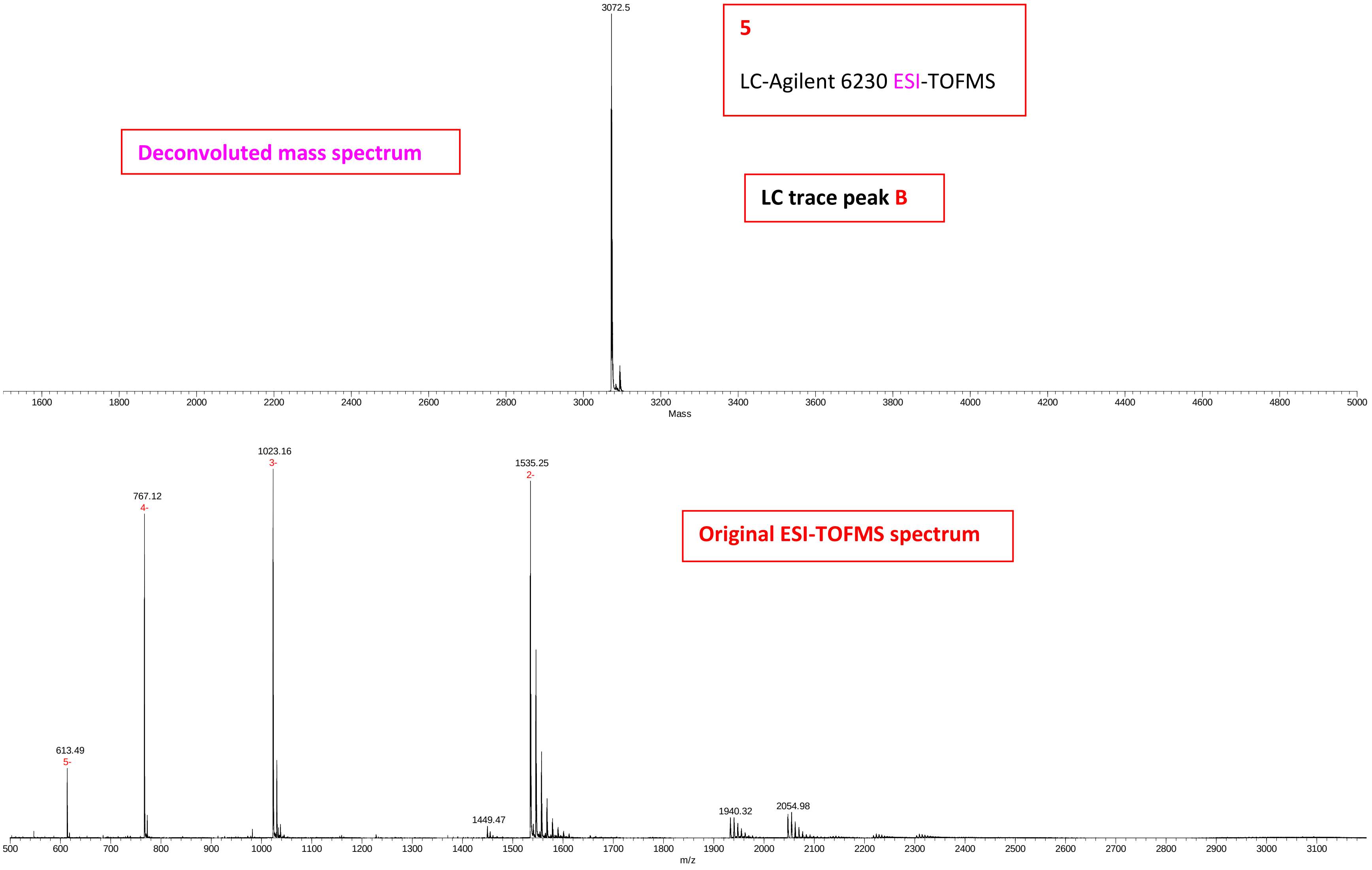

5
LC-Agilent 6230 ESI-TOFMS
Deconvoluted mass spectrum
LC trace peak B
3072.5
1600
1800
2000
2200
2400
2600
2800
3000
3200
3400
3600
3800
4000
4200
4400
4600
4800
5000
Mass
Original ESI-TOFMS spectrum
613.49
5-
767.12
4-
1023.16
3-
1449.47
1535.25
2-
1940.32
2054.98
500
600
700
800
900
1000
1100
1200
1300
1400
1500
1600
1700
1800
1900
2000
2100
2200
2300
2400
2500
2600
2700
2800
2900
3000
3100
m/z

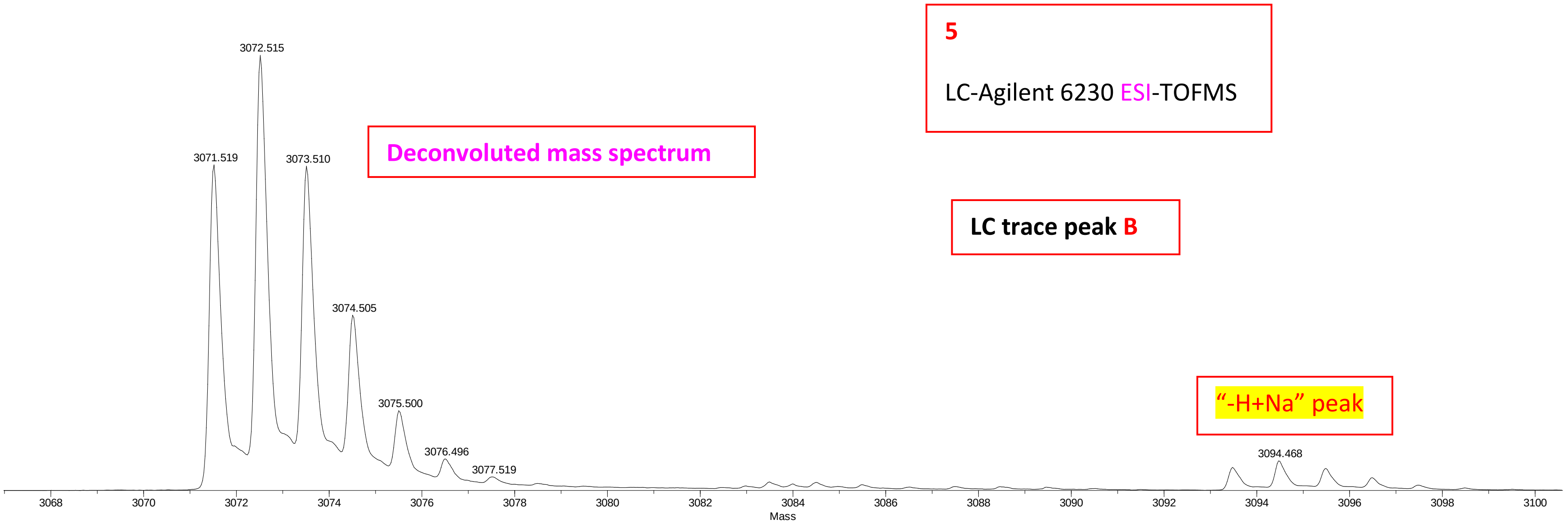
5
LC-Agilent 6230 ESI-TOFMS
Deconvoluted mass spectrum
LC trace peak B
“-H+Na” peak
3072.515
3071.519
3073.510
3074.505
3075.500
3076.496
3077.519
3094.468
3068
3070
3072
3074
3076
3078
3080
3082
3084
3086
3088
3090
3092
3094
3096
3098
3100
Mass

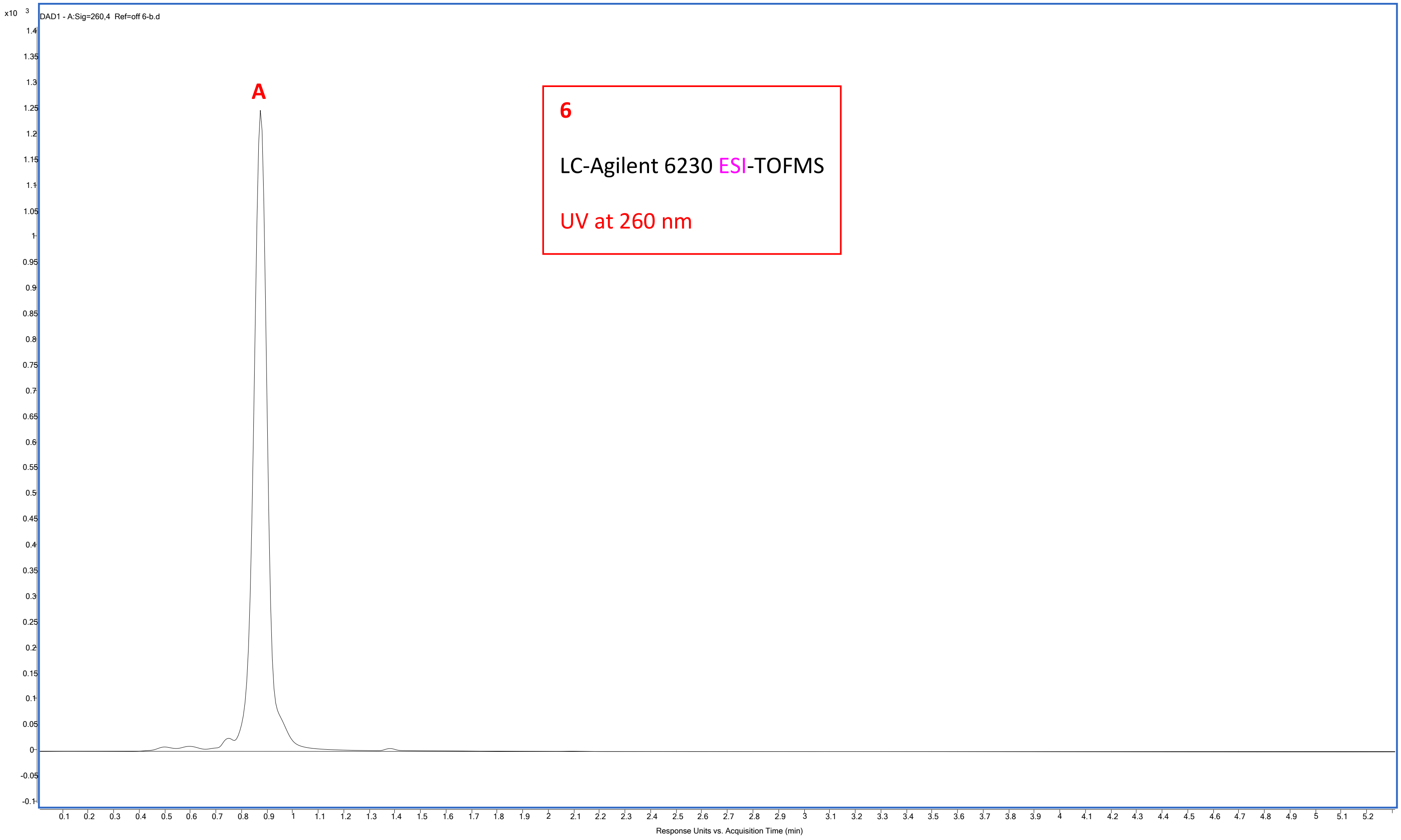

x10 3
DAD1 - A:Sig=260,4 Ref=off 6-b.d
A
6
LC-Agilent 6230 ESI-TOFMS
UV at 260 nm
Response Units vs. Acquisition Time (min)

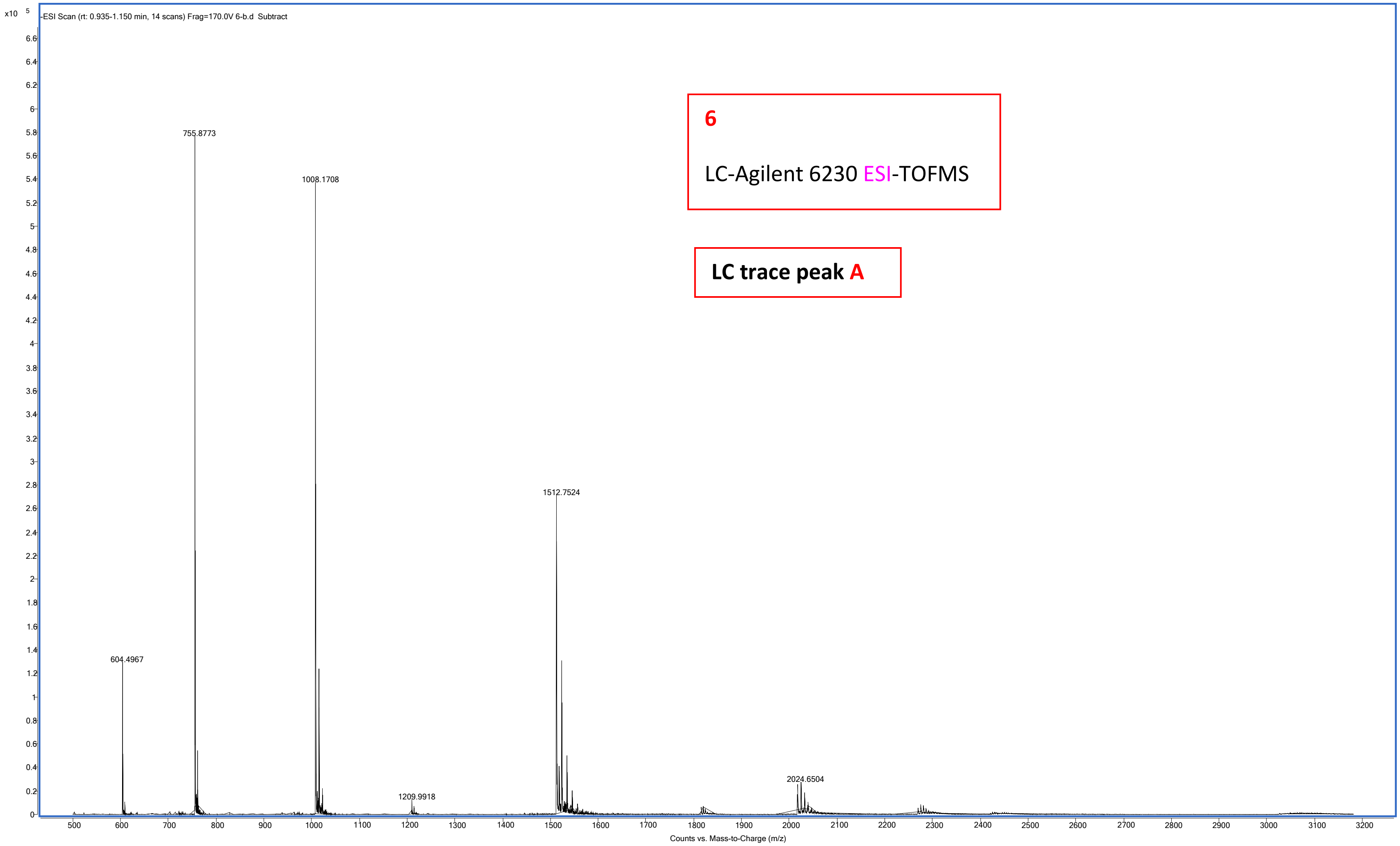

x10 5
-ESI Scan (rt: 0.935-1.150 min, 14 scans) Frag=170.0V 6-b.d Subtract
755.8773
1008.1708
1512.7524
604.4967
1209.9918
2024.6504
Counts vs. Mass-to-Charge (m/z)
6
LC-Agilent 6230 ESI-TOFMS
LC trace peak A

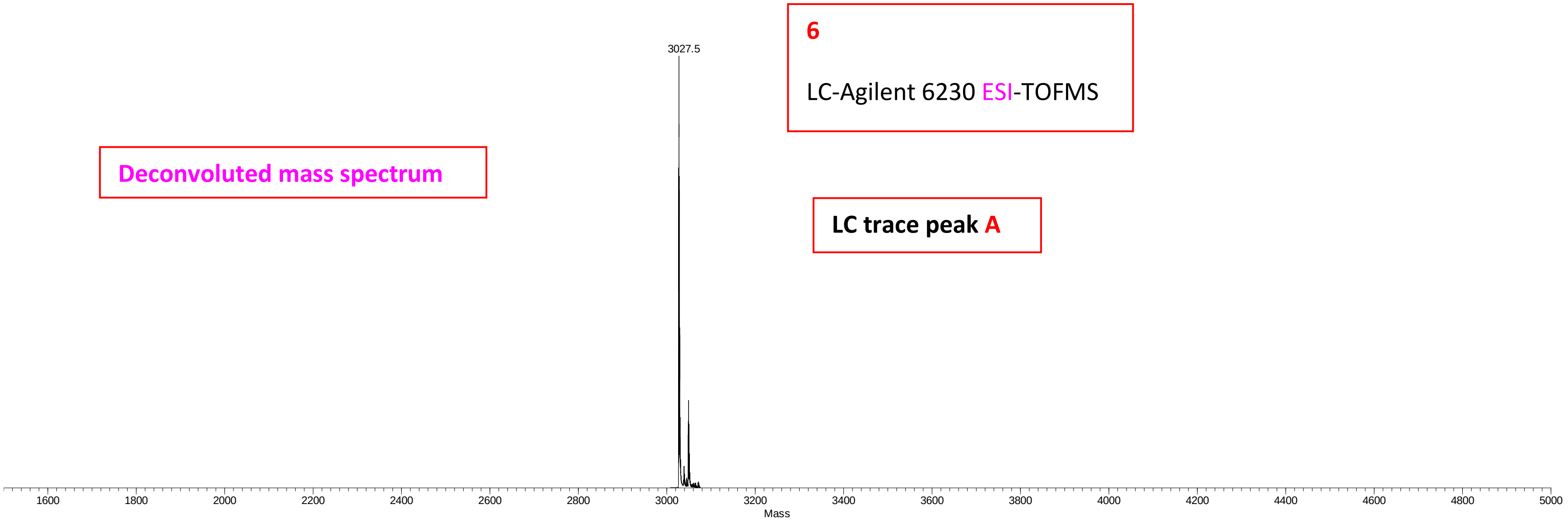

Deconvoluted mass spectrum
3027.5
6
LC-Agilent 6230 ESI-TOFMS
LC trace peak A
1600 1800 2000 2200 2400 2600 2800 3000 3200 3400 3600 3800 4000 4200 4400 4600 4800 5000
Mass

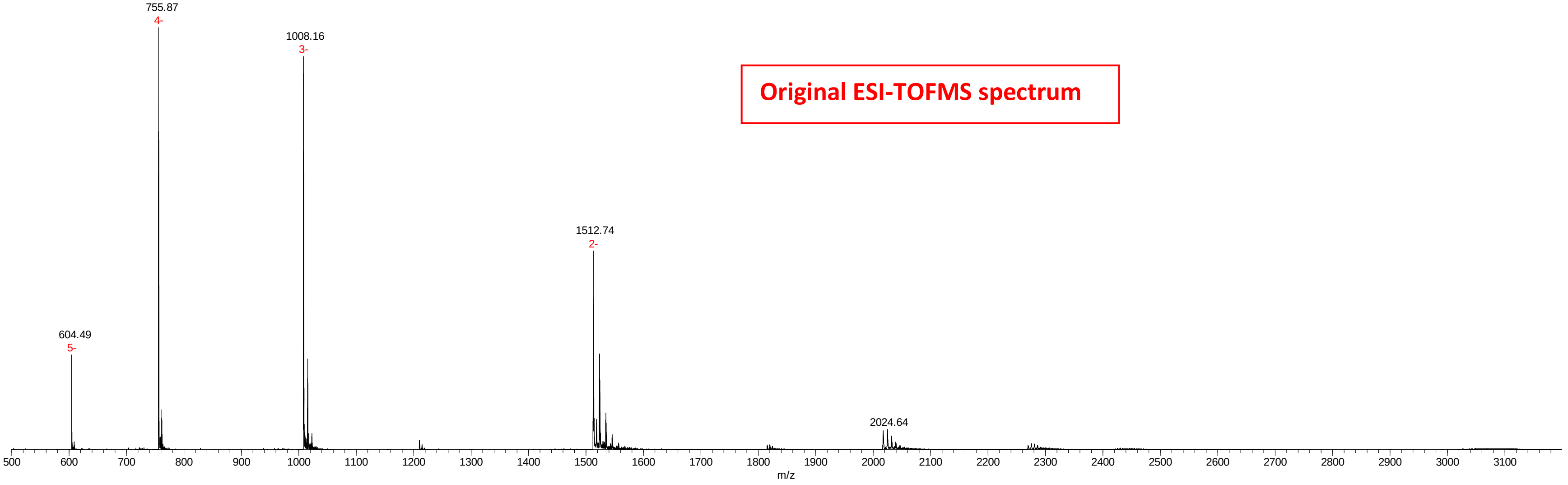

Original ESI-TOFMS spectrum
604.49
5-
755.87
4-
1008.16
3-
1512.74
2-
2024.64
500 600 700 800 900 1000 1100 1200 1300 1400 1500 1600 1700 1800 1900 2000 2100 2200 2300 2400 2500 2600 2700 2800 2900 3000 3100
m/z

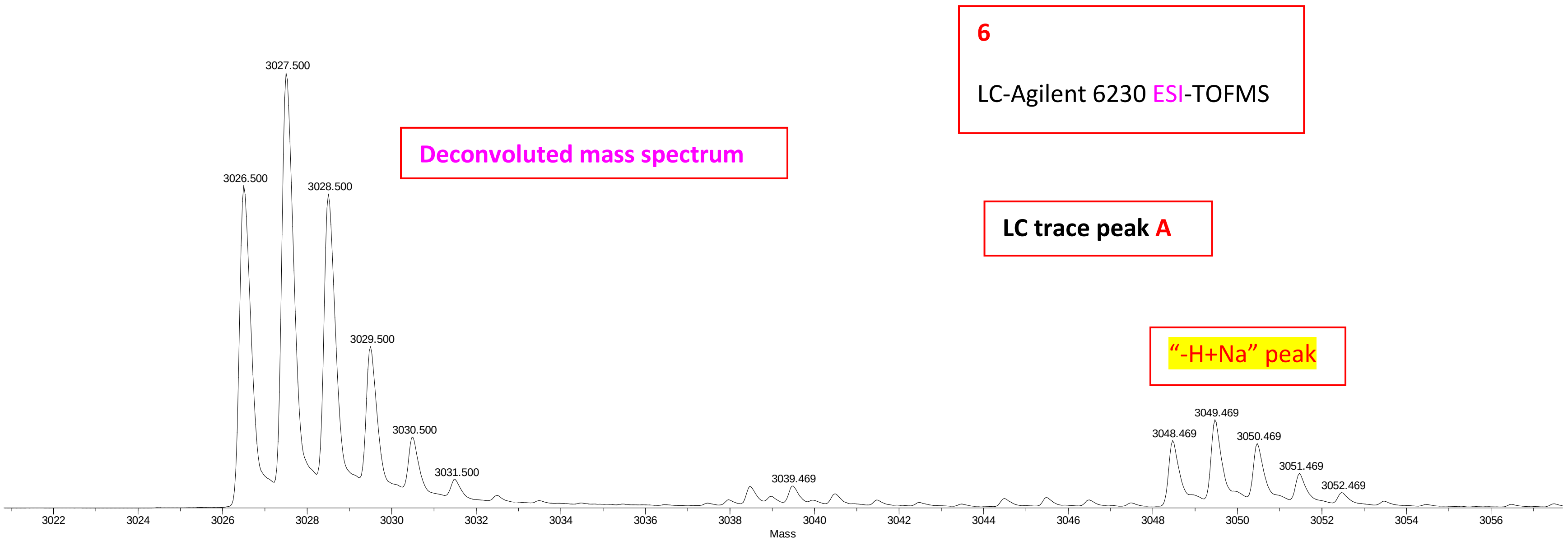

6
LC-Agilent 6230 ESI-TOFMS
Deconvoluted mass spectrum
LC trace peak A
"-H+Na" peak
3027.500
3026.500
3028.500
3029.500
3030.500
3031.500
3039.469
3048.469
3049.469
3050.469
3051.469
3052.469
3022
3024
3026
3028
3030
3032
3034
3036
3038
3040
3042
3044
3046
3048
3050
3052
3054
3056
Mass

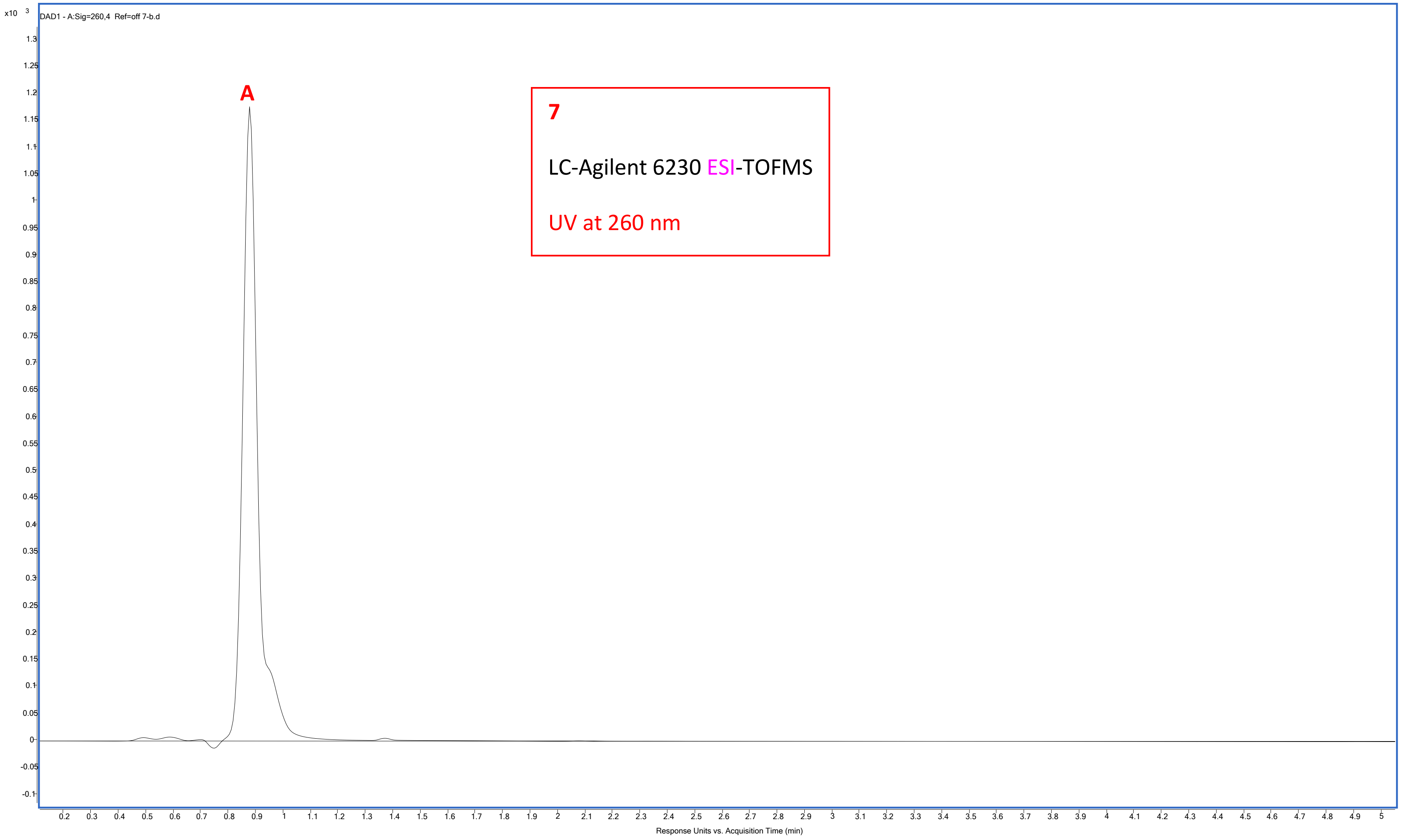

x10 3
DAD1 - A:Sig=260,4 Ref=off 7-b.d
A
7
LC-Agilent 6230 ESI-TOFMS
UV at 260 nm
Response Units vs. Acquisition Time (min)

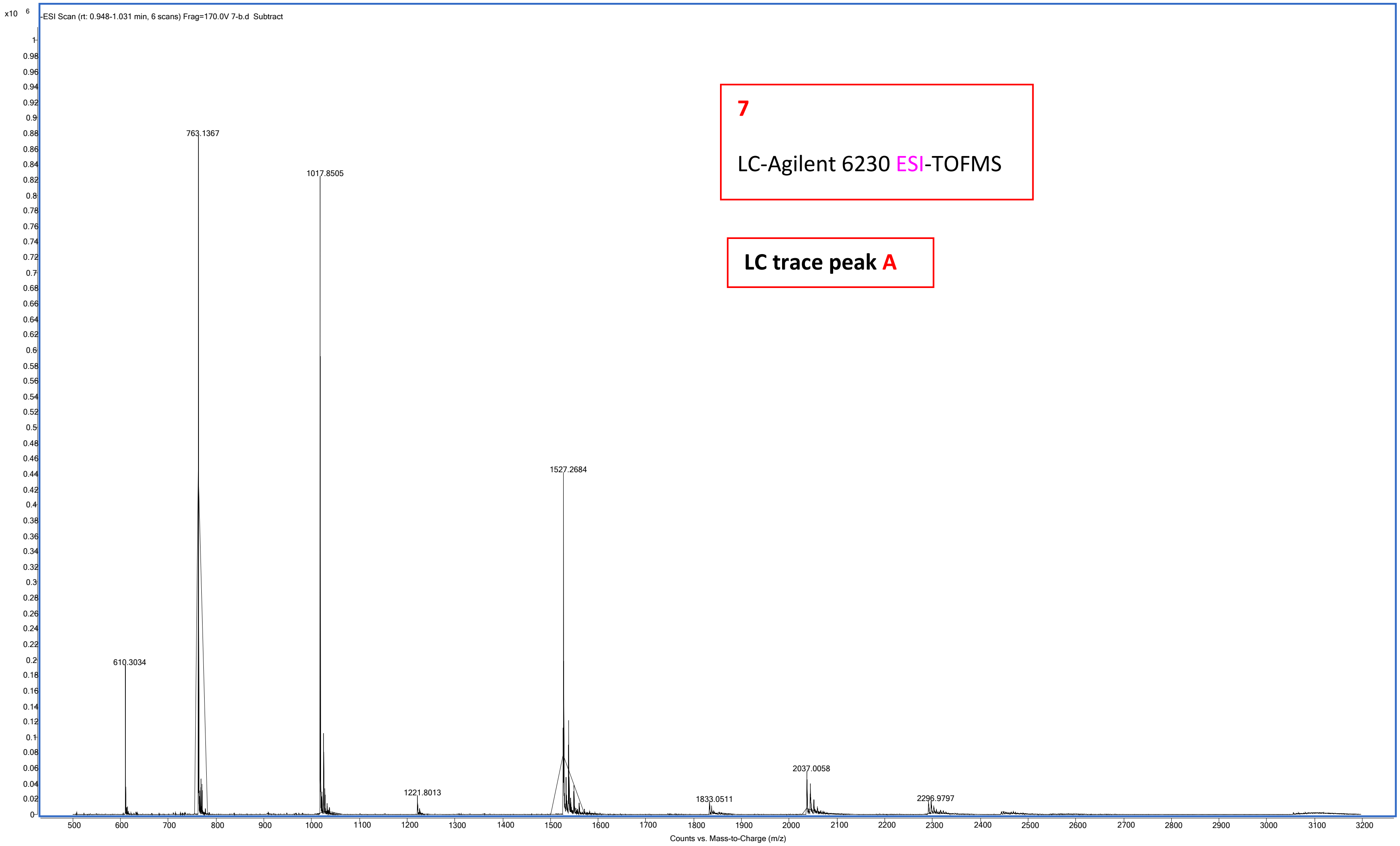

x10 6
-ESI Scan (rt: 0.948-1.031 min, 6 scans) Frag=170.0V 7-b.d Subtract
7
LC-Agilent 6230 ESI-TOFMS
LC trace peak A
763.1367
1017.8505
1527.2684
610.3034
1221.8013
1833.0511
2037.0058
2296.9797
Counts vs. Mass-to-Charge (m/z)

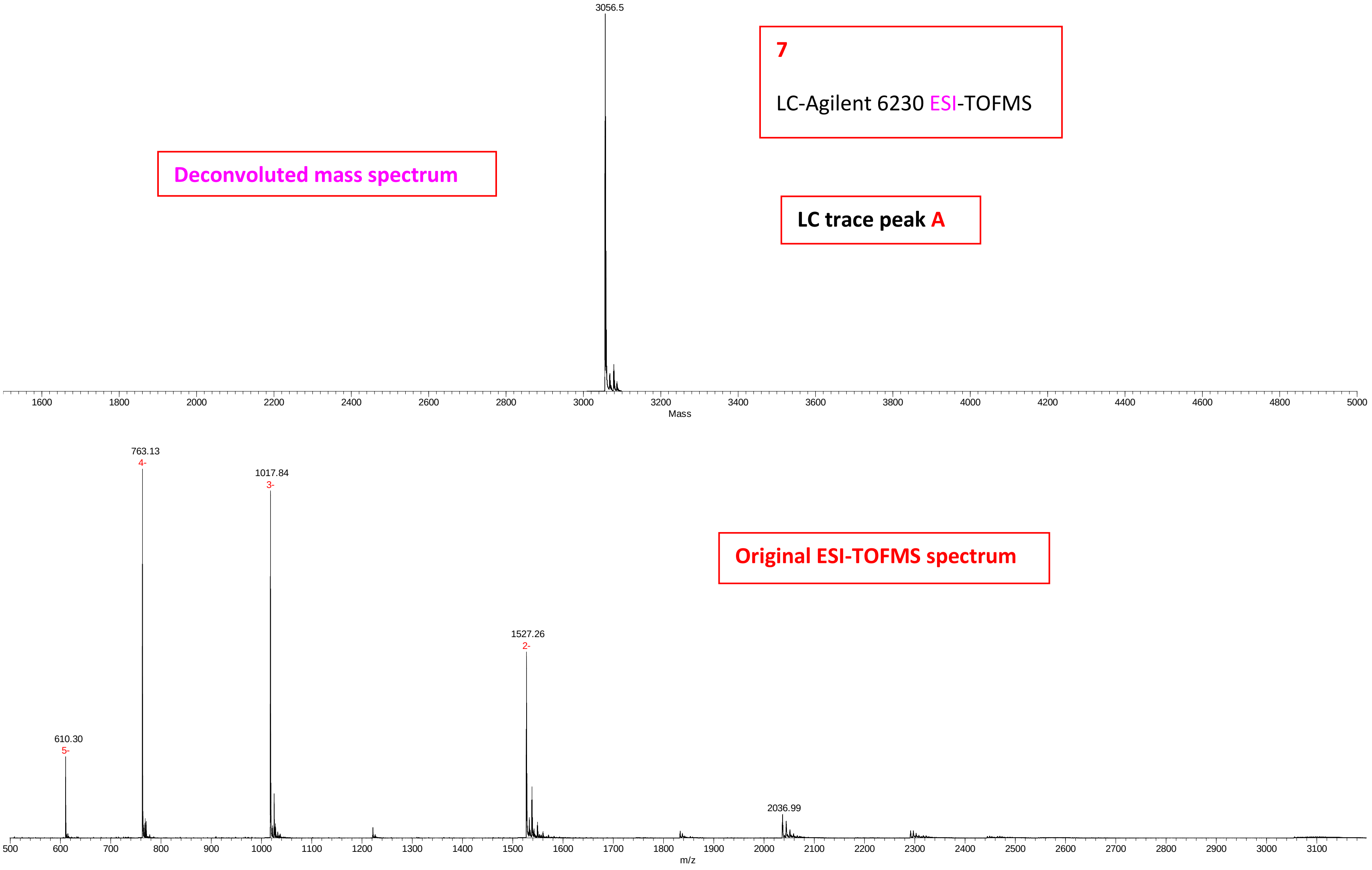
7
LC-Agilent 6230 ESI-TOFMS
Deconvoluted mass spectrum
LC trace peak A
3056.5
Mass
Original ESI-TOFMS spectrum
610.30
5-
763.13
4-
1017.84
3-
1527.26
2-
2036.99
m/z

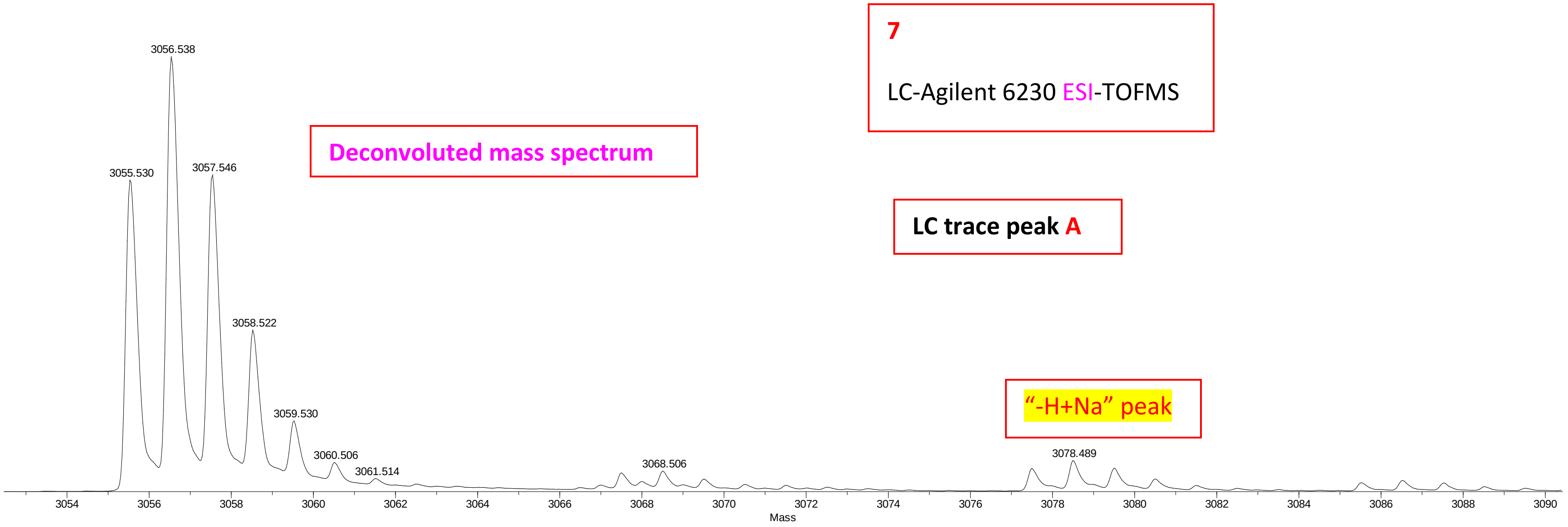
7
LC-Agilent 6230 ESI-TOFMS
Deconvoluted mass spectrum
LC trace peak A
"-H+Na" peak
3056.538
3055.530
3057.546
3058.522
3059.530
3060.506
3061.514
3068.506
3078.489
3054
3056
3058
3060
3062
3064
3066
3068
3070
3072
3074
3076
3078
3080
3082
3084
3086
3088
3090
Mass

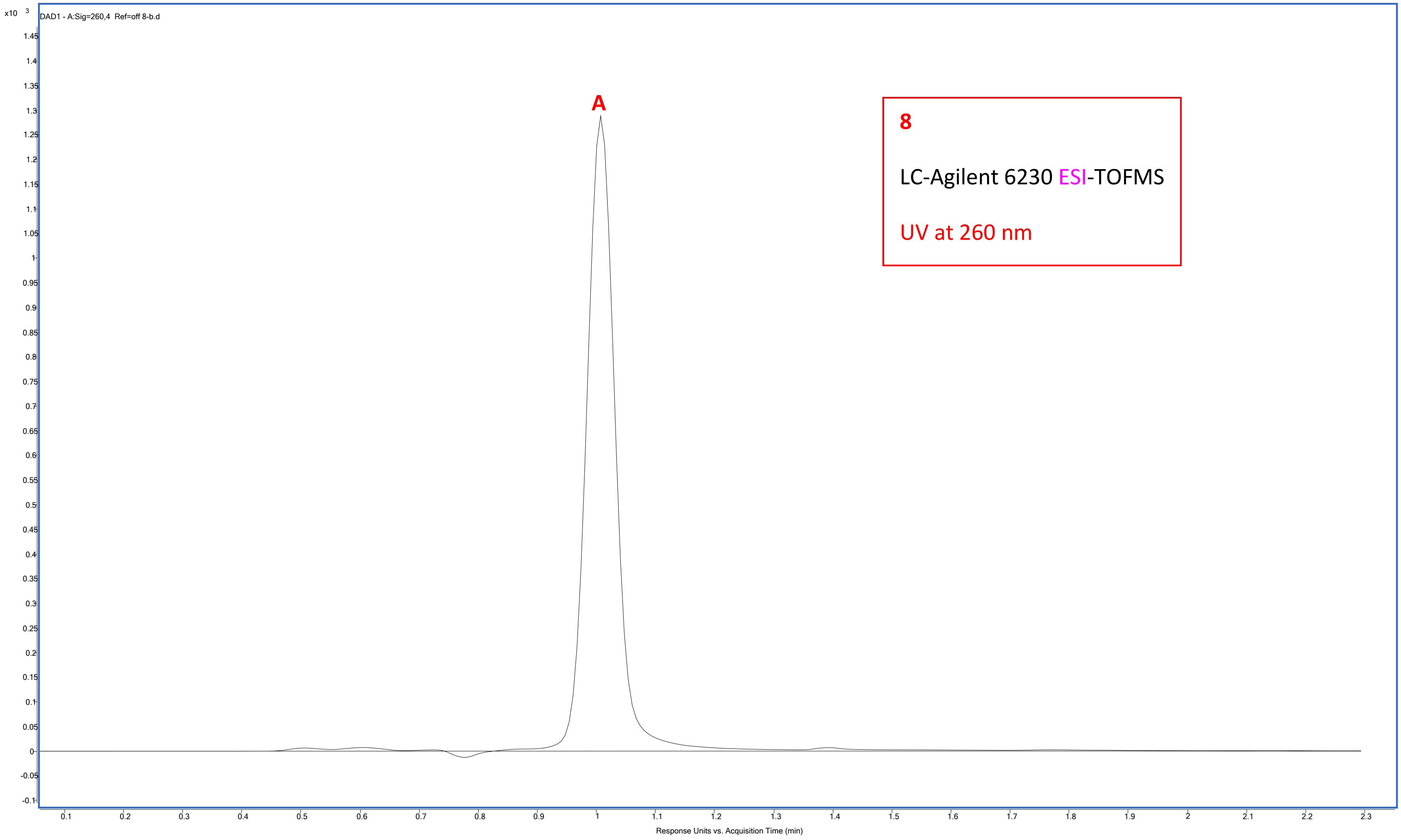
x10 3
DAD1 - A:Sig=260,4 Ref=off 8-b.d
A
8
LC-Agilent 6230 ESI-TOFMS
UV at 260 nm
Response Units vs. Acquisition Time (min)

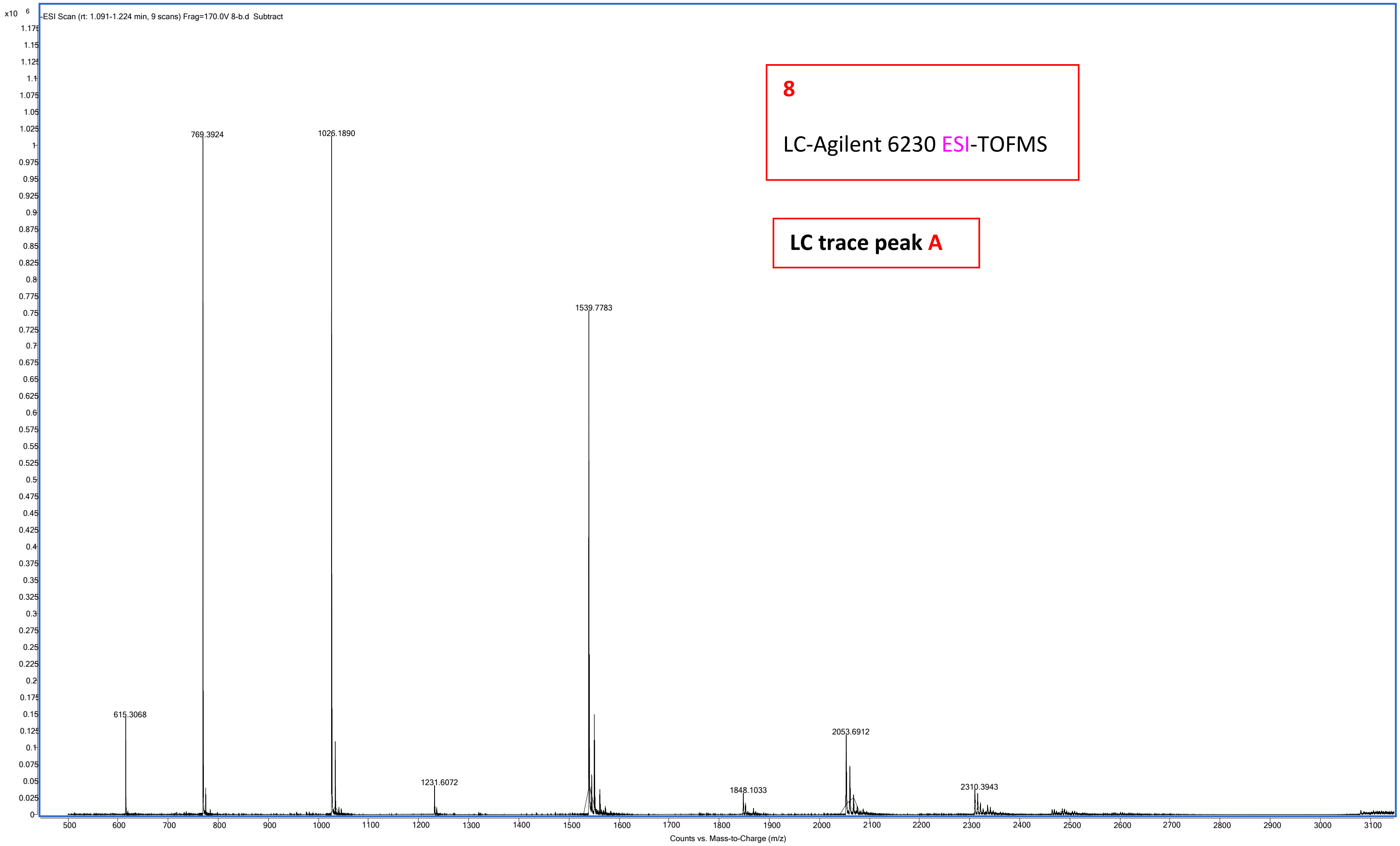

x10 6
-ESI Scan (rt: 1.091-1.224 min, 9 scans) Frag=170.0V 8-b.d Subtract
8
LC-Agilent 6230 ESI-TOFMS
LC trace peak A
769.3924
1026.1890
1539.7783
615.3068
1231.6072
1848.1033
2053.6912
2310.3943
Counts vs. Mass-to-Charge (m/z)

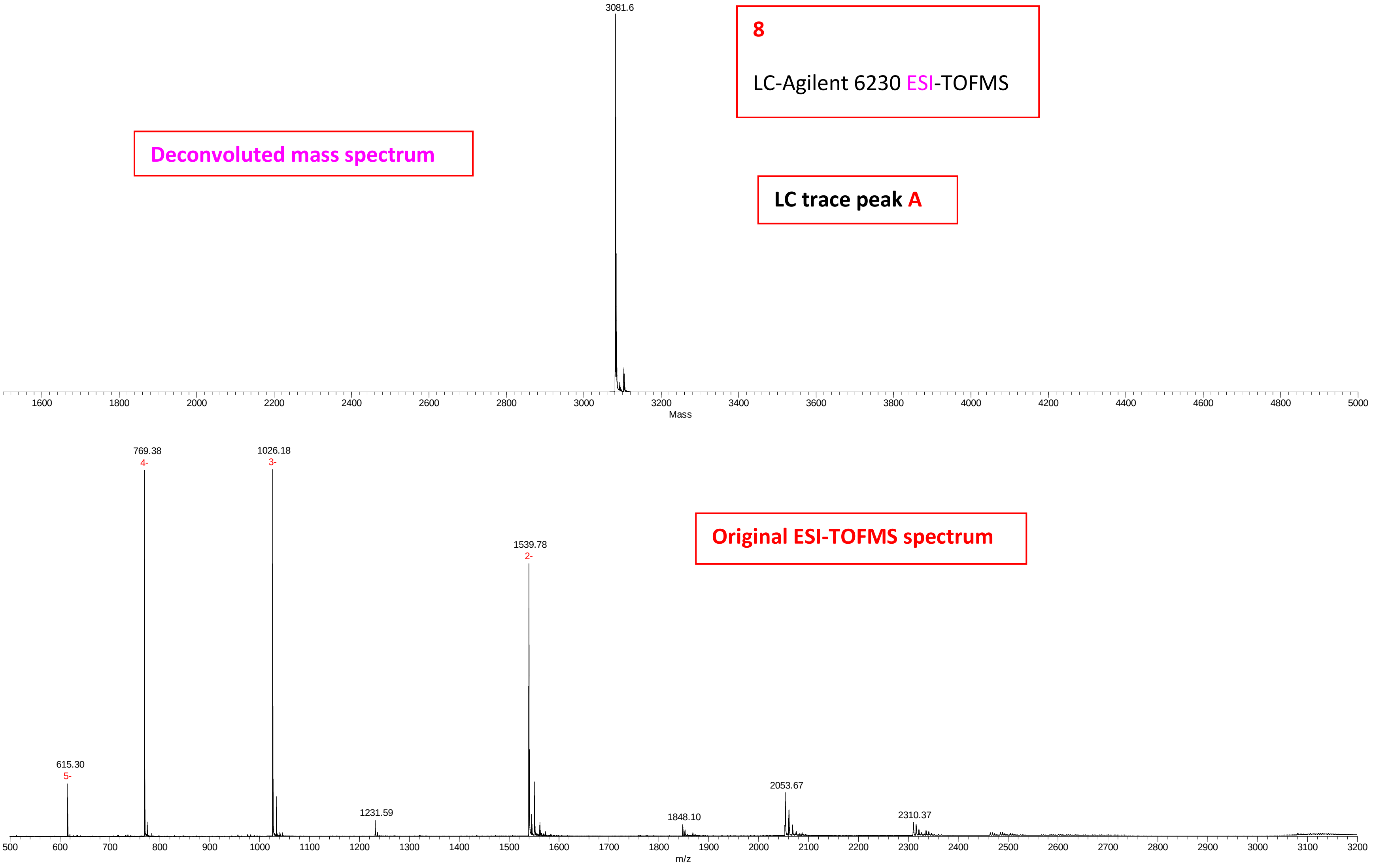
8
LC-Agilent 6230 ESI-TOFMS
Deconvoluted mass spectrum
LC trace peak A
3081.6
Mass
Original ESI-TOFMS spectrum
615.30
5-
769.38
4-
1026.18
3-
1231.59
1539.78
2-
1848.10
2053.67
2310.37
m/z

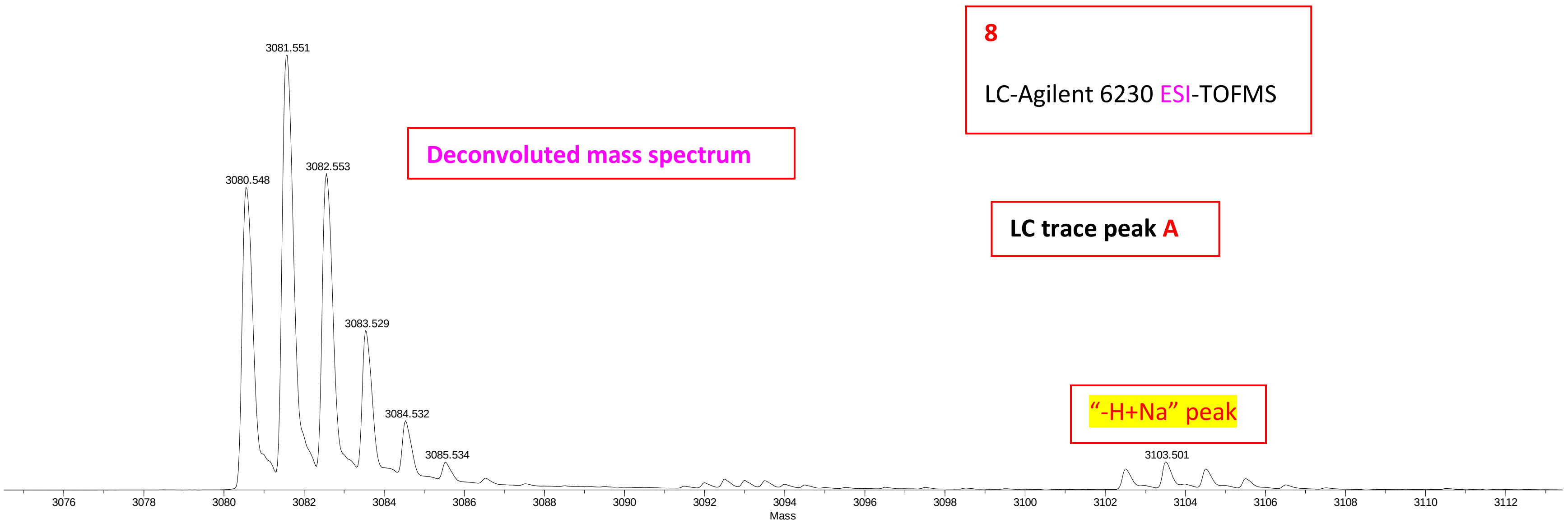
8
LC-Agilent 6230 ESI-TOFMS
Deconvoluted mass spectrum
LC trace peak A
“-H+Na” peak
3081.551
3080.548
3082.553
3083.529
3084.532
3085.534
3103.501
3076
3078
3080
3082
3084
3086
3088
3090
3092
3094
3096
3098
3100
3102
3104
3106
3108
3110
3112
Mass

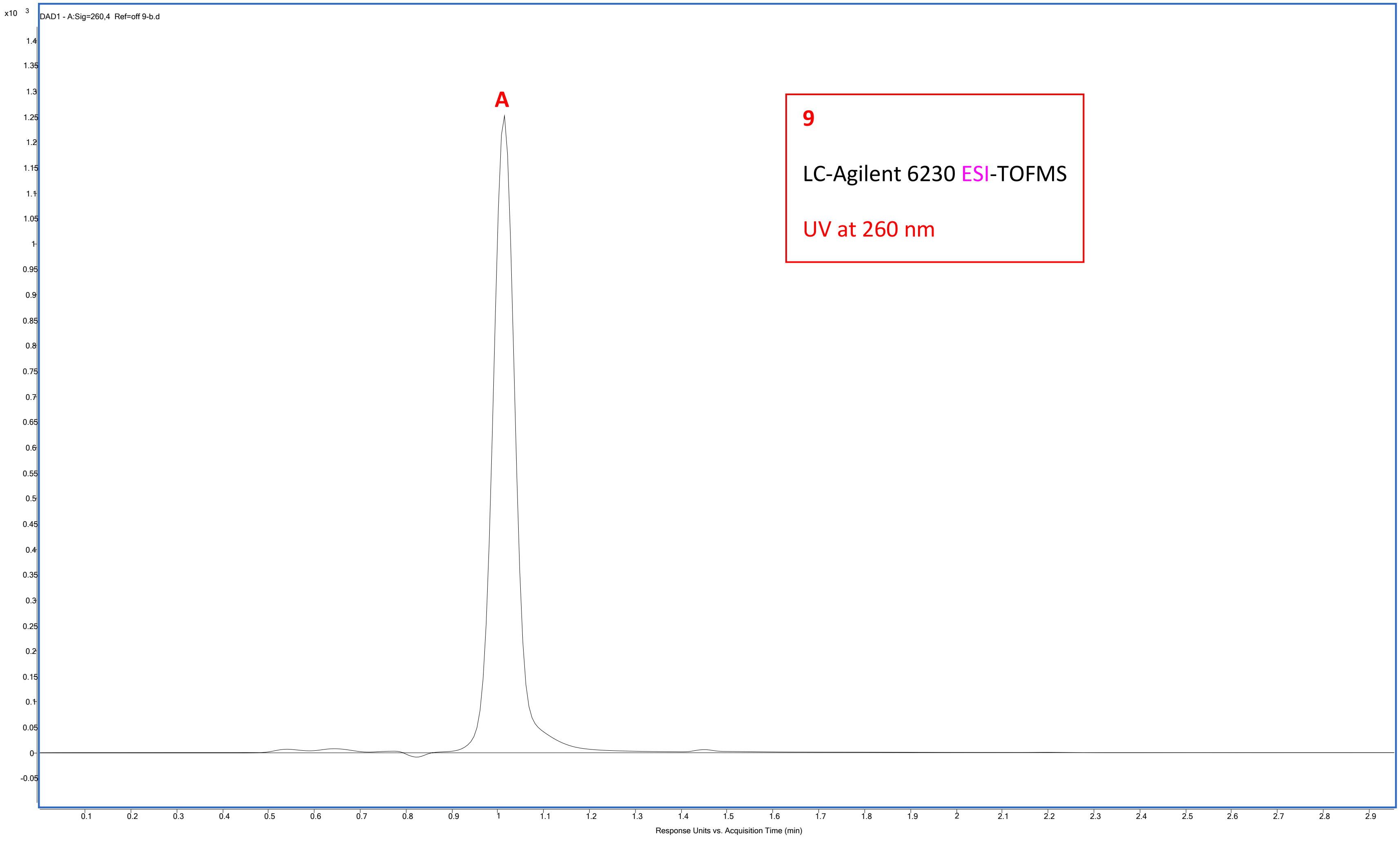

x10 3
DAD1 - A:Sig=260,4 Ref=off 9-b.d
A
9
LC-Agilent 6230 ESI-TOFMS
UV at 260 nm
Response Units vs. Acquisition Time (min)

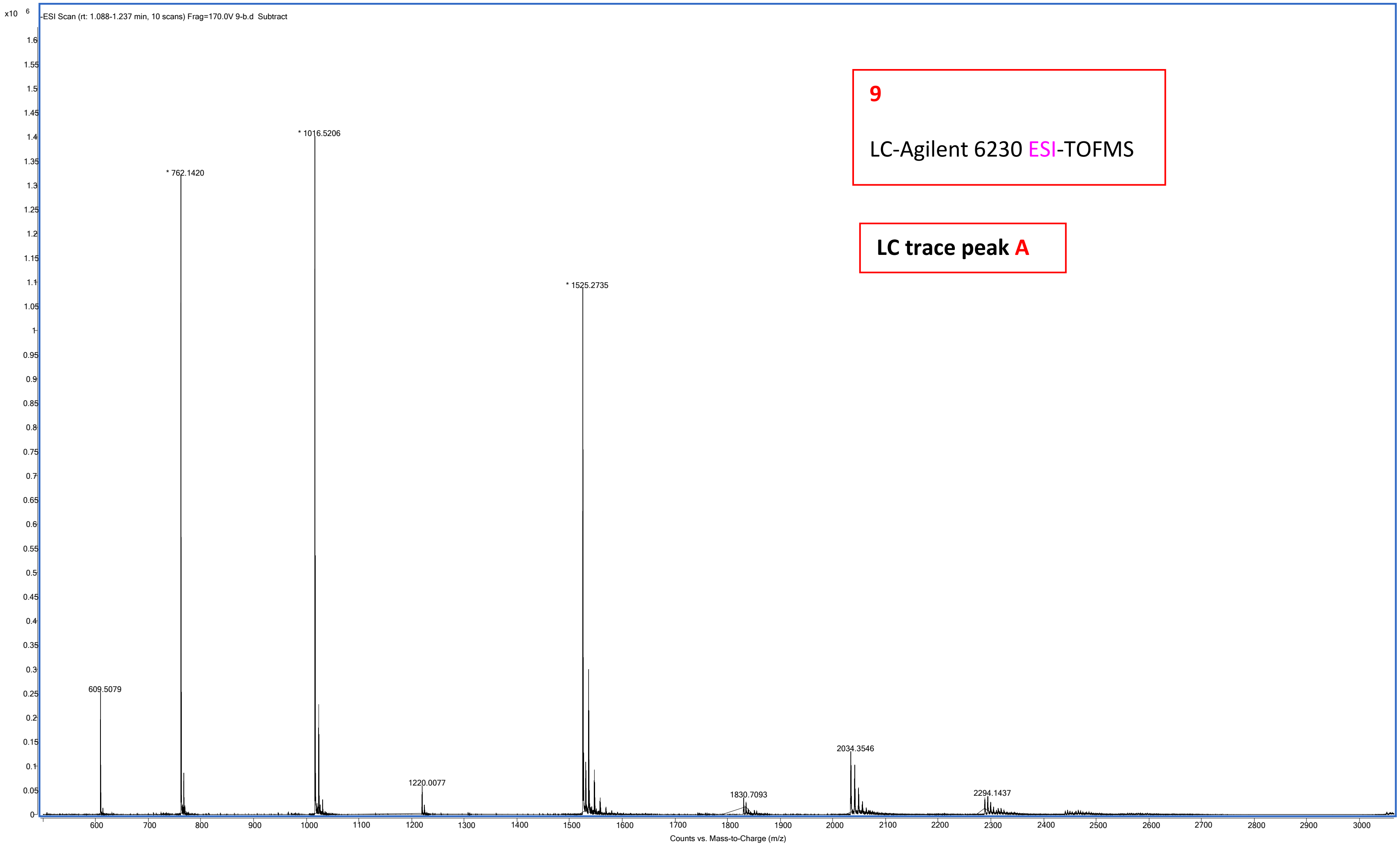
x10 6
-ESI Scan (rt: 1.088-1.237 min, 10 scans) Frag=170.0V 9-b.d Subtract
9
LC-Agilent 6230 ESI-TOFMS
LC trace peak A
* 1016.5206
* 762.1420
* 1525.2735
609.5079
1220.0077
1830.7093
2034.3546
2294.1437
Counts vs. Mass-to-Charge (m/z)

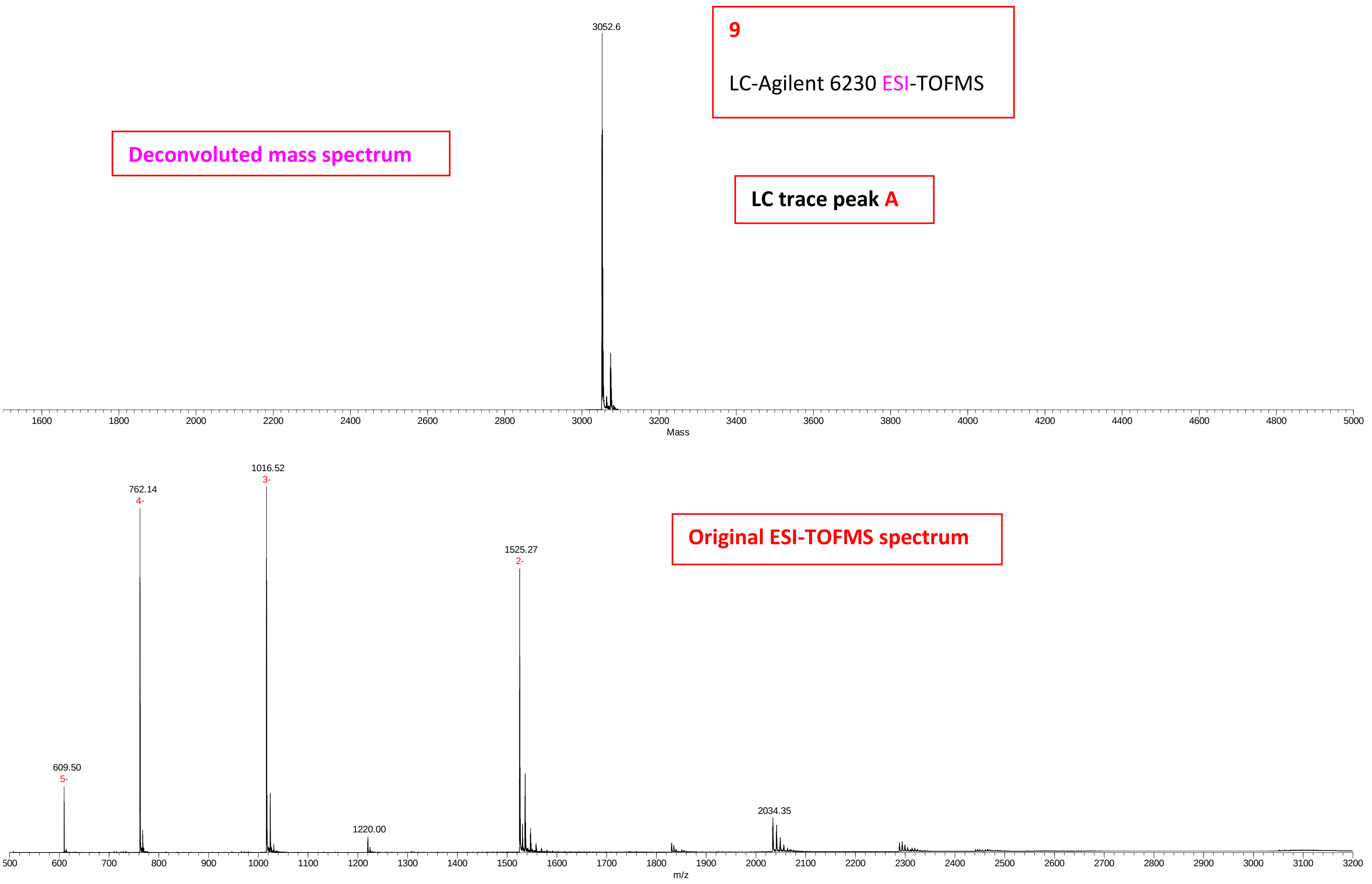
9
LC-Agilent 6230 ESI-TOFMS
Deconvoluted mass spectrum
LC trace peak A
3052.6
1600
1800
2000
2200
2400
2600
2800
3000
3200
3400
3600
3800
4000
4200
4400
4600
4800
5000
Mass
Original ESI-TOFMS spectrum
609.50
5-
762.14
4-
1016.52
3-
1220.00
1525.27
2-
2034.35
500
600
700
800
900
1000
1100
1200
1300
1400
1500
1600
1700
1800
1900
2000
2100
2200
2300
2400
2500
2600
2700
2800
2900
3000
3100
3200
m/z

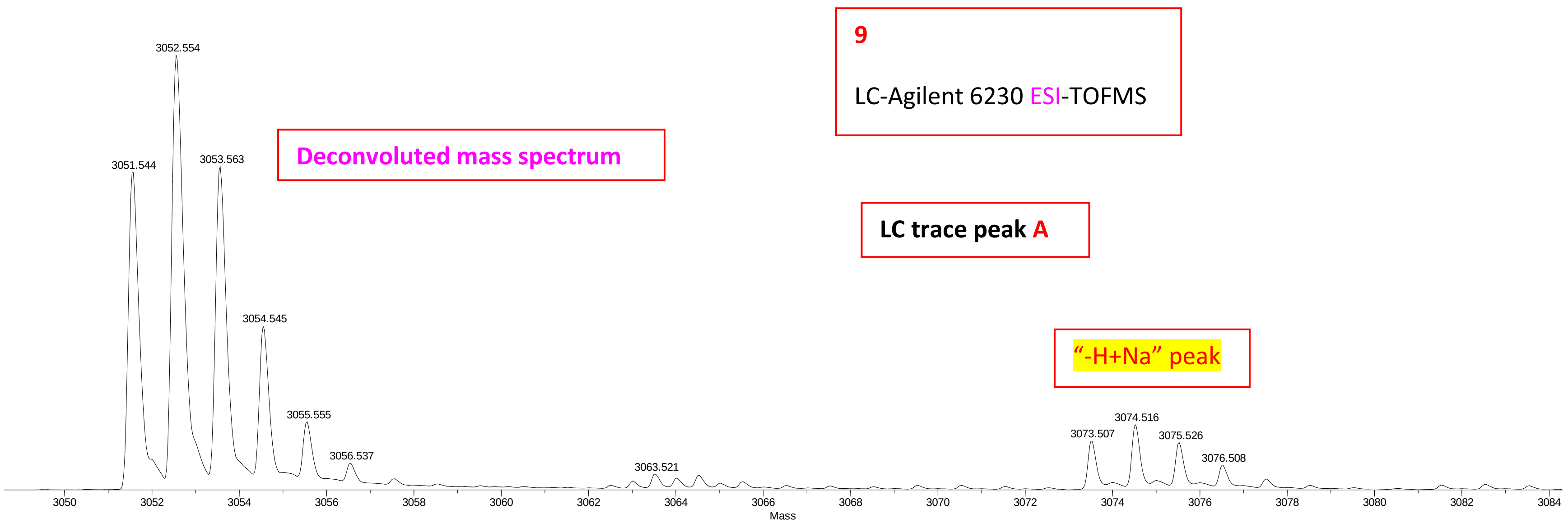

9
LC-Agilent 6230 ESI-TOFMS
Deconvoluted mass spectrum
LC trace peak A
"-H+Na" peak
3051.544
3052.554
3053.563
3054.545
3055.555
3056.537
3063.521
3073.507
3074.516
3075.526
3076.508
3050
3052
3054
3056
3058
3060
3062
3064
3066
3068
3070
3072
3074
3076
3078
3080
3082
3084
Mass

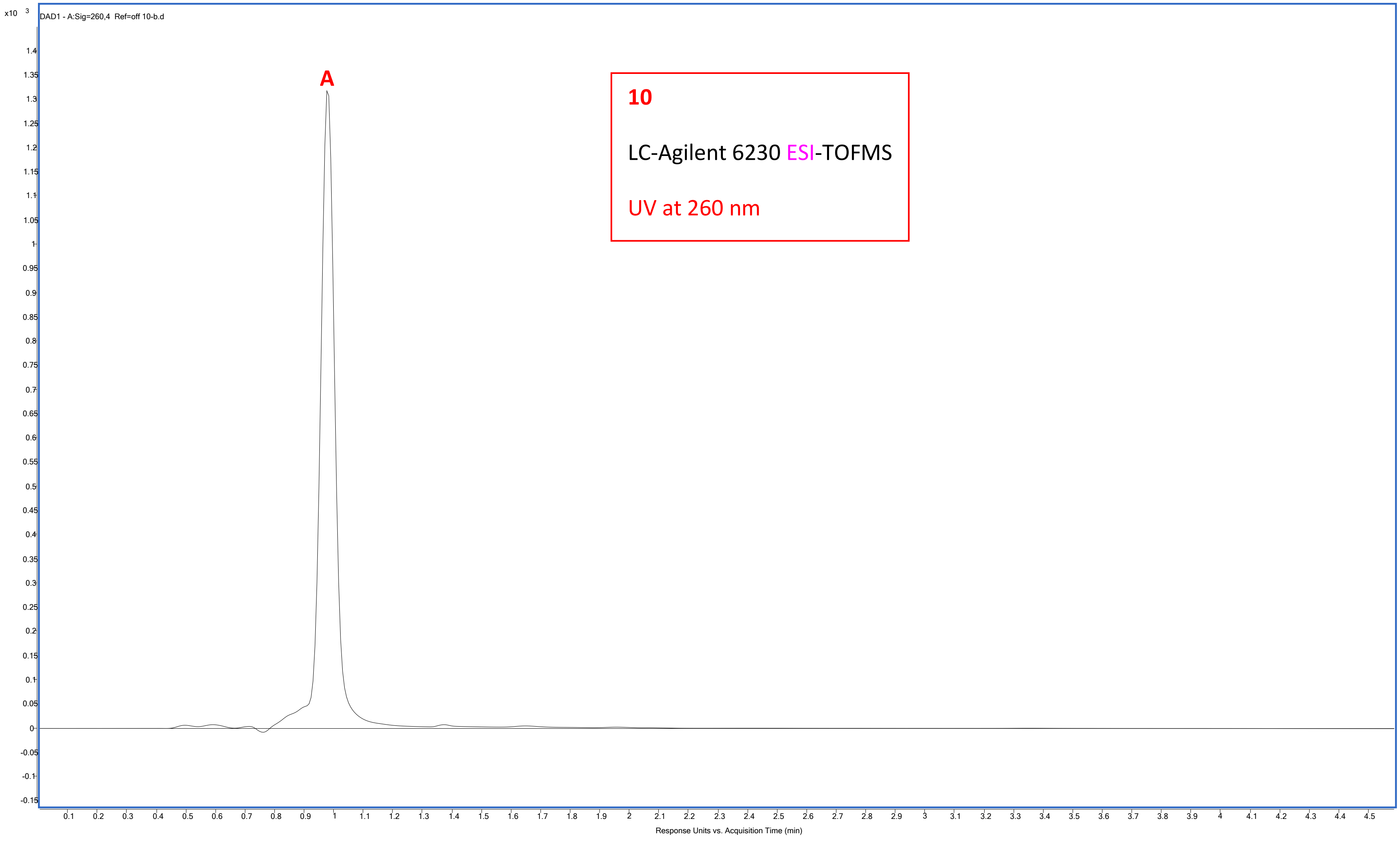

x10 3
DAD1 - A:Sig=260,4 Ref=off 10-b.d
A
10
LC-Agilent 6230 ESI-TOFMS
UV at 260 nm
Response Units vs. Acquisition Time (min)

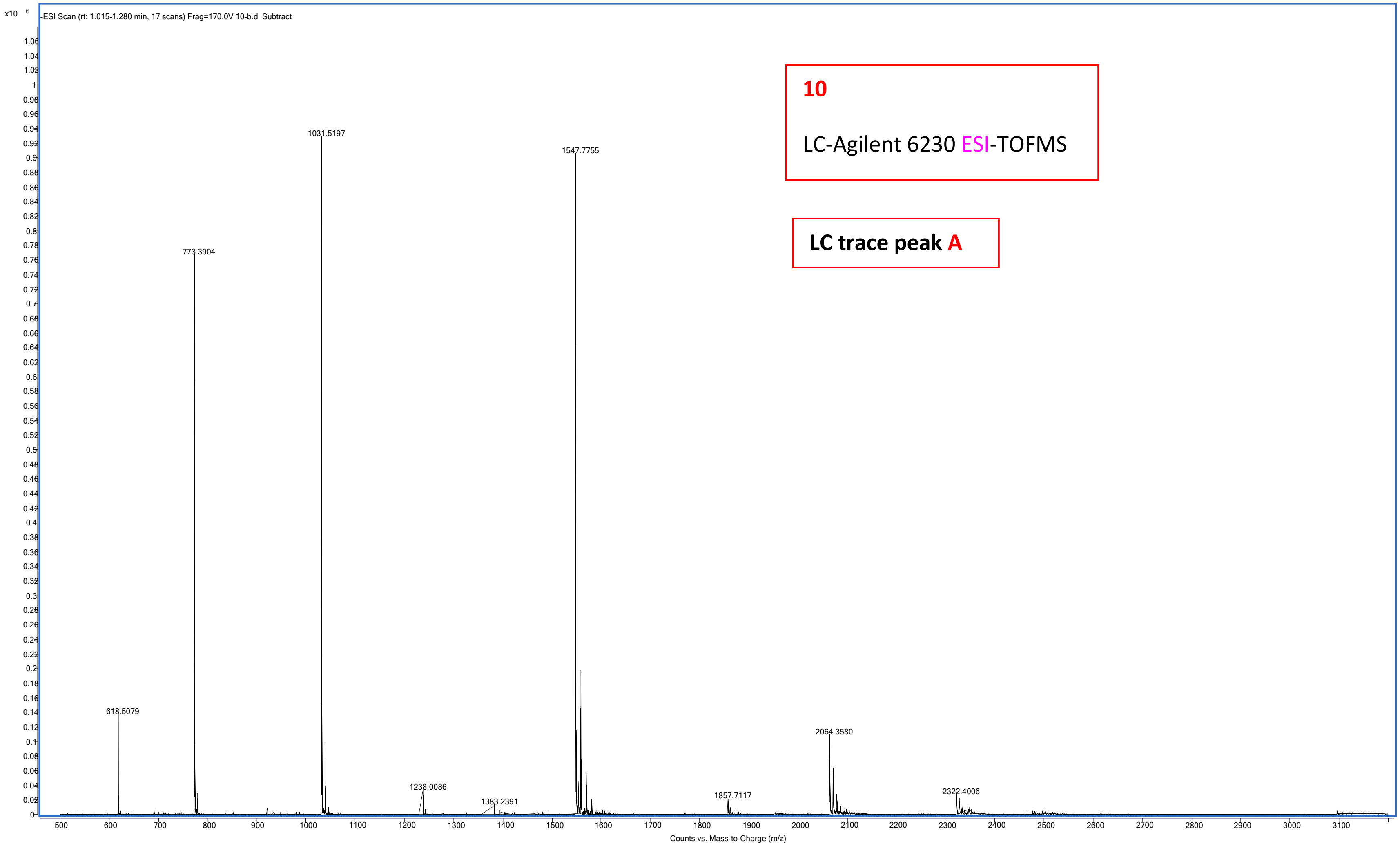

x10 6
-ESI Scan (rt: 1.015-1.280 min, 17 scans) Frag=170.0V 10-b.d Subtract
10
LC-Agilent 6230 ESI-TOFMS
LC trace peak A
618.5079
773.3904
1031.5197
1238.0086
1383.2391
1547.7755
1857.7117
2064.3580
2322.4006
Counts vs. Mass-to-Charge (m/z)

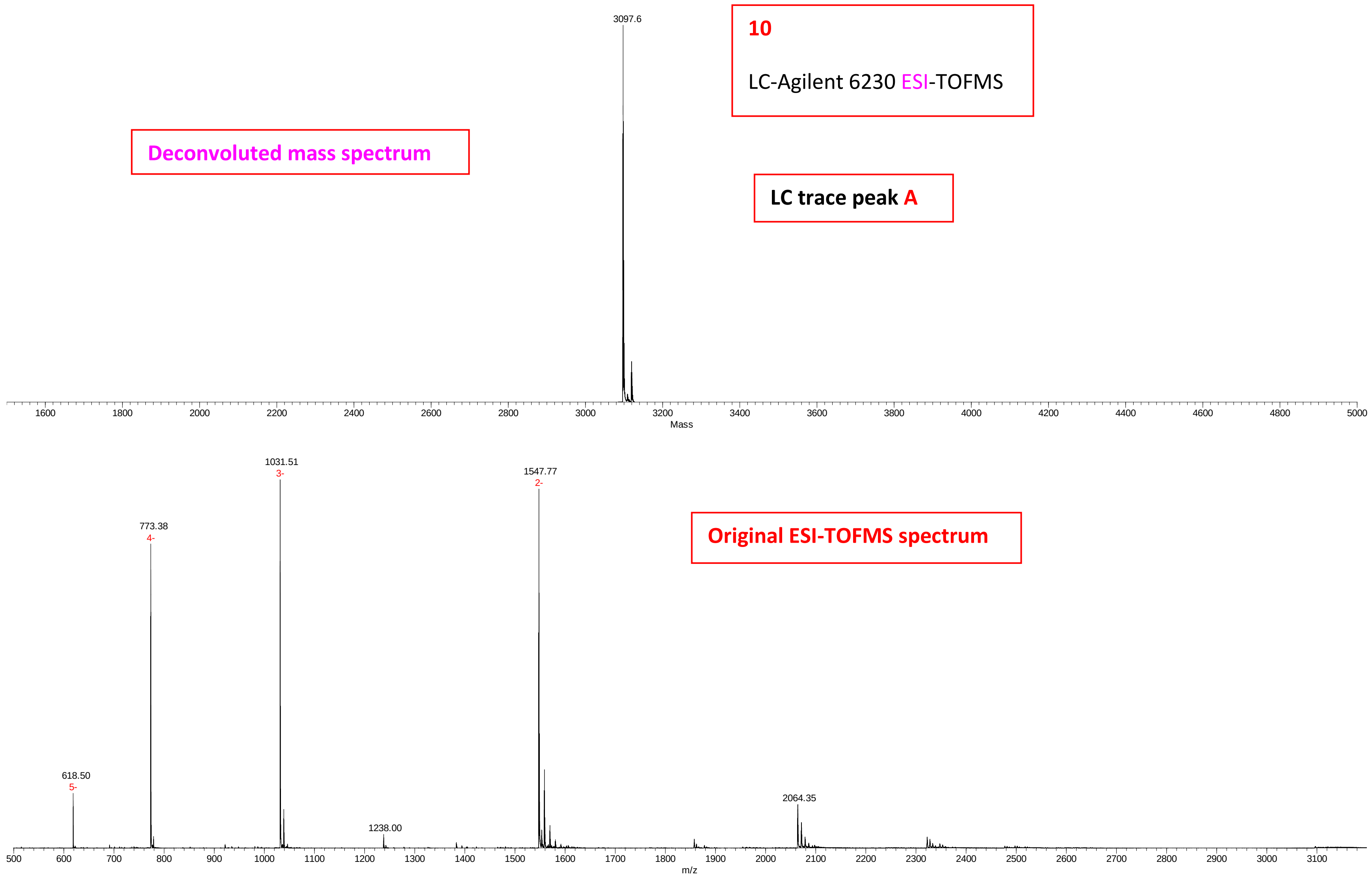
10
LC-Agilent 6230 ESI-TOFMS
Deconvoluted mass spectrum
LC trace peak A
3097.6
1600
1800
2000
2200
2400
2600
2800
3000
3200
3400
3600
3800
4000
4200
4400
4600
4800
5000
Mass
Original ESI-TOFMS spectrum
618.50
5-
773.38
4-
1031.51
3-
1238.00
1547.77
2-
2064.35
500
600
700
800
900
1000
1100
1200
1300
1400
1500
1600
1700
1800
1900
2000
2100
2200
2300
2400
2500
2600
2700
2800
2900
3000
3100
m/z

10

LC-Agilent 6230 ESI-TOFMS

Deconvoluted mass spectrum

LC trace peak A

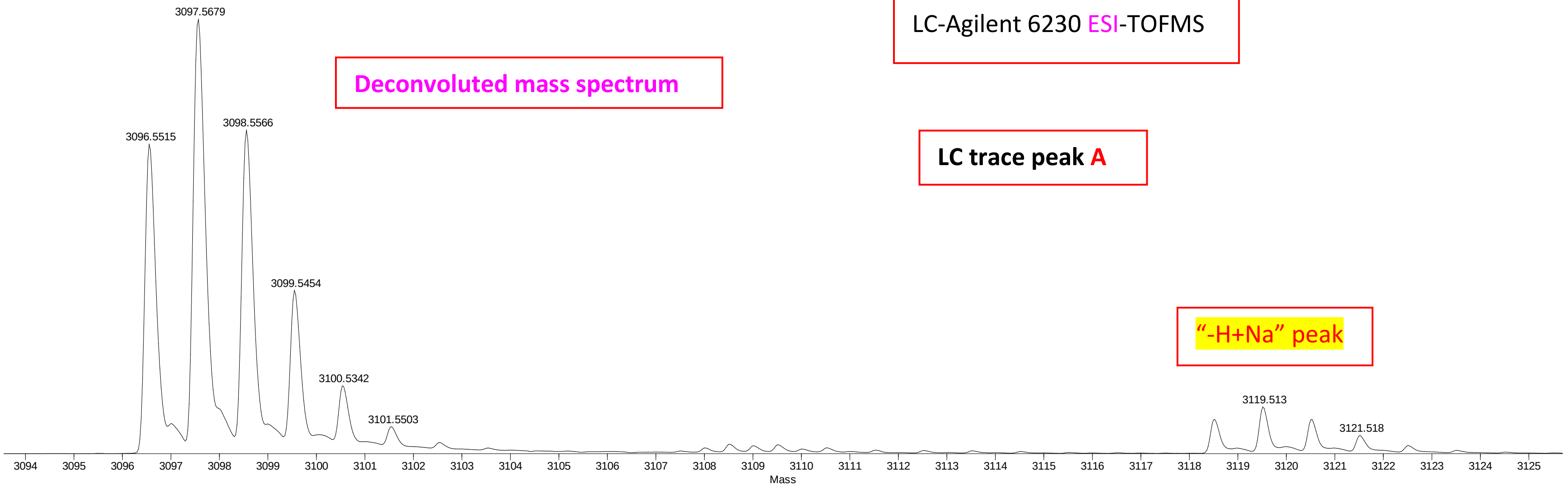

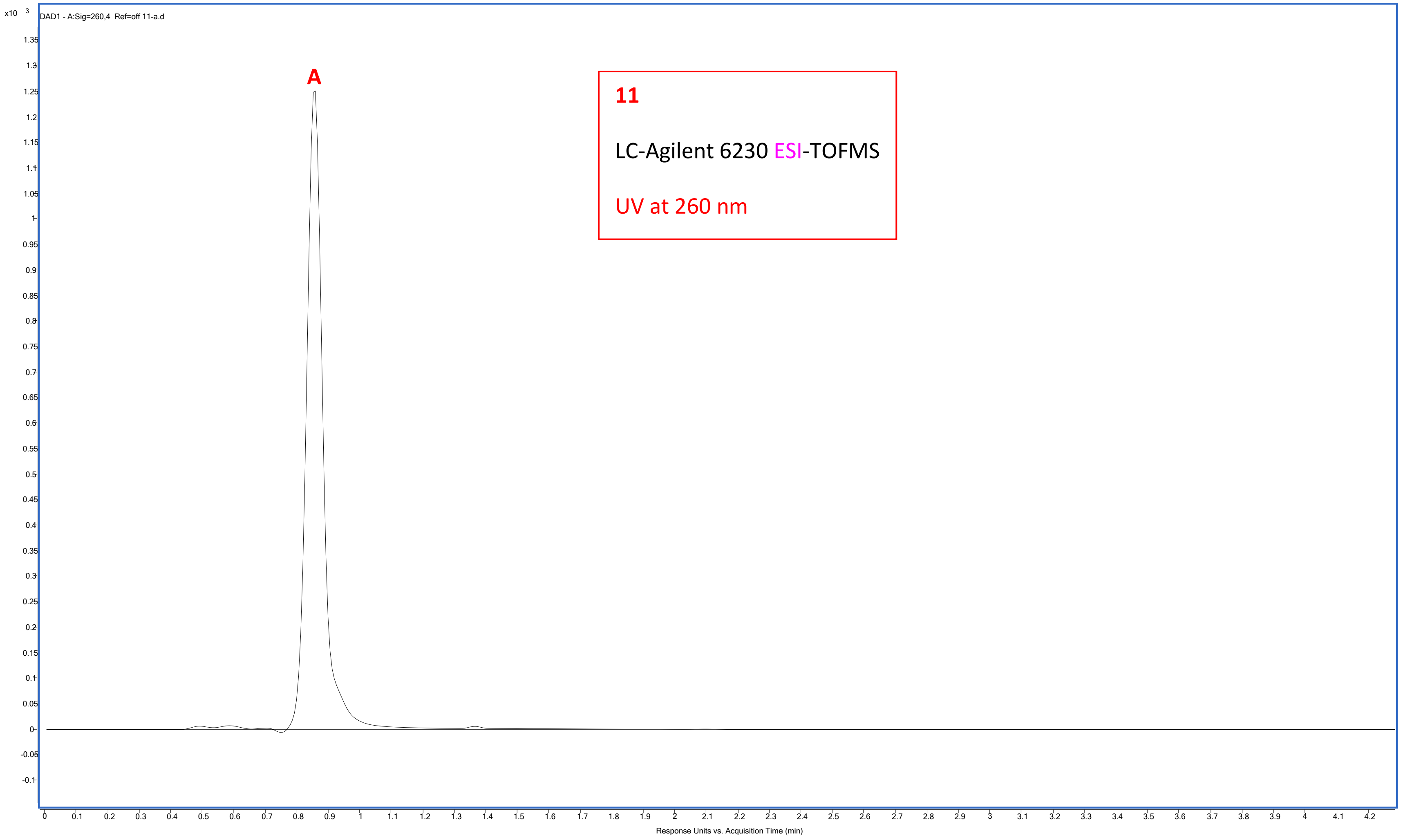

x10 3
DAD1 - A:Sig=260,4 Ref=off 11-a.d
A
11
LC-Agilent 6230 ESI-TOFMS
UV at 260 nm
Response Units vs. Acquisition Time (min)

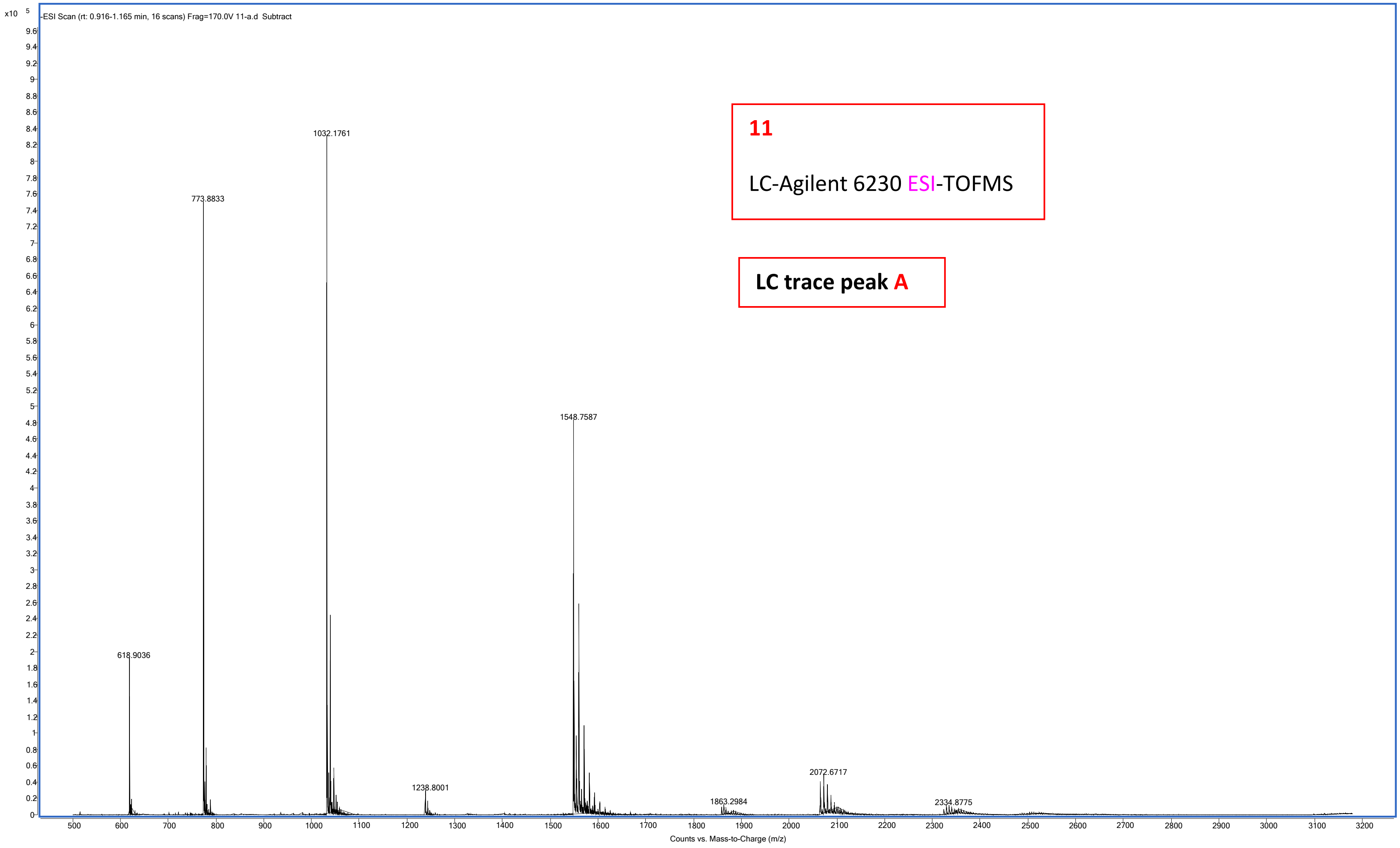
x10 5
-ESI Scan (rt: 0.916-1.165 min, 16 scans) Frag=170.0V 11-a.d Subtract
11
LC-Agilent 6230 ESI-TOFMS
LC trace peak A
1032.1761
773.8833
1548.7587
618.9036
1238.8001
1863.2984
2072.6717
2334.8775
Counts vs. Mass-to-Charge (m/z)

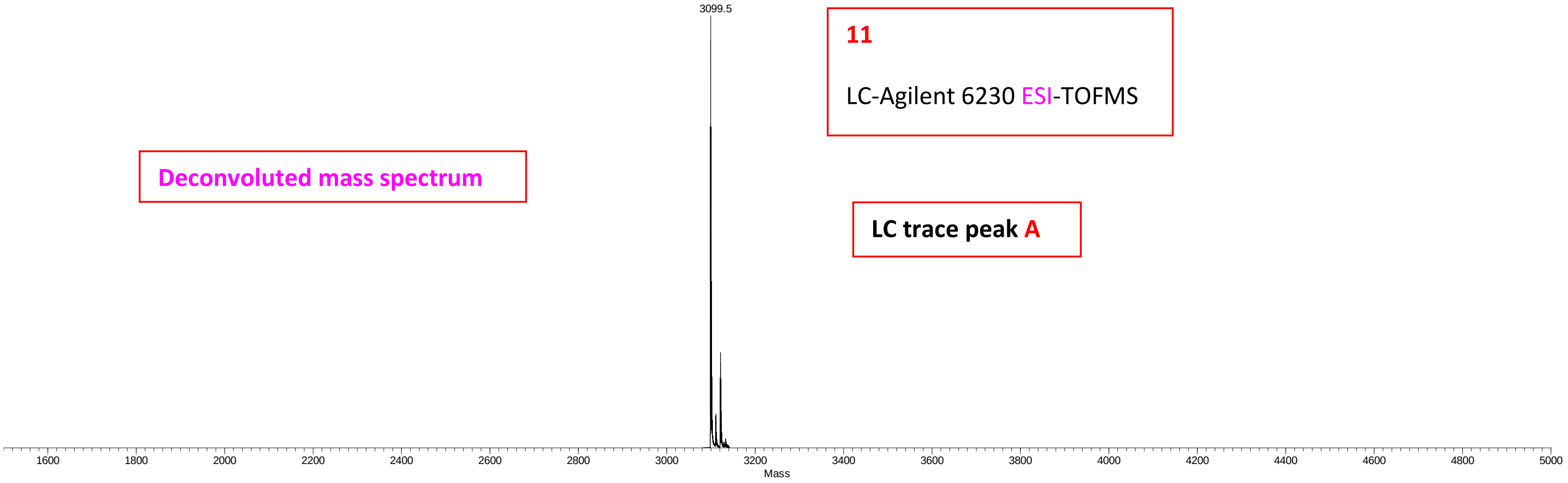

Deconvoluted mass spectrum

11

LC-Agilent 6230 ESI-TOFMS

LC trace peak A

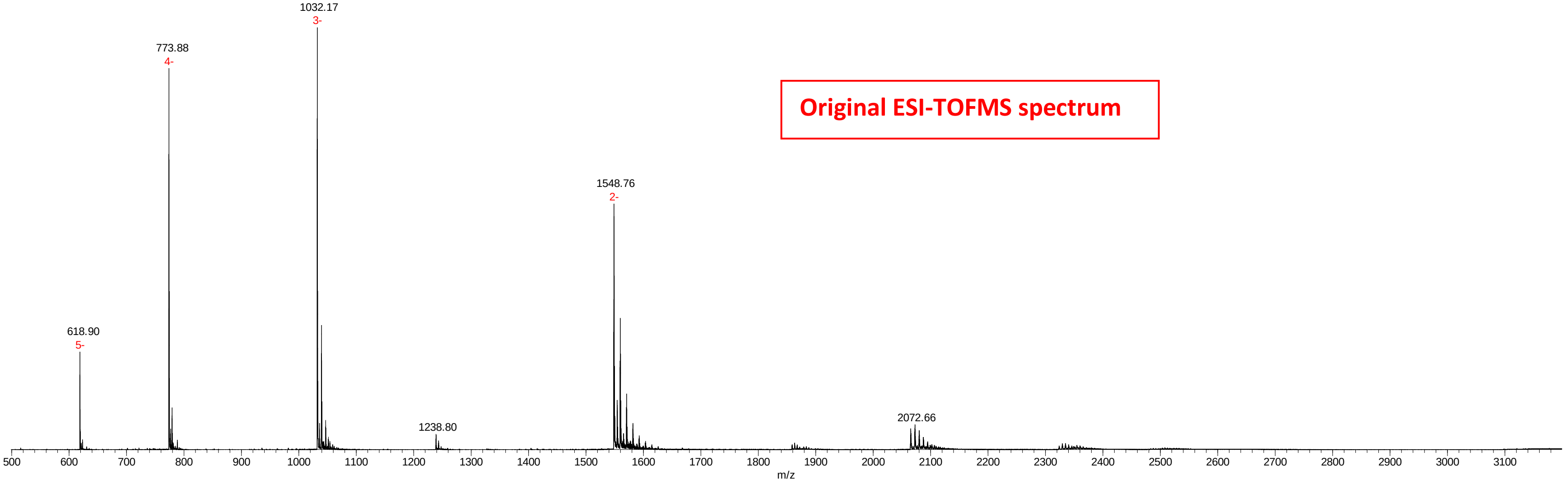

Original ESI-TOFMS spectrum

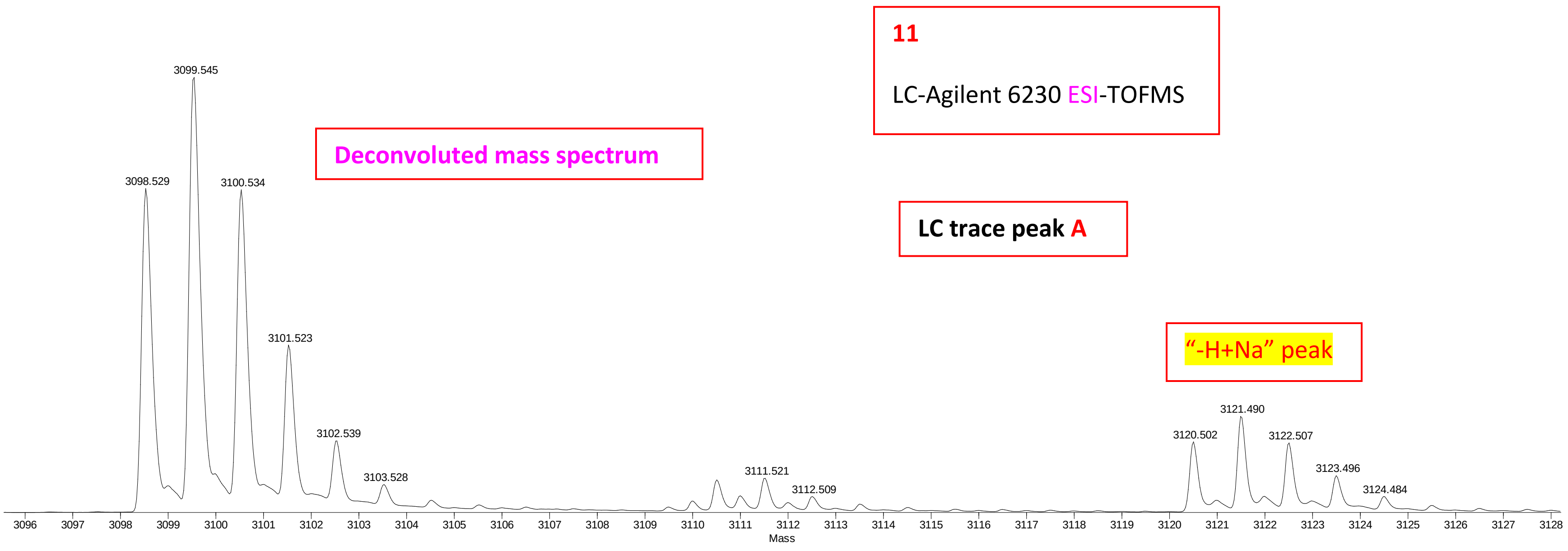
11
LC-Agilent 6230 ESI-TOFMS
Deconvoluted mass spectrum
LC trace peak A
“-H+Na” peak
3099.545
3098.529
3100.534
3101.523
3102.539
3103.528
3111.521
3112.509
3121.490
3120.502
3122.507
3123.496
3124.484
3096 3097 3098 3099 3100 3101 3102 3103 3104 3105 3106 3107 3108 3109 3110 3111 3112 3113 3114 3115 3116 3117 3118 3119 3120 3121 3122 3123 3124 3125 3126 3127 3128
Mass

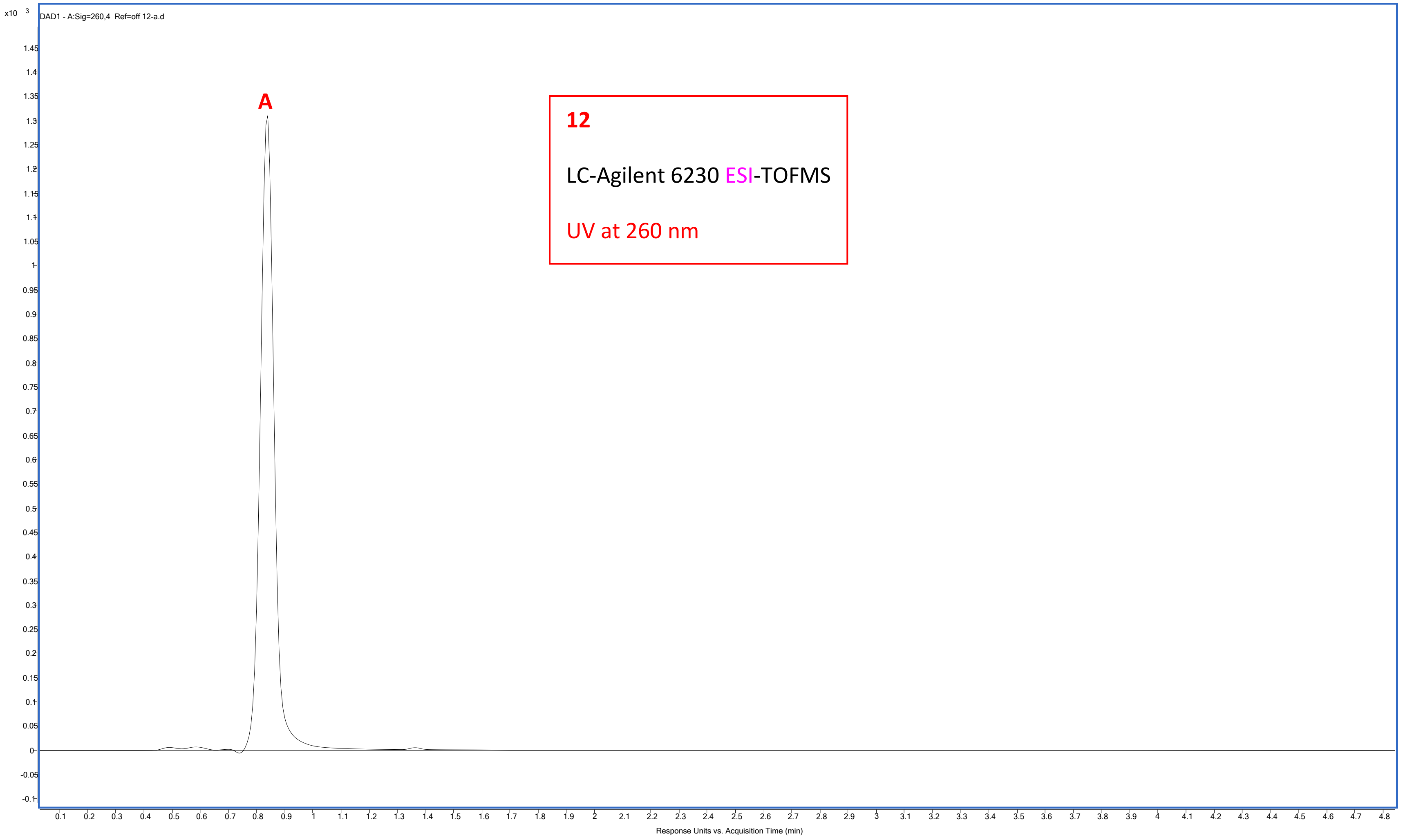
x10 3
DAD1 - A:Sig=260,4 Ref=off 12-a.d
A
12
LC-Agilent 6230 ESI-TOFMS
UV at 260 nm
Response Units vs. Acquisition Time (min)

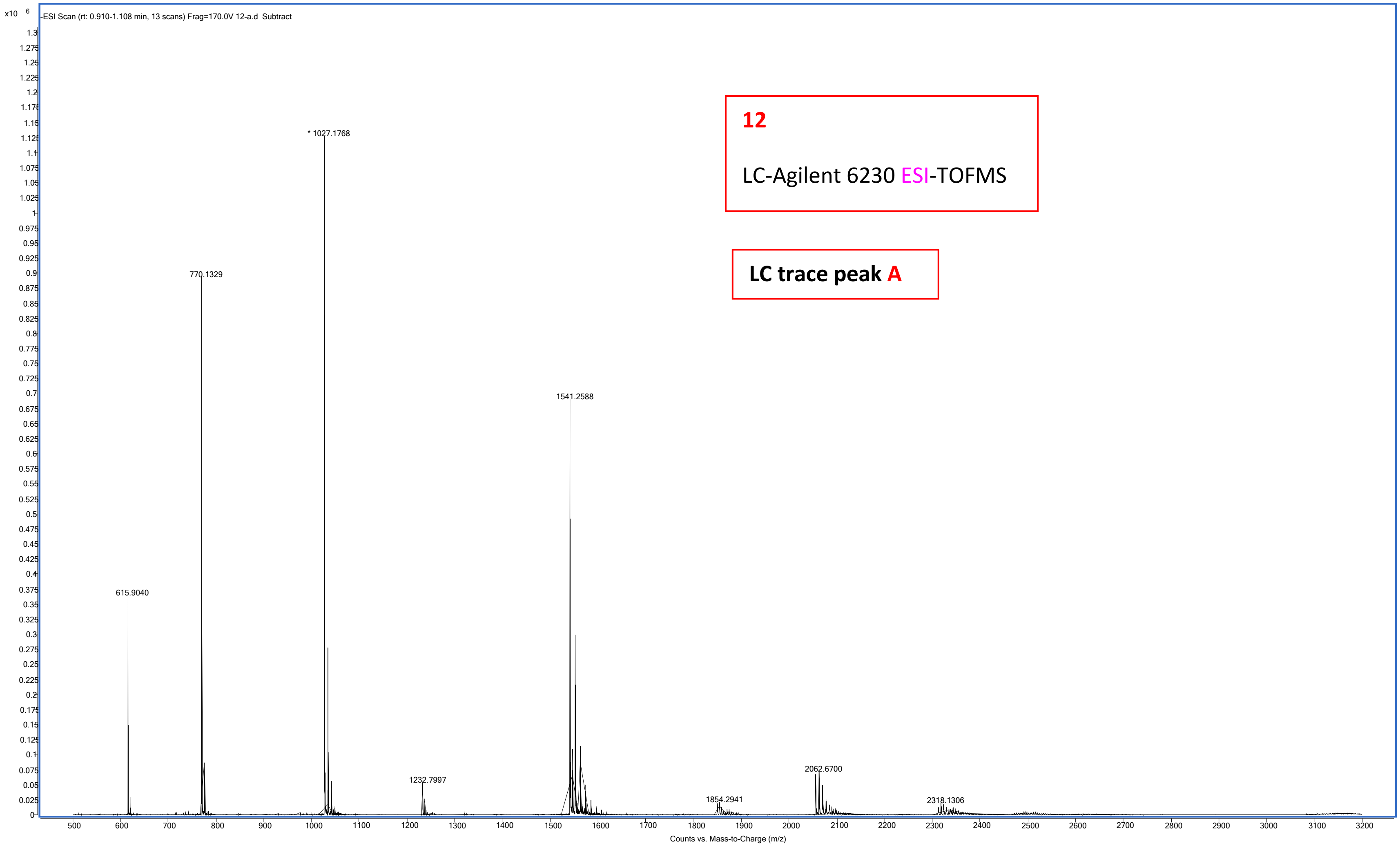

x10 6
-ESI Scan (rt: 0.910-1.108 min, 13 scans) Frag=170.0V 12-a.d Subtract
* 1027.1768
770.1329
1541.2588
615.9040
1232.7997
1854.2941
2062.6700
2318.1306
Counts vs. Mass-to-Charge (m/z)
12
LC-Agilent 6230 ESI-TOFMS
LC trace peak A

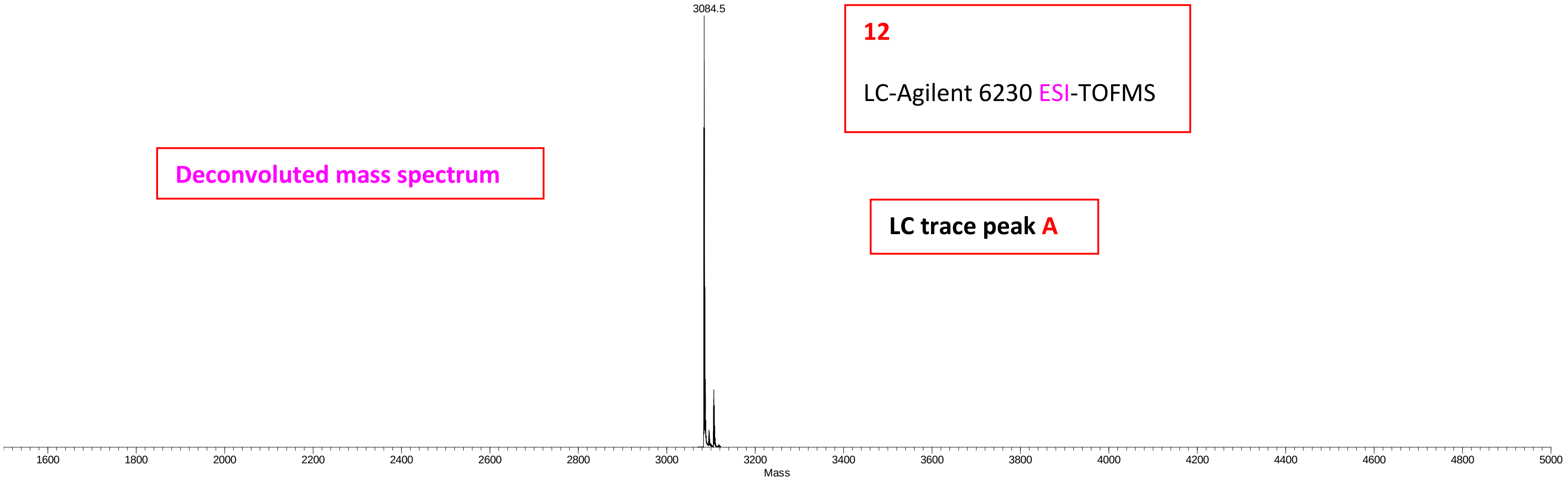

3084.5
12
LC-Agilent 6230 ESI-TOFMS
Deconvoluted mass spectrum
LC trace peak A
1600
1800
2000
2200
2400
2600
2800
3000
3200
3400
3600
3800
4000
4200
4400
4600
4800
5000
Mass

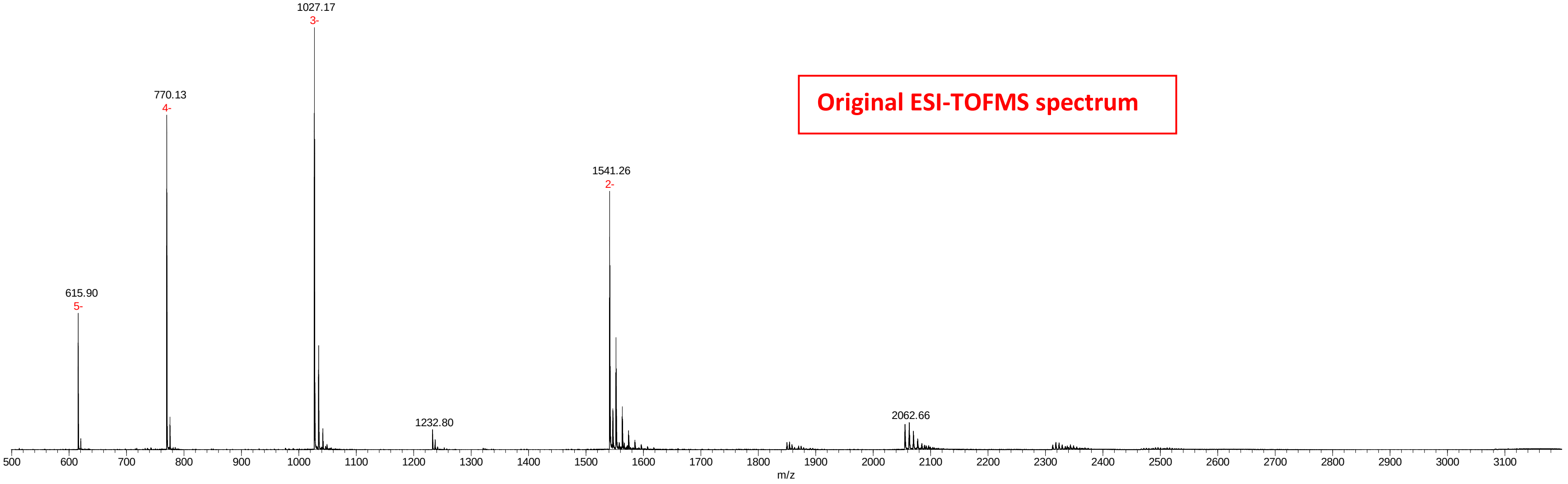

1027.17
3-
770.13
4-
Original ESI-TOFMS spectrum
1541.26
2-
615.90
5-
1232.80
2062.66
500
600
700
800
900
1000
1100
1200
1300
1400
1500
1600
1700
1800
1900
2000
2100
2200
2300
2400
2500
2600
2700
2800
2900
3000
3100
m/z

12

LC-Agilent 6230 ESI-TOFMS

Deconvoluted mass spectrum

LC trace peak A

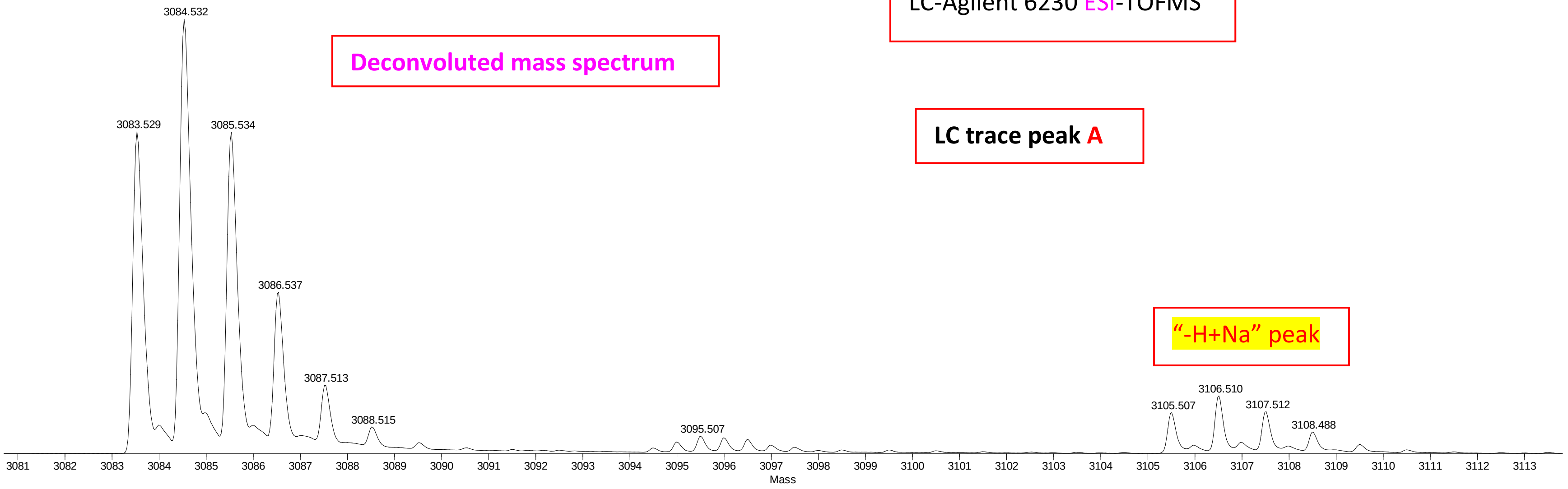

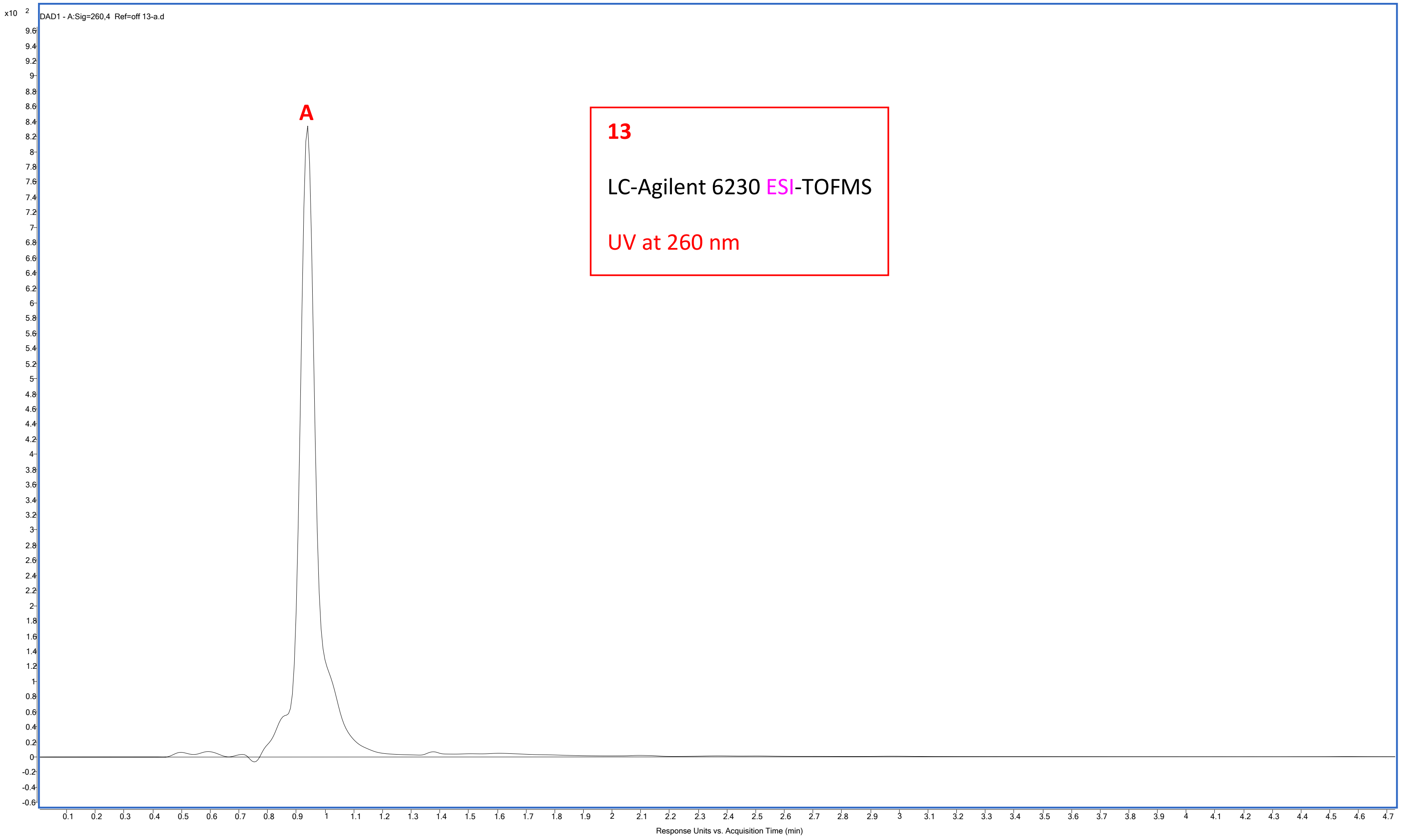

x10 2
DAD1 - A:Sig=260,4 Ref=off 13-a.d
A
13
LC-Agilent 6230 ESI-TOFMS
UV at 260 nm
Response Units vs. Acquisition Time (min)

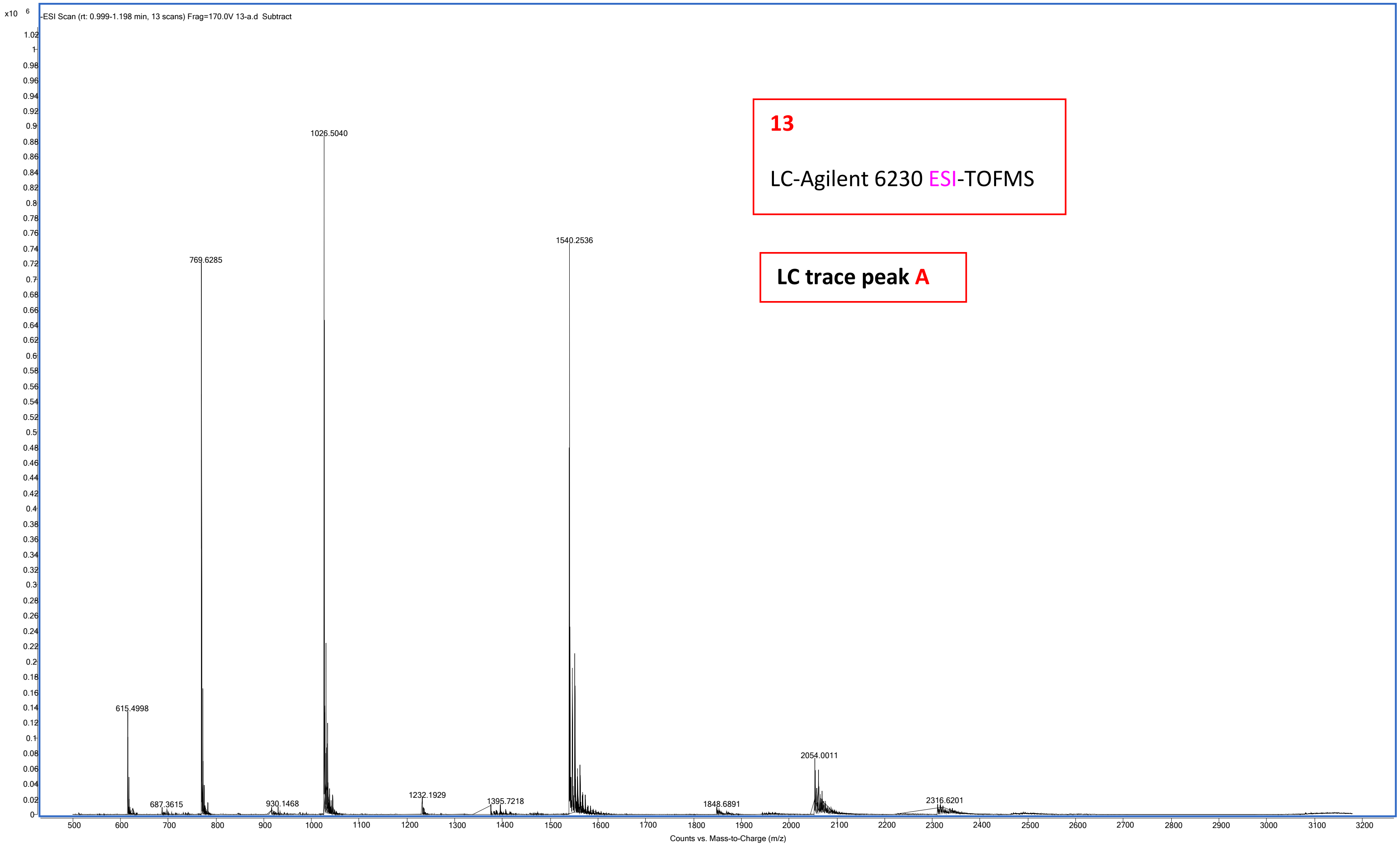

x10 6
-ESI Scan (rt: 0.999-1.198 min, 13 scans) Frag=170.0V 13-a.d Subtract
13
LC-Agilent 6230 ESI-TOFMS
LC trace peak A
1026.5040
1540.2536
769.6285
615.4998
687.3615
930.1468
1232.1929
1395.7218
1848.6891
2054.0011
2316.6201
Counts vs. Mass-to-Charge (m/z)

13

LC-Agilent 6230 ESI-TOFMS

Deconvoluted mass spectrum

LC trace peak A

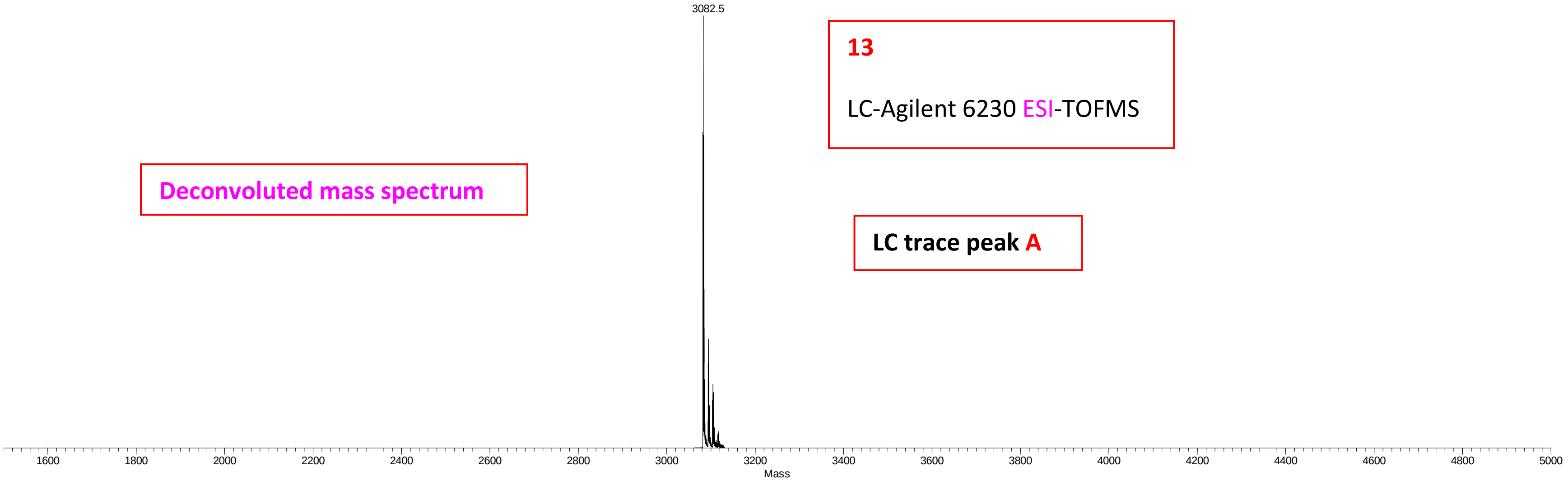

Original ESI-TOFMS spectrum

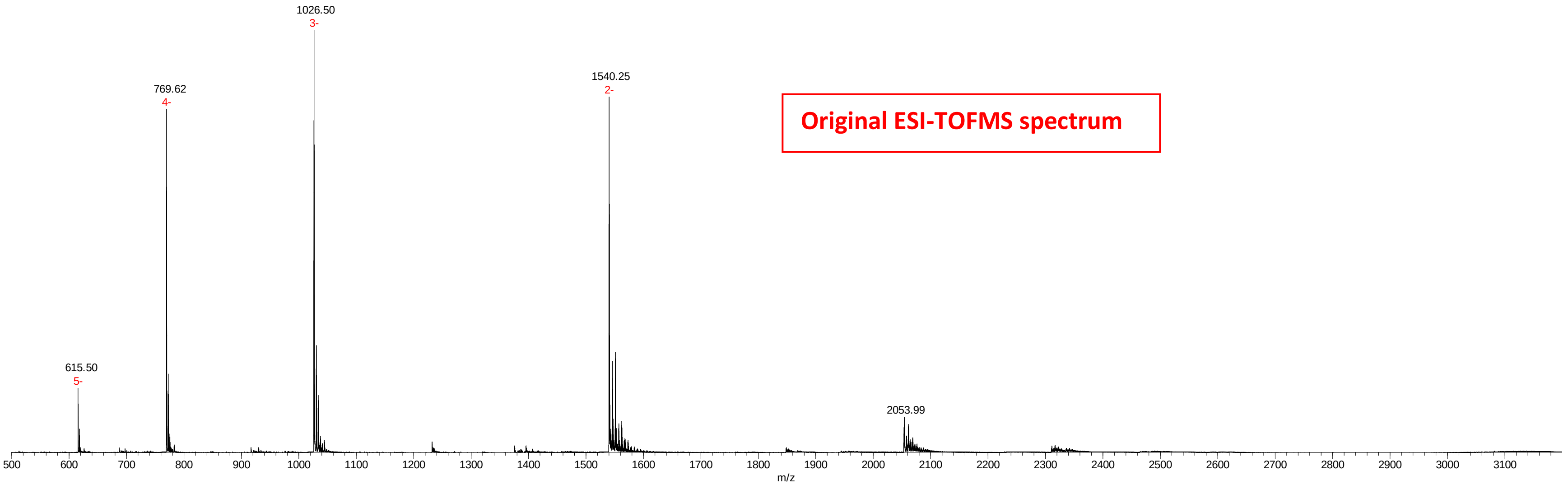

13

LC-Agilent 6230 ESI-TOFMS

Deconvoluted mass spectrum

LC trace peak A

"-H+Na" peak

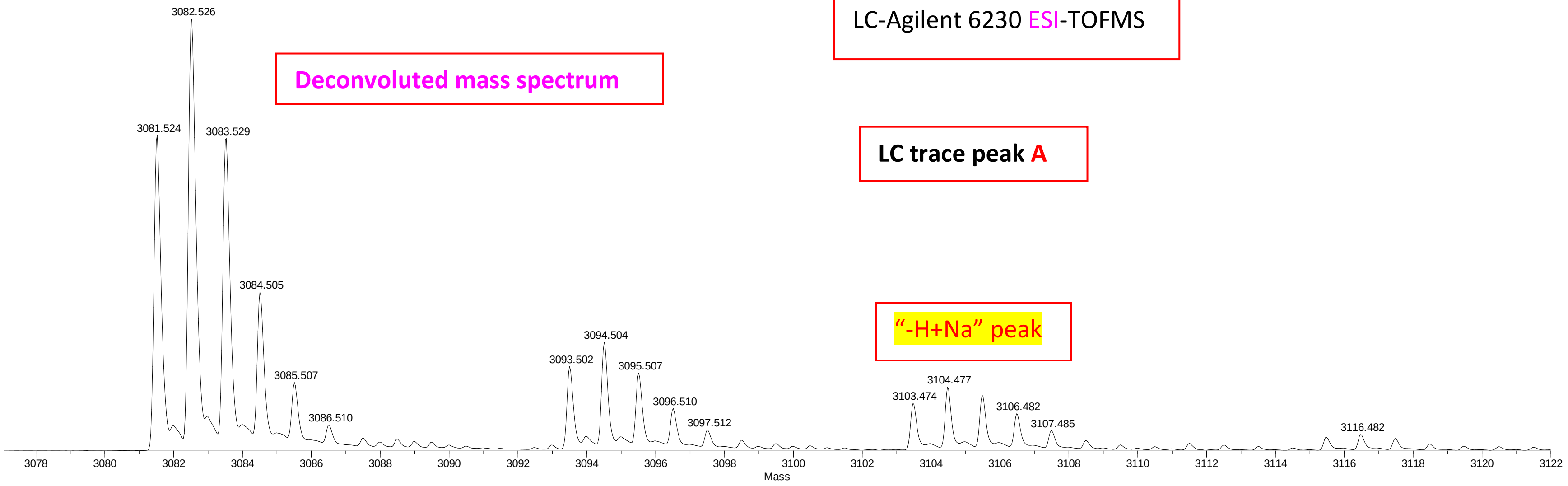

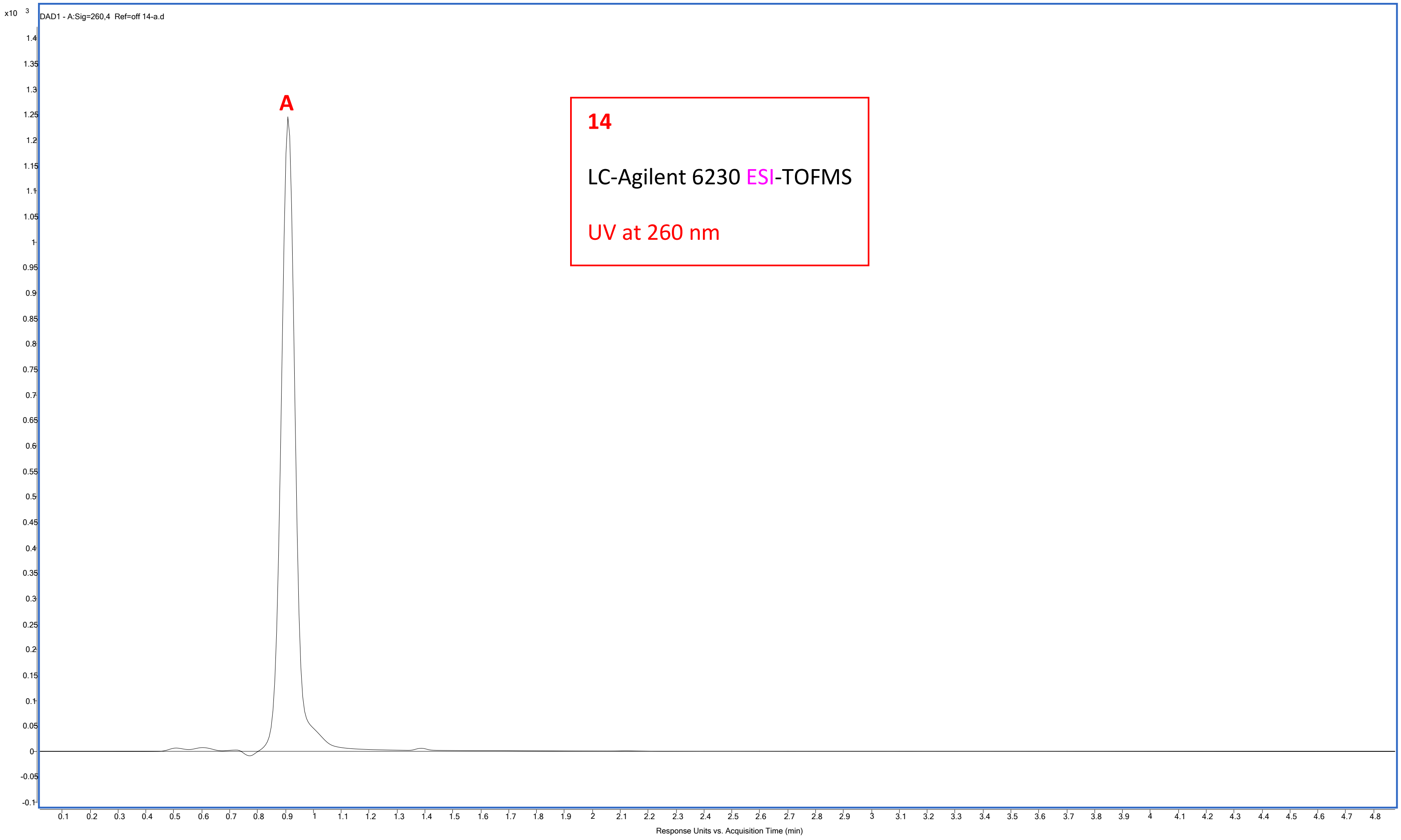
x10 3
DAD1 - A:Sig=260,4 Ref=off 14-a.d
A
14
LC-Agilent 6230 ESI-TOFMS
UV at 260 nm
Response Units vs. Acquisition Time (min)

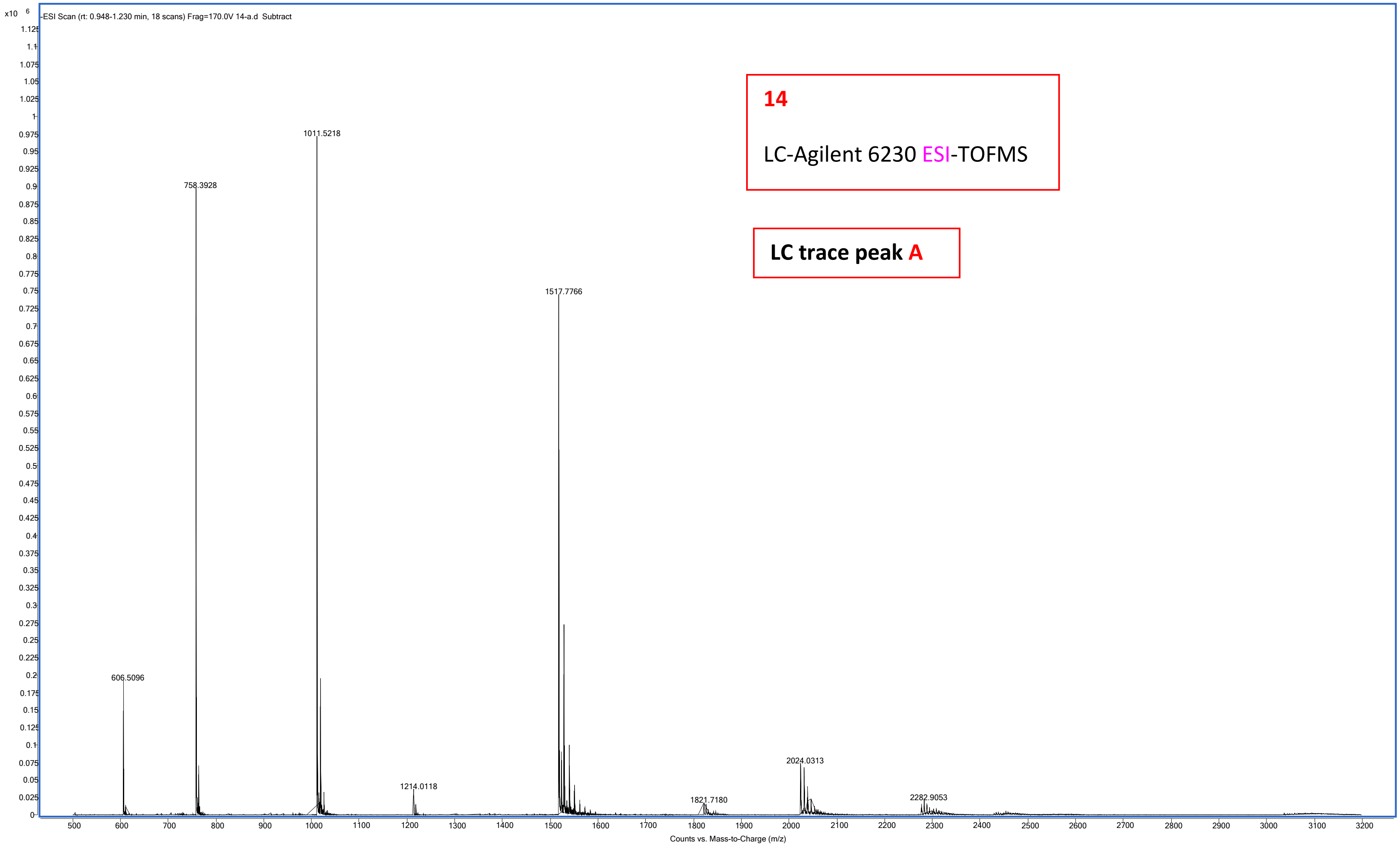

-ESI Scan (rt: 0.948-1.230 min, 18 scans) Frag=170.0V 14-a.d Subtract
x10 6
14
LC-Agilent 6230 ESI-TOFMS
LC trace peak A
606.5096
758.3928
1011.5218
1214.0118
1517.7766
1821.7180
2024.0313
2282.9053
Counts vs. Mass-to-Charge (m/z)

14

LC-Agilent 6230 ESI-TOFMS

Deconvoluted mass spectrum

LC trace peak A

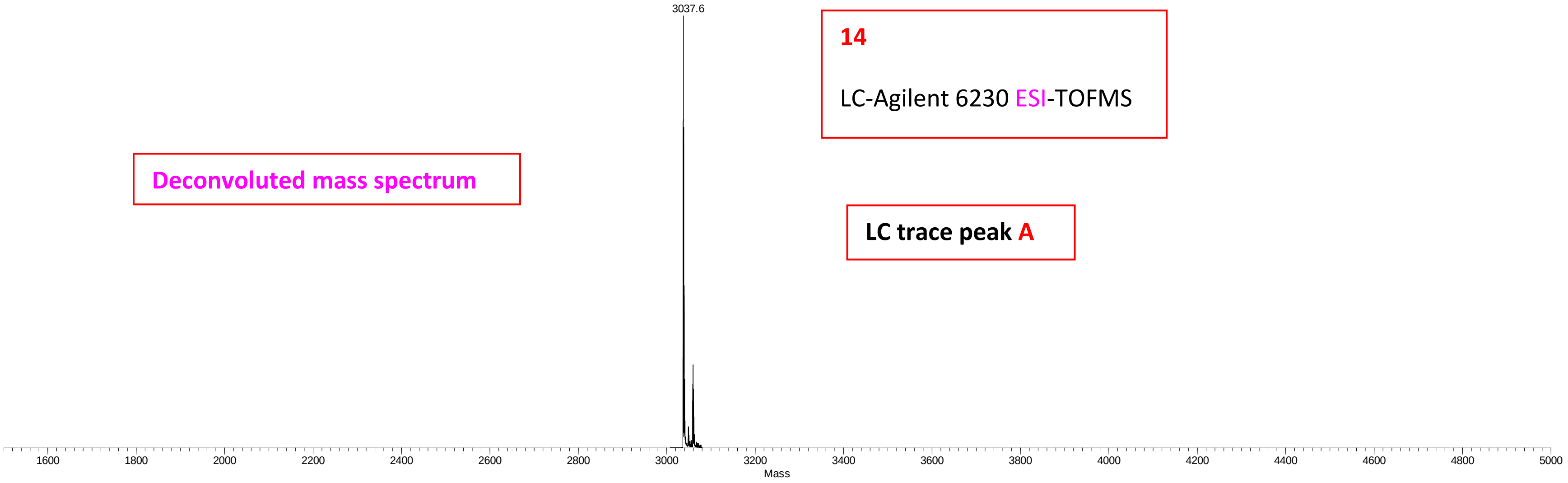

Original ESI-TOFMS spectrum

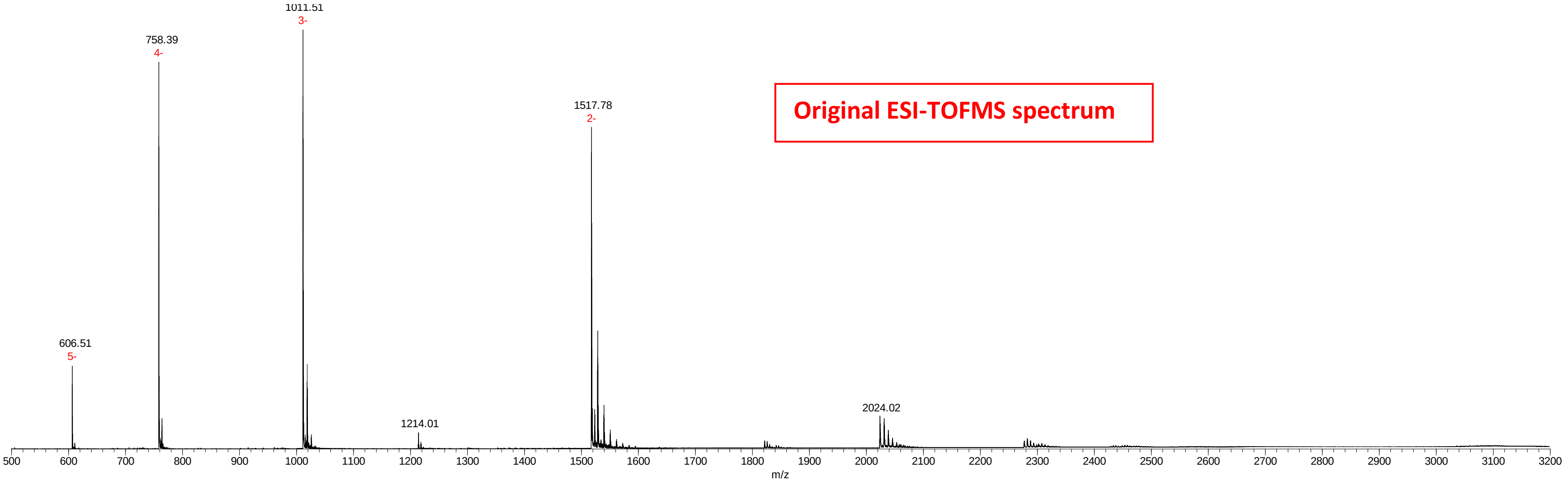

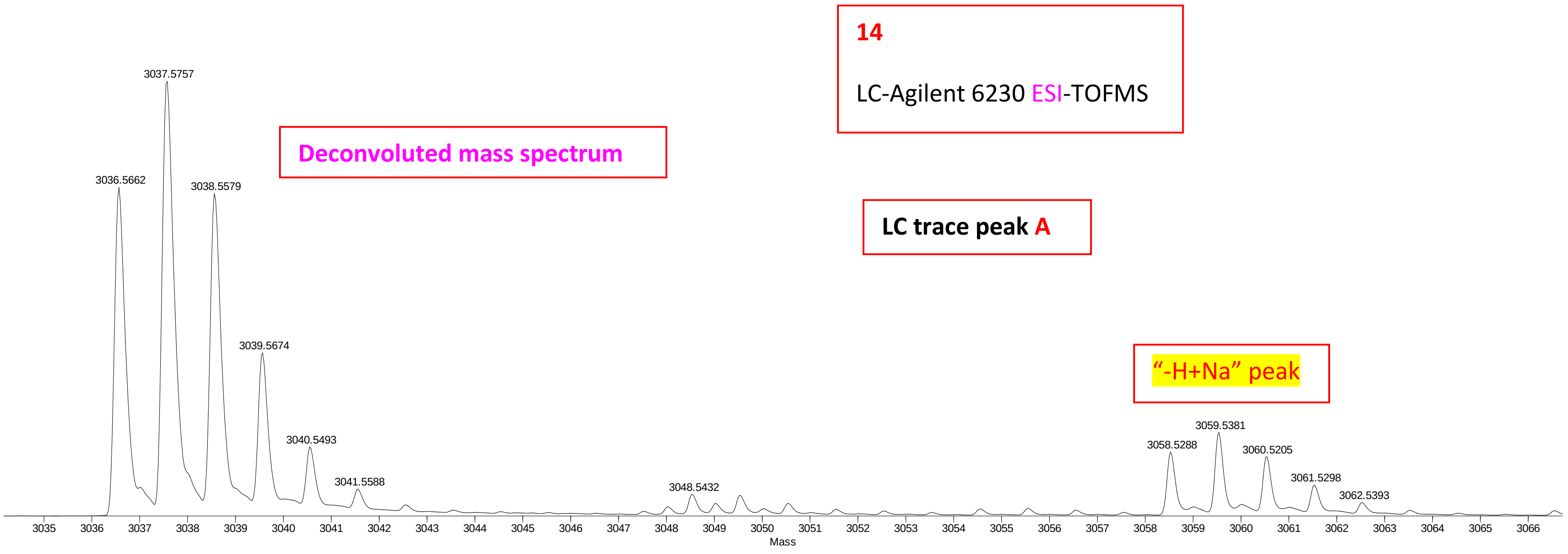
14
LC-Agilent 6230 ESI-TOFMS
Deconvoluted mass spectrum
LC trace peak A
"-H+Na" peak
3037.5757
3036.5662
3038.5579
3039.5674
3040.5493
3041.5588
3048.5432
3059.5381
3058.5288
3060.5205
3061.5298
3062.5393
Mass

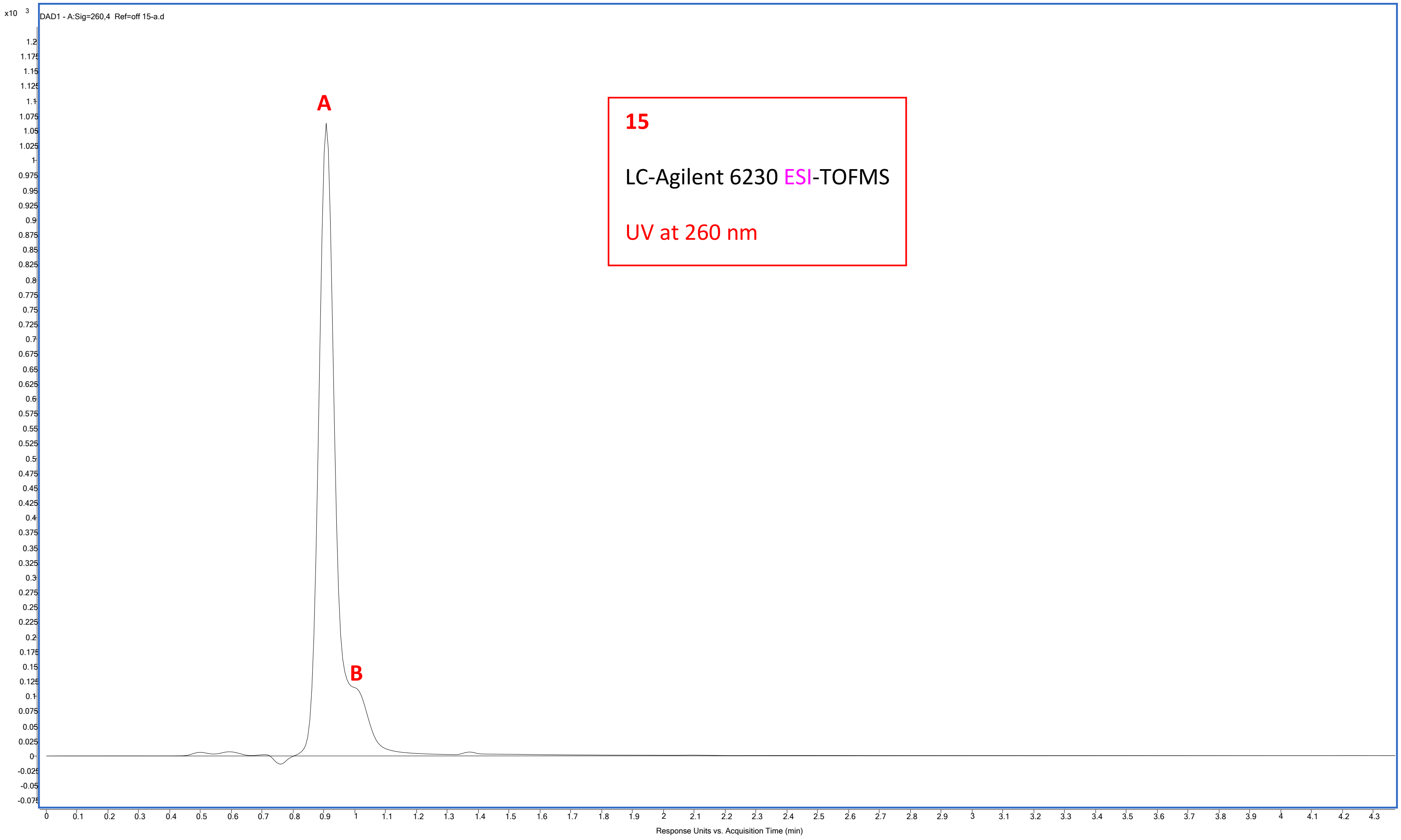
x10 3
DAD1 - A:Sig=260,4 Ref=off 15-a.d
A
B
15
LC-Agilent 6230 ESI-TOFMS
UV at 260 nm
Response Units vs. Acquisition Time (min)

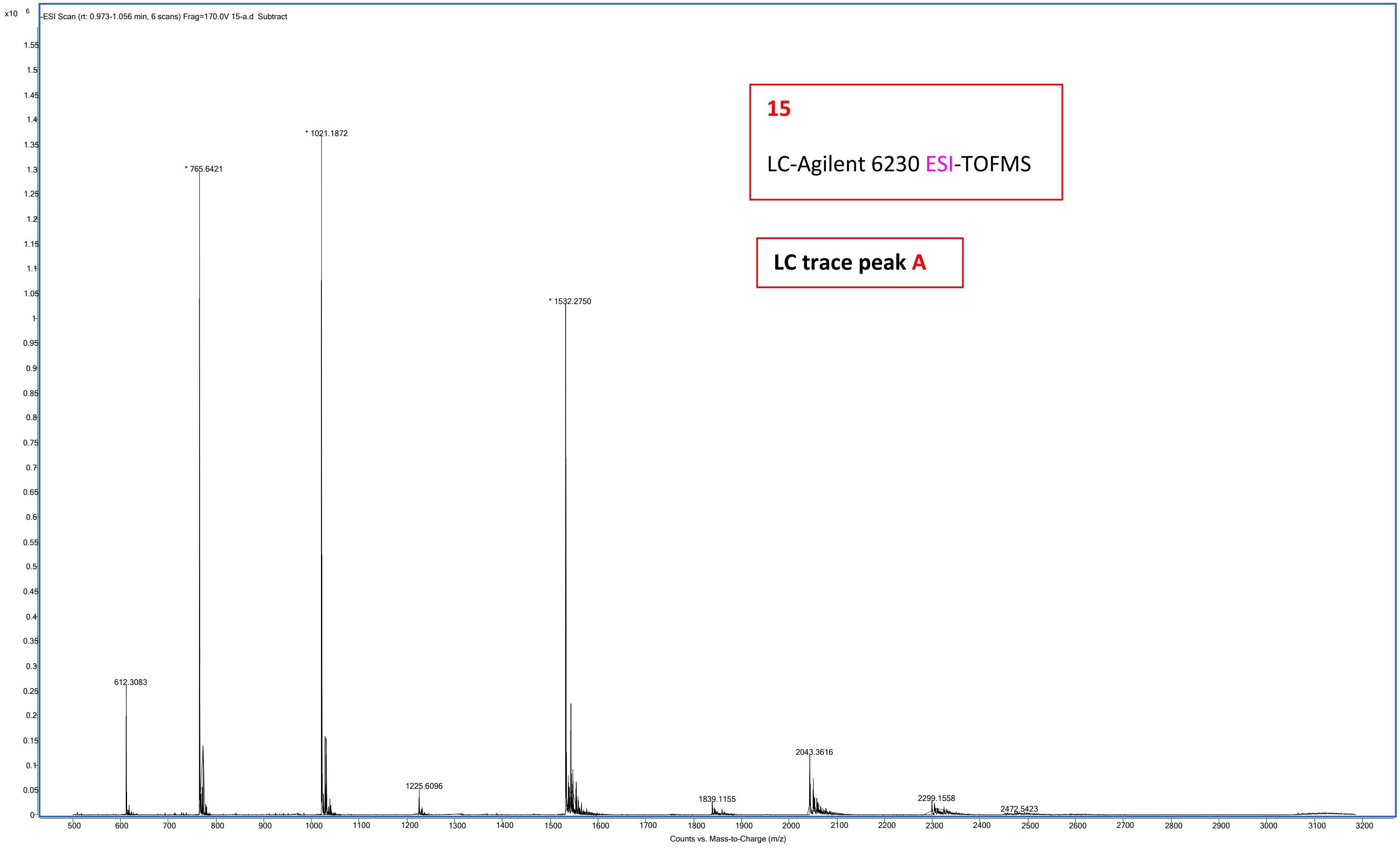

x10 6
-ESI Scan (rt: 0.973-1.056 min, 6 scans) Frag=170.0V 15-a.d Subtract
15
LC-Agilent 6230 ESI-TOFMS
LC trace peak A
* 765.6421
* 1021.1872
* 1532.2750
612.3083
1225.6096
1839.1155
2043.3616
2299.1558
2472.5423
Counts vs. Mass-to-Charge (m/z)

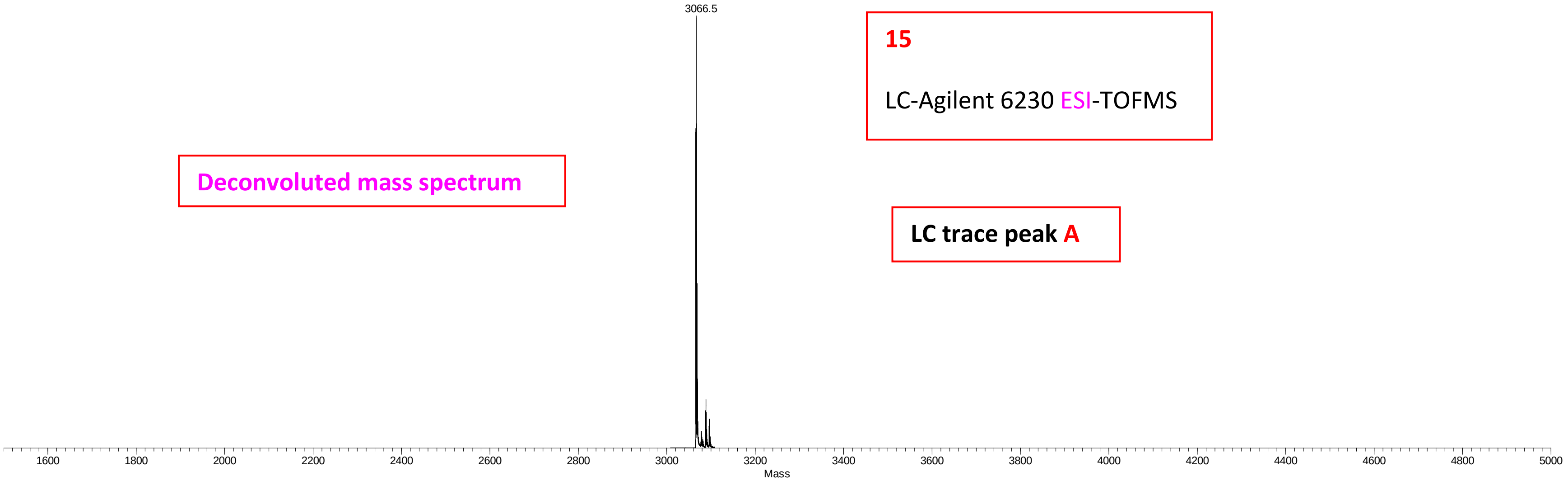

3066.5
15
LC-Agilent 6230 ESI-TOFMS
Deconvoluted mass spectrum
LC trace peak A
1600
1800
2000
2200
2400
2600
2800
3000
3200
3400
3600
3800
4000
4200
4400
4600
4800
5000
Mass

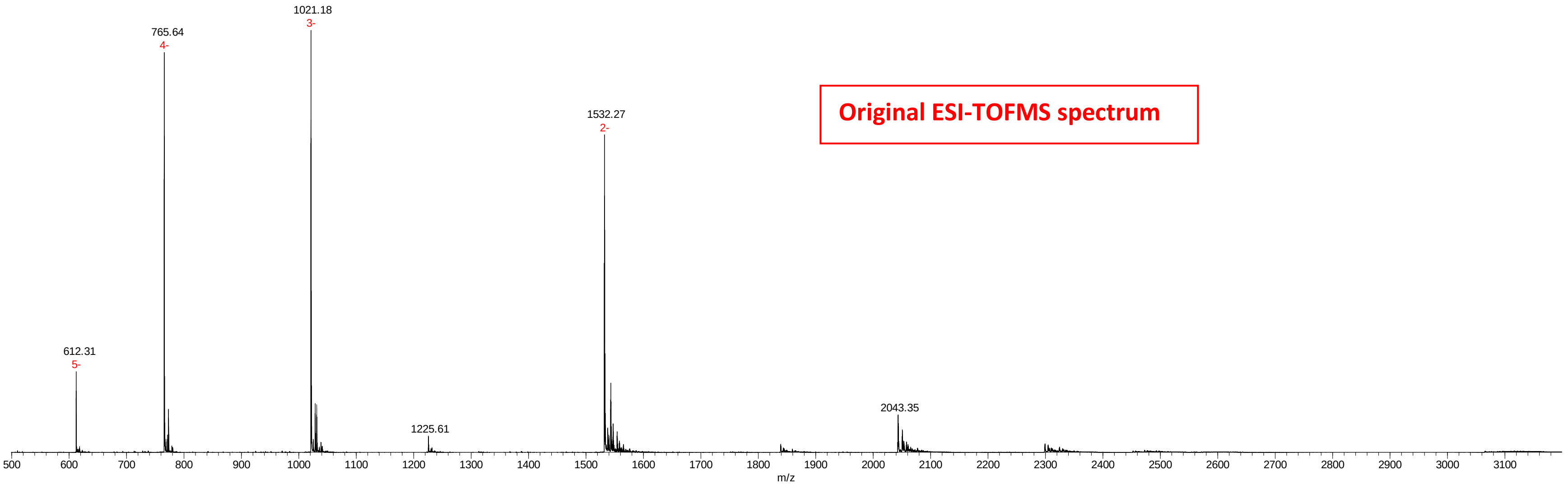

765.64
4-
1021.18
3-
1532.27
2-
612.31
5-
1225.61
2043.35
Original ESI-TOFMS spectrum
500
600
700
800
900
1000
1100
1200
1300
1400
1500
1600
1700
1800
1900
2000
2100
2200
2300
2400
2500
2600
2700
2800
2900
3000
3100
m/z

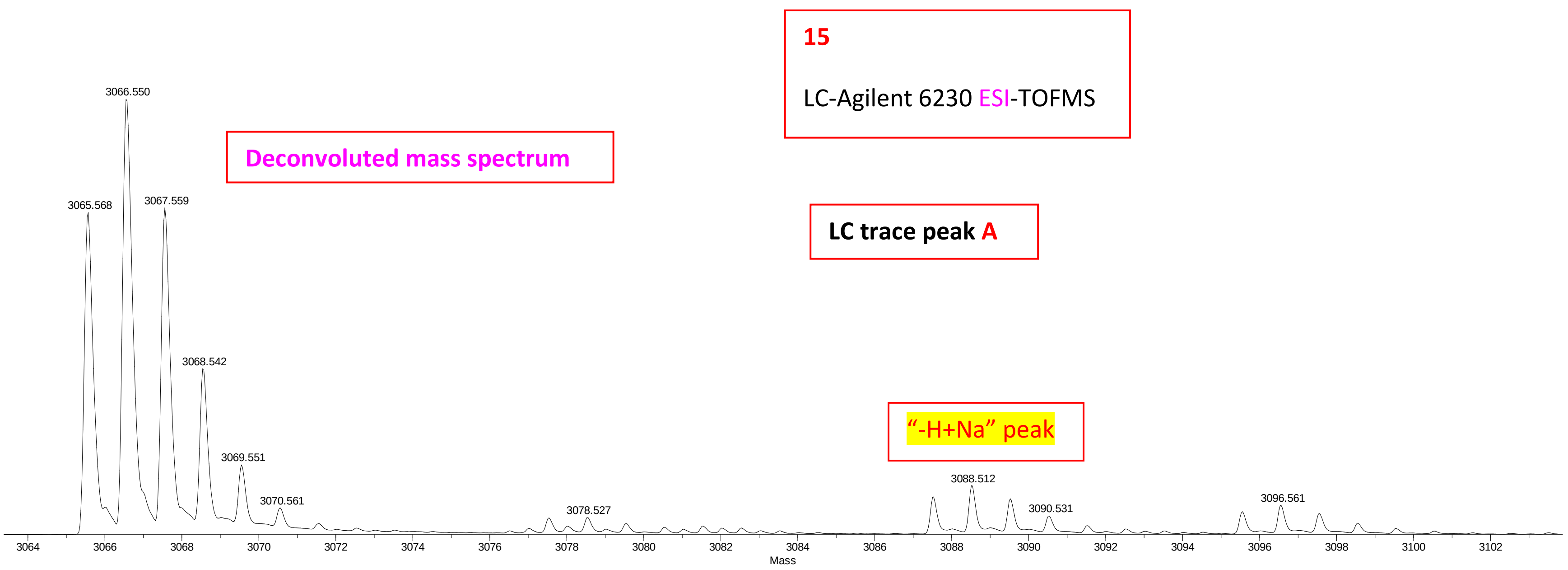
15
LC-Agilent 6230 ESI-TOFMS
Deconvoluted mass spectrum
LC trace peak A
"-H+Na" peak
3066.550
3065.568
3067.559
3068.542
3069.551
3070.561
3078.527
3088.512
3090.531
3096.561
Mass

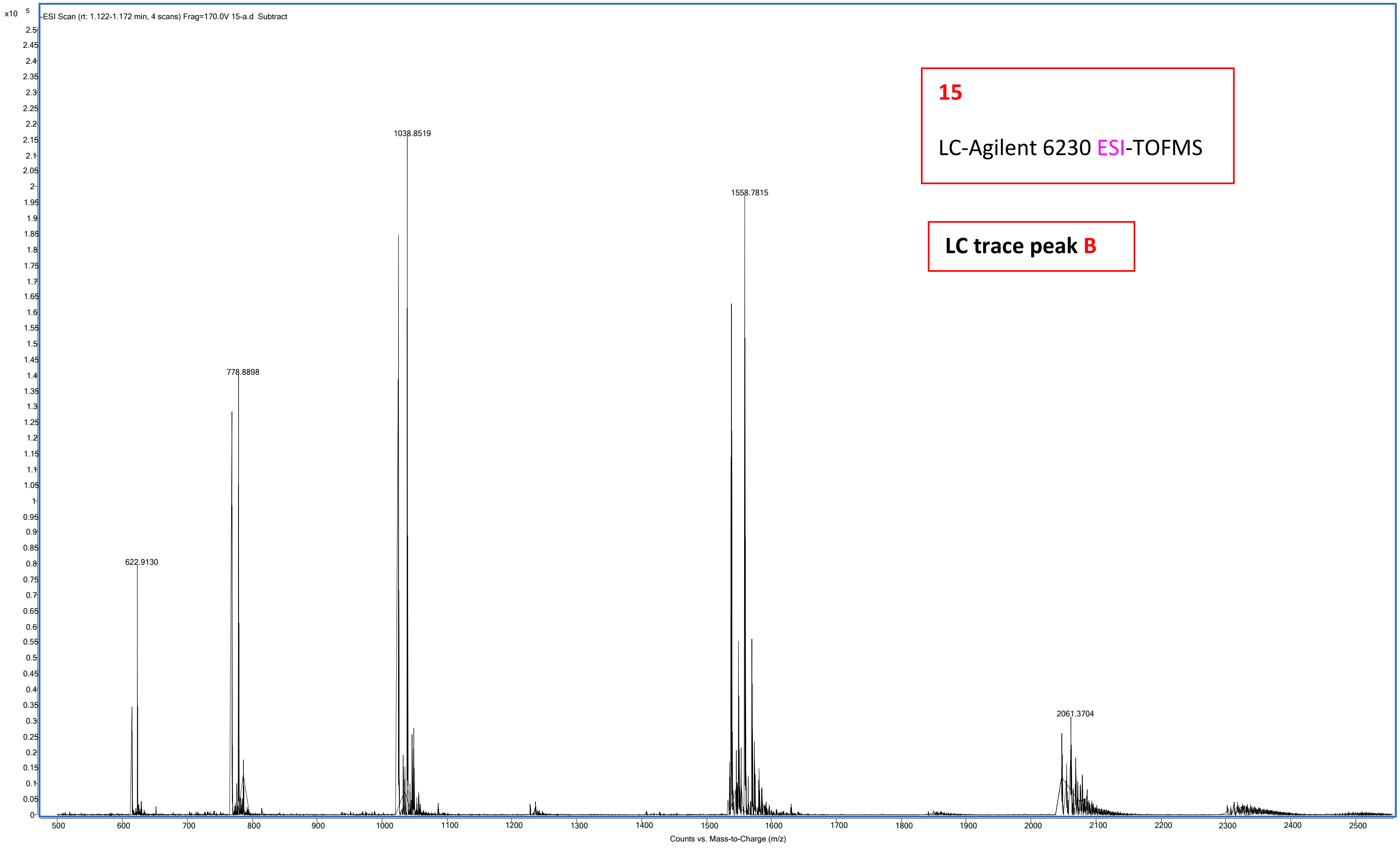

x10 5
-ESI Scan (rt: 1.122-1.172 min, 4 scans) Frag=170.0V 15-a.d Subtract
15
LC-Agilent 6230 ESI-TOFMS
LC trace peak B
1038.8519
1558.7815
778.8898
622.9130
2061.3704
Counts vs. Mass-to-Charge (m/z)

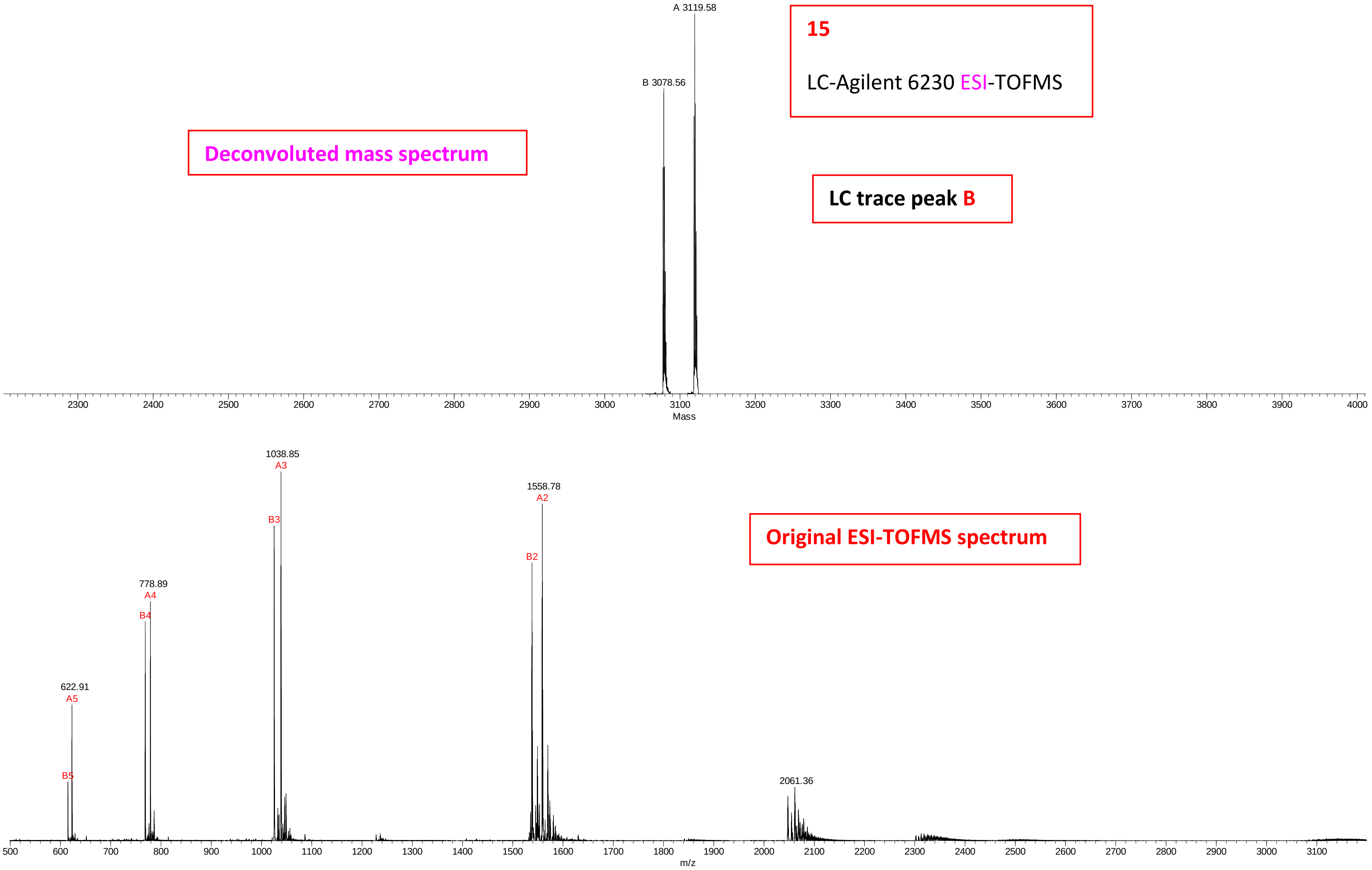
15
LC-Agilent 6230 ESI-TOFMS
Deconvoluted mass spectrum
LC trace peak B
A 3119.58
B 3078.56
Mass
Original ESI-TOFMS spectrum
1038.85
A3
B3
1558.78
A2
B2
778.89
A4
B4
622.91
A5
B5
2061.36
m/z
