# Supplementary material for: Sequence-specific fluorescence turn-on arises from base pairing-templated tautomerism in the tricyclic cytidine analogue DEAtC
Source: RSC Chem Biol. 2025 Dec 7;7(2):216–25. doi: 10.1039/d5cb00243e (PMC12690291; doi:10.1039/d5cb00243e)
Supplement: CB-007-D5CB00243E-s002 [file CB-007-D5CB00243E-s002.pdf]

Supplementary Information (SI) for RSC Chemical Biology.
This journal is © The Royal Society of Chemistry 2025

Supplementary Information for

Sequence-specific fluorescence turn-on arises from base pairing-templated tautomerism in the tricyclic cytidine analogue ^{DEA}tC

Ana Shalamberidze, Harrison R. Pearce, Andrew L. Cooksy, and Byron W. Purse*

Department of Chemistry and Biochemistry, San Diego State University, San Diego, CA, USA

*Correspondence: bpurse@sdsu.edu

Contents

Experimental Procedures……3

Synthesis of oligonucleotide probes……3

Table S1. ^{DEA}tC oligonucleotides……3

Table S2. Complementary oligonucleotides……4

Table S3. Sequences of modified complementary oligonucleotides……4

Steady-state fluorescence spectroscopy to measure emission, excitation……5

Supplemental Data and Figures……6

Figure S1. Titration of the ssDNA GXC sequence with complementary modified sequences……6

Figure S2. Titration of the ssDNA AXA' sequence with complementary modified sequences……8

Figure S3. Titration of the ssDNA CXA sequence with complementary modified sequences……10

Figure S4. Titration of the ssDNA AXA sequence with complementary modified sequences……12

Figure S5. Absorption (dashed) and excitation (solid; collected using an emission wavelength of 500 nm) of the single-stranded (ss) GXC oligonucleotide and in hybrid duplexes with ^{DEA}tC base paired with G, A, I, 8-oxo-G, and dSpacer (1′,1′-dideoxyribose). ……13

Figure S6. Emission spectra of GXC sequence with modified nucleosides……14

Figure S7. Emission spectra of AXA' sequence with modified nucleosides……14

Figure S8. Emission spectra of CXA sequence with modified nucleosides……14

Figure S9. Emission spectra of AXA sequence with modified nucleosides……………15

Table S4. Paired sequences and relative fluorescence intensity changes for ds DNA oligonucleotides for the AXA, GXC, CXA and AXA' sequences, where X = ^{DEA}tC……………………………………………………………………………..15

Table S5. Steady-State fluorescence measurements of ^{DEA}tC in single- and double-stranded DNA oligonucleotides for the AXA sequence……………………………………………16

Figure S10. Relative fluorescence intensity of the single-stranded (ss) AXA' and AXA oligonucleotides and their hybrid duplexes……………………..………….……………16

Figure S11. Excitation spectra of the single-stranded (ss) AXA' and AXA oligonucleotide and hybrid duplexes……………………………………………………………………17

Figure S12. Emission spectra recorded upon excitation at different wavelengths and normalized for the corresponding absorbance intensity for AXA'……………………..18

Figure S13. Emission spectra recorded upon excitation at different wavelengths and normalized for the corresponding absorbance intensity for AXA…………..……………19

Computational Section……………………………………………………………………...20

Monomer Calculations……………………………………………………………...20

Trimer Calculations…………………………………………………………………20

Table S6. Optimized molecular geometries of the C-like and T-like tautomer……………20

Table S7. Base Stacked and base paired geometries……………………………………...21

Table S8. TD-B3LYP monomer absorption wavelengths and oscillator strengths………..23

Table S9. TD-CAM-B3LYP monomer absorption wavelengths and oscillator strengths...23

Table S10. TD-B3LYP and TD-CAM-B3LYP absorption data ONIOM and QM………24

Table S11. Relative tautomer stability and electronic energies……………………..….25

References ……………………………………………………………………………….26

Experimental Procedures

Synthesis of oligonucleotide probes

^{DEA}tC-containing oligonucleotides were synthesized by the Keck Oligonucleotide Synthesis Facility at Yale University (New Haven, CT) using the ^{DEA}tC 2′-deoxyribonucleoside phosphoramidite, prepared in-house according to a published procedure.[1] Solid-phase oligonucleotide synthesis was carried out under standard conditions, employing a 10-fold extended coupling time for the ^{DEA}tC phosphoramidite. The resulting oligonucleotides were deprotected and purified by HPLC.

Oligonucleotides containing 8-oxo-G, O^6-MeG, nebularine, or 6mA were similarly synthesized by the Keck Facility, while oligonucleotides containing D-spacer, iso-dG, 2,6-diaminopurine, inosine, and canonical bases were prepared by Integrated DNA Technologies, Inc. (Coralville, IA). Standard solid-phase synthesis and deprotection protocols were used in all cases, except that AMA deprotection conditions were employed for the 8-oxoG oligonucleotide. All modified oligonucleotides were purified and characterized by HPLC.

Table S1. Sequences of ^{DEA}tC oligonucleotides.

Sequence Name	Sequence (5′ to 3′)	Calculated Mass	Measured Mass
GXC	CGCAGXCTCG	3164.5985	3165.6
AXA	CGCAAXATCG	3172.6148	3173.6
CXA	CGCACXATCG	3148.6036	3149.6
AXA’	CGCAAXATC	2843.5623	2844.55

The position of ^{DEA}tC is noted by X.

Table S2. Sequences of complementary oligonucleotides.

Complementary Sequence Name	Sequence (5′ to 3′)	Calculated Mass	Measured Mass
Match (G)	CGAGGCTGCG	3069.0	3068.70
	CGATGTTGCG	3059.0	3059.00
	CGATGGTGCG	3084.0	3084.10
Mismatch (A)	CGAGACTGCG	3053.0	3052.60
	CGATATTGCG	3043.0	3042.80
	CGATAGTGCG	3068.0	3067.80
Inosine (I)	CGAG/I/CTGCG	3054.0	3053.90
	CGAT/I/TTGCG	3044.0	3043.80
	CGAT/I/GTGCG	3069.0	3068.90
Dspacer	CGAG/dspacer/CTGCG	2919.9	2919.80
	CGAT/dspacer/TTGCG	2909.9	2909.90
	CGAT/dspacer/GTGCG	2934.9	2934.80
2,6-diaminopurine	CGAG/2,6-dap/CTGCG	3068.0	3068.00
	CGAT/2,6-dap/TTGCG	3058.0	3057.50
	CGAT/2,6-dap/GTGCG	3083.1	3082.90
isodG	CGAG/IsodG/CTGCG	3069.0	3068.60
	CGAT/IsodG/TTGCG	3059.0	3058.30
	CGAT/IsodG/GTGCG	3084.0	3083.50

Table S3. Sequences of modified complementary oligonucleotides.

Complementary Sequence Name	Sequence (5′ to 3′)	Calculated Mass	Measured Mass
8oxoG	CGAG/8oxoG/CTGCG	3083.5445	3084.5
	CGAT/8oxoG/TTGCG	3073.5377	3074.5
	CGAT/8oxoG/GTGCG	3098.5442	3099.5
O6MeG	CGAG/O6MeG/CTGCG	3081.5652	3082.5
	CGAT/O6MeG/TTGCG	3071.5584	3072.5
	CGAT/O6MeG/GTGCG	3096.5649	3097.6
Nebularine	CGAG/Neb/CTGCG	3036.5438	3037.6
	CGAT/Neb/TTGCG	3026.5370	3027.5
	CGAT/Neb/GTGCG	3051.5434	3052.6
6mA	CGAG/N6MeA/CTGCG	3065.5703	3066.5
	CGAT/N6MeA/TTGCG	3055.5635	3056.5
	CGAT/N6MeA/GTGCG	3080.5700	3081.6

Steady-state fluorescence spectroscopy to measure emission, excitation, and absorbance

Oligonucleotides were dissolved in Milli-Q water and 1.5 nmol was suspended in 2 mL 1 × PBS buffer at pH 7.4 prepared with nuclease-free water (deuterated water was used for solvent isotope effect studies). The single-stranded DNA sequences were titrated with up to 200% complementary sequences to achieve oversaturation and emission spectra were collected at 25% increments. The measurements were taken in quartz sub-micro cuvettes with a 1.0 cm path length (Starnacell Inc.) at 25 °C. Emission and excitation spectra were recorded under steady-state conditions using a PTI QuantaMaster QM-400 fluorometer (Horiba Scientific) and absorbance spectra were measured using a Shimadzu UC-1700 Pharmaspec spectrophotometer. The absorbance of the samples was measured with an initial absorbance near 0.10, approximately 37 µM of fluorophore, at 395 nm and the total emission spectrum was collected in the range of 405–680 nm. The excitation spectra were collected at an emission wavelength of 500 nm and in the range of 200–680 nm.

Supplemental Data and Figures

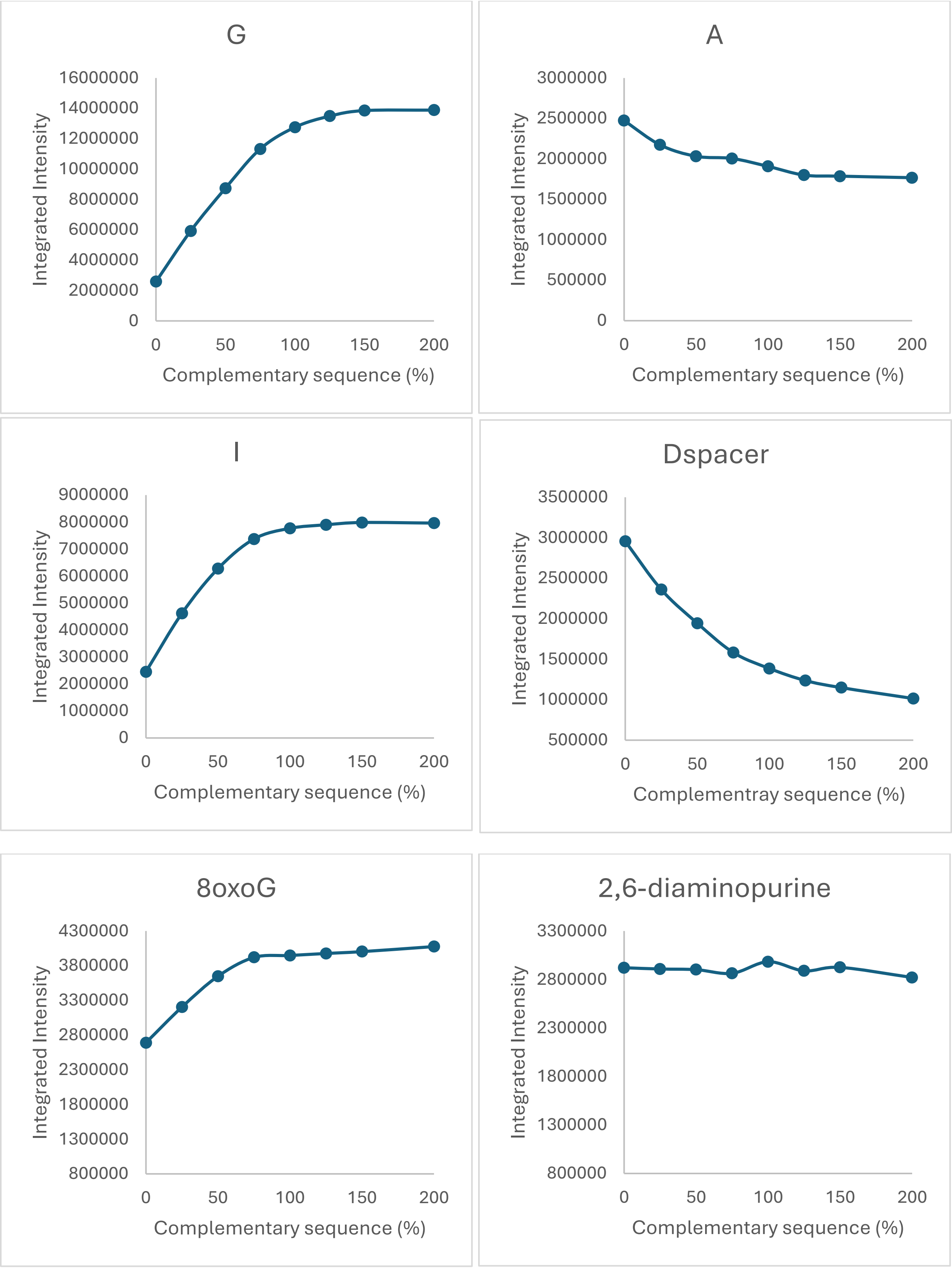

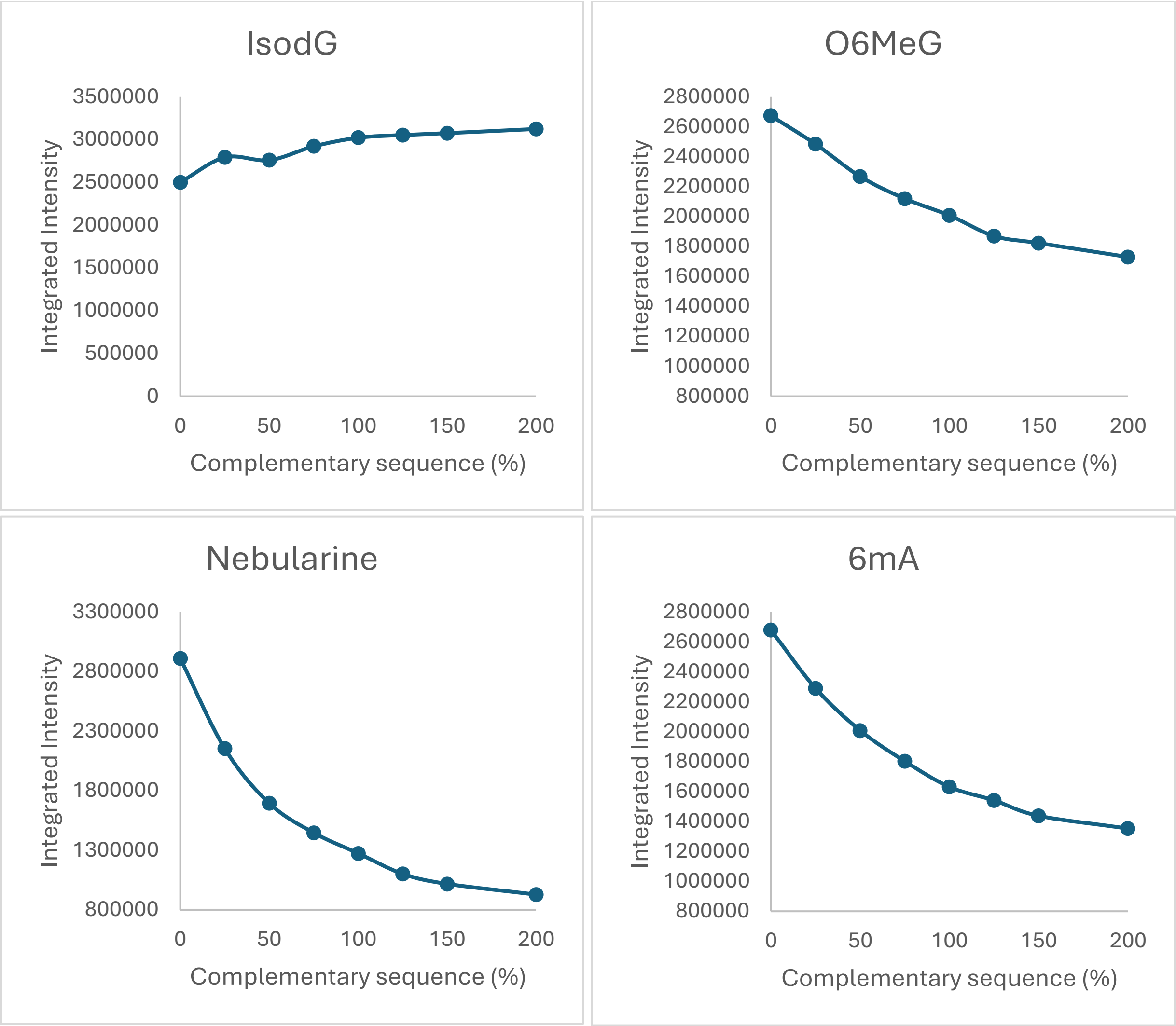

Figure S1. Emission of the ssDNA GXC sequence following titration with complementary modified sequences. Data were recorded using 1× PBS buffer at pH 7.4 with the GXC oligo concentration at 0.72 µM and the excitation wavelength of 395 nm.

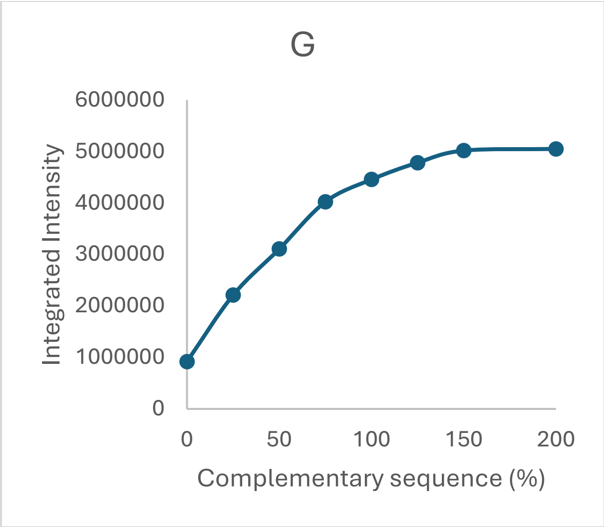
G
Integrated Intensity
6000000
5000000
4000000
3000000
2000000
1000000
0
0
50
100
150
200
Complementary sequence (%)

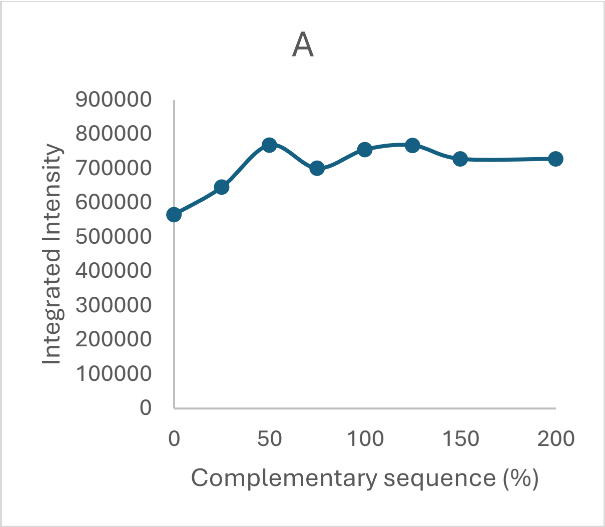
A
Integrated Intensity
900000
800000
700000
600000
500000
400000
300000
200000
100000
0
0
50
100
150
200
Complementary sequence (%)

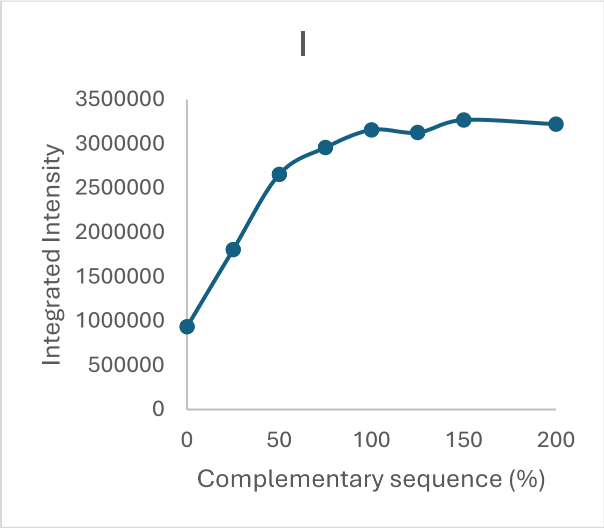
I
Integrated Intensity
3500000
3000000
2500000
2000000
1500000
1000000
500000
0
0
50
100
150
200
Complementary sequence (%)

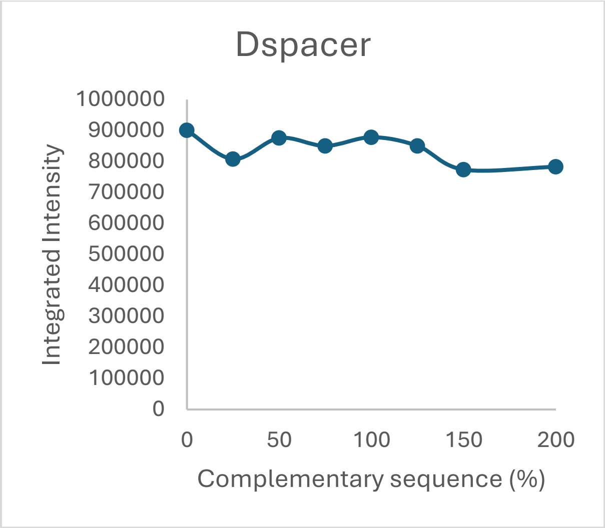
Dspacer
Integrated Intensity
1000000
900000
800000
700000
600000
500000
400000
300000
200000
100000
0
0
50
100
150
200
Complementary sequence (%)

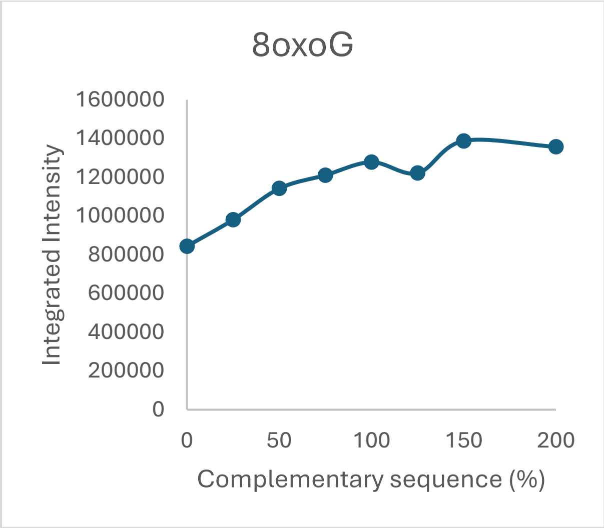
8oxoG
Integrated Intensity
1600000
1400000
1200000
1000000
800000
600000
400000
200000
0
0
50
100
150
200
Complementary sequence (%)

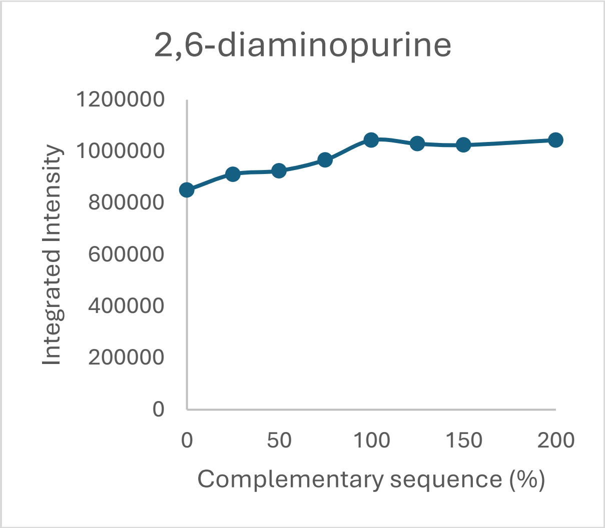
2,6-diaminopurine
Integrated Intensity
1200000
1000000
800000
600000
400000
200000
0
0
50
100
150
200
Complementary sequence (%)

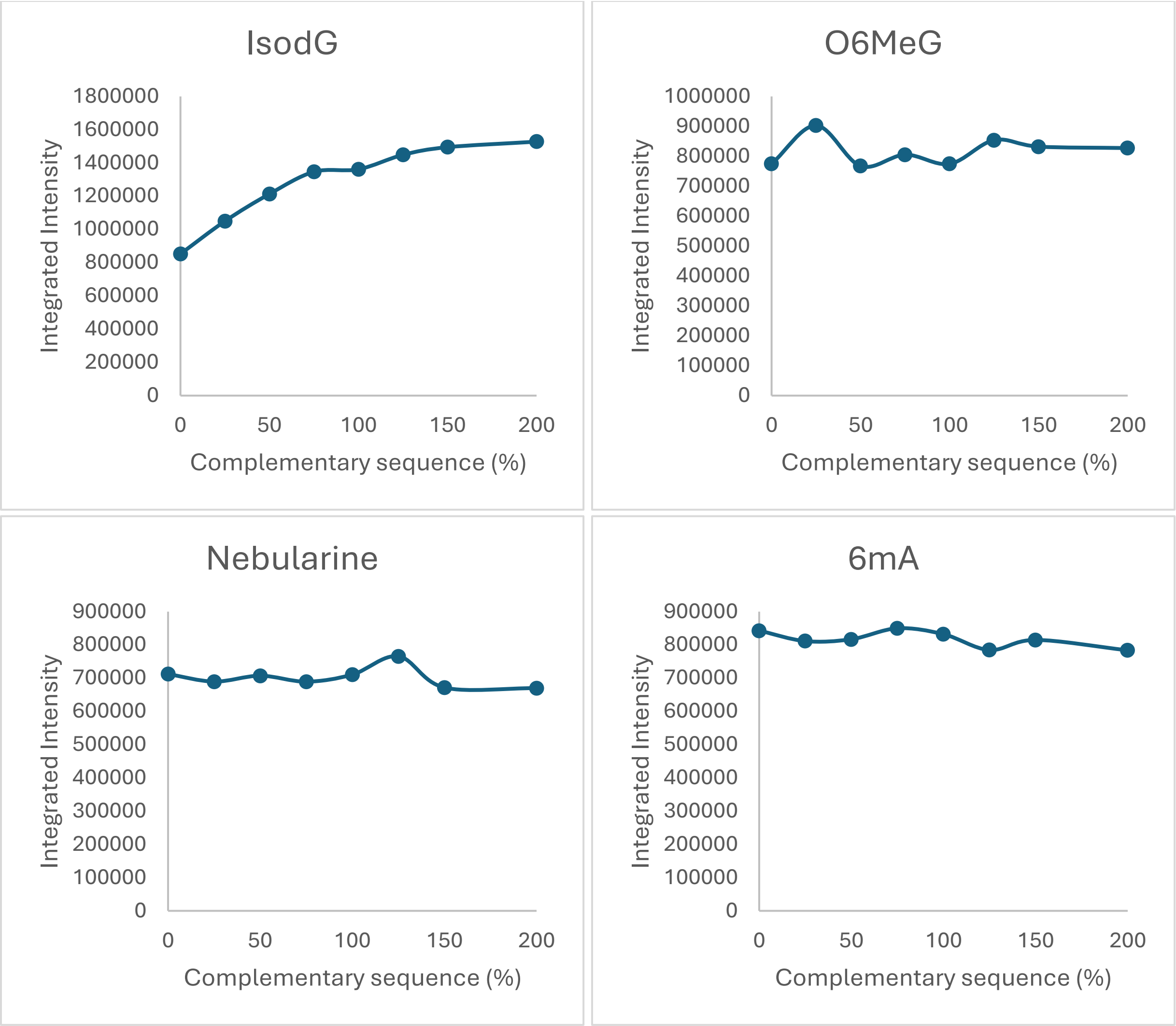

Figure S2. Emission of the ssDNA AXA’ sequence following titration with complementary modified sequences. Data were recorded using 1× PBS buffer at pH 7.4 with the AXA’ oligo concentration at 0.72 µM and the excitation wavelength of 395 nm.

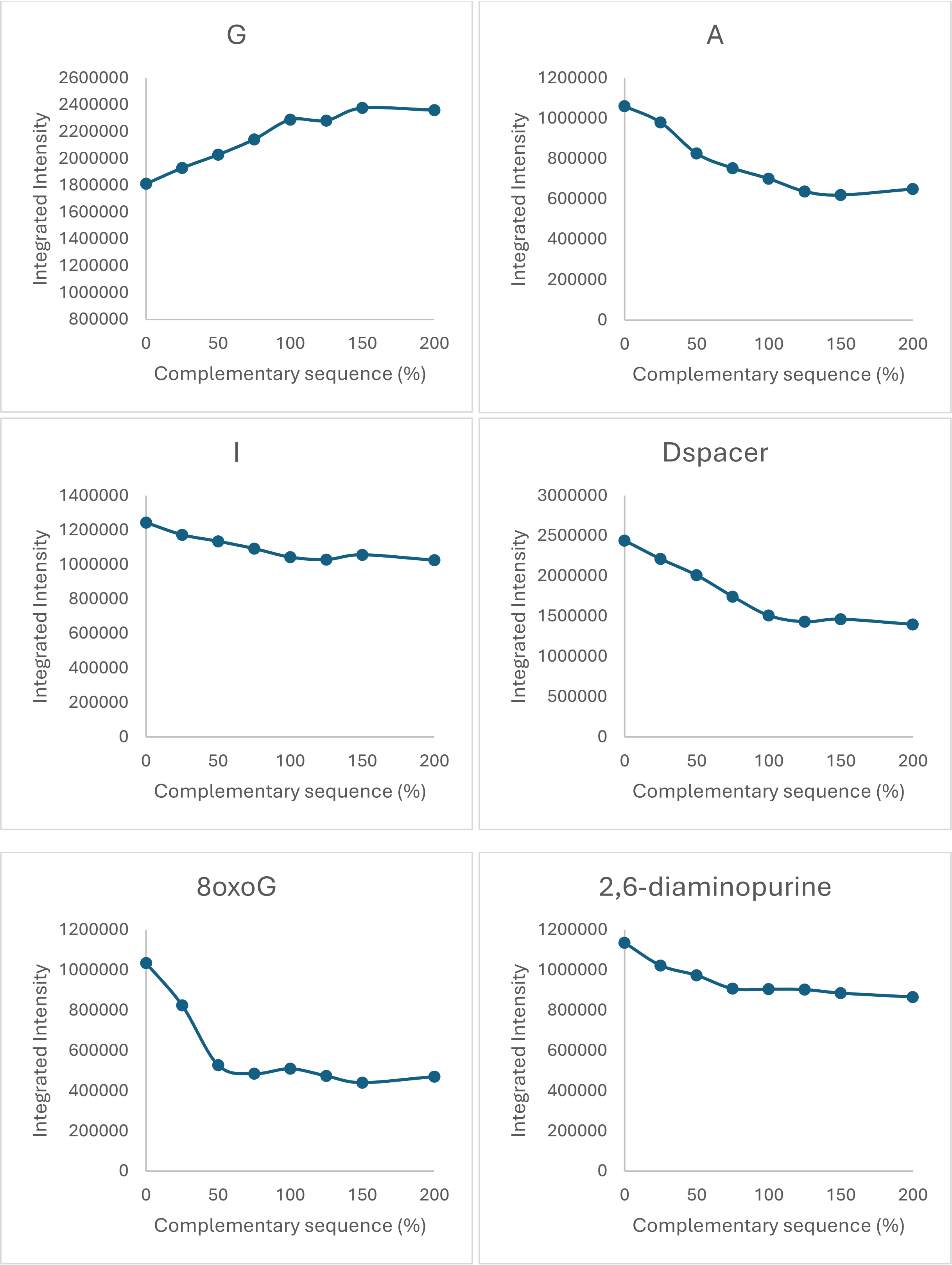
G
A
I
Dspacer
8oxoG
2,6-diaminopurine
Integrated Intensity
Complementary sequence (%)
0
50
100
150
200
2600000
2400000
2200000
2000000
1800000
1600000
1400000
1200000
1000000
800000
600000
400000
200000
3000000
2500000
1500000
500000

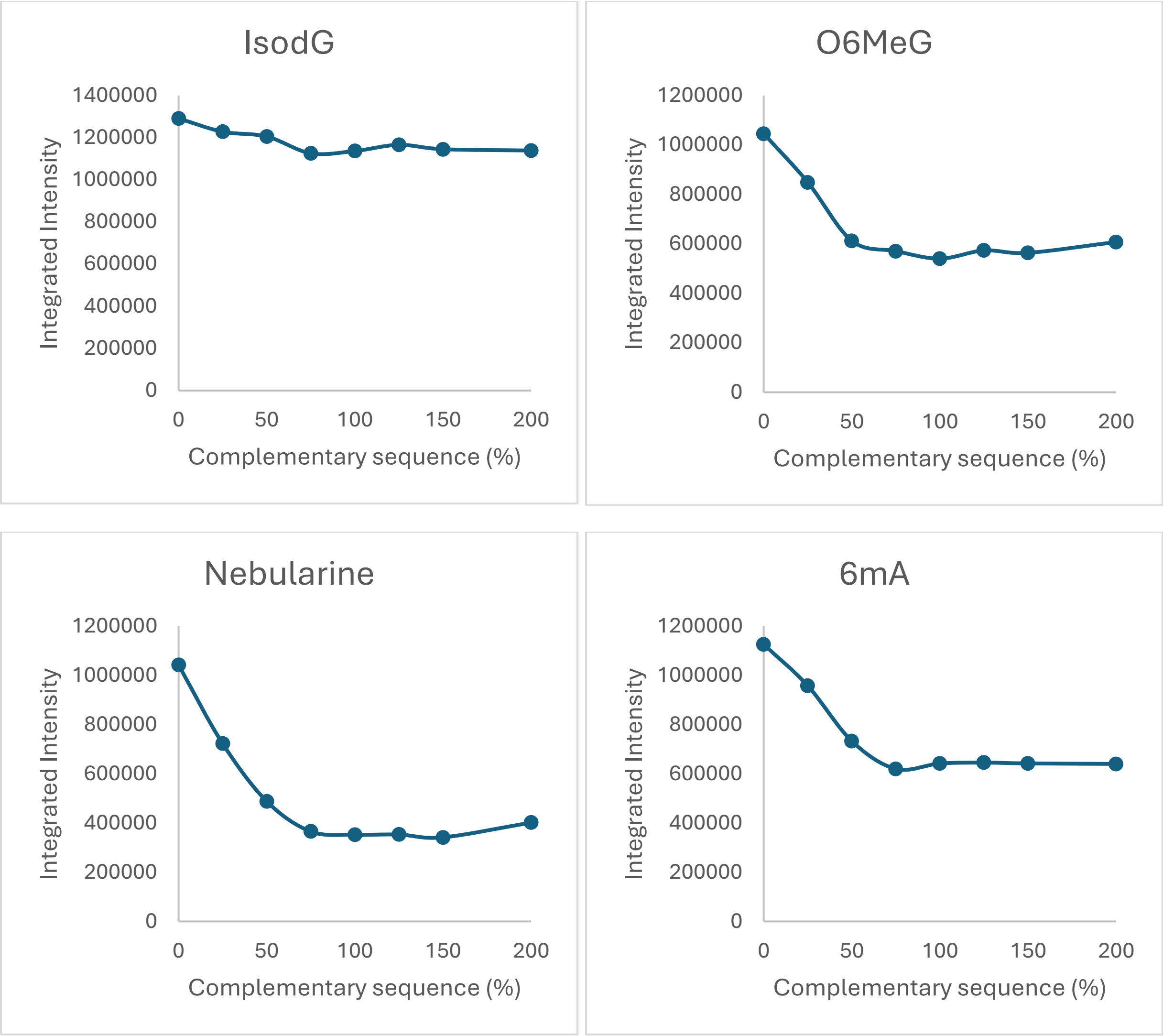

Figure S3. Emission of the ssDNA CXA sequence following titration with complementary modified sequences. Data were recorded using 1× PBS buffer at pH 7.4 with the CXA oligo concentration at 0.72 μM and the excitation wavelength of 395 nm.

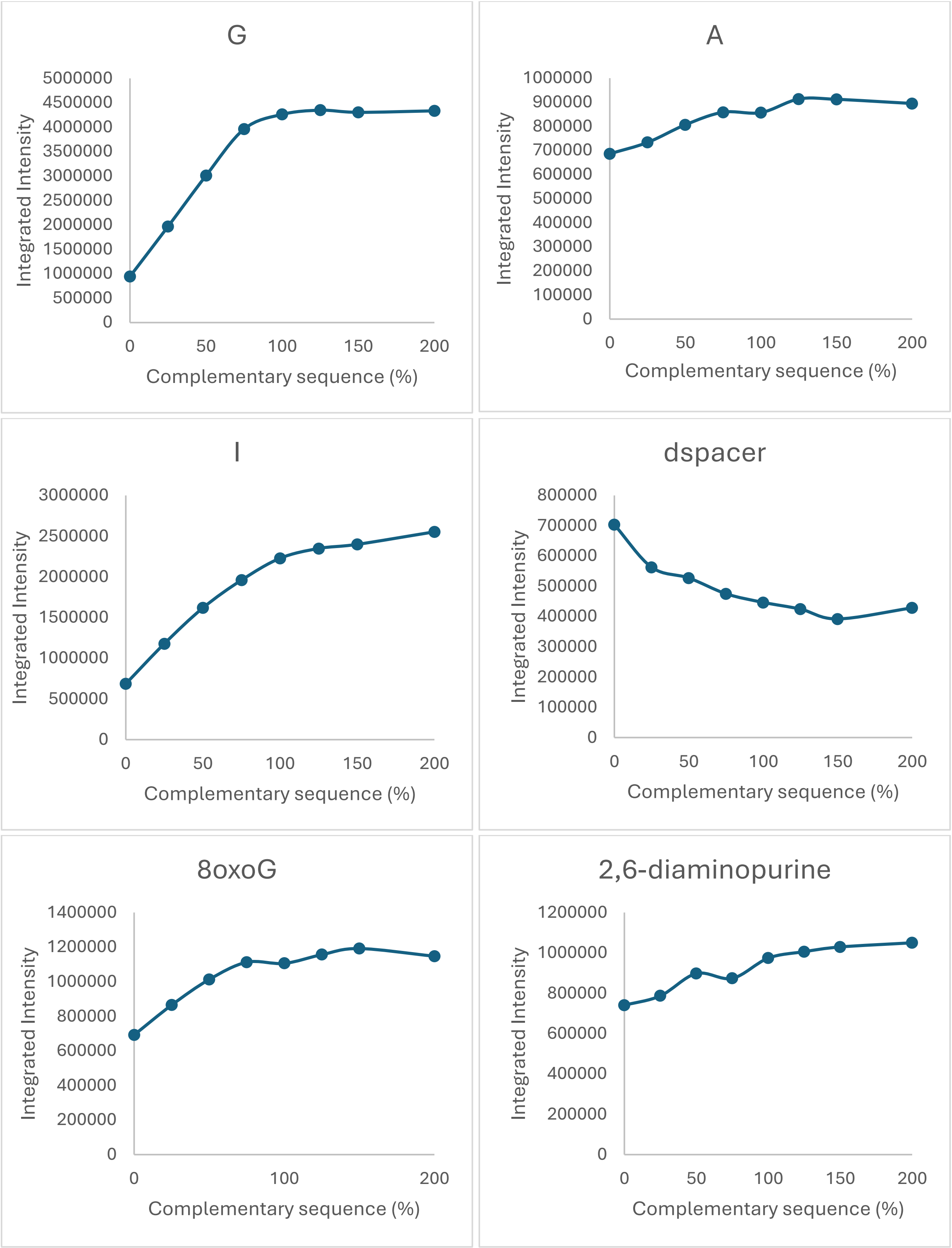
G
Integrated Intensity
5000000
4500000
4000000
3500000
3000000
2500000
2000000
1500000
1000000
500000
0
0 50 100 150 200
Complementary sequence (%)
A
Integrated Intensity
1000000
900000
800000
700000
600000
500000
400000
300000
200000
100000
0
0 50 100 150 200
Complementary sequence (%)
I
Integrated Intensity
3000000
2500000
2000000
1500000
1000000
500000
0
0 50 100 150 200
Complementary sequence (%)
dspacer
Integrated Intensity
800000
700000
600000
500000
400000
300000
200000
100000
0
0 50 100 150 200
Complementary sequence (%)
8oxoG
Integrated Intensity
1400000
1200000
1000000
800000
600000
400000
200000
0
0 100 200
Complementary sequence (%)
2,6-diaminopurine
Integrated Intensity
1200000
1000000
800000
600000
400000
200000
0
0 50 100 150 200
Complementary sequence (%)

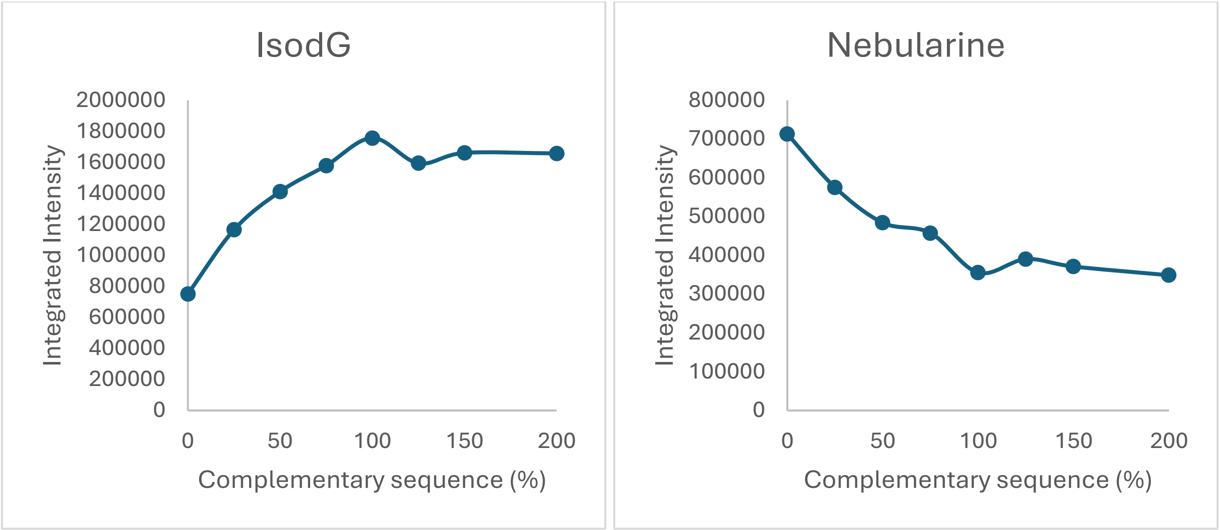

Figure S4. Emission of the ssDNA AXA sequence following titration with complementary modified sequences. Data were recorded using 1× PBS buffer at pH 7.4 with the AXA oligo concentration at 0.72 μM and the excitation wavelength of 395 nm.

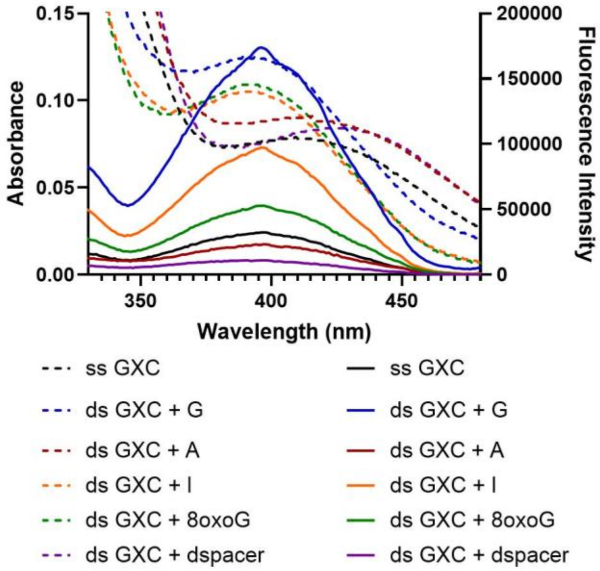

Figure S5. Absorption (dashed) and excitation (solid; collected using an emission wavelength of 500 nm) of the single-stranded (ss) GXC oligonucleotide and hybrid duplexes with ^{DEA}tC base paired with G, A, I, 8-oxo-G, and dSpacer (1′,1′-dideoxyribose).

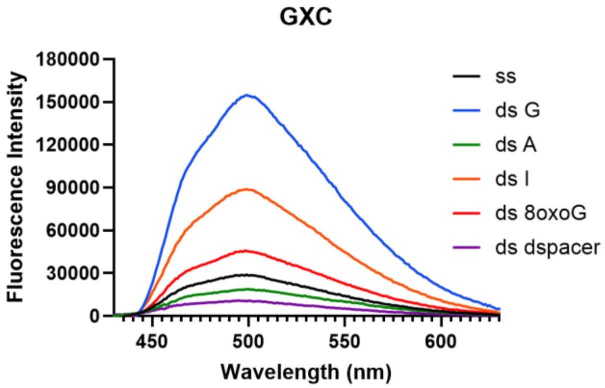

Figure S6. Emission spectra of the single-stranded (ss) GXC oligonucleotide and hybrid duplexes with ^{DEA}tC base paired with G, A, I, 8-oxo-G, and dSpacer (1′,1′-dideoxyribose). Emission spectra were recorded in 1× PBS buffer at pH 7.4 and emission spectra were collected using excitation at 395 nm.

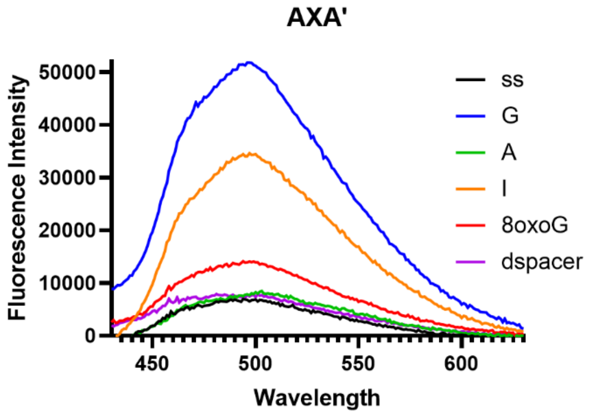

Figure S7. Emission spectra of the single-stranded (ss) AXA' oligonucleotide and hybrid duplexes with ^{DEA}tC base paired with G, A, I, 8-oxo-G, and dSpacer (1′,1′-dideoxyribose). Emission spectra were recorded in 1× PBS buffer at pH 7.4 and emission spectra were collected using excitation at 395 nm.

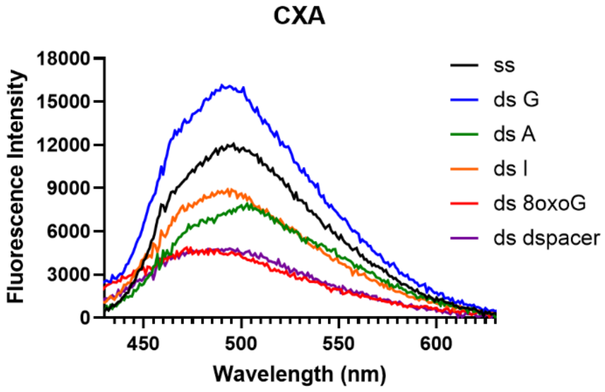

Figure S8. Emission spectra of the single-stranded (ss) CXA oligonucleotide and hybrid duplexes with ^{DEA}tC base paired with G, A, I, 8-oxo-G, and dSpacer (1′,1′-dideoxyribose). Emission spectra were recorded in 1× PBS buffer at pH 7.4 and emission spectra were collected using excitation at 395 nm.

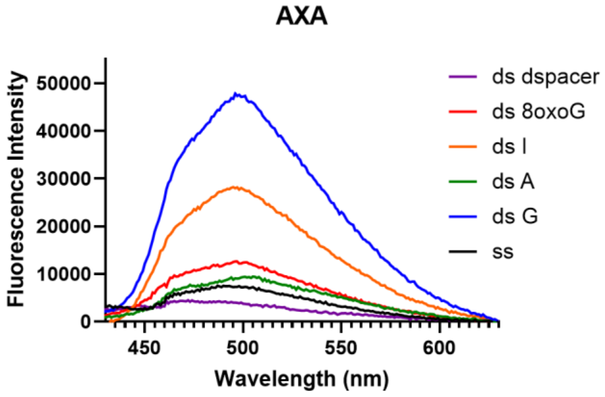

Figure S9. Emission spectra of the single-stranded (ss) AXA oligonucleotide and hybrid duplexes with ^{DEA}tC base paired with G, A, I, 8-oxo-G, and dSpacer (1′,1′-dideoxyribose). Emission spectra were recorded in 1× PBS buffer at pH 7.4 and emission spectra were collected using excitation at 395 nm.

Table S4. Paired sequences and relative fluorescence intensity changes for double-stranded DNA oligonucleotides for the CXA, AXA, GXC and AXA' sequences, where X = ^{DEA}tC.

Bases paired with ^{DEA}tC in dsDNA	CXA (5'-CGCACXATCG-3')	GXC (5'-CGCAGXCTCG-3')	AXA' (5'-CGCAAXATC-3')	AXA (5'-CGCAAXATCG-3')
ss	1	1	1	1
ds G	1.34	5.42	5.53	4.60
ds A	0.66	0.72	1.34	1.30
ds I	0.81	3.24	3.44	3.72
ds 8-oxo-G	0.45	1.53	1.66	1.66
ds d-spacer	0.28	0.32	0.84	0.61
ds 2,6-dap	0.76	1.00	1.27	1.42
ds Iso dG	0.88	1.27	1.99	2.20
ds Nebularine	0.46	0.34	0.96	0.49
ds O6MeG	0.55	0.65	0.97	-
ds 6mA	0.54	0.53	1.10	-

[a] Fluorescence intensity change is reported as the ratio of the emission intensity of each duplex with respect to ss AXA, GXC, CXA and AXA' using 395 nm excitation. [b] ss = single-stranded. [c] ds G refers to the matched complementary duplex with ^{DEA}tC base paired with G; other duplex names follow this convention.

Table S5. Steady-State fluorescence measurements of ^{DEA}tC in single- and double-stranded DNA oligonucleotides for the AXA sequence 5′-CGCATXTTCG-3′, where X = ^{DEA}tC.

Sequence name	λ_{max} Excitation / nm	λ_{max} Emission / nm	Fluorescence intensity change[a]
**ss AXA[b]**	394	494	n/a
**ds G[c]**	396	496	4.60
**ds A**	396	501	1.30
**ds I**	395	495	3.72
**ds 8oxoG**	394	496	1.66
**ds dspacer**	395	488	0.61

[a] Fluorescence intensity change is reported as the ratio of the integrated emission intensity of each duplex with respect to ss AXA using 395 nm excitation. [b] ss = single-stranded.[c] ds G refers to the matched complementary duplex with ^{DEA}tC base paired with G; other duplex names follow this convention.

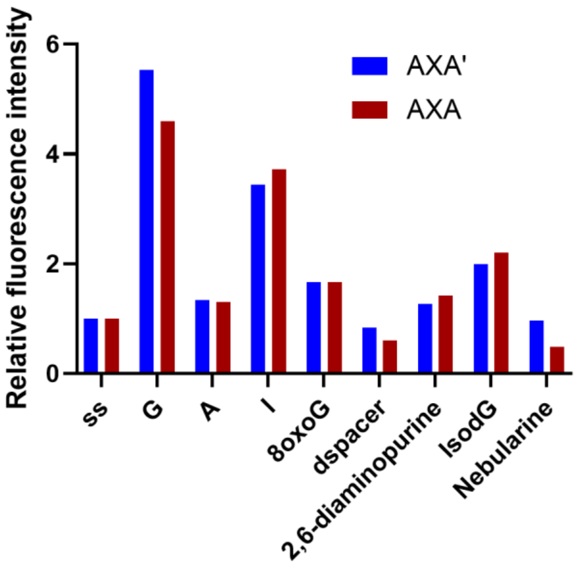

Figure S10. Relative fluorescence intensity of the single-stranded (ss) AXA' and AXA oligonucleotides and their hybrid duplexes with G, A, and noncanonical nucleobases. Fluorescence intensity change is reported as the ratio of the integrated emission intensity of each duplex with respect to the ^{DEA}tC-containing single strand, using 395 nm excitation.

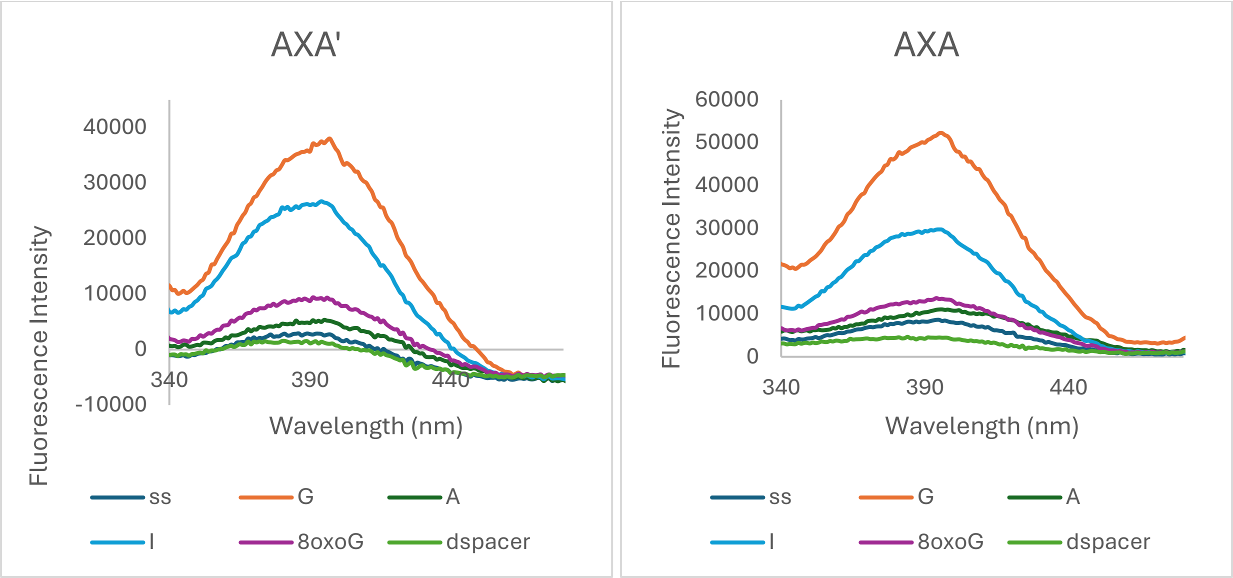

Figure S11. Excitation spectra of the single-stranded (ss) AXA' and AXA oligonucleotide and hybrid duplexes with ^{DEA}tC base paired with G, A, I, 8-oxo-G, and dSpacer (1′,1′-dideoxyribose) collected using an emission wavelength of 500 nm.

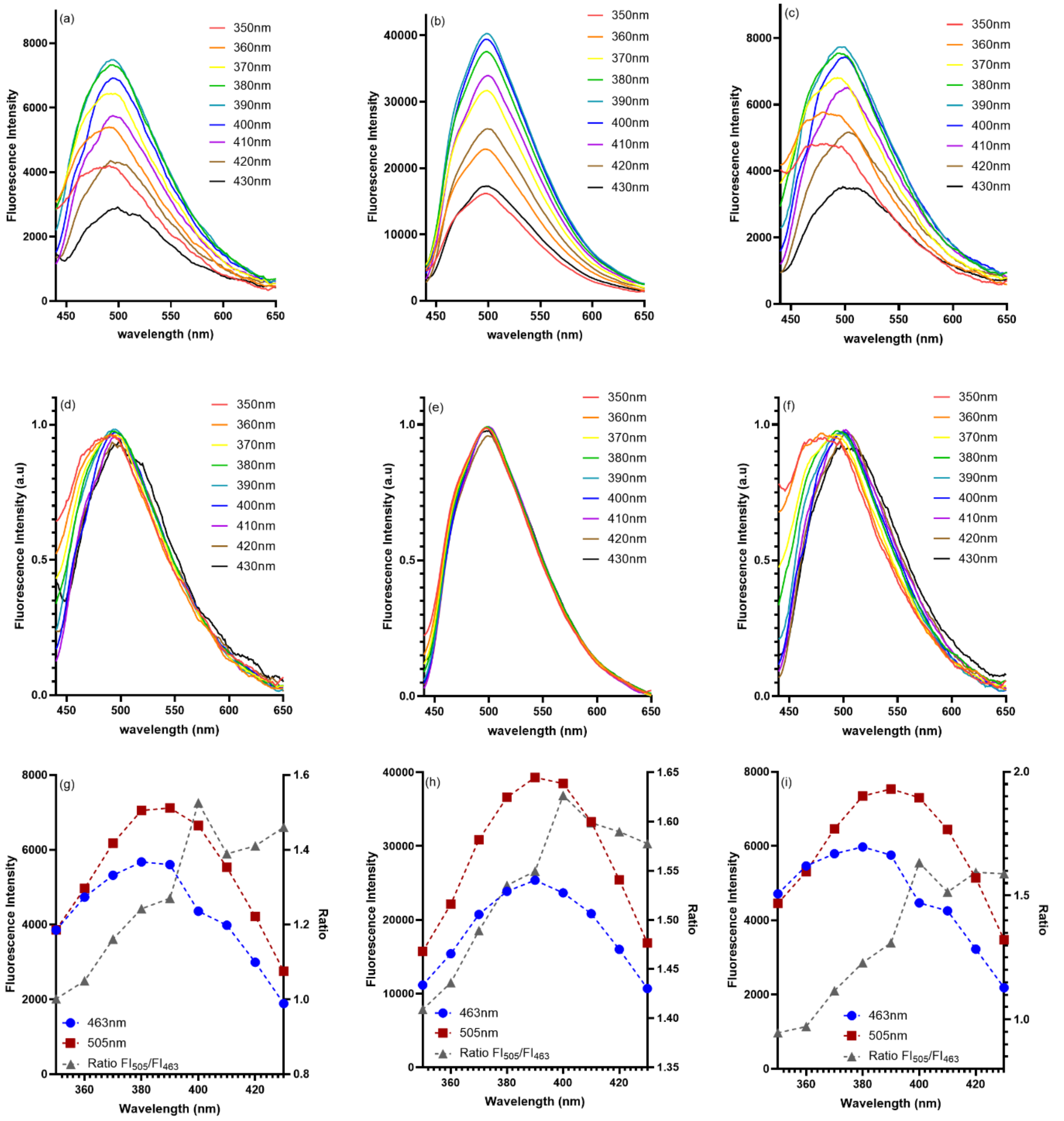

Figure S12. Emission spectra recorded upon excitation at different wavelengths and normalized for the corresponding absorbance intensity (a, b and c) or normalized to unit in intensity (d, e and f) for aqueous solutions of ss AXA’, AXA’ hybrid duplex with ^{DEA}tC paired with G, AXA’ hybrid duplex with ^{DEA}tC paired with A respectively. Extrapolated excitation spectra by plotting the emission spectra intensities at selected wavelengths (463 and 505nm) upon excitation at different wavelengths (g, h and i) of aqueous solutions of ss AXA’, AXA’ hybrid duplex with ^{DEA}tC paired with G, AXA’ hybrid duplex with ^{DEA}tC paired with A

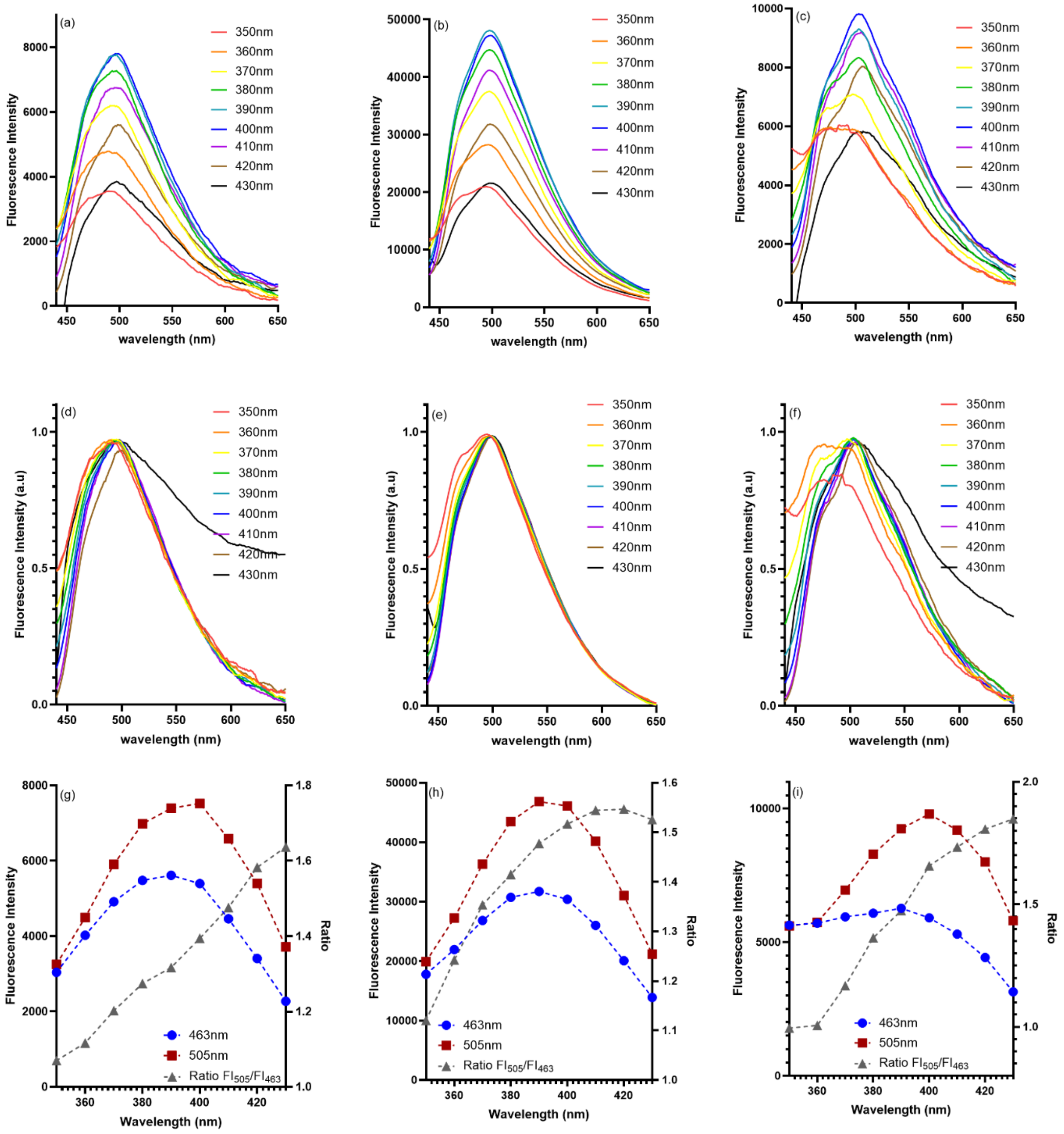

Figure S13. Emission spectra recorded upon excitation at different wavelengths and normalized for the corresponding absorbance intensity (a, b and c) or normalized to unit in intensity (d, e and f) for aqueous solutions of ss AXA, AXA hybrid duplex with ^{DEA}tC paired with G, AXA hybrid duplex with ^{DEA}tC paired with A respectively. Extrapolated excitation spectra by plotting the emission spectra intensities at selected wavelengths (463 and 505nm) upon excitation at different wavelengths (g, h and i) of aqueous solutions of ss AXA, AXA hybrid duplex with ^{DEA}tC paired with G, AXA hybrid duplex with ^{DEA}tC paired with A

Computational Section

Monomer Calculations

^{DEA}tC tautomer models were created in GaussView 6[2] with the approximation that the 1′ carbon was replaced with a methyl group. All subsequent calculations were completed with Gaussian16 version C.01.[3] The monomers were optimized at the B3LYP-D3(BJ)/cc-pVDZ/SMD level, with water as the implicit solvent. Frequency calculations were carried out to ensure that the geometries were at a minimum on the potential energy surface. The resulting monomers were slightly non-planar, with a bend running through nitrogen and sulfur atoms in the central ring of both tautomers. TD-CAM-B3LYP/aug-cc-pVDZ/SMD and TD-B3LYP/aug-cc-pVDZ/SMD single point calculations were carried out on the optimized geometries.

Table S6. Optimized molecular geometries of the C-like and T-like tautomers.

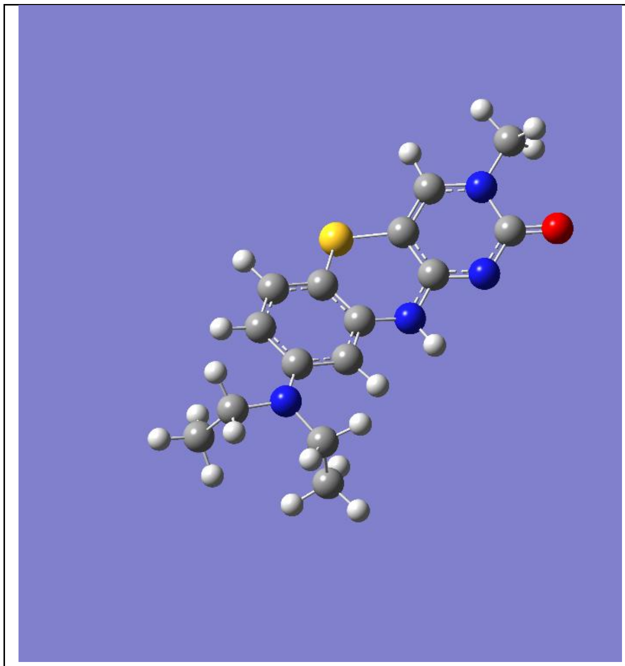	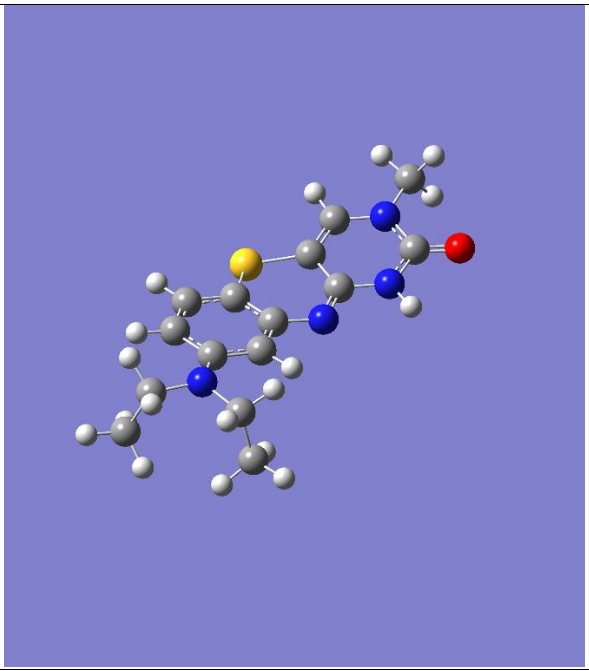
Above: ^{DEA}tC C-like monomer	Above: ^{DEA}tC T-like monomer

Trimer Calculations

An ACA trimer sequence was created with an online tool[4] from the Supercomputing Facility for Bioinformatics and Computational Biology, IIT Delhi, which uses parameters from an experimental study.[5] The cytosine monomer was modified in GaussView 6 to create a model of ^{DEA}tC. The added atoms were made non-planar in the central ring to avoid an imaginary bending mode associated with planarity in the ^{DEA}tC which was found in the monomers. All atoms other than the atoms attached to cytosine were frozen and the model was optimized with a UFF

forcefield. The ^{DEA}tC nucleobase and guanosine were unfrozen, whereas the deoxyriboses were kept frozen. The linked atoms were kept unfrozen so that frequencies could be calculated. The guanosine was manually modified to form adenine or inosine for the base paired sequences with adenine or inosine. The trimer models were optimized with an ONIOM model, with the high layer consisting of B3LYP-D3(BJ)/cc-pVDZ and the low layer consisting of a UFF forcefield. D3(BJ) parameters had to be specified in Gaussian due to the presence of the UFF molecular mechanics method in the ONIOM calculation, and the parameters chosen were the Gaussian 16 default parameters (S8=1.9889, ABJ1=0.3981, ABJ2=4.4211). In the ^{DEA}tC T-like tautomer and adenine base pairing model, self-consistent field (SCF) convergence issues arose due to solvent cavity issues, and the SMD cavity was initially changed from the default van der Waals (vdW) radius to the solvent accessible surface (SAS) for optimization and subsequently changed back to the vdW radius and re-optimized. Finally, single-point ONIOM calculations were carried out with B3LYP-D3(BJ)/aug-cc-pVDZ SMD:UFF, TD-B3LYP-D3(BJ)/aug-cc-pVDZ SMD:UFF, and TD-CAM-B3LYP-D3(BJ)/aug-cc-pVDZ SMD:UFF.

Table S7. Optimized molecular geometries of the C-like and T-like tautomers in base paired and base stacked ONIOM calculations with implicit solvent. Optimizations were carried out at the B3LYP-D3(BJ)/cc-pVDZ/SMD:UFF level with the low level frozen. The low level is depicted below in wireframe.

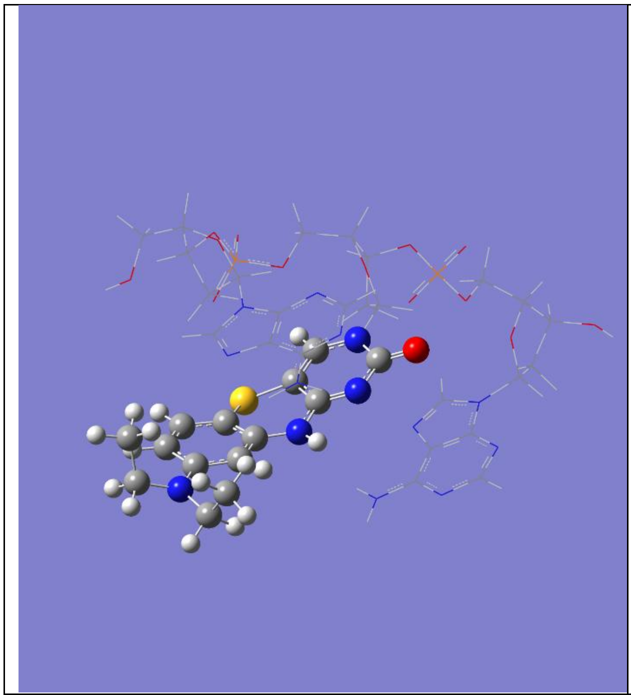	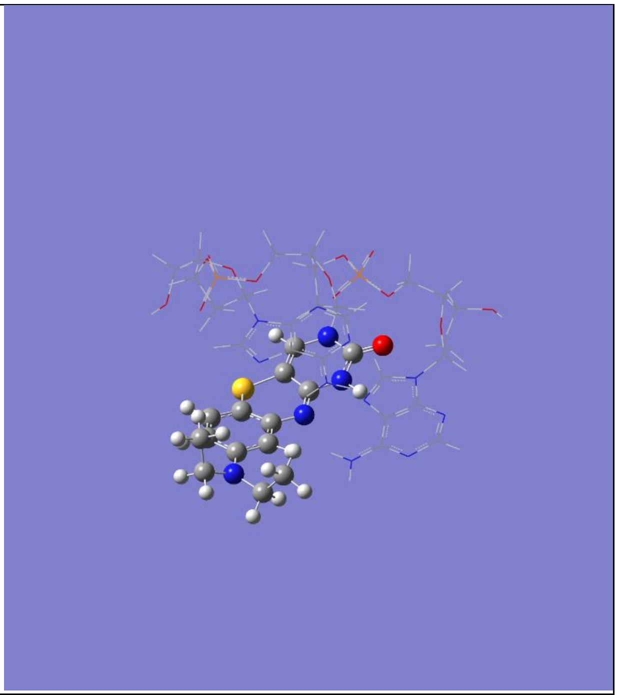
Above: ^{DEA}tC C-like single strand	Above: ^{DEA}tC T-like single strand

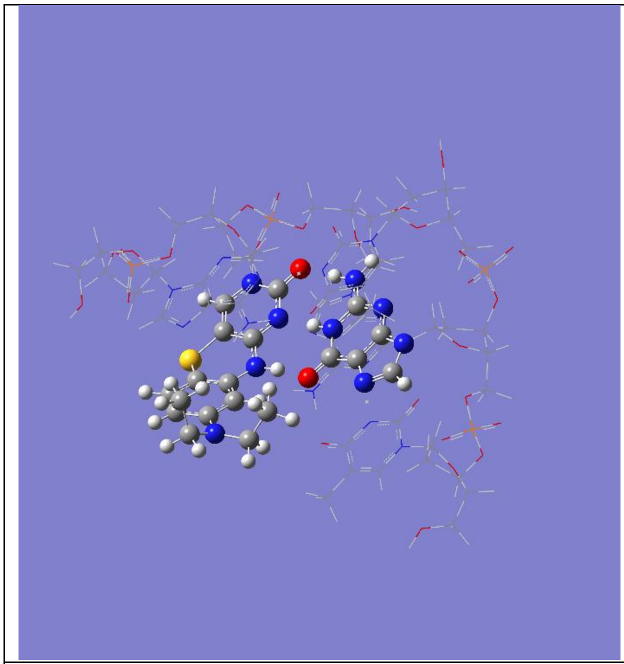

Above: ^{DEA}tC C-like: guanosine double strand

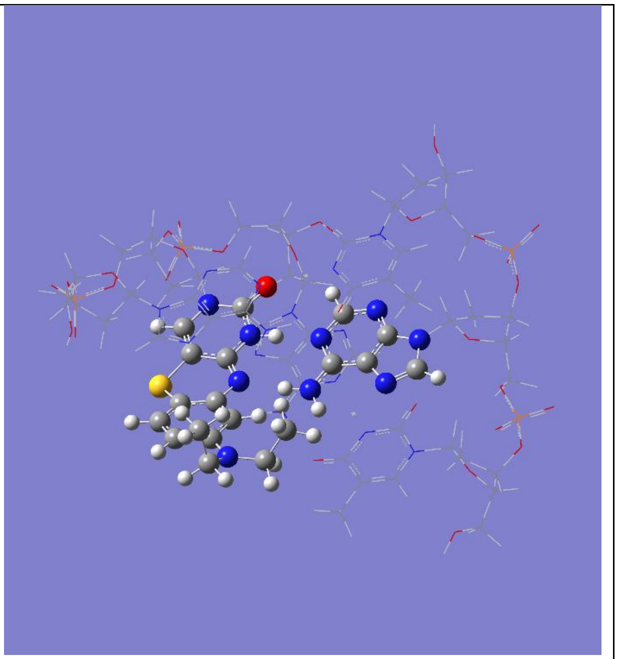

Above: ^{DEA}tC T-like: adenosine double strand

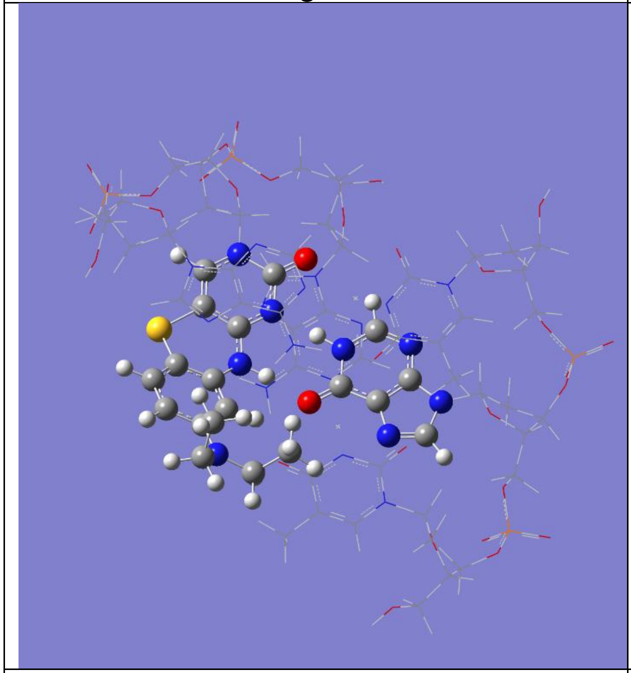

Above: ^{DEA}tC C-like: Inosine double strand

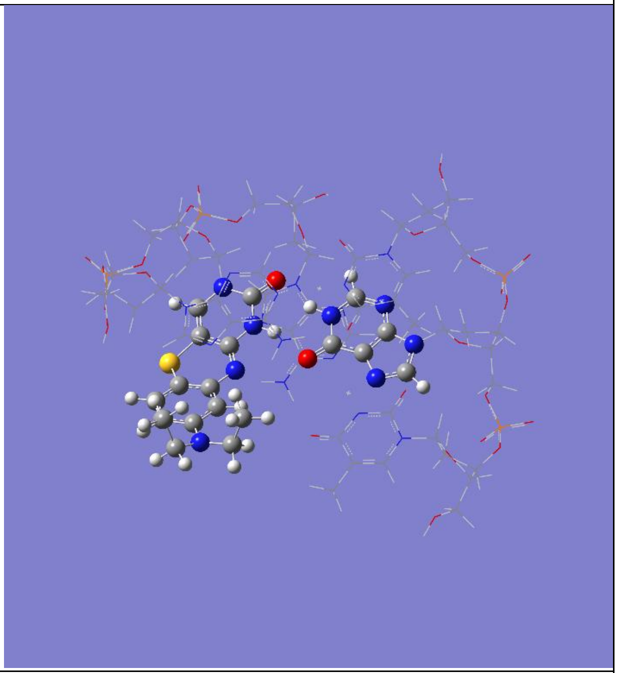

Above: ^{DEA}tC T-like: Inosine double strand

Table S8. Monomer TD-B3LYP/aug-cc-pVDZ/SMD absorption wavelength and oscillator strengths, with water as the implicit solvent. These results show convergence in CAM-B3LYP wavelengths to within 1 nm using an aug-cc-pVDZ basis set.

^{DEA}tC Monomer	TD-B3LYP/cc-pVDZ/SMD $\lambda_{max,abs}$ (nm)	TD-B3LYP/aug-cc-pVDZ/SMD $\lambda_{max,abs}$ (nm)	TD-B3LYP/aug-cc-pVTZ/SMD $\lambda_{max,abs}$ (nm)	TD-B3LYP/cc-pVDZ/SMD oscillator strength	TD-B3LYP/aug-cc-pVDZ/SMD oscillator strength	TD-B3LYP/aug-cc-pVTZ/SMD oscillator strength
C-like	396	419	419	0.0354	0.0254	0.0252
T-like	419	443	443	0.0974	0.083	0.0825

Table S9. Monomer TD-CAM-B3LYP/aug-cc-pVDZ/SMD absorption wavelength and oscillator strengths, with water as the implicit solvent. These results show convergence in TD-CAM-B3LYP wavelengths to within 1 nm using an aug-cc-pVDZ basis set.

^{DEA}tC Monomer	TD-CAM-B3LYP/cc-pVDZ/SMD $\lambda_{max,abs}$ (nm)	TD-CAM-B3LYP/aug-cc-pVDZ/SMD $\lambda_{max,abs}$ (nm)	TD-CAM-B3LYP/aug-cc-pVTZ/SMD $\lambda_{max,abs}$ (nm)	TD-CAM-B3LYP/cc-pVDZ/SMD oscillator strength	TD-CAM-B3LYP/aug-cc-pVDZ/SMD oscillator strength	TD-CAM-B3LYP/aug-cc-pVTZ/SMD oscillator strength
C-like	327	342	343	0.0886	0.0706	0.0663
T-like	361	378	NA	0.2002	0.1774	NA

Table S10. ONIOM and QM calculated absorption wavelengths base paired and base stacked ONIOM models. TD-B3LYP/aug-cc-pVDZ/SMD and TD-CAM-B3LYP/aug-cc-pVDZ/SMD absorption wavelengths, with water as the implicit solvent and a UFF forcefield included in an ONIOM scheme for the low level system. Monomers do not use the ONIOM method.

^{DEA}tC tautomer system	TD-B3LYP/aug-cc-pVDZ/SMD:UFF $\lambda_{max,abs}$ (nm)	TD-B3LYP/aug-cc-pVDZ/SMD $\lambda_{max,abs}$ (nm)	TD-CAM-B3LYP/aug-cc-pVDZ/SMD:UFF $\lambda_{max,abs}$ (nm)	TD-CAM-B3LYP/aug-cc-pVDZ/SMD $\lambda_{max,abs}$ (nm)
^{DEA}tC C-like	NA	419	NA	342
^{DEA}tC T-like	NA	443	NA	378
C-like AXA DNA with backbone	444	NA	356	NA
T-like AXA DNA with backbone	452	452	378	378
C-like:G AXA DNA with backbone	447	447	356	356
T-like:A AXA DNA with backbone	449	450	377	378
C-like:I AXA DNA with backbone	442	442	353	353
T-like:I AXA DNA with backbone	434	434	364	364

Table S11. Relative tautomer stability and electronic energies. B3LYP/aug-cc-pVDZ/SMD single point energies and energy differences with water as the implicit solvent and a UFF forcefield included in an ONIOM scheme for the low level system. Monomers do not use the ONIOM method.

^{DEA}tC	Electronic energy B3LYP_aug-cc-pVDZ//B3LYP/cc-pVDZ (E_h)		Relative energy (kcal mol^{-1})
tautomer	C-like	T-like	(C-like)-(T-like)
monomer	-1275.117414	-1275.107733	-6
AXA DNA ss	-1235.585093	-1235.577553	-5
AXA-G DNA ds	-1691.643756	—	—
AXA-A DNA ds	—	-1616.359322	—
AXA-I DNA ds	-1636.243297	-1636.199295	-28

References

(1) Turner, M. B.; Anderson, B. A.; Samaan, G. N.; Coste, M.; Burns, D. D.; Purse, B. W. Synthesis of Fluorescence Turn-On DNA Hybridization Probe Using the DEA TC 2 - Deoxycytidine Analog. *Curr. Protoc. Nucleic Acid Chem.* **2018**, e59. https://doi.org/10.1002/cpnc.59.

(2) Dennington, Roy; Keith, Todd A.; Millam, John M. *GaussView*, Ver. 6, Semichem Inc., Shawnee Mission, KS, **2016**.

(3) Frisch, M. J.; Trucks, G. W.; Schlegel, H. B.; Scuseria, G. E.; Robb, M. A.; Cheeseman, J. R.; Scalmani, G.; Barone, V.; Petersson, G. A.; Nakatsuji, H.; Li, X.; Caricato, M.; Marenich, A. V.; Bloino, J.; Janesko, B. G.; Gomperts, R.; Mennucci, B.; Hratchian, H. P.; Ortiz, J. V.; Izmaylov, A. F.; Sonnenberg, J. L.; Williams-Young, D.; Ding, F.; Lipparini, F.; Egidi, F.; Goings, J.; Peng, B.; Petrone, A.; Henderson, T.; Ranasinghe, D.; Zakrzewski, V. G.; Gao, J.; Rega, N.; Zheng, G.; Liang, W.; Hada, M.; Ehara, M.; Toyota, K.; Fukuda, R.; Hasegawa, J.; Ishida, M.; Nakajima, T.; Honda, Y.; Kitao, O.; Nakai, H.; Vreven, T.; Throssell, K.; Montgomery, J. A., Jr.; Peralta, J. E.; Ogliaro, F.; Bearpark, M. J.; Heyd, J. J.; Brothers, E. N.; Kudin, K. N.; Staroverov, V. N.; Keith, T. A.; Kobayashi, R.; Normand, J.; Raghavachari, K.; Rendell, A. P.; Burant, J. C.; Iyengar, S. S.; Tomasi, J.; Cossi, M.; Millam, J. M.; Klene, M.; Adamo, C.; Cammi, R.; Ochterski, J. W.; Martin, R. L.; Morokuma, K.; Farkas, O.; Foresman, J. B.; Fox, D. J. Gaussian˜16 Revision C.01, 2016.

(4) *DNA Sequence to Structure*; Supercomputing Facility for Bioinformatics & Computational Biology, IIT Delhi. https://scfbio-iitd.res.in/software/drugdesign/bdna.jsp (accessed 9-13-2025)

(5) Arnott S.; Campbell-Smith P.J.; Chandrasekaran R.; *Nucleic Acids*, Vol. 2; 3rd ed.; Handbook of Biochemistry and Molecular Biology, G.P. Fasman, Ed. Cleveland: CRC Press; 1976; pp. 411-422.
